# Comparative effectiveness of ten CPMs for acute exacerbation of chronic obstructive pulmonary disease: systematic review and network meta-analysis

**DOI:** 10.3389/fmed.2025.1719361

**Published:** 2026-01-15

**Authors:** Keying Li, Wenrui Huang, Tuliang Liang, Xuxin Sun, Minfang Li, Sheng Chen

**Affiliations:** 1 The Fourth Clinical Medical College of Guangzhou University of Chinese Medicine, Shenzhen, Guangdong, China; 2 Shenzhen Traditional Chinese Medicine Hospital, Shenzhen, Guangdong, China

**Keywords:** acute exacerbation of chronic obstructive pulmonary disease, Commercial Chinese Polyherbal Preparations, network meta-analysis, randomized controlled trial, systematic review

## Abstract

**Background:**

Acute exacerbation of chronic obstructive pulmonary disease (AECOPD) is a prevalent acute respiratory disease in China, necessitating effective treatments.

**Purpose:**

To investigate the therapeutic and safety profiles of Chinese patent medicines (CPMs) for AECOPD.

**Methods:**

Randomized controlled trials (RCTs) investigating CPMs for AECOPD were retrieved from PubMed, Cochrane Library, Embase, Web of Science, CNKI, VIP, Wanfang, and CBM. Reviewers independently conducted study selection, data extraction, and bias risk using the Cochrane RoB 2.0. The principal endpoints included total effective rate and pulmonary function parameters; secondary endpoints comprised arterial blood gas indices, inflammatory markers, and adverse events. A frequentist-based analytical strategy was utilized, with StataSE 18.0 for analysis.

**Results:**

84 RCTs comprising 8,477 participants and 10 CPMs were included. ZCL showed the greatest improvement in total effective rate (RR 4.26, 95% CI 2.24–8.09; SUCRA 72.4%). SWLDH ranked highest for FVC (MD 0.58, 95% CI 0.29–0.87; SUCRA 94.5%) and FEV_1_ (MD 0.85, 95% CI 0.56–1.15; SUCRA 98.0%). QKL demonstrated the most significant effect on FEV_1_/FVC (MD 8.57, 95% CI 3.80–13.34; SUCRA 87.0%) and also ranked first for pH. XZL was superior in PaO_2_ and IL-6, while RDN best reduced PaCO_2_ and IL-8. QQHT was most effective for TNF-α. The analysis found no statistically significant increase in the risk of adverse events for any CPM + CT, compared to CT alone. The level of evidence certainty was categorized as low or very low.

**Conclusion:**

CPMs may improve total effective rate, pulmonary function, blood gas parameters, and inflammatory markers in AECOPD. However, these findings require confirmation by high-quality studies.

**Systematic review registration:**

[https://www.crd.york.ac.uk/PROSPERO/view/], identifier [CRD420251059887].

## Graphical Abstract

**Figure:**
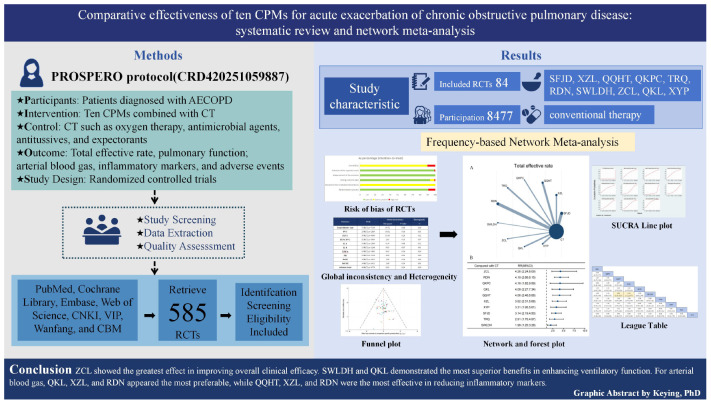
Comparative effectiveness of ten CPMs for AECOPD.

## Highlights

- This is the first PRISMA-based frequentist network meta-analysis comprehensively evaluating different dosage forms of CPMs for AECOPD.
- This study provides latest evidence supporting CPMs efficacy, showing superiority over biomedicine despite low-quality RCTs.
- ZCL, SWLDH, and QKL, QKL, XZL, and RDN, QQHT, XZL and RDN demonstrated outstanding performance in total effective rate, lung function, blood gas parameters, inflammation levels respectively.
- CPMs for phlegm-heat obstructing lung improved efficacy, lung function, blood gas, inflammation but not adverse events.

## 1 Introduction

Acute exacerbation of chronic obstructive pulmonary disease (AECOPD) is defined by a rapid worsening of respiratory symptoms—primarily dyspnea, cough, or sputum—typically within 14 days, frequently accompanied by tachypnea or tachycardia, and often driven by infection-induced local and systemic inflammation (1). The Global Burden of Disease (GBD) study predicts that the number of individuals affected by COPD could rise to approximately 600 million by the year 2050 (2). By 2060, driven by smoking and population aging, annual COPD-related deaths are expected to exceed 5.4 million (3). In the United States, COPD accounts for the second highest burden of disability-adjusted life years (DALYs) among all diseases, generating an economic burden of approximately United States dollar 40 billion annually, with acute exacerbation-related hospitalizations accounting for the largest share (4, 5).

The current treatment for AECOPD primarily relies on inhaled bronchodilators, antibiotics, and corticosteroids. Although these therapeutic modalities can alleviate symptoms, their efficacy remains limited. At the same time, progress in individualized treatment has been relatively slow (6, 7). Inhalation therapy is a crucial approach in the management of AECOPD; however, approximately two-thirds of patients globally exhibit improper use of inhalation devices (8–11). Furthermore, due to suboptimal efficacy and side effects associated with these treatments, patient adherence is generally low (12). Most clinicians resort to empirical medication practices, which often lead to the emergence of multidrug-resistant or pan-resistant bacteria, compromising the effectiveness of antibiotics. Additionally, corticosteroids provide only short-term relief; their long-term use poses a risk for deep fungal infections and thus cannot be considered safe (13). Therefore, it is imperative that we focus on exploring new therapies capable of delaying the progression of AECOPD.

Traditional Chinese medicine (TCM) has garnered substantial clinical experience in the management of AECOPD. With diverse formulations, Commercial Chinese Polyherbal Preparations (CPMs) have demonstrated unique advantages in this field. For example, Tanreqing Injection included in this study is derived from Qingqi Huatan Decoction recorded in Wenre Jingwei and is traditionally indicated for cough and dyspnea caused by phlegm-heat syndrome (14). Animal studies have also indicated that tanreqing capsules alleviate alveolar inflammatory cell infiltration and modulate cytokine expression in AECOPD mouse models (15). Qingke Pingchuan Granules are a modification of Qingjin Huatan Decoction from Jingyue Quanshu, also traditionally used for phlegm-heat obstructing the lung syndrome (16). Clinical trials have shown that qingke pingchuan granules can improve clinical efficacy and reduce inflammatory markers in patients with AECOPD (17), further supporting its traditional use. However, given the wide variety of CPMs, differences in therapeutic efficacy, pharmacokinetics, and adverse effects necessitate individualized clinical assessment.

Although several network meta-analyses have compared the efficacy of CPMs for AECOPD, these studies still have several limitations (18–20): firstly, the number of interventions included was limited, with some studies evaluating only a single CPM and most focusing on injectable formulations, leaving many commonly used forms unassessed; secondly, outcome measures were not comprehensive, as most studies focused solely on lung function or inflammatory parameters, lacking more comprehensive clinical assessment; thirdly, safety evaluations were limited, with some studies reporting only the absence of serious adverse events and lacking systematic classification and stratified analyses. These limitations restrict the ability of existing network meta-analyses to provide comprehensive and reliable evidence for clinical practice.

To address these gaps, the present study systematically reviewed and conducted a network meta-analysis of 84 studies, covering 10 commonly used CPMs across multiple formulations, systematically evaluating efficacy and safety, and ranking the different therapies. In addition, the discussion includes the pharmacological effects of key herbal components, aiming to generate stronger evidence to assist clinicians in formulating individualized treatment strategies through informed decision-making.

## 2 Methods

The present review followed the preferred reporting items for systematic reviews and meta-analyses (PRISMA) guidelines for systematic reviews and meta-analyses, including the extension for network meta-analyses (PRISMA-NMA) (21, 22). The full PRISMA checklist is available in Supplementary Appendix 1. The protocol of this systematic review and network meta-analysis was registered on PROSPERO (CRD420251059887).

### 2.1 Search strategy

We comprehensively retrieved PubMed, Cochrane Library, Embase, Web of Science, China National Knowledge Infrastructure (CNKI), VIP, Wanfang, and Chinese Biomedical Literature Database (CBM) for RCTs evaluating COMs for AECOPD. The literature search spanned from the inception of each database up to May 2025. The search strategy was based on the PICOS framework, using a blend of MeSH words and free-text words, including “AECOPD,” “Chinese patent medicine,” and “randomized controlled trial,” adapted for both English and Chinese databases. Two investigators independently reviewed the literature, and any discrepancies were resolved through discussion with a third reviewer. Additionally, potential studies were additionally identified by screening the reference lists of included articles and relevant reviews. The detailed search strategy is provided in Supplementary Appendix 2.

### 2.2 Eligibility criteria

#### 2.2.1 Population

Patients meeting diagnostic criteria for AECOPD, regardless of age, sex, etiology, race, or severity of pulmonary function impairment.

#### 2.2.2 Intervention and comparison

The control group received conventional treatment (CT), including oxygen therapy, antimicrobial agents, bronchodilators, antitussives, and expectorants, with no restriction on treatment duration. The intervention group received the same conventional treatment combined with one of the following Commercial Chinese Polyherbal Preparations: Shufeng Jiedu Capsules (SFJD), Compound Fresh Bamboo Juice Oral Liquid (XZL), Qingqi Huatan Pills (QQHT), Qingke Pingchuan Granules (QKPC), Tanreqing Injection (TRQ), Reduning Injection (RDN), Shiweilongdanhua Capsules (SWLDH), Zhichuanling Injection (ZCL), Qingkailing Injection (QKL), or Xiyanping Injection (XYP).

#### 2.2.3 Outcomes

The outcomes were categorized into primary and secondary endpoints. Primary outcomes included total effective rate and pulmonary function parameters, such as forced vital capacity (FVC), forced expiratory volume in 1 s (FEV_1_), and the ratio of FEV_1_ to forced vital capacity (FEV_1_/FVC). Secondary outcomes included arterial blood gas indices—PH, arterial partial pressure of oxygen (PaO_2_), and arterial partial pressure of carbon dioxide (PaCO_2_); inflammatory biomarkers—interleukin-6 (IL-6), interleukin-8 (IL-8), and tumor necrosis factor-alpha (TNF-α); and adverse events.

#### 2.2.4 Study design

RCTs published either in domestic or international sources were considered eligible, irrespective of blinding procedures or allocation concealment. All included studies reported complete patient data, and no restrictions were imposed based on publication language.

### 2.3 Study identification and data acquisition

All retrieved records were imported into EndNote 21 for duplicate removal. Firstly, two reviewers independently screened titles and abstracts, including studies with uncertain eligibility for full-text assessment. Secondly, disagreements were resolved through discussion or consultation with a third reviewer. Thirdly, full texts were reviewed based on predefined inclusion criteria to identify eligible studies. Data extraction was conducted independently by two reviewers using a standardized form, with a third reviewer verifying the accuracy and resolving discrepancies. Extracted data included study characteristics (author, publication year), participant details (sample size, age, disease duration), intervention information (drug names, treatment duration for both groups), and outcome measures, including total effective rate (%), pulmonary function parameters [FVC(L), FEV_1_(L), FEV_1_/FVC(%)], arterial blood gas indices [PH, PaO_2_(mmHg), PaCO_2_(mmHg)], inflammatory markers [IL-6(pg/mL), IL-8(pg/mL), TNF-α(pg/Ml)], and adverse event counts. These outcomes were measured at the end of the treatment period, with only a few trials reporting any post-treatment follow-up. Long-term prognostic outcomes, including mortality, mechanical ventilation, length of stay, readmissions, and time to next exacerbation, were also searched. However, these outcomes were reported in too few trials to allow meaningful synthesis.

In addition, total effective rate was standardized across trials according to four-level therapeutic criteria (clinical control, markedly effective, effective, ineffective) commonly used in AECOPD clinical research (23), which was calculated as the proportion of patients classified as clinical control, markedly effective, or effective among all participants.

### 2.4 Appraisal of bias and quality evaluation

We evaluated potential bias across all eligible studies utilizing the Cochrane Risk of Bias 2.0 tool (RoB2.0) (24), encompassing five critical domains: randomization process, deviations from intended interventions, missing outcome data, measurement of outcomes, and selective reporting. Each domain was systematically rated based on signaling questions outlined in the RoB 2.0 guidelines. Studies with low risk across all domains were classified as low risk of bias (score = 1); those with some concerns but no high-risk judgments were rated as having some concerns (score = 2); and those with at least one high-risk domain were considered high risk of bias (score = 3). Two reviewers independently performed the assessments, resolving disagreements through discussion or consultation with a third reviewer when necessary. Additionally, the certainty of evidence was evaluated using the Confidence in Network Meta-Analysis (CINeMA) framework (25, 26), which assesses six dimensions: within-study bias, reporting bias, indirectness, imprecision, heterogeneity, and incoherence. The confidence in each comparison was appraised comprehensively based on both direct and indirect evidence across these domains.

### 2.5 Data synthesis and analysis

A frequentist NMA was conducted using Stata 18.0 to integrate direct and indirect evidence for comprehensive comparative estimates. Network plots were generated to illustrate the relationships among interventions. Effect sizes for dichotomous outcomes—including total effective rate and adverse event incidence—were expressed as risk ratios (RR) with 95% confidence intervals (CI). Continuous outcomes—pulmonary function, blood gas, and inflammatory markers—were analyzed using mean differences (MD) with 95% CIs. Between-study heterogeneity was estimated using the restricted maximum likelihood method and classified by τ^2^ values as low (< 0.04), low-moderate (0.04–0.16), moderate-high (0.16–0.36), or high (> 0.36) (27–29). Global inconsistency was assessed using the design-by-treatment interaction model. The transitivity assumption was evaluated by comparing distributions of patient age and disease duration. Treatment rankings were visualized by cumulative ranking curves and surface under the cumulative ranking curve (SUCRA) plots, where values closer to 1 indicate superior efficacy (30). League tables were constructed to present pairwise comparisons systematically, showing statistical differences and effect estimates between interventions. Sensitivity analysis was used to investigate sources of heterogeneity. Publication bias was evaluated by funnel plots supplemented with linear regression of standardized effect estimates against their standard errors to detect asymmetry.

## 3 Results

### 3.1 Literature retrieval and screening findings

**FIGURE 1:**
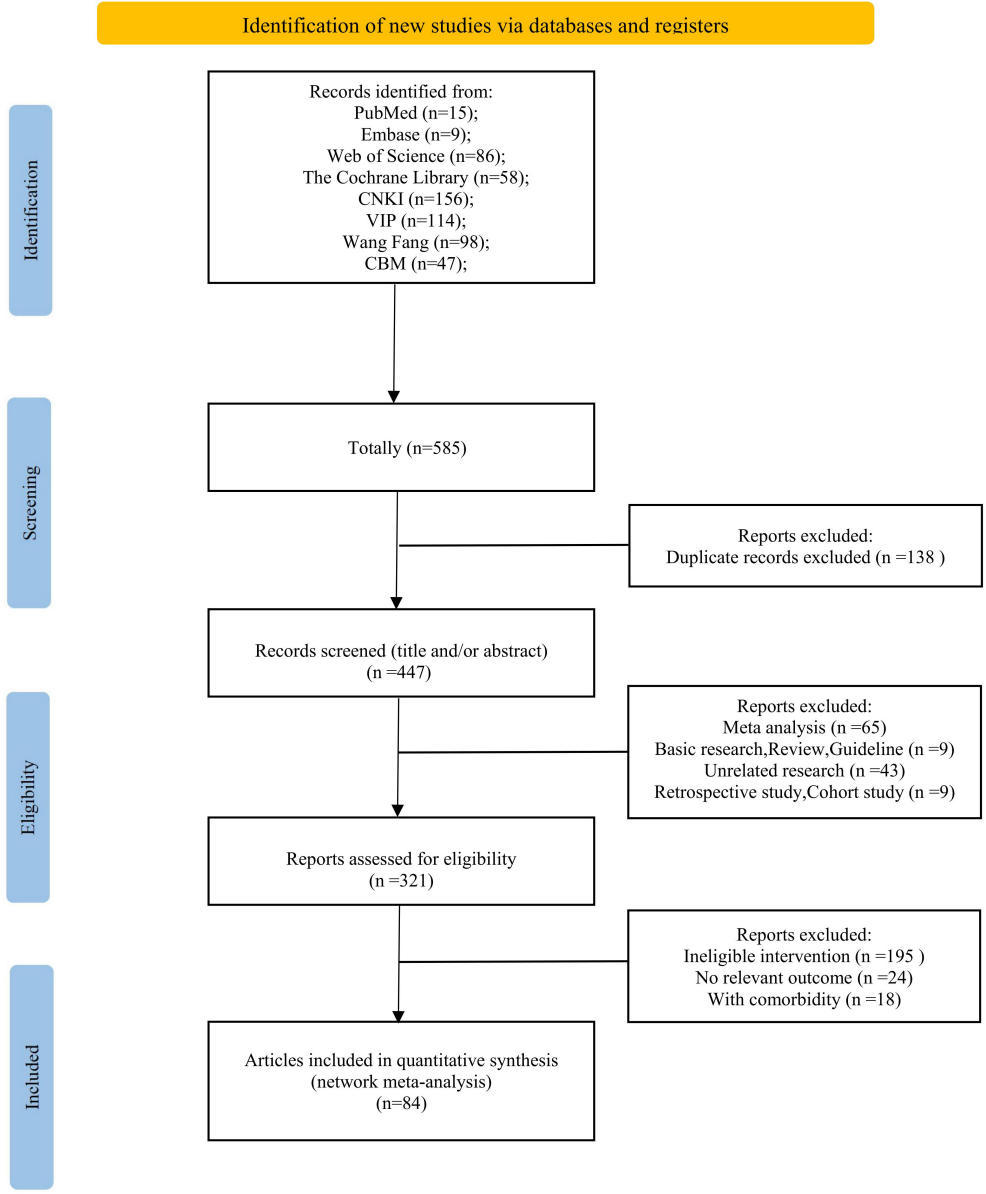
Flow diagram of study selection.

Our comprehensive retrieval collected 585 articles, all managed in EndNote. After removing 114 duplicates, excluding 70 articles through title and abstract screening (including 45 meta-analyses, 1 basic research study, 5 reviews, 18 irrelevant studies, and 1 guideline), full-text evaluation excluded 16 studies with comorbidities, 18 with non-compliant interventions, and 7 retrospective studies, we left eligible randomized controlled trials (Figure 1).

### 3.2 Characteristics of included studies

An aggregate of 84 Chinese-language randomized controlled trials was included, involving 8,477 participants. Patients had a mean age of 62.89 years, standard deviation (SD) 6.44, and an average disease course of 10.19 years (SD 5.28). Treatment duration ranged from 7 to 90 days. Based on the search results, we identified 10 commonly used CPMs, including SFJD, XZL, QQHT, QKPC, TRQ, RDN, SWLDH, ZCL, QKL, and XYP. In the intervention groups, these medicines were combined conventional therapy (CT) with CPMs. The control groups received CT alone. Characteristics of the included studies were detailed in Supplementary Table S3.1.

Component analysis of the 10 CPMs revealed that *Ephedra wraithiana* I. M. Johnst (Mahuang), *Scutellaria baicalensis* Georgi (Huangqin), *Prunus sibirica* L (Xingren), *Pinellia ternata* (Thunb.) Makino (Banxia), *Citrus japonica* Thunb (Chenpi), and *Glycyrrhiza uralensis* Fisch. ex DC (Gancao) appeared most frequently. These herbs commonly possess properties of clearing lung heat, relieving cough and asthma, and regulating qi with phlegm resolution—actions particularly effective for alleviating core symptoms such as cough, purulent sputum, and chest tightness. Botanical names were verified through World Flora Online^1^ and the Medicinal Plant Names Services (MPNS).^2^ Detailed information on traditional functions, active components, chemical constituents, and pharmacological action of these herbs was presented in Supplementary Table S3.2. Details of the suppliers, batch numbers, specification, prescription dosages, quality control contents, and preparation methods of Commercial Chinese Polyherbal Preparations were provided in Supplementary Table S3.3. Due to proprietary or regulatory reasons, the exact content of some herbal ingredients was not publicly disclosed.

### 3.3 Risk of bias, evidence quality, and network reliability assessment

The bias assessment outcomes for all included RCTs are documented in Supplementary Appendix 4. Among the 84 included studies, 76 (90.5%) were rated as low risk regarding random sequence generation, while all studies (100.0%) presented potential concerns regarding deviations from intended interventions and showed low risk in outcome measurement. A low risk of bias due to missing outcome data was observed in 79 studies (94.0%), and 83 (98.8%) were at low risk for selective reporting. 75 studies (89.3%) were rated as having some concerns, whereas 9 studies (10.7%) were deemed to be at high risk of bias, primarily due to insufficient reporting on blinding procedures for participants, investigators, or outcome assessors. Inconsistency analysis identified significant inconsistency for the PH outcome (*P* < 0.05), suggesting the need for future high-quality randomized trials. The τ^2^ estimates were generally low to moderate; however, higher heterogeneity was noted for FEV_1_/FVC, PaO_2_, and PaCO_2_, possibly related to variations in treatment duration and dosage (Supplementary Appendix 5).

We used CINeMA to evaluate the quality of evidence, which showed the confidence in all pairwise comparisons was very low to low (Supplementary Appendix 6). Based on the available study-level characteristics, no major violations of the transitivity assumption were identified across the networks; however, the assessment was limited by incomplete reporting of several potential effect modifiers (Supplementary Table S6.1). Publication bias was evaluated using funnel plots (Supplementary Appendix 7).

### 3.4 Total effective rate

**FIGURE 2:**
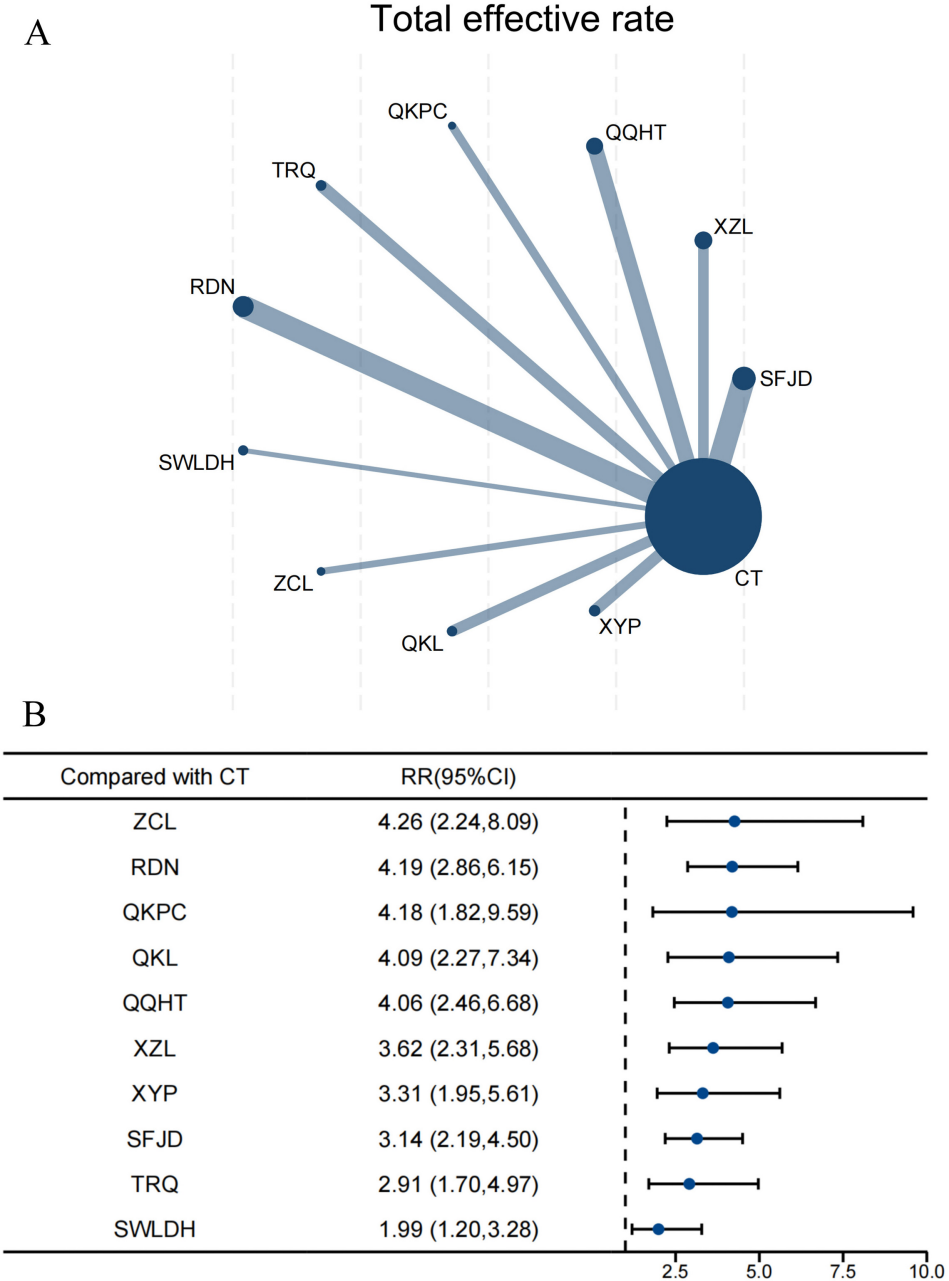
Network and forest plots for total effective rate. **(A)** Comparative network of CPMs and CT in AECOPD, where node size indicates the number of participants per group, and edge thickness represents the number of trials per comparison. **(B)** Forest plot summarizing network estimates of effect size for total effective rate across CPMs and CT.

For the total effective rate, this NMA included 73 trials involving a total of 7,339 participants. The network diagram shows that 10 CPMs combined with CT were directly compared with CT alone, with RDN, QQHT, and SFJD being the most common (Figure 2A). The forest plot indicates that, compared with CT alone, all 10 CPMs combined with CT significantly improved the total effective rate (Figure 2B). Among these, ZCL (RR 4.26, 95% CI 2.24–8.09, SUCRA 72.4%, low confidence in evidence) most significantly improved the total effective rate, followed by RDN (4.19, 95% CI 2.86–6.15, SUCRA 70.2%, low certainty of evidence) (Supplementary Table S6.2; Supplementary Figure S8.1). Further comparisons showed that RDN (2.11, 95% CI 1.12–3.97, SUCRA 70.2%, very low certainty of evidence) and QQHT (2.04, 95% CI 1.01–4.15, SUCRA 67.8%, very low certainty of evidence) were superior to SWLDH in improving the total effective rate (Supplementary Table S10.1). Evidence quality, as assessed by CINeMA, was predominantly categorized as low or very low (Supplementary Table S6.2).

### 3.5 Pulmonary function parameters

#### 3.5.1 FVC

**FIGURE 3:**
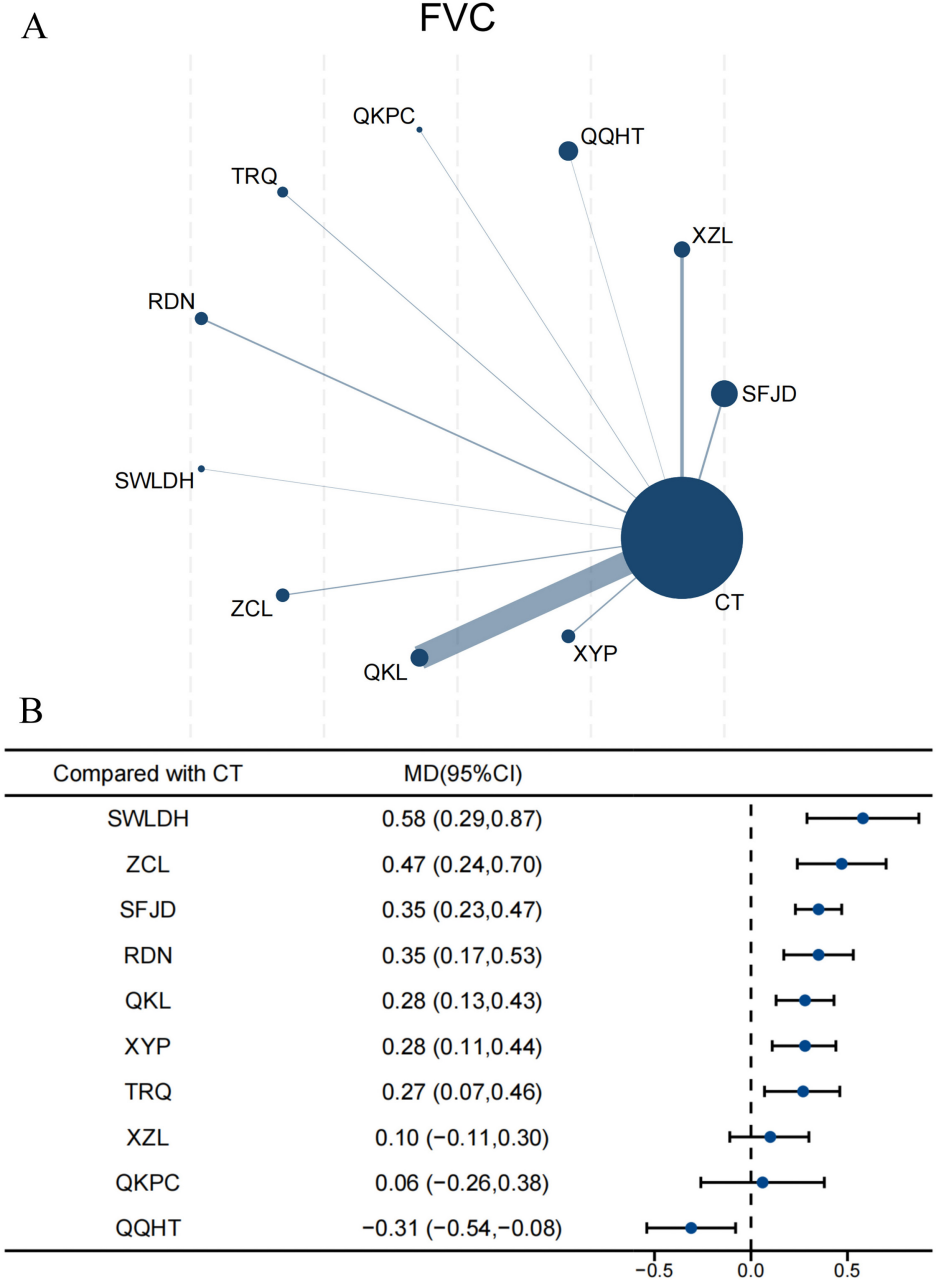
Network and forest plots for FVC. **(A)** Comparative network of CPMs and CT in AECOPD, where node size indicates the number of participants per group, and edge thickness represents the number of trials per comparison. **(B)** Forest plot summarizing network estimates of effect size for FVC across CPMs and CT.

For FVC, 25 RCTs involving 2,805 participants were enrolled in the NMA. All CPMs combined with CT were compared against CT alone, with QKL being the most frequently studied (Figure 3A). The results showed all CPMs combined with CT, except XZL, QKPC, and QQHT, significantly improved FVC compared with CT alone (Figure 3B). SWLDH yielded the most significant improvement (MD 0.58, 95% CI 0.29–0.87; SUCRA 94.5%; low confidence), followed by ZCL (0.47, 95% CI 0.24–0.70; SUCRA 85.9%; low confidence) (Supplementary Table S6.3; Supplementary Figure S8.2). Indirect comparisons suggested that SFJD (0.25, 95% CI 0.02–0.49; SUCRA 69.6%; very low confidence), SWLDH (0.48, 95% CI 0.13–0.84; SUCRA 94.5%; very low confidence), and ZCL (0.37, 95% CI 0.07–0.68; SUCRA 85.9%; very low confidence) were superior to XZL. Additionally, SWLDH (0.52, 95% CI 0.09–0.95; SUCRA 94.5%; very low confidence) and ZCL (0.41, 95% CI 0.01–0.80; SUCRA 85.9%; very low confidence) were better than QKPC. Except for QKPC, the other eight CPMs showed superiority over QQHT (Supplementary Table S10.2). Evidence quality, as assessed by CINeMA, was predominantly categorized as low or very low (Supplementary Table S6.3).

#### 3.5.2 FEV_1_

**FIGURE 4:**
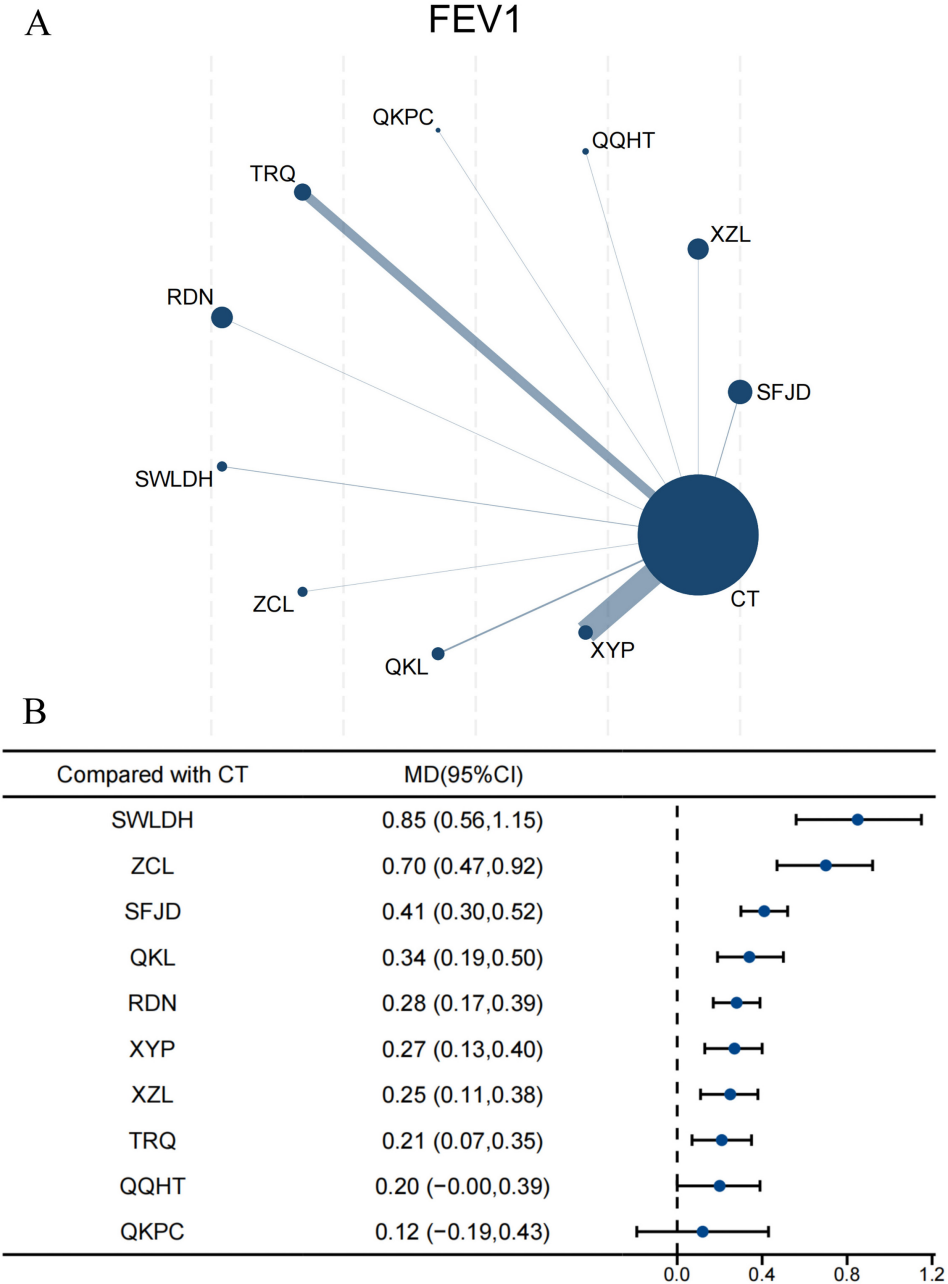
Network and forest plots for FEV_1_. **(A)** Comparative network of CPMs and CT in AECOPD, where node size indicates the number of participants per group, and edge thickness represents the number of trials per comparison. **(B)** Forest plot summarizing network estimates of effect size for FEV_1_ across CPMs and CT.

For FEV_1_, 39 RCTs involving 4,090 participants were enrolled in this NMA. Ten CPMs combined with CT had direct comparisons with CT alone, with XYP being the most frequently studied (Figure 4A). The forest plot showed all CPMs except QQHT and QKPC significantly improved FEV_1_ compared with CT alone (Figure 4B). SWLDH demonstrated the most significant improvement in FEV_1_ (MD 0.85, 95% CI 0.56–1.15; SUCRA 98.0%; very low confidence), followed by ZCL (0.70, 95% CI 0.47–0.92; SUCRA 91.7%; very low confidence) (Supplementary Table S6.4; Supplementary Figure S8.3). Indirect comparisons revealed SFJD (0.21, 95% CI 0.03–0.38; SUCRA 75.7%; very low confidence) outperformed TRQ in improving FEV_1_. Apart from ZCL, SWLDH showed superiority over the other eight CPMs. Likewise, except for SWLDH, ZCL was superior to the other eight CPMs (Supplementary Table S10.3). The certainty of evidence, as assessed by CINeMA, was predominantly rated as very low (Supplementary Table S6.4).

#### 3.5.3 FEV_1_/FVC

**FIGURE 5:**
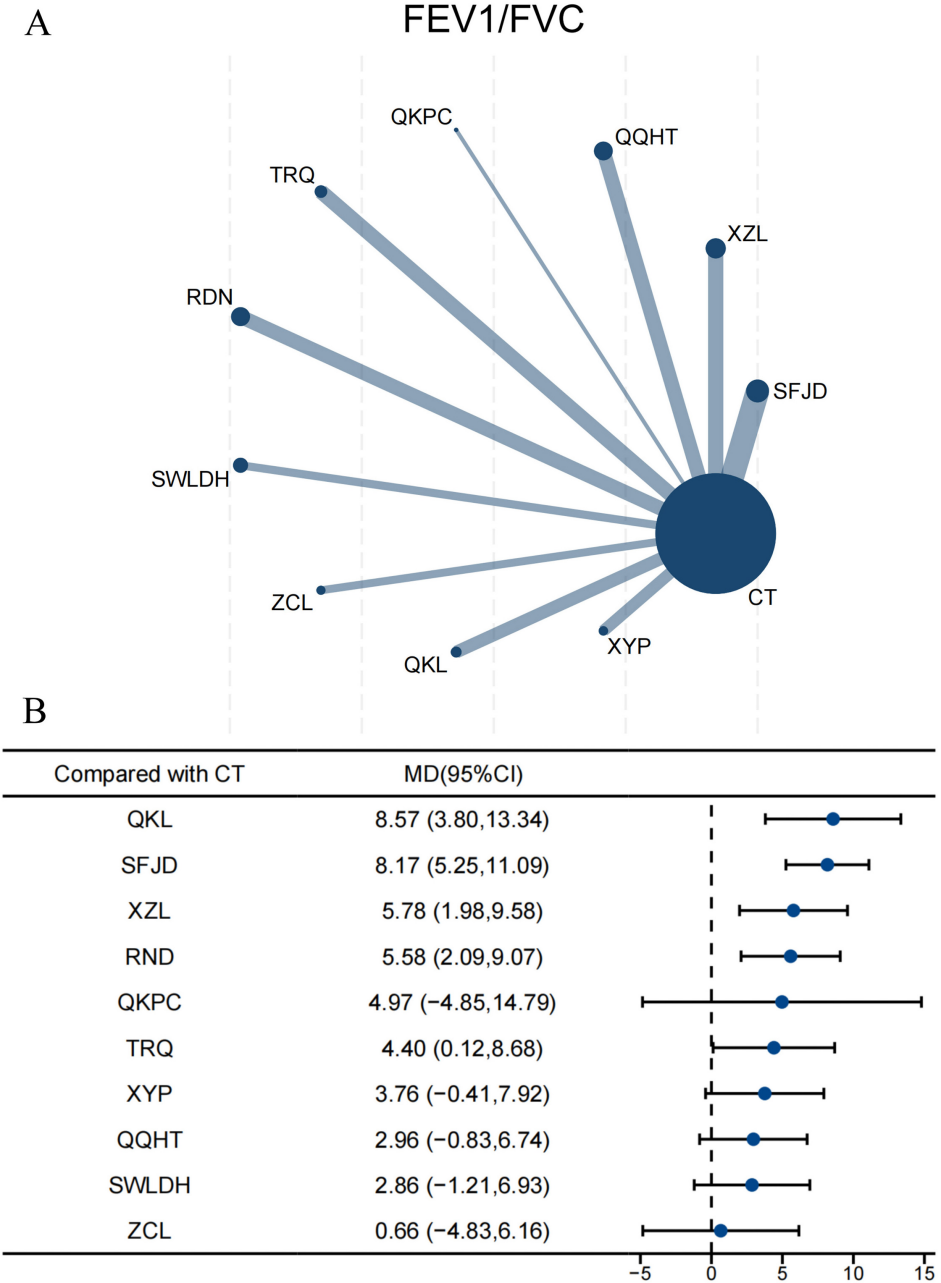
Network and forest plots for FEV_1_/FVC. **(A)** Comparative network of CPMs and CT in AECOPD, where node size indicates the number of participants per group, and edge thickness represents the number of trials per comparison. **(B)** Forest plot summarizing network estimates of effect size for FEV_1_/FVC across CPMs and CT.

For FEV_1_/FVC, 52 RCTs comprising 5,904 participants were incorporated into the NMA. Ten CPMs combined with CT were directly compared with CT alone, with SFJD being the most frequently investigated (Figure 5A). The forest plot indicated that, compared with CT alone, QKL, XZL, and RDN significantly improved FEV_1_/FVC (Figure 5B). QKL demonstrated the most pronounced improvement (MD 8.57, 95% CI 3.80–13.34; SUCRA 87.0%; very low confidence), followed by SFJD (8.17, 95% CI 5.25–11.09; SUCRA 86.9%; very low confidence) (Supplementary Table S6.5; Supplementary Figure S8.4). Indirect comparisons showed that both QKL (7.91, 95% CI 0.63–15.18; SUCRA 87.0%; very low confidence) and SFJD (7.51, 95% CI 1.29–13.73; SUCRA 86.9%; very low confidence) were superior to ZCL. Moreover, SFJD also showed better efficacy than SWLDH and QQHT (Supplementary Table S10.4). According to the CINeMA assessment, the certainty of the evidence was predominantly classified as very low (Supplementary Table S6.5).

### 3.6 Blood gas parameters

#### 3.6.1 PH

For PH, 10 RCTs involving 1,216 participants were included in the NMA. The network plot showed direct comparisons between five CPMs combined with CT and CT alone, with RDN being the most frequently assessed (Supplementary Figure S9.1A1). Both QKL (MD 0.09, 95% CI 0.01–0.17; SUCRA 90.3%; very low confidence) and RDN (0.06, 95% CI 0.03–0.09; SUCRA 76.7%; very low confidence) significantly improved PH, with QKL showing the greatest effect (Supplementary Figures S8.5, S9.1A2 and Supplementary Table S6.6). Indirect comparisons among the CPMs revealed no statistically significant differences (Supplementary Table S10.5). The quality of evidence, as evaluated by the CINeMA approach, was determined to be very low (Supplementary Table S6.6).

#### 3.6.2 PaO_2_

For PaO_2_, 34 RCTs involving 3,613 participants were included in the NMA. Nine CPMs combined with CT were directly compared with CT alone, with QQHT and RDN being the most frequently studied (Supplementary Figure S9.2B1). The forest plot indicated XZL, RDN, QKL, and QQHT significantly increased PaO_2_ levels (Supplementary Figure S9.2B2). XZL showed the greatest improvement (MD 17.68, 95% CI 11.10–24.26; SUCRA 98.2%; low confidence), followed by RDN (9.96, 95% CI 4.62–15.30; SUCRA 73.4%; low confidence) (Supplementary Table S6.7; Supplementary Figure S8.6). Indirect comparisons revealed that XZL was superior to SFJD, QQHT, QKPC, TRQ, and XYP (Supplementary Table S10.6). The CINeMA evaluation rated the certainty of the evidence as ranging from very low to low (Supplementary Table S6.7).

#### 3.6.3 PaCO_2_

For PaCO_2_, 34 RCTs involving 3,613 participants were included in the NMA. Nine CPMs combined with CT were directly compared with CT alone, with QQHT and RDN being the most frequently investigated (Supplementary Figure S9.3C1). The forest plot showed all CPMs except SWLDH and XYP significantly reduced PaCO_2_ levels compared with CT (Supplementary Figure S9.3C2). RDN demonstrated the most pronounced effect (MD −10.38, 95% CI −14.46 to −6.29; SUCRA 83.0%; very low confidence), followed by TRQ (−10.16, 95% CI −15.01 to −5.30; SUCRA 80.2%; very low confidence) (Supplementary Table S6.8, Supplementary Figure S8.7). Indirect comparisons showed no statistically significant differences among the CPMs (Supplementary Table S10.7). The total certainty of evidence assessed by CINeMA was predominantly deemed as very low (Supplementary Table S6.8).

### 3.7 Inflammatory markers

#### 3.7.1 IL-6

For IL-6, 18 RCTs involving 2,050 participants were included in the NMA. Nine CPMs combined with CT were directly compared with CT alone, with TRQ being the most frequently studied (Supplementary Figure S9.4D1). Only XZL (MD −26.89, 95% CI −51.50 to −2.29; SUCRA 83.5%; low confidence) and SWLDH (−26.40, 95% CI −51.51 to −1.79; SUCRA 83.0%; low confidence) significantly reduced IL-6 levels compared with CT alone, with XZL showing the greatest effect (Supplementary Table S6.9; Supplementary Figures S8.8, S9.4D2). Indirect comparisons in CPMs showed no statistically significant differences (Supplementary Table S10.8). The total certainty of evidence assessed by CINeMA was deemed as very low to low (Supplementary Table S6.9).

#### 3.7.2 IL-8

For IL-8, 15 RCTs involving 1,266 participants were included in the NMA. Five CPMs combined with CT were directly compared with CT, with QKL being the most frequently studied (Supplementary Figure S9.5E1). The forest plot showed RDN, SFJD, and QKL significantly reduced IL-8 levels (Supplementary Figure S9.5E2). RDN demonstrated the greatest reduction (MD −12.33, 95% CI −20.71 to −3.95; SUCRA 87.9%; very low confidence), followed by SFJD (MD −7.82, 95% CI −14.53 to −1.11; SUCRA 62.7%; very low confidence) (Supplementary Table S6.10; Supplementary Figure S8.9). Indirect comparisons among the CPMs showed no statistically significant differences (Supplementary Table S10.9). The certainty of evidence assessed by CINeMA was predominantly judged to be very low (Supplementary Table S6.10).

#### 3.7.3 TNF-α

For TNF-α, 19 RCTs involving 1,863 subjects were included in the NMA. Seven CPMs combined with CT were directly compared with CT alone, with ZCL being the most frequently investigated (Supplementary Figure S9.6F1). The forest plot showed QQHT, SFJD, TRQ, and ZCL significantly reduced TNF-α levels compared with CT alone (Supplementary Figure S9.6F2). QQHT showed the strongest effect (MD −7.28, 95% CI −12.37 to −2.19; SUCRA 78.6%; low confidence), followed by SFJD (−6.31, 95% CI −9.45 to −3.17; SUCRA 72.0%; low confidence) (Supplementary Table S6.11; Supplementary Figure S8.10). Indirect comparisons revealed SFJD (MD −5.83, 95% CI −11.55 to −0.11; SUCRA 72.0%; low confidence) was superior to RDN (Supplementary Table S10.10). The certainty of evidence assessed by CINeMA was rated as very low to low (Supplementary Table S6.11).

### 3.8 Adverse event

For adverse events, the NMA included 44 RCTs with 4,776 participants. The network plot showed direct comparisons between 10 CPMs combined with CT and CT alone, with SFJD and RDN being the most frequently compared (Supplementary Figure S9.7G1). The forest plot indicated no statistically significant differences among the 10 CPMs in reducing adverse events compared with CT alone (Supplementary Figure S9.7G2), as did indirect comparisons (Supplementary Table S10.11). The general level of evidence certainty assessed by CINeMA was predominantly judged to be very low (Supplementary Table S6.12).

Nausea, vomiting, and diarrhea were the most frequently reported adverse events (Supplementary Appendix 11). Gastrointestinal reactions were observed in both groups, with an incidence of 43.3%, followed by rash (12.1%). Other reported adverse events included tremor, palpitations, anorexia, blurred vision, abnormal liver function, dry mouth, hoarseness, and insomnia. The incidence of adverse events did not differ significantly between the CPMs combination with CT and CT alone. Given the complex baseline conditions of patients with chronic diseases, some adverse events may be disease-related rather than directly attributable to the medications. Moreover, the concomitant use of CPMs and conventional drugs may obscure the safety profile of CPMs themselves. Current evidence remains insufficient to confirm the safety of combined therapy, highlighting the necessity for additional rigorously designed studies.

### 3.9 Sensitivity analysis

To evaluate the robustness of our results, a sensitivity analysis was conducted by excluding studies identified as having a high risk of bias, followed by reanalysis of the remaining trials. The results, shown in Supplementary Appendix 12, aligned with the primary analysis, confirming the robustness of our conclusions.

## 4 Discussion

### 4.1 Principal findings

This NMA systematically assessed and compared the efficacy and safety of 10 CPMs for AECOPD, based on 84 RCTs involving 8,477 participants. The assessed outcomes included total effective rate, pulmonary function (FVC, FEV_1_, FEV_1_/FVC), arterial blood gas analysis (PH, PaO_2_, PaCO_2_), inflammatory markers (IL-6, IL-8, TNF-α), and adverse events. The results showed all CPMs significantly improved total effective rate, with ZCL being the most effective. Most CPMs also improved pulmonary function, with SWLDH showing the greatest benefit for FVC and FEV_1_, and QKL for FEV_1_/FVC. In terms of blood gas analysis, QKL and RDN effectively improved blood gas parameters, with QKL most effective for PH, XZL for increasing PaO_2_, and RDN for reducing PaCO_2_. Most CPMs also reduced inflammatory markers, with XZL exerting the greatest effect on IL-6, RDN on IL-8, and QQHT on TNF-α. However, no statistically significant benefit in reducing adverse events was identified for any of the CPMs. On the whole, the findings suggest that CPMs combined with conventional therapy offer benefits in improving clinical efficacy, pulmonary function, blood gas parameters, and inflammatory responses. Nevertheless, the certainty of evidence was assessed as very low to low, additional high-quality RCTs are warranted to confirm these findings.

### 4.2 Role of Chinese patent medicine in patients with AECOPD

In TCM, AECOPD is classified under the categories of lung distension, dyspnea, and cough (31). The condition is predominantly caused by external pathogenic factors, among which phlegm turbidity is considered a key pathological product (32). Excessive phlegm-heat obstructs the lung qi, leading to recurrent cough and dyspnea. The therapeutic principle of clearing heat and resolving phlegm has been shown to alleviate symptoms, improve pulmonary ventilation, and suppress inflammatory mediators and oxidative stress in patients with AECOPD (33). Consistently, the results of our network meta-analysis also confirmed the significant efficacy of CPMs.

The 10 CPMs analyzed in this study mainly exert therapeutic effects by clearing heat and resolving phlegm. Based on their herbal compositions and traditional indications, they can be classified into four categories: (1) wind-dispersing and heat-clearing agents (SFJD, QKL); (2) heat-clearing, phlegm-resolving, and cough-relieving agents (XZL, QQHT, SWLDH, QKPC); (3) heat-clearing and detoxifying agents (TRQ, RDN, XYP); (4) phlegm-resolving and dyspnea-relieving agents (ZCL). Common herbal components among these formulations include *Prunus sibirica* L. (Xingren), *Ephedra wraithiana* I. M. Johnst (Mahuang), *Forsythia suspensa* (Thunb.) Vahl (Lianqiao), *Scutellaria baicalensis* Georgi (Huangqin), and *Fritillaria sichuanica* S. C. Chen (Chuanbeimu). *Prunus sibirica* L and *Ephedra wraithiana* I. M. Johnst relax bronchial smooth muscle and improve airway ventilation; *Forsythia suspensa* (Thunb.) Vahl and *Scutellaria baicalensis* Georgi exhibit anti-inflammatory effects and modulate proinflammatory cytokine expression; *Fritillaria sichuanica* S. C. Chen promotes expectoration. These pharmacological actions may underlie the clinical benefits of CPMs in AECOPD management, although their precise mechanisms are unknown.

### 4.3 Pharmacological mechanisms of critical common herbal compounds and typical CPMs

#### 4.3.1 Pharmacological actions of major herbal components

*Prunus sibirica* L. (Xingren) possesses antitussive, antiasthmatic, inflammation-suppressing, antinociceptive activities and is frequently utilized to alleviate cough and asthma symptoms. Its active constituents include amygdalin, apricot kernel oil, proteins, vitamins, trace elements, and carbohydrates (34, 35). Preclinical studies have shown amygdalin may inhibit the TGF-β1/p-Smad2/3 signaling pathway, blocking epithelial-mesenchymal transition and delaying airway structural damage during acute exacerbations (36). It is frequently included in CPMs for cough relief and asthma control in AECOPD.

*Ephedra wraithiana* I. M. Johnst (Mahuang) exhibits anti-inflammatory, antioxidant, antiviral, diuretic, and antiallergic effects. Its major active compound, ephedrine, has been shown to stimulate dendritic cells to express IL-10 and inhibit TNF-α production mediated by the PI3K/Akt and PGN pathways, contributing to its anti-inflammatory action (37). Ephedrine also directly activates β-receptors on bronchial smooth muscle cells, increases intracellular and plasma cyclic adenosine monophosphate (cAMP) levels, and promotes bronchial smooth muscle relaxation. (38). Moreover, the stems and seeds of *Ephedra wraithiana* I. M. Johnst contain various antibacterial constituents. For example, phenolic compounds isolated from *Ephedra wraithiana* I. M. Johnst show considerable broad-spectrum antimicrobial activity, targeting both gram-positive and gram-negative bacteria and fungi (39). However, the use of *Ephedra wraithiana* I. M. Johnst requires caution due to its alkaloid-associated toxicity, which is commonly incorporated into Commercial Chinese Polyherbal Preparations for relieving cough and dyspnea in AECOPD.

*Forsythia suspensa* (Thunb.) Vahl (Lianqiao) is a frequently employed ingredient in for traditional herbal medicine with anti-inflammatory, antibacterial, and antioxidant properties, frequently applied in the treatment of inflammatory and infectious diseases (40). Forsythoside A, the principal bioactive compound, demonstrates significant anti-inflammatory, antioxidant, and antimicrobial properties. Forsythoside A has been reported in preclinical research to mitigate inflammation by regulating multiple signaling cascades, including NF-κB, MAPK, JAK/STAT, and Nrf2 pathways (41). Furthermore, forsythiaside B has been demonstrated to confer protection against cigarette smoke-induced lung injury via activation of the Nrf2 pathway and inhibition of NF-κB signaling (42).

*Scutellaria baicalensis* Georgi (Huangqin) exhibits anti-inflammatory, antioxidant, antibacterial, antiviral, and immunomodulatory properties, making it a commonly used herb for treating lung-heat syndromes. Its main constituents include flavonoids, volatile oils, and organic acids (43). Studies have illustrated that baicalin, a representative flavonoid, attenuates airway inflammation caused by cigarette smoke exposure in rats by targeting the HDAC2/NF-κB/PAI-1 signaling pathway (44). Therefore, similar to *Forsythia suspensa* (Thunb.) Vahl, *Scutellaria baicalensis* Georgi is frequently incorporated into traditional formulations for AECOPD management targeting inflammation.

*Fritillaria sichuanica* S. C. Chen (Chuanbeimu) possesses antitussive, expectorant, anti-inflammatory, bronchodilatory, and antioxidant properties. Herbal preparations containing *Fritillaria sichuanica* S. C. Chen are widely used for treating chronic cough and sputum production (45). The alkaloids extracted from *Fritillaria sichuanica* S. C. Chen significantly prolong cough latency and reduce cough counts in murine models (46). In COPD rat models, imperialine—one of its key steroidal alkaloids—attenuates pulmonary emphysema progression and airway remodeling by regulating pro-inflammatory cytokines IL-1β, IL-6, IL-8, TNF-α, and restoring MMP-9/TIMP-1 balance (47).

#### 4.3.2 Pharmacological actions of key CPMs

In this study, ZCL showed the most prominent effect in improving total effective rate. Its potential mechanisms may involve upregulating BPIFA1 expression, thereby inhibiting bronchial constriction, inflammation, and epithelial-mesenchymal transition (48). The key constituents of ZCL—β-sitosterol and wogonin have been reported to suppress airway mucus secretion, reduce eosinophil infiltration and IL-4, IL-5, and IL-13 levels, thus preventing airway remodeling (49).

XZL demonstrated the most favorable effects in increasing PaO_2_ levels and reducing IL-6 concentrations. Studies have shown that XZL possesses significant expectorant properties, helping to clear airway secretions, relieve airway obstruction, and improve alveolar ventilation and gas exchange (50). Furthermore, its core herbal component, *Succus Bambusae* (zhuli), exerts antitussive, bronchodilatory, and expectorant effects by modulating inflammatory responses as well as the cAMP, HIF-1α, and VEGF signaling pathways (51).

SWLDH showed the most pronounced efficacy in improving FVC and FEV_1_ levels. Experimental studies have demonstrated SWLDH exerts anti-inflammatory, antipyretic, and antibacterial properties effects, effectively suppressing early inflammatory responses and exerting antipyretic effects in both infectious and non-infectious fever models (52).

QKL demonstrated the greatest efficacy in improving FEV_1_/FVC ratio and blood PH. Animal studies have shown that QKL mitigates pulmonary inflammation by inhibiting the PI3K/AKT and SRC/STAT3 pathways (53), delays lung tissue remodeling, and enhances alveolar ventilation efficiency. Network pharmacology analyses have indicated that QKL active compounds exert anti-inflammatory, immunomodulatory, and antipyretic activities, potentially involving several intracellular signaling mechanisms, notably TNF-α, TRP channel-mediated inflammatory regulation, cAMP, cGMP-PKG, and Th17 cell differentiation (54). Moreover, QKL may reduce airway resistance and improve ventilation by modulating gut microbiota and lung-associated genes expression like Pla2g2a and Pla2g5 (55).

RDN showed the most significant effects in reducing PaCO_2_ and IL-8 levels. Its anti-inflammatory activity involves multiple targets and pathways, including inhibition of pro-inflammatory cytokines, suppression of neutrophil extracellular trap (NET) formation, and blockade of ERK1/2 pathway activation in acute lung injury models (56). Network pharmacology studies suggested that RDN exerted its anti-inflammatory impacts mainly through the PI3K-AKT, cGMP-PKG, and calcium signaling pathways (57).

QQHT exhibited a marked effect in reducing TNF-α levels. Its anti-inflammatory mechanism may involve the inhibition of the JAK2/STAT3 and ERK/p38 MAPK signaling pathways, as well as modulation of the autophagy-related protein p62 pathway, thereby alleviating pulmonary inflammation (58–60).

On the whole, These CPMs appeared to target key pathological processes of AECOPD—mucus hypersecretion, airway inflammation, and ventilatory dysfunction.

The multiple active constituents contained in CPMs may exert synergistic therapeutic effects. However, further systematic and high-quality studies are required to clarify their optimal dosage, safety profiles, and clinical value.

### 4.4 Advantages and challenges of combined Chinese and Western medical approaches for AECOPD

The core advantage of combined Chinese and biomedicine in managing AECOPD lies in its multi-target synergistic effects and toxicity reduction. By combining the local control of biomedicine with the holistic regulation of TCM, this approach aims for both symptom relief and root cause management, potentially enhancing therapeutic efficacy. biomedicine can rapidly suppress inflammation and improve pulmonary ventilation in the short term, while TCM modulates inflammatory cytokine secretion, exerts antioxidant effects, and reduces airway mucus production via various molecular targets and signaling routes, thereby producing a synergistic effect. In AECOPD, airway mucosal infiltration by neutrophils is prominent, with markedly upregulation of inflammatory cytokines such as IL-6, TNF-α, and IL-1β (61). Western therapies, typically corticosteroids and antibiotics, are effective in rapidly controlling inflammation but carry significant risks with prolonged use.

The key active constituents—total flavonoids from *Trichosanthes* L. (Gua lou)—have been shown to significantly reduce IL-1β, IL-6, and IL-13 at both mRNA and protein levels in lung tissues by inhibiting the EGFR/PI3K/AKT and EGFR/STAT3 pathways, thereby alleviating inflammation and mucus hypersecretion (62). Similarly, quercetin effectively attenuates pulmonary inflammation responses and oxidative stress in murine models (63). Other bioactives such as baicalin and curcumin exert anti-inflammatory, antioxidant, anti-apoptotic, and anti-fibrotic roles against COPD through influencing multiple molecular pathways, including JAK3/STAT3/NF-κB, MAPK, Nrf2, and TGF-β1/Smad2/3 (64).

CPMs or integrated Chinese-Western therapies have shown distinct advantages in the management of AECOPD, mainly through their multi-target effects, holistic regulation, and potential for individualized treatment. These approaches may help slow disease progression, reduce the frequency of acute exacerbations, and mitigate adverse effects associated with conventional pharmacotherapy, making them suitable for long-term management of chronic diseases and implementation in primary care. However, several challenges remain, including the lack of high-quality evidence from RCTs, incomplete elucidation of pharmacological mechanisms, variability in formulation standardization and quality control, insufficie research on herb-drug interactions, and underdeveloped reimbursement policies and clinical implementation strategies. Therefore, further systematic and scientifically rigorous studies are urgently needed. The application of bioinformatics, multi-omics technologies, and artificial intelligence may facilitate the standardization and scientific validation of CPMs in AECOPD treatment.

### 4.5 Advantages and limitations

This study employed NMA to comprehensively assess the efficacy and safety of 10 CPMs in combination with CT for AECOPD. The findings offered valuable insights for optimizing integrative treatment strategies but were subject to several limitations.

First, all eligible trials were written and published in Chinese, excluding international trials. Such inclusion criteria could result in language and geographic bias, which in turn may compromise the generalizability of the results. Additionally, the primary endpoint, total effective rate, is a composite measure that may exhibit heterogeneity across studies, is less directly comparable to patient-centered or hard outcomes, and its applicability is limited to Chinese AECOPD trials. Additionally, a small number of RCTs for certain CPMs may contribute to publication bias.

Second, in assessing transitivity and clinical heterogeneity, although age and disease duration were assessed for transitivity, key effect modifiers—such as AECOPD severity, GOLD stage, pneumonia status, and baseline lung function—were poorly reported in most trials. This limits the validity of indirect comparisons. Correspondingly, PH showed significant inconsistency, and FEV_1_/FVC, PaO_2_, and PaCO_2_ exhibited high heterogeneity, likely due to unmeasured variations in disease severity, CPM dosage, or treatment duration. On the other hand, CT regimens were largely similar across trials—including oxygen therapy, antimicrobial agents, bronchodilators, antitussives, and expectorants, the effects are expected to be broadly similar. However, minor differences in drug choice, dosing, or delivery may still contribute to heterogeneity. Such variability should be considered when interpreting the relative effects of CPM + CT versus CT alone, and may partially impact the transitivity assumption and clinical heterogeneity in the network meta-analysis. In addition, the absence of dedicated studies on individual CPMs prevented closed-loop indirect comparisons, potentially elevating the risk of bias in the analysis and limiting the rigorous testing of the consistency assumption.

Third, given the low quality of the existing evidence and the generally inadequate methodological reporting in the included RCTs, clinicians should exercise caution when applying these findings. Although SUCRA-based rankings were presented, these results should be interpreted as exploratory rather than definitive evidence, since most comparisons were rated as low or very low certainty by CINeMA. Therefore, these results should not be over-interpreted and are intended only to provide a general overview, rather than conclusive guidance on the relative superiority of the CPMs.

Additionally, the absence of long-term and patient-important outcomes represents a major limitation, and the current findings cannot inform long-term prognosis or outcomes most relevant to patients. Regarding safety outcomes, although the NMA showed no statistically significant differences in adverse events between CPM + CT and CT alone, the certainty of evidence was very low according to CINeMA. Therefore, these results should not be interpreted as evidence of equivalence in safety. Clinicians should be cautious when interpreting the safety findings, as the current evidence is insufficient to draw definitive conclusions.

Therefore, to further confirm the clinical benefits of CPMs in AECOPD treatment, rigorously controlled, large, multicenter RCTs should be prioritized in future research. This will contribute to more dependable evidence for evidence-based practice and promote the standardized integration of traditional Chinese and Western medicinal strategies.

## 5 Conclusion

In summary, ZCL showed the greatest effect in improving total effective rate. SWLDH and QKL demonstrated the most superior benefits in enhancing ventilatory function in patients with AECOPD. For arterial blood gas improvement, QKL, XZL, and RDN appeared to be the most preferable, while QQHT, XZL, and RDN were the most effective in reducing inflammatory markers. Selecting CPMs based on specific clinical needs may help achieve personalized treatment goals. However, these results are limited by the small sample sizes and variable quality of existing studies. Therefore, large-scale, multi-center RCTs are warranted to validate the clinical value of CPM combinations, providing more robust evidence on their efficacy and safety, supporting individualized treatment strategies and optimizing clinical decision-making in AECOPD management.

## Data Availability

The datasets presented in this study can be found in online repositories. The names of the repository/repositories and accession number(s) can be found at: PubMed, Cochrane Library, Embase, Web of Science, CNKI, VIP, Wanfang, and CBM.

## Footnotes

1
www.worldfloraonline.org

2
http://mpns.kew.org
