# Supplementary material for: Comparative effectiveness of ten CPMs for acute exacerbation of chronic obstructive pulmonary disease: systematic review and network meta-analysis
Source: Front Med (Lausanne). 2026 Jan 15;12:1719361. doi: 10.3389/fmed.2025.1719361 (PMC12852438; doi:10.3389/fmed.2025.1719361)
Supplement: Supplementary file 1 [file Table_1.docx]

**
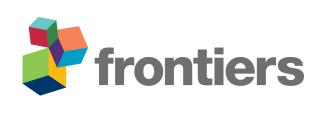
**

***Supplementary Material***

Comparative effectiveness of ten CPMs for acute exacerbation of chronic obstructive pulmonary disease: systematic review and network meta-analysis

Table of contents

*Appendix 1: PRISMA-NMA checklist.................................................................................................................1*

*Appendix 2: Search strategy.............................................................................................................................. 5*

*Appendix 3: Characteristics of included studies ...............................................................................................6*

*Appendix 4: Risk of bias of randomized clinical trials....................................................................................30*

*Appendix 5: Evaluation of inconsistency and heterogeneity ...........................................................................33*

*Appendix6:CINeMA Assessment .....................................................................................................................34*

*Appendix 7: Funnel plot of each outcome........................................................................................................66*

*Appendix 8: SUCRA and cumulative probability plots....................................................................................72*

*Appendix9:Network meta-analysis of various CPMs on secondary endpoints of AECOPD.........................83*

*Appendix10:League table of Summary Estimates for CPMs on AECOPD of 84 Trials................................90*

*Appendix 11: Specific Examples of Adverse Events.......................................................................................100*

*Appendix 12: Sensitivity analyses..................................................................................................................103*

**Appendix 1: PRISMA NMA Checklist**

| **Section/Topic** | **Item #** | **Checklist Item** | **Reported on Page #** |
| --- | --- | --- | --- |
| **TITLE** |  |  |  |
| Title | 1 | Identify the report as a systematic review *incorporating a network meta-analysis (or related form of meta-analysis).* | 1 |
|  |  |  |  |
| **ABSTRACT** |  |  |  |
| Structured summary | 2 | Provide a structured summary including, as applicable:  **Background:** main objectives  **Methods:** data sources; study eligibility criteria, participants, and interventions; study appraisal; and *synthesis methods, such as network meta-analysis.*  **Results:** number of studies and participants identified; summary estimates with corresponding confidence/credible intervals; *treatment rankings may also be discussed. Authors may choose to summarize pairwise comparisons against a chosen treatment included in their analyses for brevity.*  **Discussion/Conclusions:** limitations; conclusions and implications of findings.  **Other:** primary source of funding; systematic review registration number with registry name. | 1-2 |
|  |  |  |  |
| **INTRODUCTION** |  |  |  |
| Rationale | 3 | Describe the rationale for the review in the context of what is already known*, including mention of why a network meta-analysis has been conducted.* | ***3*** |
| Objectives | 4 | Provide an explicit statement of questions being addressed, with reference to participants, interventions, comparisons, outcomes, and study design (PICOS). | 4 |
|  |  |  |  |
| **METHODS** |  |  |  |
| Protocol and registration | 5 | Indicate whether a review protocol exists and if and where it can be accessed (e.g., Web address); and, if available, provide registration information, including registration number. | 5 |
| Eligibility criteria | 6 | Specify study characteristics (e.g., PICOS, length of follow-up) and report characteristics (e.g., years considered, language, publication status) used as criteria for eligibility, giving rationale. *Clearly describe eligible treatments included in the treatment network, and note whether any have been clustered or merged into the same node (with justification).* | ***5*** |
| Information sources | 7 | Describe all information sources (e.g., databases with dates of coverage, contact with study authors to identify additional studies) in the search and date last searched. | 5 |
| Search | 8 | Present full electronic search strategy for at least one database, including any limits used, such that it could be repeated. | 5-6 |
| Study selection | 9 | State the process for selecting studies (i.e., screening, eligibility, included in systematic review, and, if applicable, included in the meta-analysis). | 6 |
| Data collection process | 10 | Describe method of data extraction from reports (e.g., piloted forms, independently, in duplicate) and any processes for obtaining and confirming data from investigators. | 6 |
| Data items | 11 | List and define all variables for which data were sought (e.g., PICOS, funding sources) and any assumptions and simplifications made. | 6 |
| **Geometry of the network** | **S1** | Describe methods used to explore the geometry of the treatment network under study and potential biases related to it. This should include how the evidence base has been graphically summarized for presentation, and what characteristics were compiled and used to describe the evidence base to readers. | ***7*** |
| Risk of bias within individual studies | 12 | Describe methods used for assessing risk of bias of individual studies (including specification of whether this was done at the study or outcome level), and how this information is to be used in any data synthesis. | 6 |
| Summary measures | 13 | State the principal summary measures (e.g., risk ratio, difference in means). *Also describe the use of additional summary measures assessed, such as treatment rankings and surface under the cumulative ranking curve (SUCRA) values, as well as modified approaches used to present summary findings from meta-analyses.* | 6 |
| Planned methods of analysis | 14 | Describe the methods of handling data and combining results of studies for each network meta-analysis. This should include, but not be limited to:   - *Handling of multi-arm trials;* - *Selection of variance structure;* - *Selection of prior distributions in Bayesian analyses; and* - *Assessment of model fit.* | 7 |
| **Assessment of Inconsistency** | **S2** | Describe the statistical methods used to evaluate the agreement of direct and indirect evidence in the treatment network(s) studied. Describe efforts taken to address its presence when found. | 7 |
| Risk of bias across studies | 15 | Specify any assessment of risk of bias that may affect the cumulative evidence (e.g., publication bias, selective reporting within studies). | **7** |
| Additional analyses | 16 | Describe methods of additional analyses if done, indicating which were pre-specified. This may include, but not be limited to, the following:   - Sensitivity or subgroup analyses; - Meta-regression analyses; - *Alternative formulations of the treatment network; and* - *Use of alternative prior distributions for Bayesian analyses (if applicable).* | ***7*** |
| **RESULTS†** |  |  |  |
| Study selection | 17 | Give numbers of studies screened, assessed for eligibility, and included in the review, with reasons for exclusions at each stage, ideally with a flow diagram. | 8 |
| **Presentation of network structure** | **S3** | Provide a network graph of the included studies to enable visualization of the geometry of the treatment network. | ***8*** |
| **Summary of network geometry** | **S4** | Provide a brief overview of characteristics of the treatment network. This may include commentary on the abundance of trials and randomized patients for the different interventions and pairwise comparisons in the network, gaps of evidence in the treatment network, and potential biases reflected by the network structure. | ***8*** |
| Study characteristics | 18 | For each study, present characteristics for which data were extracted (e.g., study size, PICOS, follow-up period) and provide the citations. | 8 |
| Risk of bias within studies | 19 | Present data on risk of bias of each study and, if available, any outcome level assessment. | 9 |
| Results of individual studies | 20 | For all outcomes considered (benefits or harms), present, for each study: 1) simple summary data for each intervention group, and 2) effect estimates and confidence intervals. *Modified approaches may be needed to deal with information from larger networks.* | ***Appendix3*** |
| Synthesis of results | 21 | Present results of each meta-analysis done, including confidence/credible intervals. *In larger networks, authors may focus on comparisons versus a particular comparator (e.g. placebo or standard care), with full findings presented in an appendix. League tables and forest plots may be considered to summarize pairwise comparisons.* If additional summary measures were explored (such as treatment rankings), these should also be presented. | 10 |
| **Exploration for inconsistency** | **S5** | Describe results from investigations of inconsistency. This may include such information as measures of model fit to compare consistency and inconsistency models, *P* values from statistical tests, or summary of inconsistency estimates from different parts of the treatment network. | ***Appendix5*** |
| Risk of bias across studies | 22 | Present results of any assessment of risk of bias across studies for the evidence base being studied. | 9 |
| Results of additional analyses | 23 | Give results of additional analyses, if done (e.g., sensitivity or subgroup analyses, meta-regression analyses*, alternative network geometries studied, alternative choice of prior distributions for Bayesian analyses,* and so forth). | 17 |
| **DISCUSSION** |  |  |  |
| Summary of evidence | 24 | Summarize the main findings, including the strength of evidence for each main outcome; consider their relevance to key groups (e.g., healthcare providers, users, and policy-makers). | 17 |
| Limitations | 25 | Discuss limitations at study and outcome level (e.g., risk of bias), and at review level (e.g., incomplete retrieval of identified research, reporting bias). *Comment on the validity of the assumptions, such as transitivity and consistency. Comment on any concerns regarding network geometry (e.g., avoidance of certain comparisons).* | 23 |
| Conclusions | 26 | Provide a general interpretation of the results in the context of other evidence, and implications for future research. | 24 |
| **FUNDING** |  |  |  |
| Funding | 27 | Describe sources of funding for the systematic review and other support (e.g., supply of data); role of funders for the systematic review. This should also include information regarding whether funding has been received from manufacturers of treatments in the network and/or whether some of the authors are content experts with professional conflicts of interest that could affect use of treatments in the network. | ***24*** |

**Appendix 2: Search strategy**

Table S1. Search strategy of PubMed

| # | Searches |
| --- | --- |
| 1 | (Acute exacerbation of chronic obstructive pulmonary disease[Title/Abstract]) |
| 2 | ("Medicine, Chinese Traditional"[MeSH] OR "Drugs, Chinese Herbal"[MeSH] OR "Medicine, East Asian Traditional"[MeSH] OR "Chinese Patent Medicine"[Title/Abstract] OR "Chinese Herbal Medicine"[Title/Abstract] OR "Chinese Traditional Medicine"[Title/Abstract] OR "TCM"[Title/Abstract] OR "Traditional Chinese Medicine"[Title/Abstract] OR "Chinese Proprietary Medicine"[Title/Abstract] OR "Chinese Herbal Drugs"[Title/Abstract] OR "Chinese Herbal Formulations"[Title/Abstract]) |
| 3 | ("Randomized Controlled Trials as Topic"[MeSH] OR "Randomized Controlled Trial"[Title/Abstract] OR "Randomized Clinical Trial"[Title/Abstract] OR "RCT"[Title/Abstract] OR "Randomized Trial"[Title/Abstract] OR "Randomised Controlled Trial"[Title/Abstract] OR "Randomised Clinical Trial"[Title/Abstract]) |
| 4 | #1 AND #2 AND #3 |

Table S2. Search strategy of Cochrane Central Register of Controlled Trials

| # | Searches |
| --- | --- |
| 1 | ("Acute exacerbation of chronic obstructive pulmonary disease") |
| 2 | ("Medicine, Chinese Traditional" OR "Drugs, Chinese Herbal" OR "Medicine, East Asian Traditional" OR "Chinese Patent Medicine" OR "Chinese Herbal Medicine" OR "Chinese Traditional Medicine" OR "TCM" OR "Traditional Chinese Medicine" OR "Chinese Proprietary Medicine" OR "Chinese Herbal Drugs" OR "Chinese Herbal Formulations") |
| 3 | ("Randomized Controlled Trials" OR "Randomized Controlled Trial" OR "Randomized Clinical Trial" OR "RCT" OR "Randomized Trial" OR "Randomised Controlled Trial" OR "Randomised Clinical Trial") |
| 4 | #1 AND #2 AND #3 |

Table S3. Search strategy of Embase

| # | Searches |
| --- | --- |
| 1 | ('Acute exacerbation of chronic obstructive pulmonary disease':ab,ti) |
| 2 | ('Medicine, chinese traditional':ab,ti OR 'drugs, chinese herbal':ab,ti OR 'medicine, east asian traditional':ab,ti OR 'chinese patent medicine':ab,ti OR 'chinese herbal medicine':ab,ti OR 'chinese traditional medicine':ab,ti OR 'TCM':ab,ti OR 'traditional chinese medicine':ab,ti OR 'chinese proprietary medicine':ab,ti OR 'chinese herbal drugs':ab,ti OR 'chinese herbal formulations':ab,ti) |
| 3 | ('Randomized Controlled Trials':ab,ti OR 'Randomized Controlled Trial':ab,ti OR 'Randomized Clinical Trial':ab,ti OR 'RCT':ab,ti OR 'Randomized Trial':ab,ti OR 'Randomised Controlled Trial':ab,ti OR 'Randomised Clinical Trial':ab,ti) |
| 4 | #1 AND #2 AND #3 |

Table S4. Search strategy of Web of Science

| # | Searches |
| --- | --- |
| 1 | TS=(Acute exacerbation of chronic obstructive pulmonary disease) |
| 2 | ((((((((((TS=(Medicine, chinese traditional)) OR TS=(drugs, chinese herbal)) OR TS=(medicine, east asian traditional)) OR TS=(chinese patent medicine)) OR TS=(chinese herbal medicine)) OR TS=(chinese traditional medicine)) OR TS=(TCM)) OR TS=(traditional chinese medicine)) OR TS=(chinese proprietary medicine)) OR TS=(chinese herbal drugs)) OR TS=(chinese herbal formulations) |
| 3 | ((((((TS=(Randomized Controlled Trials)) OR TS=(Randomized Controlled Trial)) OR TS=(Randomized Clinical Trial)) OR TS=(RCT)) OR TS=(Randomized Trial)) OR TS=(Randomised Controlled Trial)) OR TS=(Randomised Clinical Trial) |
| 4 | #1 AND #2 AND #3 |

Table S5. Search strategy of CNKI, VIP, WanFang and CBM

| # | Searches |
| --- | --- |
| 1 | (急性加重慢性阻塞性肺疾病 OR 慢性阻塞性肺病急性加重 OR 慢性阻塞性肺病急性发作 OR 急性加重期慢性阻塞性肺疾病 OR 慢性阻塞性肺疾病加重期 OR 慢阻肺急性加重期 OR 慢阻肺急性发作 OR 慢性阻塞性肺疾病急性发作 OR 慢性阻塞性肺疾病急性加重 OR 慢性阻塞性肺病急性发作期 OR 慢性阻塞性肺部疾病急性发作 OR 慢性阻塞性肺疾病急性发作期 OR 慢性阻塞性肺疾病急性加重期) |
| 2 | (中医药 OR 中草药 OR 中药 OR 中医 OR 传统中医 OR 中成药 OR 中医药方 OR 中药制剂) |
| 3 | (疏风解毒胶囊 OR 复方鲜竹沥液 OR 鲜竹沥口服液 OR 清气化痰丸 OR 清咳平喘颗粒 OR 痰热清注射液 OR  热毒宁注射液 OR 十味龙胆花胶囊 OR 止喘灵注射液 OR 清开灵注射液 OR 喜炎平注射液) |
| 4 | #2 OR #3 |
| 5 | (随机对照试验 OR 随机临床试验 OR RCT OR 随机试验 OR 随机对照研究) |
| 4 | #1 AND #4 AND #5 |

**Appendix 3: Characteristics of included studies**

Table S3.1: Baseline of characteristics of included studies

| study ID | Sample size  (T/C) | Age  (mean±SD),Years | Disease duration (mean ± SD),Years | Follow-up  Duration,Days | Treatment Group | Control Group | outcomes |
| --- | --- | --- | --- | --- | --- | --- | --- |
| Yang2021^[1]^ | 49/49 | 66.61±11.03/66.85±11.18 | 9.38±2.68/9.49±2.71 | 7 | SFJD | CT | 123467⑪ |
| He2020^[2]^ | 106/106 | 57.37±6.35/57.49±6.47 | 5.61±1.82/5.69±1.88 | 14 | SFJD | CT | 1234⑨⑩⑪ |
| Chen2021^[3]^ | 30/30 | 65.79±8.40/63.21±7.15 | - | 10 | SFJD | CT | 8⑪ |
| Jiang2022^[4]^ | 64/63 | 62.56±2.61/62.61±2.57 | 6.72±2.61/6.75±2.05 | - | SFJD | CT | 134⑪ |
| Tian2021^[5]^ | 50/50 | 67.82±8.41/69.55±6.74 | 11.75±7.85/10.28±8.53 | 8 | SFJD | CT | 145⑪ |
| Yin2022^[6]^ | 50/50 | 63.02±2.80/63.10±4.04 | 5.32±0.71/5.22±1.07 | 30 | SFJD | CT | 14 |
| Tang2022^[7]^ | 54/55 | 57.33±4.81/56.74±5.14 | 8.91±4.79/8.74±1.42 | 14 | SFJD | CT | 123456789 |
| Wang2016^[8]^ | 41/39 | 63.53±9.34/62.53±8.53 | - | - | SFJD | CT | 167 |
| Zhang2015^[9]^ | 65/65 | 67±5.9/66±7.7 | 4.1±2.2/4.3±1.5 | 10 | SFJD | CT | 17⑨⑩⑪ |
| Li2025^[10]^ | 30/30 | 69.60±6.48/69.66±6.27 | - | 7 | SFJD | CT | 1234 |
| Yu2024^[11]^ | 45/45 | 65.72±10.18/64.50±9.78 | 4.66±1.29/5.23±1.40 | 7 | SFJD | CT | 123457 |
| Chen2020^[12]^ | 33/33 | 61.7±4.26/62.3±5.21 | - | 14 | SFJD | CT | 14⑨⑩ |
| Zhang2019^[13]^ | 30/30 | 61.48±3.15/62.32±3.21 | 19.45±2.26/20.21±2.15 | 14 | SFJD | CT | 134 |
| Wang2015^[14]^ | 60/60 | 54.0±6.5/55.0±7.3 | 9.4±4.3/8.7±6.3 | 7 | SFJD | CT | 1⑨⑩⑪ |
| Wang2020^[15]^ | 41/41 | 62.9±8.0/62.1±7.5 | 11.4±4.3/11.9±4.6 | 14 | XZL | CT | 1467⑪ |
| He2021^[16]^ | 80/80 | 58.93±8.94/59.43±9.21 | 10.74±2.19/11.15±2.54 | 14 | XZL | CT | 145⑨⑩ |
| Luo2020^[17]^ | 50/50 | 58.88±5.58/58.80±5.68 | 5.85±1.12/5.93±1.18 | 9 | XZL | CT | 167⑨⑩⑪ |
| Wang2017^[18]^ | 161/160 | 63.98±9.74/65.00±9.91 | 13.16±8.14/13.36±8.45 | 7 | XZL | CT | 13457⑧⑨⑩⑪ |
| Pei2019^[19]^ | 63/63 | 65.87±7.39/65.83±7.21 | 15.27±4.11/15.21±4.06 | 14 | XZL | CT | 1234 |
| Zheng2016^[20]^ | 59/59 | 62.12±4.17/61.33±4.28 | 8.53±2.11/8.47±2.08 | 7 | XZL | CT | 1234⑨⑩ |
| Li2024^[21]^ | 40/40 | 59.29±5.41/59.35±5.45 | - | 14 | XZL | CT | 123457⑨⑩⑪ |
| Wang2021^[22]^ | 200/200 | 62.96±7.78/62.04±7.43 | - | 14 | QQHT | CT | 124⑪ |
| Hou2019^[23]^ | 40/40 | 53.24±5.16/55.83±5.11 | 7.64±2.25/8.47±2.51 | 14 | QQHT | CT | 13456⑨⑩⑪ |
| Jiang2019^[24]^ | 31/30 | 57.60±12.35/58.55±12.38 | 8.32±3.75/8.17±3.84 | 10 | QQHT | CT | 167 |
| Jiang2021^[25]^ | 32/32 | 57.60±12.35/58.55±12.38 | 8.32±3.75/8.17±3.84 | 10 | QQHT | CT | 1⑨⑩⑪ |
| Liu2019^[26]^ | 30/30 | 53.14±5.15/55.75±5.12 | 7.54±2.15/8.54±3.12 | 14 | QQHT | CT | 13467⑨⑩⑪ |
| Wei2020^[27]^ | 45/45 | 63.14±8.15/65.75±5.12 | 7.84±2.65/8.04±3.16 | 10 | QQHT | CT | 145⑨⑩ |
| Ji2017^[28]^ | 50/50 | 53.10±5.18/54.08±5.29 | 7.61±2.60/7.85±2.63 | 14 | QQHT | CT | 146⑨⑩ |
| Qu2023^[29]^ | 43/43 | 72.21±6.32/71.35±6.17 | 11.72±3.81/11.05±3.28 | 14 | QQHT | CT | 14⑨⑩⑮ |
| Wu2014^[30]^ | 40/40 | 63.0±12.5/61.7±11.8 | 7.5±3.2/7.2±3.0 | 7 | QKPC | CT | 123⑧⑨⑩ |
| Cai2023^[31]^ | 40/40 | 65.80±5.31/63.68±8.70 | 10.95±1.91/13.08±3.13 | 7 | QKPC | CT | 1⑧⑨⑩⑪ |
| Yu2024^[32]^ | 48/47 | 64.8±10.2/66.4±9.5 | 30.7±3.6/35.3±6.1 | 14 | QKPC | CT | 15⑨⑩⑪ |
| Liu2024^[33]^ | 50/50 | 71.20±8.83/74.93±8.18 | 15.30±7.14/13.45±8.24 | 14 | QKPC | CT | 14⑪ |
| Li2009^[34]^ | 48/48 | 60±5.3/58±6.2 | - | 14 | TRQ | CT | 13⑧⑨⑩⑪ |
| Liu2012^[35]^ | 31/31 | 61.2±11.5/59.5±11.4 | - | 14 | TRQ | CT | 134 |
| Yu2019^[36]^ | 61/60 | 65.19±10.35/63.47±8.77 | 10.36±3.10/11.17±3.65 | 14 | TRQ | CT | 1234⑧⑨⑩ |
| Xiang2022^[37]^ | 50/50 | 70.90±4.70/71.65±4.72 | 12.37±1.99/12.10±1.98 | 10 | TRQ | CT | 23457⑧⑨⑩⑪ |
| Zhang2024^[38]^ | 38/38 | 68.13±10.25/68.26±7.90 | 11.74±4.83/11.89±4.38 | 10 | TRQ | CT | 12345⑪ |
| Zhang2006^[39]^ | 37/37 | 63.25±10.15/62.50±11.30 | 18.12±6.02/17.55±5.40 | 7 | TRQ | CT | 1⑨⑩⑪ |
| Hong2008^[40]^ | 22/21 | 65±7.2/66±6.7 | - | 10 | TRQ | CT | 1⑪ |
| Zhang2010^[41]^ | 36/20 | 61.5±11.5/59.4±11.2 | - | 10 | TRQ | CT | 17⑪ |
| Yao2020^[42]^ | 75/75 | 55.07±7.78/54.19±7.82 | 10.05±3.61/9.42±3.47 | 14 | TRQ | CT | 349⑩ |
| Rao2012^[43]^ | 50/50 | 62.1±6.8/62.4±7.3 | - | 7 | RDN | CT | 14⑪ |
| Sun2012^[44]^ | 51/51 | - | - | 10 | RDN | CT | 134567⑧⑨⑩⑪ |
| Ma2020^[45]^ | 43/43 | 57.11±5.36/59.35±4.42 | 10.13±4.02/10.51±4.11 | 10 | RDN | CT | 134⑨⑩⑪ |
| Yu2022^[46]^ | 35/35 | 64.80±6.49/64.91±5.97 | 6.98±1.75/6.73±1.82 | 14 | RDN | CT | 123⑨⑩ |
| Shao2023^[47]^ | 45/45 | 65.5±4.3/66.7±4.1 | - | 10 | RDN | CT | 123⑪ |
| Tang2021^[48]^ | 45/45 | 63±6/63±7 | 6.5±1.9/7.1±1.8 | 14 | RDN | CT | 12345 |
| Pang2015^[49]^ | 55/55 | 55.00±4.25/54.00±3.65 | - | 14 | RDN | CT | 1⑨⑩ |
| Zeng2014^[50]^ | 114/110 | - | - | 10 | RDN | CT | 4 |
| Chen2014^[51]^ | 77/77 | 64.7±12.2/65.8±11.8 | 22.1±12.5/22.9±10.6 | 7 | RDN | CT | 123⑧⑨⑩⑪ |
| Lu2018^[52]^ | 44/44 | 65.9±6.78/67.1±4.59 | - | 7 | RDN | CT | 1⑪ |
| Wang2013^[53]^ | 48/48 | 58.9±9.4/59.5±9.8 | - | 7 | RDN | CT | 1 |
| Wei2014^[54]^ | 37/33 | - | - | 10 | RDN | CT | 134 |
| Zhou2014^[55]^ | 30/30 | 72.6±5.4/73.1±5.8 | - | 7 | RDN | CT | 1⑧⑨⑩⑪ |
| Peng2021^[56]^ | 36/36 | 65.83±8.23/63.92±10.32 | 12.44±4.74/12.31±5.14 | 10 | RDN | CT | 1567 |
| Sun2022^[57]^ | 65/65 | 67.90±8.60/68.62±8.59 | - | 28 | SWLDH | CT | 4 |
| Fu2021^[58]^ | 60/60 | 70.63±5.46/70.32±5.38 | 12.21±1.89/12.30±1.92 | 14 | SWLDH | CT | 1458 |
| Liu2020^[59]^ | 52/51 | 41.96±5.47/42.58±6.27 | 3.28±0.84/3.16±0.73 | 14 | SWLDH | CT | 12345⑪ |
| Peng2019^[60]^ | 58/58 | 62.27±9.63/58.55±10.28 | 8.32±5.16/7.95±4.86 | 15 | SWLDH | CT | 147⑨⑩ |
| Zhang2015^[61]^ | 82/81 | 58.15±11.23/57.68±11.32 | 16.34±9.26/15.57±8.85 | 10 | SWLDH | CT | 134⑪ |
| Lei2019^[62]^ | 36/36 | 55.8±10.5/56.3±10.2 | - | 10 | SWLDH | CT | 5 |
| Re2022^[63]^ | 39/39 | 63.2±4.6/62.5±4.8 | 10.8±5.3/10.4±5.5 | 14 | ZCL | CT | 14⑪ |
| Zhang2017^[64]^ | 30/30 | 64.51±11.31/65.04±12.42 | 7.43±2.55/7.50±2.62 | 7 | ZCL | CT | 1 |
| Zhang2025^[65]^ | 30/30 | 65.87±4.26/65.79±4.30 | 5.56±2.21/5.54±2.17 | 7 | ZCL | CT | 1234⑪ |
| Zhao2024^[66]^ | 100/100 | 49.91±6.27/49.26±5.73 | 3.58±0.62/3.63±0.57 | 14 | ZCL | CT | 12345⑪ |
| Zeng2009^[67]^ | 23/23 | 70.5±8.6/69.8±9.5 | - | 15 | QKL | CT | 67 |
| Sun2001^[68]^ | 34/30 | - | - | 7 | QKL | CT | 167 |
| Ding2013^[69]^ | 60/60 | 62.3±6.8/62.4±7.4 | - | 10 | QKL | CT | 46⑪ |
| Li2016^[70]^ | 46/46 | 66.53±3.42/66.54±3.45 | 6.65±0.58/6.71±0.62 | 14 | QKL | CT | 123467⑧⑨⑩⑪ |
| Cao2012^[71]^ | 60/60 | 65.28±11.45/66.43±10.28 | - | 7 | QKL | CT | 1234⑨⑩⑪ |
| Chang2006^[72]^ | 29/29 | - | - | 14 | QKL | CT | 123⑪ |
| Yang2009^[73]^ | 45/45 | 71.5±9.3/70.4±8.9 | - | 14 | QKL | CT | 1234⑨⑩ |
| He2008^[74]^ | 45/45 | - | - | 7 | QKL | CT | 1⑪ |
| Xie2022^[75]^ | 40/40 | 83.58±7.01/85.75±10.53 | 13.53±4.70/12.15±4.51 | 7 | XYP | CT | 2345 |
| Zeng2018^[76]^ | 59/59 | 67.7±11.6/69.3±11.4 | - | 14 | XYP | CT | 1 |
| Liang2012^[77]^ | 15/15 | - | - | 14 | XYP | CT | 3⑨⑩ |
| Chang2015^[78]^ | 44/44 | - | - | 10 | XYP | CT | 1 |
| Zhang2014^[79]^ | 46/46 | 67.84±7.4/67.6±7.2 | 4.8±3.4/4.6±3.2 | 14 | XYP | CT | 1234⑪ |
| Han2012^[80]^ | 23/27 | - | - | 14 | XYP | CT | 1 |
| Zhang2011^[81]^ | 40/40 | 68.3±5.5/66.9±7.4 | - | 10 | XYP | CT | 1⑪ |
| Liu2016^[82]^ | 30/30 | - | - | - | XYP | CT | 14⑨⑩ |
| Zhang2017^[83]^ | 45/45 | 45.89±4.69/46.21±4.59 | - | 10 | XYP | CT | 1234 |
| Han2015^[84]^ | 62/60 | - | - | 14 | XYP | CT | 3⑨⑩ |

NOTE: T, treatment group; C, control group; CT, Conventional biomedicine treatment;①Total effective rate ; ②FEV1; ③FVC; ④FEV1/FVC(%); ⑤IL-6;⑥IL-8;⑦TNF-α;⑧PH⑨PaO2;⑩PaCO2;⑪Adverse event.Shufeng Jiedu Capsule (SFJD), Fresh Bamboo Juice Oral Liquid (XZL), Qingqi Huatan Pill (QQHT),Qingke Pingchuan Granule(QKPC), Tanreqing Injection (TRQ), Reduning Injection (RDN), Shiwei Longdanhua Capsule(SWLDH), Zhichuanling Injection (ZCL),Qingkailing Injection (QKL),Xiyanping Injection (XYP).

**References**

1. Yang Q, Wang J, Zhi J, Chen G. Clinical efficacy and safety of Shufeng Jiedu Capsule combined with dual bronchodilators in the treatment of acute exacerbation of chronic obstructive pulmonary disease (COPD) of phlegm-heat obstructing lung type in the elderly. Chin J Tradit Chin Med Pharm. 2021;36(03):1761–4. doi:10.88888/j.1673-1727.2021.3.1761-1764.
2. He W, Yuan Y, Ao S. Clinical efficacy of Shufeng Jiedu Capsule combined with salmeterol/fluticasone in acute exacerbation of COPD and its effect on SAA, IL-1β and PCT levels. Chin Arch Tradit Chin Med. 2020;38(04):252–5. doi:10.13193/j.issn.1673-7717.2020.04.060.
3. Chen W, Zhang N. Effect of Shufeng Jiedu Capsule on sTREM-1, YKL-40, SP-D, and Fib in acute exacerbation of COPD. Chin J Tradit Chin Med Pharm. 2021;36(02):1171–4. doi:10.88888/j.1673-1727.2021.2.1171-1174.
4. Jiang X, Wang D, Yi Z. Clinical observation of Shufeng Jiedu Capsule combined with tiotropium bromide in the treatment of acute exacerbation of COPD. Chin J Emerg Tradit Chin Med. 2022;31(06):1045–8. doi:10.3969/j.issn.1004-745X.2022.06.030.
5. Tian T, Tang C, Qu X. Clinical observation of Shufeng Jiedu Capsule combined with conventional therapy in the treatment of acute exacerbation of COPD (phlegm-heat obstructing lung syndrome). Chin J Emerg Tradit Chin Med. 2021;30(01):124–7. doi:10.3969/j.issn.1004-745X.2021.01.035.
6. Yin J, Lin Y, Lu C, Li N, Zhou Q, Yu Q, et al. Study on the value of Shufeng Jiedu Capsule as an adjuvant treatment in acute exacerbation of COPD. Liaoning J Tradit Chin Med. 2022;49(11):120–3. doi:10.13192/j.issn.1000-1719.2022.11.031.
7. Tang F, Liang Z, Han X, Tang Y, Lv G. Effect of Shufeng Jiedu Capsule on inflammatory response, airway remodeling, and hemorheology in acute exacerbation of COPD. Chin J Emerg Tradit Chin Med. 2022;31(07):1181–4. doi:10.3969/j.issn.1004-745X.2022.07.013.
8. Wang F, Lin S, Wu D, He H, Shi H. Effect of Shufeng Jiedu Capsule on IL-8 and TNF-α in patients with acute exacerbation of COPD. Chin J Emerg Tradit Chin Med. 2016;25(11):2171–3. doi:10.3969/j.issn.1004-745X.2016.11.052.
9. Zhang L, Li Y. Effect of Shufeng Jiedu Capsule on efficacy and nutritional indicators in acute exacerbation of COPD. Beijing Med J. 2015;37(10):974–6. doi:10.15932/j.0253-9713.2015.10.021.
10. Li Y, Xia Y, Liang J, Zhang N. Clinical observation of Shufeng Jiedu Capsule in the treatment of acute exacerbation of COPD (phlegm-heat obstructing lung syndrome). Chin J Emerg Tradit Chin Med. 2020;29(11):2025–7. doi:10.3969/j.issn.1004-745X.2020.11.041.
11. Yu H, Chen D. Clinical study on Shufeng Jiedu Capsule combined with triple nebulized inhalation therapy for acute exacerbation of chronic obstructive pulmonary disease. J New Chin Med. 2024;56(22):58–62. doi:10.13457/j.cnki.jncm.2024.22.012.
12. Chen L, Gan H, Zhou W, Jin L, Rao J. Clinical observation of Shufeng Jiedu Capsule combined with biomedicine in the treatment of acute exacerbation of COPD of phlegm-heat obstructing lung type. Beijing J Tradit Chin Med. 2020;39(01):59–62. doi:10.16025/j.1674-1307.2020.01.018.
13. Zhang J, Xu S. Observation on the efficacy of Shufeng Jiedu Capsule in the treatment of acute exacerbation of COPD. Chin J Emerg Tradit Chin Med. 2019;28(03):505–7. doi:10.3969/j.issn.1004-745X.2019.03.036.
14. Wang T. Clinical observation of Shufeng Jiedu Capsule in treating mild to moderate patients with acute exacerbation of COPD. Pract J Card Cereb Pneum Vasc Dis. 2015;23(07):149–51. doi:10.3969/j.issn.1008-5971.2015.07.048.
15. Wang L, Lin Z, Shan S, Tang S. Clinical study on compound Xianzhuli oral solution combined with fudosteine for acute exacerbation of COPD. Mod Drugs Clin. 2020;35(06):1166–70. doi:10.7501/j.issn.1674-5515.2020.06.023.
16. He S, Zhang F. Clinical study of compound Xianzhuli oral solution combined with compound ipratropium bromide in acute exacerbation of COPD. Chin J Prim Med Pharm. 2021;28(09):1353–8. doi:10.3760/cma.j.issn.1008-6706.2021.09.015.
17. Luo X, Shen L. Clinical study of compound Xianzhuli oral solution combined with tiotropium bromide powder aerosol in acute exacerbation of COPD. Mod Drugs Clin. 2020;35(05):955–8. doi:10.7501/j.issn.1674-5515.2020.05.028.
18. Wang L, Zhao N. Clinical study of compound Xianzhuli oral solution combined with ipratropium bromide in the treatment of acute exacerbation of COPD. Mod Drugs Clin. 2017;32(08):1565–9. doi:10.7501/j.issn.1674-5515.2017.08.040.
19. Pei Y, Zhou H. Clinical study on Xianzhuli oral liquid combined with salmeterol/fluticasone in the treatment of acute exacerbation of COPD. J New Chin Med. 2019;51(04):137–9. doi:10.13457/j.cnki.jncm.2019.04.043.
20. Zheng H, Li Z. Observation on the efficacy of Xianzhuli oral liquid as adjuvant therapy in acute exacerbation of COPD. J New Chin Med. 2016;48(04):222–4. doi:10.13457/j.cnki.jncm.2016.04.084.
21. Li Z, Cheng C. Clinical observation of Xianzhuli oral liquid in the treatment of acute exacerbation of chronic obstructive pulmonary disease. Guangming J Tradit Chin Med. 2024;39(15):3046–8. doi:10.3969/j.issn.1003-8914.2024.15.027.
22. Wang Y. Clinical effect analysis of modified Qingqi Huatan Pill in the treatment of acute exacerbation of chronic obstructive pulmonary disease. Front Med. 2021;11(23):185–6.
23. Hou T, Liu R, He J, Ye C. Clinical efficacy of modified Qingqi Huatan Pill in treating acute exacerbation of COPD and its effects on inflammatory response, airway remodeling, and thrombosis mechanism. Chin J Exp Tradit Med Formulae. 2019;25(10):74–80. doi:10.13422/j.cnki.syfjx.20182427.
24. Jiang Y, Hou T, Chen Y, Yue T, Yang Z. Effect of modified Qingqi Huatan Pill on inflammatory response in AECOPD patients via HIF-1α signaling pathway. Syst Med. 2021;6(24):29–32. doi:10.19368/j.cnki.2096-1782.2021.24.029.
25. Jiang Y, Hou T, Chen Y, Yue T, Yang Z, Duan W. Clinical efficacy of modified Qingqi Huatan Pill in the treatment of acute exacerbation of COPD and its effect on MMP-9/TIMP-1 imbalance. World Complex Med. 2021;7(12):36–9,43. doi:10.11966/j.issn.2095-994X.2021.07.12.10.
26. Liu R, Hou T, He J, Ye C. Clinical efficacy of modified Qingqi Huatan Pill in treating phlegm-heat obstructing lung syndrome in acute exacerbation of COPD and its effect on serum TNF-α, IL-8, and MMP-9. Chin J Exp Tradit Med Formulae. 2019;25(09):31–7. doi:10.13422/j.cnki.syfjx.20182212.
27. Wei R, Shi S. Clinical observation of modified Qingqi Huatan Pill in treating acute exacerbation of COPD (phlegm-heat obstructing lung syndrome). Chin J Emerg Tradit Chin Med. 2020;29(10):1846–9. doi:10.3969/j.issn.1004-745X.2020.10.044.
28. Ji S, Ma D, Cui H, Chen H. Clinical observation of Qingqi Huatan Pill combined with nebulized inhalation in the treatment of COPD with phlegm and blood stasis syndrome. Pharmacol Clin Chin Mater Med. 2017;33(02):179–82. doi:10.13412/j.cnki.zyyl.2017.02.050.
29. Qu J, Tian Y, Zhao Z, Ma X, Liu Z. Clinical study on Qingqi Huatan Pill combined with salmeterol/fluticasone in the treatment of acute exacerbation of COPD. Mod Drugs Clin. 2023;38(07):1687–92. doi:10.7501/j.issn.1674-5515.2023.07.022.
30. Wu X, Wang B, Sang Y. Observation on the efficacy of Qingke Pingchuan Granules as adjunctive therapy in acute exacerbation of COPD with phlegm-heat obstructing lung syndrome. J New Chin Med. 2014;46(11):62–4. doi:10.13457/j.cnki.jncm.2014.11.024.
31. Cai Z, Peng J, Huang J, Chen L. Clinical observation of Qingke Pingchuan Granules combined with conventional biomedicine in treating acute exacerbation of COPD. Chin Tradit Herb Drugs. 2023;54(14):4584–9. doi:10.7501/j.issn.0253-2670.2023.14.019.
32. Yu X, Yan H, Su Y, Nie J, Ma Y, Liu Y, et al. Clinical efficacy of Qingke Pingchuan Granules in the treatment of acute exacerbation of COPD (phlegm-heat obstructing lung syndrome) and its effect on serum IL-6 and PCT. Chin Tradit Herb Drugs. 2024;55(11):3768–73. doi:10.7501/j.issn.0253-2670.2024.11.019.
33. Liu R, Xu C, Wang X, Wang S, Fan N, Li H, et al. Clinical study of Qingke Pingchuan Granules in the treatment of acute exacerbation of COPD with phlegm-heat obstructing lung syndrome. Mod Drugs Clin. 2024;39(12):3119–24. doi:10.7501/j.issn.1674-5515.2024.12.017.
34. Li S, Hua X. Clinical observation of Tanreqing in the treatment of acute exacerbation of chronic obstructive pulmonary disease. Chin J Pract Intern Med. 2009;29(S2):71–2.
35. Liu J. Observation on the efficacy of Tanreqing Injection in the treatment of chronic obstructive pulmonary disease. Pharmacol Clin Chin Mater Med. 2012;28(02):171–3. doi:10.13412/j.cnki.zyyl.2012.02.004.
36. Yu Q, Ouyang L. Effects of Tanreqing Injection on pulmonary vascular resistance and oxidative stress in patients with phlegm-turbid obstructing lung type COPD during acute exacerbation. Chin J Basic Med Tradit Chin Med. 2019;25(10):1380–2,403. doi:10.19945/j.cnki.issn.1006-3250.2019.10.020.
37. Xiang Y, Zhang Q, Liu J, Yang H. Clinical efficacy of Tanreqing Injection combined with salbutamol sulfate in elderly patients with acute exacerbation of COPD and its effect on inflammatory cytokines and immune function. Chin J Geriatr. 2022;42(24):5983–6. doi:10.3969/j.issn.1005-9202.2022.24.017.
38. Zhang M, Li Q, Xiong Q, Li J, Xie L, Lu J, Li Z. Evaluation of clinical efficacy of Tanreqing Injection combined with ceftazidime in the treatment of phlegm-heat obstructing lung syndrome in acute exacerbation of COPD. Chin J Exp Tradit Med Formulae. 2024;30(1):170–5. doi:10.13422/j.cnki.syfjx.20240397.
39. Zhang W, Sun Z, Liu J, Lao W. Clinical observation of Tanreqing Injection in the treatment of acute exacerbation of COPD in 37 cases. J New Chin Med. 2006;(06):48–9. doi:10.13457/j.cnki.jncm.2006.06.026.
40. Hong B, Yu Y, Yin L, Zhou Y, Bao Y. Observation on the efficacy of Tanreqing Injection in treating acute exacerbation of COPD. Chin J Mod Appl Pharm. 2008;(S2):725–7. doi:10.13748/j.cnki.issn1007-7693.2008.s2.004.
41. Zhang C, Zou X, Su Q, Wang X, Chen B. Observation on the therapeutic effect of Tanreqing Injection on acute exacerbation of chronic obstructive pulmonary disease. Jiangsu Med J. 2010;36(21):2578–9. doi:10.19460/j.cnki.0253-3685.2010.21.043.
42. Yao H, Li Q, Chen D, Sun X, Ye F, Cai Z, et al. Effect of Tanreqing Injection on lung function and pulmonary vascular resistance in phlegm-turbid obstructing type COPD. Chin Arch Tradit Chin Med. 2020;38(2):210–3. doi:10.13193/j.issn.1673-7717.2020.02.052.
43. Yao Z. Clinical observation of Reduning in the treatment of acute exacerbation of chronic obstructive pulmonary disease. Zhejiang J Tradit Chin Med. 2012;47(11):853. doi:10.3969/j.issn.0411-8421.2012.11.054.
44. Sun G, Luo Y, Zhang Q, Zhai J. Clinical study of Reduning Injection as adjuvant therapy in acute exacerbation of COPD. Guide China Med. 2012;10(32):11–3. doi:10.15912/j.cnki.gocm.2012.32.280.
45. Ma Y, Han L. Clinical study of Reduning Injection combined with compound ipratropium bromide in the treatment of acute exacerbation of COPD. Mod Drugs Clin. 2020;35(12):2430–5. doi:10.7501/j.issn.1674-5515.2020.12.029.
46. Yu S, Zhang L, Kang Q, Qiu Y, Wu T. Clinical efficacy of Reduning Injection combined with antibiotics in treating acute exacerbation of COPD. J Hubei Univ Chin Med. 2022;24(06):18–21. doi:10.3969/j.issn.1008-987x.2022.06.04.
47. Shao X, Le Z, Liu R, Zhu R. Evaluation of efficacy and safety of Reduning Injection combined with salmeterol/fluticasone in patients with acute exacerbation of COPD. World J Integr Tradit West Med. 2023;18(01):93–7,103. doi:10.13935/j.cnki.sjzx.230114.
48. Tang H. Study on the efficacy of Reduning Injection in acute exacerbation of COPD and its effect on inflammatory cytokines and lung function. Shanxi Med J. 2021;50(2):257–60. doi:10.3969/j.issn.0253-9926.2021.02.035.
49. Pang L, Zang N, Liu C, Zheng W, Lü X. Anti-inflammatory and immunomodulatory effects of Reduning Injection in phlegm-heat obstructing lung syndrome of acute exacerbation of COPD. World Sci Technol Mod Tradit Chin Med. 2015;17(06):1225–9. doi:10.11842/wst.2015.06.019.
50. Zeng J, Zhang J, Zhang H. Clinical observation of Reduning Injection in acute exacerbation of chronic obstructive pulmonary disease. J Emerg Crit Care Med Intern Med. 2014;20(01):43,59. doi:10.11768/nkjwzzzz20140117.
51. Chen W, Lin J. Clinical observation of Reduning Injection in 77 cases of acute exacerbation of COPD. J Tradit Chin Med Inform. 2014;20(13):69–71. doi:10.13862/j.cnki.cn43-1446/r.2014.13.025.
52. Lu S. Evaluation of the efficacy of Reduning Injection in treating acute exacerbation of COPD. World Latest Med Inf (Dig). 2018;18(86):189. doi:10.19613/j.cnki.1671-3141.2018.86.110.
53. Wan C. Clinical analysis of Reduning Injection in the treatment of acute exacerbation of COPD. Guide China Med. 2013;11(35):192–3. doi:10.15912/j.cnki.gocm.2013.35.040.
54. Wei Y. Clinical value analysis of Reduning Injection in the treatment of acute exacerbation of COPD. Shenzhen J Integr Tradit Chin West Med. 2014;24(05):29–30. doi:10.16458/j.cnki.1007-0893.2014.05.038.
55. Zhou J. Clinical efficacy observation of Reduning Injection in acute exacerbation of COPD. J Pract Cardiopulm Cerebrovasc Dis. 2014;22(11):83–4. doi:10.16808/j.cnki.issn1003-7705.2021.12.001.
56. Peng Q, Liu Q, Liu C, Liu M, Li L. Efficacy of Reduning Injection in treating AECOPD and its effect on TLR4/NF-κB inflammatory signaling pathway. Hunan J Tradit Chin Med. 2021;37(12):1–4,20. doi:10.16808/j.cnki.issn1003-7705.2021.12.001.
57. Sun Z, Li C, Lei S, Peng F, He X, Wu S. Clinical efficacy of Shiwai Longdanhua Capsule combined with conventional treatment in acute exacerbation of COPD patients. Chin J Tradit Chin Med. 2022;44(06):2063–6. doi:10.3969/j.issn.1001-1528.2022.06.063.
58. Fu Q, Guo D. Effects of Shiwai Longdanhua Capsule combined with salbutamol sulfate on cellular immune function, inflammatory cytokines, and coagulation function in elderly patients with acute exacerbation of COPD. Chin J Geriatr. 2021;41(21):4697–9. doi:10.3969/j.issn.1005-9202.2021.21.030.
59. Liu P, Shi X, Yang M. Clinical study of Shiwai Longdanhua Capsule combined with tiotropium bromide in acute exacerbation of COPD. Mod Drugs Clin. 2020;35(07):1341–4. doi:10.7501/j.issn.1674-5515.2020.07.011.
60. Peng G, Liu X, Zhang D, Long X. Clinical efficacy observation of Shiwai Longdanhua Capsule combined with salmeterol/fluticasone in acute exacerbation of COPD and its effect on airway remodeling. Chin J Tradit Chin Med. 2019;37(10):2487–91. doi:10.13193/j.issn.1673-7717.2019.10.044.
61. Zhang W, Fan C, Gao J, Wang B, Zheng J. Clinical study of Shiwai Longdanhua Capsule combined with biomedicine in acute exacerbation of COPD. Chin J Tradit Chin Med Inf. 2015;(2):13–6. doi:10.3969/j.issn.1005-5304.2015.02.005.
62. Lei J, Li J, An Y. Clinical efficacy of Shiwai Longdanhua Capsule combined with biomedicine in AECOPD and its effect on serum PCT, CRP, IL-6, and MDA levels. Harbin Med J. 2019;39(05):485–6.
63. Reyihanguli A, Axian G. Regulatory effect of Zhichuanling Injection combined with tiotropium bromide inhalation powder on Th17/regulatory T cell balance in patients with acute exacerbation of COPD (phlegm-turbid obstructing lung syndrome). China Hosp Pharm Eval Anal. 2022;22(04):407–10. doi:10.14009/j.issn.1672-2124.2022.04.005.
64. Zhang L, Liu L, Wang W. Clinical observation of nebulized Zhichuanling Injection in acute exacerbation of COPD. World J Tradit Chin Med. 2017;12(07):1562–5. doi:10.3969/j.issn.1673-7202.2017.07.020.
65. Zhang A, Chen J, Wang S, Yan X, Cui Y, Wang Y, et al. Clinical study of Zhichuanling Injection in acute exacerbation of COPD. Hebei J Tradit Chin Med. 2025;47(03):410–3,8. doi:10.3969/j.issn.1002-2619.2025.03.013.
66. Zhao N, Tang P, Zhu W. Clinical efficacy of doxophylline combined with Zhichuanling oral liquid in acute exacerbation of COPD and its effect on lung function and inflammatory cytokines. Reflexotherapy Rehabil Med. 2024;5(13):157–60.
67. Zeng Y, Yang R, Wang L, Zou X, Xiong W. Effect of Qingkailing Injection on immune function in patients with acute exacerbation of COPD. Med Herald. 2009;28(01):65–8. doi:10.3870/yydb.2009.01.023.
68. Sun F. Effect of Qingkailing Injection on serum cytokines in patients with COPD. Chin J Pharmacology Clin. 2001;(01):43–4. doi:10.3969/j.issn.1001-859X.2001.01.027.
69. Ding Z. Effect of Qingkailing Injection on lung function and serum IL-8 in acute exacerbation of COPD. Zhejiang J Tradit Chin Med. 2013;48(03):174. doi:10.3969/j.issn.0411-8421.2013.03.011.
70. Li B, Lu W, Wu Z, Luo R. Clinical study of Qingkailing Injection combined with tiotropium bromide in treatment of COPD. Mod Drugs Clin. 2016;31(06):784–7. doi:10.7501/j.issn.1674-5515.2016.06.012.
71. Cao X. Observation on efficacy of Qingkailing Injection in treating 60 cases of COPD. China Community Doctor. 2012;28(14):14.
72. Chang Y, Zhang J, Chen B. Clinical observation of Qingkailing Injection in acute exacerbation of COPD. Chin J Tradit Chin Med Emerg. 2006;(05):490–1. doi:10.3969/j.issn.1004-745X.2006.05.024.
73. Yang K, He J. Clinical efficacy observation of Qingkailing Injection in COPD. J Clin Pulmonol. 2009;14(07):981–2. doi:10.3969/j.issn.1009-6663.2009.07.078.
74. He L. Clinical experience of Qingkailing Injection in treating COPD. Hebei Med J. 2008;(02):213–4. doi:10.3969/j.issn.1006-6233.2008.02.039.
75. Xie J, Deng S, Li X, Qu G, Peng X. Effect of Andrographolide on patients with acute exacerbation of COPD. Guangzhou Med. 2022;53(01):6–11. doi:10.3969/j.issn.1000-8535.2022.01.002.
76. Zeng Q. Clinical efficacy observation of Xiyanping Injection in acute exacerbation of COPD. Chin J Mod Appl Pharm. 2018;12(18):81–3. doi:10.14164/j.cnki.cn11-5581/r.2018.18.045.
77. Liang R, Shao Y, Jin S. Efficacy of Xiyanping Injection in acute exacerbation of COPD. J Harbin Med Univ. 2012;46(01):78–81. doi:10.3969/j.issn.1000-1905.2012.01.021.
78. Chang C. Effect of Xiyanping Injection in acute exacerbation of COPD. Dajia Health (Acad Ed). 2015;9(08):131–2. doi:10.3969/j.issn.1009-6019.2015.04.149.
79. Zhang X, Wang Y, Guo A. Effects of Xiyanping on inflammatory factors and lung function in elderly patients with acute exacerbation of COPD. Chin J Integr Clin. 2014;30(09):932–5. doi:10.3760/cma.j.issn.1008-6315.2014.09.012.
80. Han D, Li F. Clinical study of Xiyanping on lung function and inflammatory factors in acute exacerbation of COPD. Chin Primary Health Care. 2012;(07):1078–9. doi:10.3760/cma.j.issn.1008-6706.2012.07.074.
81. Zhang W, Chen X. Observation of Xiyanping adjuvant therapy in elderly patients with acute exacerbation of COPD. Global Tradit Chin Med. 2011;4(04):304–5. doi:10.3969/j.issn.1674-1749.2011.04.022.
82. Liu H, Dong S, Li J, Li A. Clinical efficacy of Xiyanping as adjuvant treatment in acute exacerbation of COPD. Trop Med J. 2016;16(10):1293–5.
83. Zhang N, Liu Q. Efficacy and effect on lung function of Xiyanping adjuvant treatment for acute exacerbation of COPD. Bethune Med J. 2017;15(01):85–6. doi:10.16485/j.issn.2095-7858.2017.01.043.
84. Han F, Zhang X. Clinical observation of Xiyanping in acute exacerbation of COPD. Mod J Integr Tradit Chin West Med. 2015;24(08):870–2. doi:10.3969/j.issn.1008-8849.2015.08.029.

Table S3.2: Characteristics of included Chinese patent medicines

| CPMs | TCM function(s) of CPMs | Ingredients of CPMs formula | Chemical components & Pharmacological effects of CPMs |
| --- | --- | --- | --- |
| Shufeng Jiedu Capsule (SFJD) | **Disperses wind, clears heat, detoxifies and relieves sore throat,alleviates cough and resolves phlegm.**  It is used for treating sore throat, cough, fever, and headache caused by wind-heat invasion, as well as upper respiratory tract infections, acute pharyngitis, or tonsillitis presenting with wind-heat syndromes. | - *[Forsythia suspensa](https://www.worldfloraonline.org/taxon/wfo-0000832217)*[(Thunb.) Vahl](https://www.worldfloraonline.org/taxon/wfo-0000832217) - *[Reynoutria japonica](https://www.worldfloraonline.org/taxon/wfo-0000832217)* [Houtt](https://www.worldfloraonline.org/taxon/wfo-0000832217) - *[Patrinia scabiosifolia](https://www.worldfloraonline.org/taxon/wfo-0000832217)*[Link](https://www.worldfloraonline.org/taxon/wfo-0000832217) - *[Isatis tinctoria](https://www.worldfloraonline.org/taxon/wfo-0000832217)* [L](https://www.worldfloraonline.org/taxon/wfo-0000832217) - *[Phragmites australis](https://www.worldfloraonline.org/taxon/wfo-0000832217)* [(Cav.) Trin. ex Steud](https://www.worldfloraonline.org/taxon/wfo-0000832217) - *[Glycyrrhiza uralensis](https://www.worldfloraonline.org/taxon/wfo-0000832217)* [Fisch.ex DC](https://www.worldfloraonline.org/taxon/wfo-0000832217) - *[Verbena officinalis](https://www.worldfloraonline.org/taxon/wfo-0000832217)* [L](https://www.worldfloraonline.org/taxon/wfo-0000832217) - *[Hohenackeria bupleurifolia](https://www.worldfloraonline.org/taxon/wfo-0000832217)* [Fisch. & C.A.Mey](https://www.worldfloraonline.org/taxon/wfo-0000832217) | 1. *Forsythia suspensa*  Chemical components: Forsythiaside A, forsythin, protocatechuic acid, quercetin  Pharmacological effects: Anti-inflammatory, antibacterial, antiviral, antioxidant   1. *Reynoutria japonica*   Chemical components: Emodin, resveratrol, polydatin, flavonoids  Pharmacological effects: Antioxidant, antibacterial and cardioprotective activities  3. *Patrinia scabiosifolia*  Chemical components: Flavonoids, triterpenoids, organic acids and volatile oils  Pharmacological effects: Antibacterial, antitumor, immunomodulatory, antioxidant  4. *Isatis tinctoria*  Chemical components: Indigotin, indirubin, indole derivatives, polysaccharides  Pharmacological effects:Antiviral,immunomodulatory, and detoxifying activities  5. *Phragmites australis*  Chemical components:Polysaccharides, flavonoids, volatile oils  Pharmacological effects: Diuretic, antipyretic, anti-inflammatory   1. *Glycyrrhiza uralensis*   Chemical components: Glycyrrhizic acid, flavonoids, glycyrrhizic saponins  Pharmacological effects:Anti-inflammatory, antiviral, antioxidant,antispasmodic  7. *Verbena officinalis*  Chemical components: Coumarins, flavonoids, triterpenoids, volatile oils  Pharmacological effects: Antibacterial, circulation-promoting, analgesic  8. *Hohenackeria bupleurifolia*  Chemical components:Saikosaponins, flavonoids, volatile oils  Pharmacological effects: Antipyretic, hepatoprotective, antidepressant activities |
| Fresh Bamboo Juice Oral Liquid (XZL) | **Clears heat, resolves phlegm, and relieves cough.**  It is indicated for conditions caused by phlegm-heat obstructing the lung, such as cough, thick yellow sputum, sore throat, and restlessness due to internal heat, as well as for patients with lung-heat cough presenting with these manifestations. | - *Succus Bambusae* - *Houttuynia cordata* Thunb - *Pinellia ternata* (Thunb.) Makino - *Zingiber officinale* Roscoe - *Eriobotrya japonica* (Thunb.) Lindl - *[Platycodon grandiflorus A.DC](https://www.worldfloraonline.org/taxon/wfo-0000816758)* - *Mentha canadensis* L | 1. *Succus Bambusae*  Chemical components: Bamboo quinones, flavonoids, organic acids  Pharmacological effects: Heat-clearing, phlegm-resolving, antitussive   1. *Houttuynia cordata*   Chemical components: Volatile oils, flavonoids, polysaccharides, alkaloids  Pharmacological effects: Antioxidant, and heat-clearing detoxifying activities  3. *Pinellia ternata*  Chemical components: Alkaloids, volatile oils, saponins, steroids, amino acids  Pharmacological effects: Phlegm-resolving and cough-relieving, anti-inflammatory  4. *Zingiber officinale*  Chemical components: volatile oils, shogaols, flavonoids, organic acids  Pharmacological effects: Exterior-releasing and cold-dispersing, antiemetic  5. *Eriobotrya japonica*  Chemical components:Triterpenoids, flavonoids, polysaccharides, organic acids  Pharmacological effects: Lung-clearing and phlegm-resolving, cough-relieving  6. *Platycodon grandiflorus*  Chemical components:Saponins, polysaccharides, flavonoids, and volatile oils  Pharmacological effects: Expectorant, antitussive, anti-inflammatory  7. *Mentha canadensis*  Chemical components: Menthol, menthone, flavonoids, phenolic compounds  Pharmacological effects:Exterior-releasing, heat-clearing, cooling analgesic |
| Qingqi Huatan Pill (QQHT) | **Clears heat, resolves phlegm, relieves cough, and descends adverse Qi.**  It is used to treat cough, thick yellow sputum, chest tightness and shortness of breath because of phlegm-heat obstructing the lung, as well as chronic bronchitis, or lung-heat cough who present with these symptoms. | - *Arisaema erubescens* (Wall.) Schott - *Trichosanthes kirilowii* Maxim - *Scutellaria baicalensis* Georgi - *Pinellia ternata*(Thunb.) Makino - *Citrus japonica* Thunb - *Prunus sibirica*L - *Wolfiporia cocos* (F.A. Wolf) Ryvarden & Gilb - *Citrus trifoliata* L - *Zingiber officinale* Roscoe | 1. *Arisaema erubescens*   Chemical constituents: Bile acids, saponins, alkaloids, amino acids  Pharmacological effects: Anticonvulsant, wind-dispelling antispasmodic activities   1. *Trichosanthes kirilowii*   Chemical components: Trichosanthin saponins, polysaccharides, proteins  Pharmacological effects: Heat-clearing and phlegm-resolving, antitumor   1. *Scutellaria baicalensis*   Chemical components: Baicalin and wogonin, sterols, organic acids,polysaccharides  Pharmacological effects: Heat-clearing and dampness-drying,anti-inflammatory   1. *Pinellia ternata*   Chemical components: Alkaloids, volatile oils, saponins, steroids, amino acids  Pharmacological effects: Phlegm-resolving and cough-relieving, anti-inflammatory   1. *Citrus japonica*   Chemical components: volatile oils , flavonoids, polysaccharides, organic acids  Pharmacological effects: Regulating Qi and drying dampness, resolving phlegm   1. *Prunus sibirica*   Chemical components: Amygdalin, volatile oils, fixed oils, unsaturated fatty acids  Pharmacological effects: Antitussive, expectorant activities,anti-inflammatory   1. *Wolfiporia cocos*   Chemical components: Triterpenoids, polysaccharides, poricoic acids, sterols  Pharmacological effects: Diuretic and dampness-draining, spleen-strengthening   1. *Citrus trifoliata*   Chemical components: Volatile oils, flavonoids, neohesperidin  Pharmacological effects: Regulating Qi and relieving distension, anti-inflammatory   1. *Zingiber officinale*   Chemical components: volatile oils, shogaols, flavonoids, organic acids  Pharmacological effects: Exterior-releasing and cold-dispersing, antiemetic |
| Qingke Pingchuan Granule(QKPC) | **Clears heat, disperses lung, relieves cough and suppresses asthma.**  It is indicated for respiratory conditions characterized by phlegm-heat, manifesting as cough, sputum overproduction, chest oppression, and wheezing, particularly during acute episodes of chronic bronchitis or bronchial asthma. | - *Ephedra wraithiana* I.M.Johnst - *Prunus sibirica*L - *Gypsum Fibrosum* - *Glycyrrhiza glabra* L - *Fagopyrum dibotrys*(D.Don) Hara - *Houttuynia cordata* Thunb - *Fritillaria sichuanica* S.C.Chen - *Ardisia japonica* (Thunb.) Blume - *Eriobotrya japonica* (Thunb.) Lindl - *Perilla ocymoides* L  \|  \| \| --- \| | 1. *Ephedra wraithiana*   Chemical components: Alkaloids, flavonoids, volatile oils, tannins  Pharmacological effects: Sweating and releasing the exterior, dispersing the lung and relieving asthma  2. *Prunus sibirica*  Chemical components: Amygdalin, volatile oils, fixed oils, unsaturated fatty acids  Pharmacological effects: Antitussive, expectorant activities,anti-inflammatory  3. *Gypsum Fibrosum*  Chemical components: Calcium sulfate dihydrate  Pharmacological effects:Clearing heat and purging fire, relieving thirst  4. *Glycyrrhiza glabra*  Chemical components: Glycyrrhizic acid, flavonoids, glycyrrhizic saponins  Pharmacological effects:Anti-inflammatory, antiviral, antioxidant,antispasmodic  5. *Fagopyrum dibotrys*  Chemical components: Flavonoids, organic acids, polysaccharides  Pharmacological effects: Clearing heat and detoxification, anti-inflammatory   1. *Houttuynia cordata*   Chemical components: Volatile oils, flavonoids, polysaccharides, alkaloids  Pharmacological effects: Antioxidant, and heat-clearing detoxifying activities   1. *Fritillaria sichuanica*   Chemical components:Steroidal alkaloids, polysaccharides, volatile oils  Pharmacological effects: Moistening the lungs and relieving cough, expectorant   1. *Ardisia japonica*   Chemical components:Triterpenoid saponins, flavonoids, volatile oils, organic acids  Pharmacological effects: Clearing heat and detoxification, antimicrobial   1. *Eriobotrya japonica*   Chemical components:Triterpenoids, flavonoids, polysaccharides, organic acids  Pharmacological effects: Lung-clearing and phlegm-resolving, cough-relieving   1. *Perilla ocymoides*   Chemical components:Volatile oils, flavonoids, sterols, polysaccharides  Pharmacological effects: Dispersing the lung to relieve cough, antiallergic |
| Tanreqing Injection (TRQ) | **Clears heat, resolves phlegm, and detoxification.**  It is used to treat symptoms associated with phlegm-heat and internal toxin accumulation, including productive cough with yellow sputum, chest congestion, wheezing or fever, applicable to acute or chronic respiratory infections. | - *Scutellaria baicalensis* Georgi - *Capra hircus* L - *Ursus thibetanus* G. Cuvier - *Lonicera japonica* Thunb - *[Forsythia suspensa](https://www.worldfloraonline.org/taxon/wfo-0000832217)*[(Thunb.) Vahl](https://www.worldfloraonline.org/taxon/wfo-0000832217) | 1. *Scutellaria baicalensis*  Chemical components: Baicalin and wogonin, sterols, organic acids,polysaccharides  Pharmacological effects: Heat-clearing and dampness-drying,anti-inflammatory  2. *Capra hircus*  Chemical components: Keratin, collagen, peptides, alkaloids  Pharmacological effects: Clearing heat and calming the liver, sedative  3. *Ursus thibetanus*  Chemical components: Ursodeoxycholic acid, cholic acid, deoxycholic acid  Pharmacological effects: Clearing heat and detoxification, hepatoprotective  4. *Lonicera japonica*  Chemical components: Chlorogenic acid, luteoloside, lonicerin, volatile oils  Pharmacological effects: Clearing heat and detoxification, anti-inflammatory  5. *Forsythia suspensa*  Chemical components: Forsythiaside, phillyrin, chlorogenic acid, quercetin  Pharmacological effects:Clearing heat and detoxification, antibacterial |
| Reduning Injection (RDN) | **Clears heat, dispells wind,and detoxifies effects.**  It is indicated for treating patients with symptoms caused by heat-toxin syndrome, such as fever, sore throat, swelling, and pain, as well as those suffering from infections or inflammatory conditions presenting with these symptoms. | - *Artemisia annua* L - *Lonicera japonica* Thunb - *Gardenia jasminoides* J.Ellis | 1.*Artemisia annua*  Chemical components:V olatile oils, flavonoids, sesquiterpenes, phenolic acids  Pharmacological effects: Antibacterial, anti-inflammatory, antioxidant, anthelmintic  2. *Lonicera japonica*  Chemical components: Chlorogenic acid, luteoloside, lonicerin, volatile oils  Pharmacological effects: Clearing heat and detoxification, anti-inflammatory  3. *Gardenia jasminoides*  Chemical components: Geniposide, genipin, gardenoside, flavonoids  Pharmacological effects: Heat-clearing and fire-purging, hepatoprotective,sedative |
| Shiwei Longdanhua Capsule(SWLDH) | **Clears heat and resolves phlegm, relieves cough and asthma.**  It is used to treat cough, wheezing, and yellow phlegm caused by phlegm-heat obstructing the lungs, with or without fever, as well as patients with infectious or inflammatory diseases presenting these symptoms. | - *Gentiana scabra*Bunge - *Rhododendron anthopogonoides* Maxim - *Glycyrrhiza uralensis* Fisch. ex DC - *Przewalskia tangutica* Maxim - *Corydalis hendersonii* Hemsl - *Fritillaria sichuanica* S.C.Chen - *Berberis amurensis* Rupr - *Codonopsis convolvulacea* Kurz var. convolvulacea - *Phlomis younghusbandii* Mukerjee - *Inula racemosa* Hook.f  \| \|  \| \| --- \| \| \| --- \| --- \| | 1. *Gentiana scabra*  Chemical components: Gentiopicroside,flavonoids, xanthones, alkaloids  Pharmacological effects: Liver-cleansing and heat-clearing, anti-inflammatory  2. *Rhododendron anthopogonoides*  Chemical components: Volatile oils, flavonoids, triterpenoids, phenolic compounds  Pharmacological effects: Wind-dispersing and dampness-drying, antioxidant  3. *Glycyrrhiza uralensis*  Chemical components: Paeoniflorin, paeonol, benzoic acid  Pharmacological effects: Analgesic, anti-inflammatory, improves blood circulation  4. *Przewalskia tangutica*  Chemical components: Alkaloids, flavonoids, saponins, steroids  Pharmacological effects: Heat-clearing and detoxifying, expectorant properties  5. *Corydalis hendersonii*  Chemical components: Isoquinoline Alkaloids, Phenolic Glycosides  Pharmacological effects: Anticancer,Cardioprotective,Anti-inflammatory  6. *Fritillaria sichuanica*  Chemical components: Saikosaponins, flavonoids, polysaccharides  Pharmacological effects: Anti-inflammatory, hepatoprotective, immune regulation   1. *Berberis amurensis*   Chemical components: Isoquinoline alkaloids, flavonoids, phenolic compounds  Pharmacological effects:Heat-clearing and detoxifying, anti-inflammatory  8. *Codonopsis convolvulacea*  Chemical components: Polysaccharides, alkaloids, saponins, flavonoids  Pharmacological effects: Immunomodulatory, antioxidant, anti-inflammatory  *9. Phlomis younghusbandii*  Chemical components:Flavonoids, phenolic acids, triterpenoids, volatile oils  Pharmacological effects: Anti-inflammatory,immunomodulatory,antioxidant  10. *Inula racemosa*  Chemical components: Sesquiterpene lactones , essential oils, flavonoids, sterols  Pharmacological effects: Anti-inflammatory, bronchodilatory, expectorant |
| Zhichuanling Injection (ZCL) | **Bronchodilation, antitussive, and expectorant.**  It is indicated for treating symptoms with asthma, wheezing, cough, and sputum production because of respiratory diseases characterized by bronchial obstruction or bronchospasm | - *Ephedra wraithiana* I.M.Johnst - *Prunus sibirica*L - *Datura stramonium* L - *[Forsythia suspensa](https://www.worldfloraonline.org/taxon/wfo-0000832217)*[(Thunb.) Vahl](https://www.worldfloraonline.org/taxon/wfo-0000832217) | 1. *Ephedra wraithiana*  Chemical components: Alkaloids, flavonoids, volatile oils, tannins  Pharmacological effects: Sweating and releasing the exterior, dispersing the lung and relieving asthma  2. *Prunus sibirica*  Chemical components: Amygdalin, volatile oils, fixed oils, unsaturated fatty acids  Pharmacological effects: Antitussive, expectorant activities,anti-inflammatory  3. *Datura stramonium*  Chemical components: Tropane alkaloids, flavonoids, volatile oils, organic acids.  Pharmacological effects: Analgesic, antispasmodic, sedative, anticholinergic  4. *Forsythia suspensa*  Chemical components: Forsythiaside, phillyrin, chlorogenic acid, quercetin  Pharmacological effects:Clearing heat and detoxification, antibacterial |
| Qingkailing Injection (QKL) | **Clears heat and detoxify, resolves phlegm and unblocks collaterals, awaken the mind and opens orifices.**  It is indicated for treating symptoms caused by heat toxins and phlegm obstructing the meridians, such as fever, headache, dizziness, impaired consciousness, and convulsions, as well as patients with infectious or inflammatory diseases exhibiting these symptoms. | - *Acidum cholicum* - *Chenodeoxycholic Acid* - *Bubalus bubalis* L - *Scutellaria baicalensis* Georgi - *Pteria martensii* (Dunker) - *Gardenia jasminoides* J.Ellis - *Isatis tinctoria* L - *Lonicera japonica* Thunb | 1.*Acidum cholicum*  Chemical components: Hydroxyl groups, a carboxylic acid side chain  Pharmacological effects: Facilitating emulsification and absorption of dietary fats  2. *Chenodeoxycholic Acid*  Chemical components: Hydroxyl groups, a terminal carboxyl group  Pharmacological effects: Stimulating bile flow, dissolves cholesterol gallstones  3. *Bubalus bubalis*  Chemical components: Proteins, amino acids, inorganic salts  Pharmacological effects: Heat-clearing, cooling blood and arresting convulsions  4. *Scutellaria baicalensis*  Chemical components: Baicalin and wogonin, sterols, organic acids,polysaccharides  Pharmacological effects: Heat-clearing and dampness-drying,anti-inflammatory  5. *Pteria martensii*  Chemical components: Calcium carbonate, magnesium, strontium, trace  Pharmacological effects: Tranquilizing and sedative, calming the liver  6. *Gardenia jasminoides*  Chemical components: Geniposide, genipin, gardenoside, flavonoids  Pharmacological effects: Heat-clearing and fire-purging, hepatoprotective,sedative  7. *Isatis tinctoria*  Chemical components:Alkaloids, nucleosides, organic acids, polysaccharides  Pharmacological effects:Heat-clearing and detoxifying, antiviral, antibacterial  8. *Lonicera japonica*  Chemical components: Chlorogenic acid, luteoloside, lonicerin, volatile oils  Pharmacological effects: Clearing heat and detoxification, anti-inflammatory |
| Xiyanping Injection (XYP) | **Clears heat and detoxify, relieves cough and stops diarrhea.**  It is used to manage symptoms resulting from toxic-heat and inflammation,  including fever, sore throat, cough, yellow sputum, and respiratory discomfort,  suitable for patients with acute upper respiratory tract infections, bronchitis, or pneumonia who exhibit these clinical manifestations. | - *Andrographis paniculata*(Burm.f.) Wall. ex Nee  \|  \| \| --- \| | *Andrographis paniculata*  Chemical components: Diterpenoid lactones, flavonoids, phenolic acids.  Pharmacological effects: Anti-inflammatory, antiviral, antibacterial, antipyretic |

Table S3.3: Detailed informations of included Chinese patent medicines

| CPMs | Source | Batch number | Specification | Prescription | Quality Control Marker Compounds | Method of extraction |
| --- | --- | --- | --- | --- | --- | --- |
| Shufeng Jiedu Capsule (SFJD) | Anhui Jiren Pharmaceutical Co., Ltd | China Drug Approval Number Z20090047 | 0.52 g per capsule | *Forsythia suspensa* 360g  *Reynoutria japonica* 450g  *Patrinia scabiosifolia* 360g  *Isatis tinctoria* 360g  *Phragmites australis* 270g  *Glycyrrhiza uralensis* 180g  *Verbena officinalis* 360g  *Hohenackeria bupleurifolia* 360g | Each capsule contains not less than 0.20 mg of forsythoside (C₂₇H₃₄O₁₁), calculated as the content derived from Forsythia suspensa, and not less than 3.0 mg of polydatin (C₂₀H₂₂O₈), calculated as the content derived from Polygonum cuspidatum. | *Reynoutria japonica* and *Isatis tinctoria* were extracted with ethanol and concentrated. *Forsythia suspensa* and *Hohenackeria bupleurifolia* underwent volatile oil extraction. *Patrinia scabiosifolia* and other herbs were water-extracted and concentrated. All extracts were mixed with excipients (dextrin and colloidal silicon dioxide), granulated, blended with volatile oil, and encapsulated into 1000 capsules. |
| Fresh Bamboo Juice Oral Liquid (XZL) | Zhejiang Taikang Pharmaceutical Group Co., Ltd | China Drug Approval Number Z36021505 | 10 or 20 ml per vial | *Succus Bambusae* 400ml  *Houttuynia cordata* 150g  *Pinellia ternata* 25g  *Zingiber officinale* 25g  *Eriobotrya japonica* 150g  *Platycodon grandiflorus*75g  *Mentha canadensis* 1ml | Each 1 mL contains not less than 2.5 μg of salicylic acid (C₇H₆O₃), calculated as the content derived from *Succus Bambusae.* | Ginger juice was ethanol-extracted, while *Houttuynia cordata* was steam-distilled. The residues were decocted with *Pinellia ternata* and other herbs, concentrated, alcohol-precipitated, then mixed with *Succus Bambusae*, sugar, ginger juice, *Houttuynia cordata*, and *Mentha canadensis* oil to make 1000 ml final product. |
| Qingqi Huatan Pill (QQHT) | Henan Jinhongtang Pharmaceutical Co., Ltd. | China Drug Approval Number Z41021649 | 6 g per sachet | *Arisaema erubescens* 150g  *Trichosanthes kirilowii* 100g  *Scutellaria baicalensis* 100g  *Pinellia ternata* 150g  *Citrus japonica* 100g  *Prunus sibirica* 100g  *Wolfiporia cocos* 100g  *Citrus trifoliata* 100g  *Zingiber officinale* 100g | Each 1 g of the product contains not less than 6.5 mg of baicalin (C₂₁H₁₈O₁₁), calculated as the content derived from Radix Scutellariae. | All ingredients except *Trichosanthes kirilowii* were pulverized into fine powder, mixed with the seeds, and sieved. *Zingiber officinale* was crushed, pressed with water to obtain juice, then used to prepare pill cores by layering with the powder mixture. The pills were dried to yield the final product |
| Qingke Pingchuan Granule(QKPC) | Changchun Leiyunshang Pharmaceutical Co., Ltd | China Drug Approval Number Z20040047 | 10 g per sachet | *Ephedra wraithiana*135g  *Prunus sibirica* 135g  *Gypsum Fibrosum* 450g  *Glycyrrhiza glabra* 135g  *Fagopyrum dibotrys* 300g  *Houttuynia cordata* 300g  *Fritillaria sichuanica* 135g  *Ardisia japonica 225g*  *Eriobotrya japonica* 135g  *Perilla ocymoides* 135g | Each sachet contains not less than 25 mg of amygdalin (C₂₀H₂₇NO₁₁), calculated as the content derived from *Prunus sibirica*. | *Fritillaria sichuanica* coarse powder was macerated in 70% ethanol for 48h and percolated to obtain 1000ml extract; *Houttuynia cordata* was steam-distilled for 3h; gypsum was pre-decocted, then combined with other herbs (including *Ephedra wraithiana*) for triple decoction. The extracts were concentrated to 1.30-1.35 density (50℃), mixed with *Fritillaria sichuanica* extract, granulated with sucrose (330g) and dextrin, then sprayed with volatile oil to produce 1000g granules. |
| Tanreqing Injection (TRQ) | Shanghai Kaibao Pharmaceutical Co., Ltd | China Drug Approval Number Z20030054 | 10 mL per vial | *Scutellaria baicalensis*  *Capra hircus*  *Ursus thibetanus*  *Lonicera japonica*  *Forsythia suspensa* | Each 1 mL contains 5.40-5.46 mg of baicalin (C₂₁H₁₈O₁₁) from *Scutellaria baicalensis* and 0.077-0.145 mg of forsythoside E (C₂₉H₃₆O₁₅) from *Forsythia suspensa.* | *Ursus thibetanus* was saponified and acid-precipitated to obtain total bile acids; goat horn was hydrolyzed and alcohol-precipitated for amino acids; *Scutellaria baicalensis*, *Lonicera japonica*, and *Forsythia suspensa* were extracted and purified via ethanol precipitation and solvent extraction. All extracts were combined with propylene glycol (10%), carbon-treated, pH-adjusted (7.0-8.0), sterile-filtered, and terminally sterilized (121°C, 20min). |
| Reduning Injection (RDN) | Jiangsu Kangyuan Pharmaceutical Co., Ltd | China Drug Approval Number Z20050217 | 10 mL per vial | *Artemisia annua250g*  *Lonicera japonica 750g*  *Gardenia jasminoides 600g* | Each 1 mL contains 1.12% to 2.41% of solid content, calculated as the combined extract of *Lonicera japonica* and *Artemisia annua*. | The preparation involved three key extraction processes: *Lonicera japonica* underwent water extraction with alcohol precipitation and acidic ethyl acetate extraction, *Artemisia annua* was processed for volatile oil distillation and aqueous extraction, while Gardenia jasminoides was extracted with ethanol followed by acidic n-butanol purification. The combined extracts were pH-adjusted, treated with activated carbon, solubilized using HS-15, and sequentially processed through ultrafiltration and 0.22 μm sterile filtration under aseptic conditions. |
| Shiwei Longdanhua Capsule(SWLDH) | Tibet Tibetan Pharmaceutical Group Co., Ltd | China Drug Approval Number Z20010046 | 0.45 g per capsule | *Gentiana scabra*  *Rhododendron anthopogonoides*  *Glycyrrhiza uralensis*  *Przewalskia tangutica*  *Corydalis hendersonii*  *Fritillaria sichuanica*  *Berberis amurensis*  *Codonopsis convolvulacea*  *Phlomis younghusbandii*  *Inula racemosa* | Each 1 g contains not less than 1.25 mg of gentiopicroside (C₁₆H₂₀O₉), calculated as the content derived from *Gentiana scabra*, and not less than 1.35 mg of berberine (C₂₀H₁₈NO₄), calculated as the content derived from *Berberis amurensis*. | All herbs are pulverized into fine powder, sieved, uniformly mixed, sterilized, and encapsulated into 1000 capsules. |
| Zhichuanling Injection (ZCL) | Jiangsu Suzhong Pharmaceutical Group Co., Ltd | China Drug Approval Number Z10910007 | 10 mL per vial | *Ephedra wraithiana* 150g  *Prunus sibirica* 150g  *Datura stramonium* 30g  *Forsythia suspensa* 150*g* | Each 1 mL contains 0.50 to 0.80 mg of total *Forsythia suspensa*, calculated as ephedrine (C₁₀H₁₅NO), and not less than 15μg of scopolamine(C₁₇H₂₁NO₄), calculated as the content derived from *Datura stramonium*. | The four herbs were decocted twice in water. The combined decoction was concentrated to 150 mL, then sequentially precipitated with 70% and 85% ethanol. After concentration, the solution was diluted to 800 mL, pH-adjusted, brought to a final volume of 1000 mL with water for injection, filled, and sterilized. |
| Qingkailing Injection (QKL) | Yabao Sichuan Pharmaceutical Co., Ltd | China Drug Approval Number Z20163036 | 10 mL per vial | *Acidum cholicum* 3.25g  *Chenodeoxycholic Acid* 3.75g  *Bubalus bubalis* 25g  *Scutellaria baicalensis* 5g  *Pteria martensii* 50g  *Gardenia jasminoides* 25g  *Isatis tinctoria* 200g  *Lonicera japonica 60g* | Each 1 mL of the product contains 1.50 to 3.50 mg of *Acidum cholicum1* (C₂₄H₄₀O₅), not less than 0.20 mg of geniposide (C₁₇H₂₄O₁₀) calculated from 6*Fructus Gardeniae*, not less than 3.5 mg of baicalin (C₂₁H₁₈O₁₁), and 2.2 to 3.0 mg of total nitrogen (N). | *Isatis tinctoria*, *Gardenia jasminoides* and *Lonicera japonica* were decocted and purified by alcohol precipitation. *Bubalus bubalis* and *Pteria martensii* were hydrolyzed and alcohol-precipitated. The extracts were mixed with *Acidum cholicum* and *Chenodeoxycholic Acid* ethanol solution, pH-adjusted, combined with *Scutellaria baicalensis* solution, adjusted to 1000 mL, carbon-treated, filled and sterilized. |
| Xiyanping Injection (XYP) | Jiangxi Qingfeng Pharmaceutical Co., Ltd | China Drug Approval Number Z20026249 | 2 mL (50 mg) per vial | *Andrographis paniculata* | Each 1 mL contains andrographolide (C₂₀H₃₀O₅) with a linear range of 24.84–248.4 μg/mL | Andrographolide was dissolved in absolute ethanol, sulfonated with sulfuric acid at 0°C for 48 h, quenched with ethanol, neutralized to pH 7.3 with sodium hydroxide, and purified by 85% ethanol precipitation. The sulfonated product was decolorized with activated carbon, concentrated, redissolved, treated twice with activated carbon, adjusted to pH 7.3–7.5, sterile-filtered, filled, and sterilized at 105°C for 30 min. |

**Appendix 4:** **Risk of bias of randomized clinical trials**

Figure S4: Overall risk of bias presented as percentage of each risk of bias item across all included studies.

Green = Low risk, Red = High risk, Yellow = Some concerns.

Table S4: Study level risk of bias assessment using Cochrane risk of bias tool 2.0 for assessing risk of bias of randomized clinical trials.

| Study | Randomization process | Deviations from intended interventions | Missing outcome data | Measurement of the outcome | Selection of the reported result | Overall |
| --- | --- | --- | --- | --- | --- | --- |
| Yang2021 | Low | Some concerns | Low | Low | Low | Some concerns |
| 1He2020 | Low | Some concerns | Low | Low | Low | Some concerns |
| Chen2021 | Low | Some concerns | Low | Low | Low | Some concerns |
| Jiang2022 | Low | Some concerns | Low | Low | Low | Some concerns |
| Tian2021 | Low | Some concerns | Low | Low | Low | Some concerns |
| Yin2022 | Low | Some concerns | Low | Low | Low | Some concerns |
| Tang2022 | Low | Some concerns | Low | Low | Low | Some concerns |
| Wang2016 | Low | Some concerns | Low | Low | Low | Some concerns |
| Zhang2015 | Low | Some concerns | Low | Low | Low | Some concerns |
| Li2025 | Low | Some concerns | Low | Low | Low | Some concerns |
| Yu2024 | Low | Some concerns | Low | Low | Low | Some concerns |
| Chen2020 | Low | Some concerns | Low | Low | Low | Some concerns |
| Zhang2019 | High | Some concerns | Low | Low | Low | High |
| Wang2015 | Low | Some concerns | Low | Low | Low | Some concerns |
| Wang2020 | Low | Some concerns | Low | Low | Low | Some concerns |
| He2021 | Low | Some concerns | Low | Low | Low | Some concerns |
| Luo2020 | Low | Some concerns | Low | Low | Low | Some concerns |
| Wang2017 | Low | Some concerns | Low | Low | Low | Some concerns |
| Pei2019 | High | Some concerns | Low | Low | Low | High |
| Zheng2016 | Low | Some concerns | Low | Low | Low | Some concerns |
| Li2024 | Low | Some concerns | Low | Low | Low | Some concerns |
| Wang2021 | Low | Some concerns | Low | Low | Low | Some concerns |
| Hou2019 | Low | Some concerns | Some concerns | Low | Low | Some concerns |
| Jiang2019 | Low | Some concerns | Some concerns | Low | Low | Some concerns |
| Jiang2021 | Low | Some concerns | Some concerns | Low | Low | Some concerns |
| Liu2019 | Low | Some concerns | Some concerns | Low | Low | Some concerns |
| Wei2020 | Low | Some concerns | Low | Low | Low | Some concerns |
| Ji2017 | Low | Some concerns | Low | Low | Low | Some concerns |
| Qu2023 | Low | Some concerns | Low | Low | Low | Some concerns |
| Wu2014 | Low | Some concerns | Low | Low | Low | Some concerns |
| Cai2023 | Low | Some concerns | Low | Low | Low | Some concerns |
| Yu2024 | Low | Some concerns | Low | Low | Low | Some concerns |
| Liu2024 | Low | Some concerns | Low | Low | Low | Some concerns |
| Li2009 | Low | Some concerns | Low | Low | Low | Some concerns |
| Liu2012 | Low | Some concerns | Low | Low | Low | Some concerns |
| Yu2019 | Low | Some concerns | Low | Low | Low | Some concerns |
| Xiang2022 | Low | Some concerns | Low | Low | Low | Some concerns |
| Zhang2024 | Low | Some concerns | Low | Low | Low | Some concerns |
| Zhang2006 | Low | Some concerns | Low | Low | Low | Some concerns |
| Hong2008 | Low | Some concerns | Low | Low | Low | Some concerns |
| Zhang2010 | Low | Some concerns | Low | Low | Low | Some concerns |
| Yao2020 | Low | Some concerns | Low | Low | Low | Some concerns |
| Rao2012 | Low | Some concerns | Low | Low | Low | Some concerns |
| Sun2012 | Low | Some concerns | Low | Low | Low | Some concerns |
| Ma2020 | Low | Some concerns | Low | Low | Low | Some concerns |
| Yu2022 | Low | Some concerns | Low | Low | Low | Some concerns |
| Shao2023 | Low | Some concerns | Low | Low | Low | Some concerns |
| Tang2021 | Low | Some concerns | Low | Low | Low | Some concerns |
| Pang2015 | Low | Some concerns | Low | Low | Low | Some concerns |
| Zeng2014 | Low | Some concerns | Some concerns | Low | Low | Some concerns |
| Chen2014 | Low | Some concerns | Low | Low | Low | Some concerns |
| Lu2018 | Low | Some concerns | Low | Low | Low | Some concerns |
| Wang2013 | High | Some concerns | Low | Low | Low | High |
| Wei2014 | High | Some concerns | Low | Low | Low | High |
| Zhou2014 | Low | Some concerns | Low | Low | Low | Some concerns |
| Peng2021 | Low | Some concerns | Low | Low | Low | Some concerns |
| Sun2022 | Low | Some concerns | Low | Low | Low | Some concerns |
| Fu2021 | Low | Some concerns | Low | Low | Low | Some concerns |
| Liu2020 | Low | Some concerns | Low | Low | Low | Some concerns |
| Peng2019 | Low | Some concerns | Low | Low | Low | Some concerns |
| Zhang2015 | Low | Some concerns | Low | Low | Low | Some concerns |
| Lei2019 | Low | Some concerns | Low | Low | Low | Some concerns |
| Re2022 | Low | Some concerns | Low | Low | Low | Some concerns |
| Zhang2017 | Low | Some concerns | Low | Low | Low | Some concerns |
| Zhang2025 | Low | Some concerns | Low | Low | Low | Some concerns |
| Zhao2024 | Low | Some concerns | Low | Low | Low | Some concerns |
| Zeng2009 | Low | Some concerns | Low | Low | Low | Some concerns |
| Sun2001 | Low | Some concerns | Low | Low | Low | Some concerns |
| Ding2013 | Low | Some concerns | Low | Low | Low | Some concerns |
| Li2016 | High | Some concerns | Low | Low | Low | High |
| Cao2012 | Low | Some concerns | Low | Low | Low | Some concerns |
| Chang2006 | Low | Some concerns | Low | Low | Low | Some concerns |
| Yang2009 | Low | Some concerns | Low | Low | Low | Some concerns |
| He2008 | Low | Some concerns | Low | Low | Low | Some concerns |
| Xie2022 | High | Some concerns | Low | Low | Low | High |
| Zeng2018 | Low | Some concerns | Low | Low | Low | Some concerns |
| Liang2012 | Low | Some concerns | Low | Low | Low | Some concerns |
| Chang2015 | High | Some concerns | Low | Low | Low | High |
| Zhang2014 | Low | Some concerns | Low | Low | Low | Some concerns |
| Han2012 | Low | Some concerns | High | Low | High | High |
| Zhang2011 | Low | Some concerns | Low | Low | Low | Some concerns |
| Liu2016 | High | Some concerns | Low | Low | Low | High |
| Zhang2017 | Low | Some concerns | Low | Low | Low | Some concerns |
| Han2015 | Low | Some concerns | Low | Low | Low | Some concerns |

**Appendix 5: Evaluation of Inconsistency and Heterogeneity**

| **Outcomes** | **Study** | **Global inconsistency** | | **Heterogeneity** |
| --- | --- | --- | --- | --- |
|  |  | **Chi square** | **P value** | ***τ²*** |
| **Total effective rate** | 73 RCT, n= 7339 | 19.72 | 0.06 | 0.00 |
| **FVC** | 25 RCT, n= 2805 | 10.92 | 0.16 | 0.01 |
| **FEV1** | 39 RCT, n= 4090 | 15.43 | 0.28 | 0.02 |
| **FEV1/FVC** | 52 RCT, n= 5904 | 2.03 | 0.15 | 0.97 |
| **IL-6** | 18 RCT, n= 2050 | 0.19 | 0.66 | 0.32 |
| **IL-8** | 15 RCT, n= 1266 | 9.93 | 0.07 | 0.41 |
| **TNF-α** | 19 RCT, n= 1863 | 8.43 | 0.10 | 0.36 |
| **PH** | 10 RCT, n= 1216 | 4.99 | 0.03 | 0.00 |
| **PaO2** | 34 RCT, n= 3613 | 1.98 | 0.16 | 0.64 |
| **PaCO2** | 34 RCT, n= 3613 | 1.68 | 0.19 | 0.83 |
| **Adverse event** | 44 RCT, n= 4776 | 0.93 | 0.34 | 0.00 |

**Appendix 6: CINeMA Assessment**

We use the CINeMA framework to evidence certainty, assessing it for each network estimate based on the following criteria:

**A: Within study bias:** We classified the overall risk of bias for each study as low risk of bias, the risk of bias as moderate when none of the four assessed risk of bias items were rated as high risk, and the risk of bias as high when one or both items were rated as high risk. See Appendix 4 for the bias assessment. The

risk of bias for a pairwise comparison of each drug is shown in **Figure S6.1-6.20.**

Figure S6.1:Risk of bias contribution by intervention group in **total effective rate**


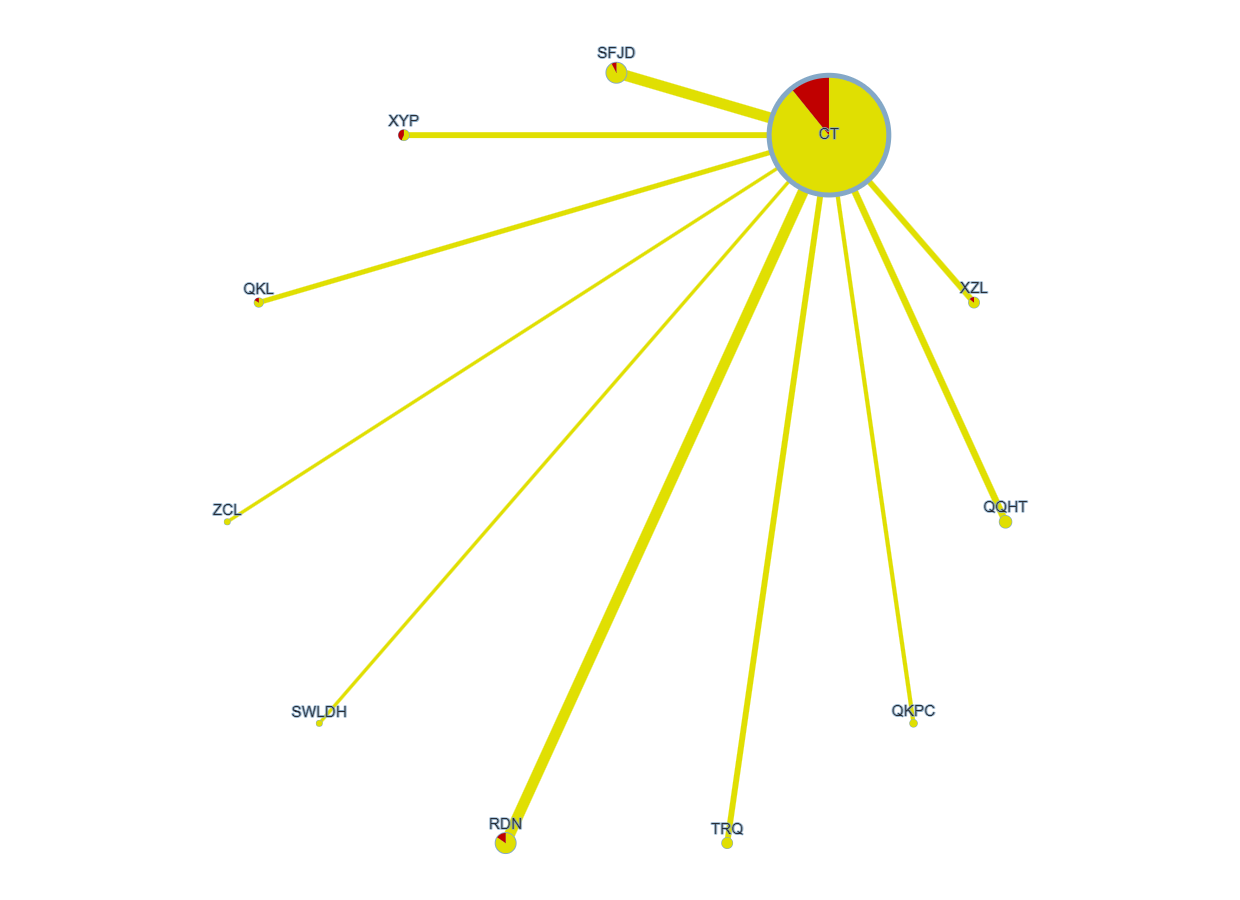


NOTE: **SFJD**: Shufeng Jiedu Capsule; **XZL**: Fresh Bamboo Juice Oral Liquid; **QQHT**: Qingqi Huatan Pill; **QKPC**: Qingke Pingchuan Granule; **TRQ**: Tanreqing Injection; **RDN**: Reduning Injection; **SWLDH**: Shiwei Longdanhua Capsule;

**ZCL**: Zhichuanling Injection; **QKL**: Qingkailing Injection; **XYP**: Xiyanping Injection; **CT**: Conventional biomedicine treatment.

Figure S6.2:Overall risk of bias by treatment comparison in **total effective rate**


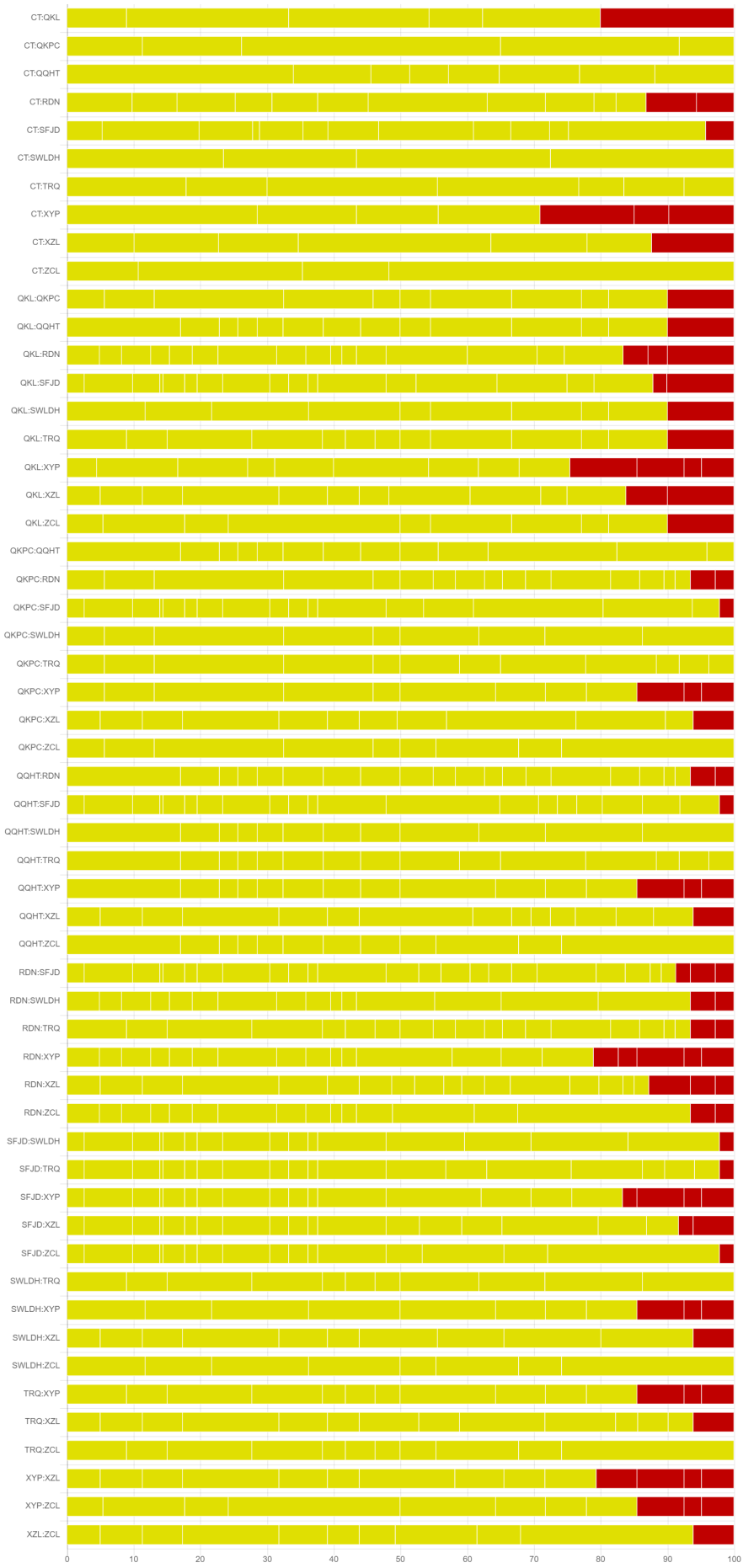


NOTE: **SFJD**: Shufeng Jiedu Capsule; **XZL**: Fresh Bamboo Juice Oral Liquid; **QQHT**: Qingqi Huatan Pill; **QKPC**: Qingke Pingchuan Granule; **TRQ**: Tanreqing Injection; **RDN**: Reduning Injection; **SWLDH**: Shiwei Longdanhua Capsule;

**ZCL**: Zhichuanling Injection; **QKL**: Qingkailing Injection; **XYP**: Xiyanping Injection; **CT**: Conventional biomedicine treatment.

Figure S6.3:Risk of bias contribution by intervention group in **FVC**


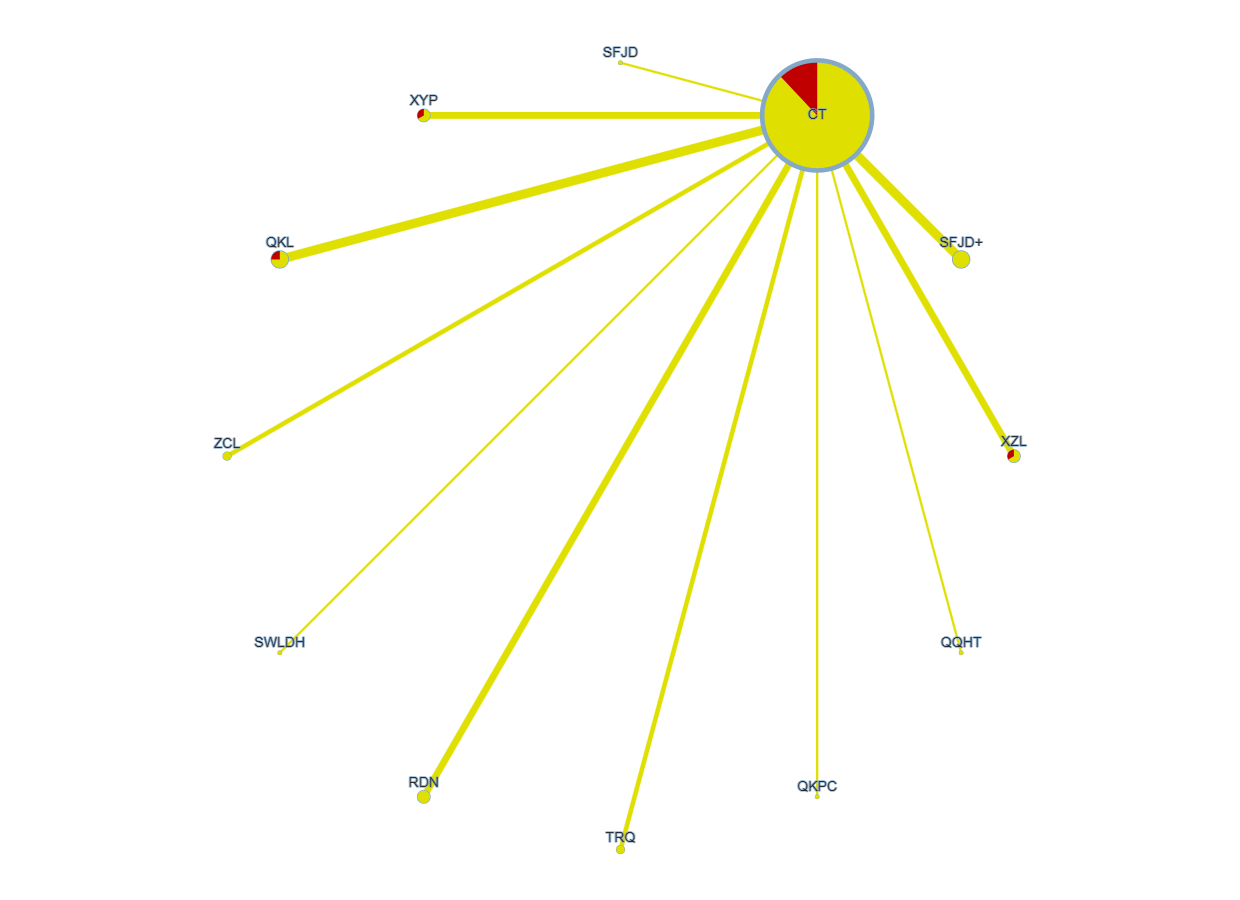


NOTE: **SFJD**: Shufeng Jiedu Capsule; **XZL**: Fresh Bamboo Juice Oral Liquid; **QQHT**: Qingqi Huatan Pill; **QKPC**: Qingke Pingchuan Granule; **TRQ**: Tanreqing Injection; **RDN**: Reduning Injection; **SWLDH**: Shiwei Longdanhua Capsule;

**ZCL**: Zhichuanling Injection; **QKL**: Qingkailing Injection; **XYP**: Xiyanping Injection; **CT**: Conventional biomedicine treatment.

Figure S6.4:Overall risk of bias by treatment comparison in **FVC**


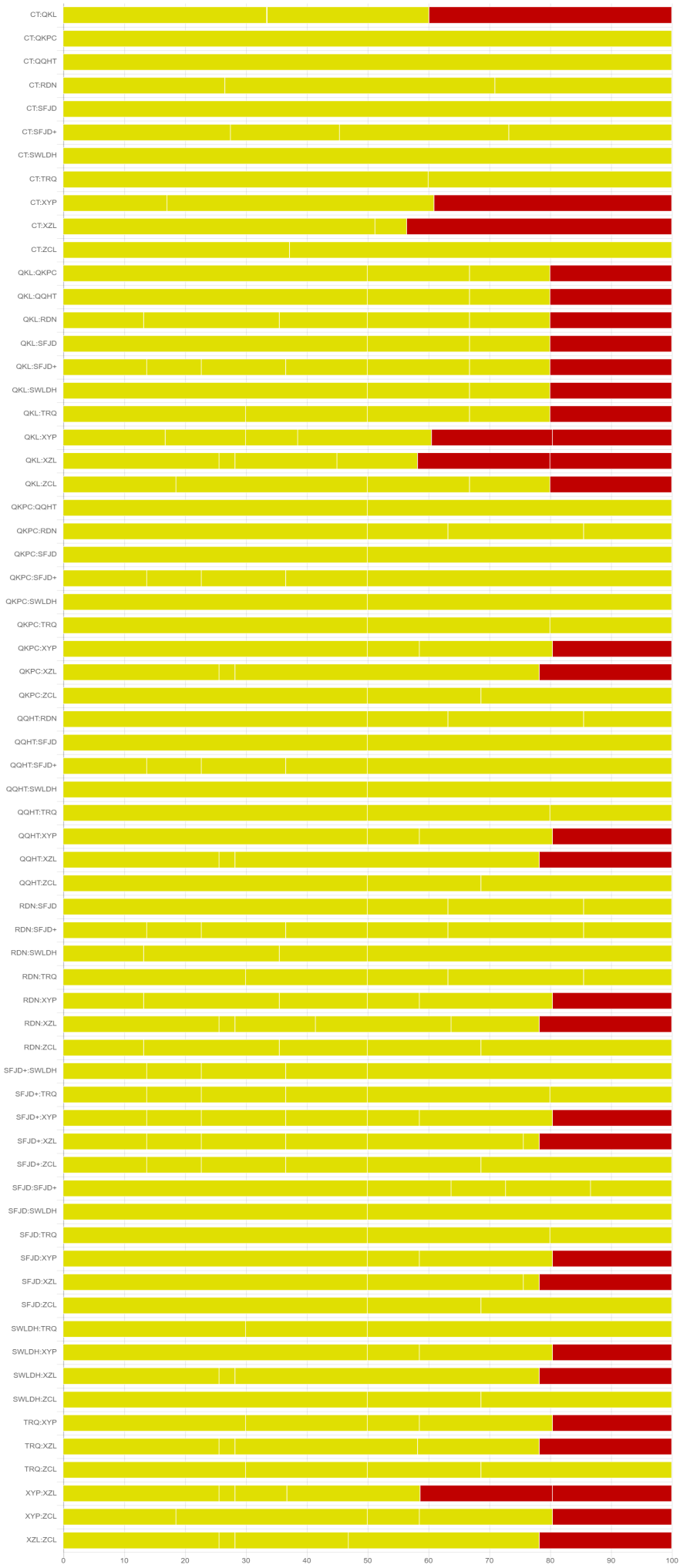


NOTE: **SFJD**: Shufeng Jiedu Capsule; **XZL**: Fresh Bamboo Juice Oral Liquid; **QQHT**: Qingqi Huatan Pill; **QKPC**: Qingke Pingchuan Granule; **TRQ**: Tanreqing Injection; **RDN**: Reduning Injection; **SWLDH**: Shiwei Longdanhua Capsule;

**ZCL**: Zhichuanling Injection; **QKL**: Qingkailing Injection; **XYP**: Xiyanping Injection; **CT**: Conventional biomedicine treatment.

Figure S6.5:Risk of bias contribution by intervention group in **FEV1**


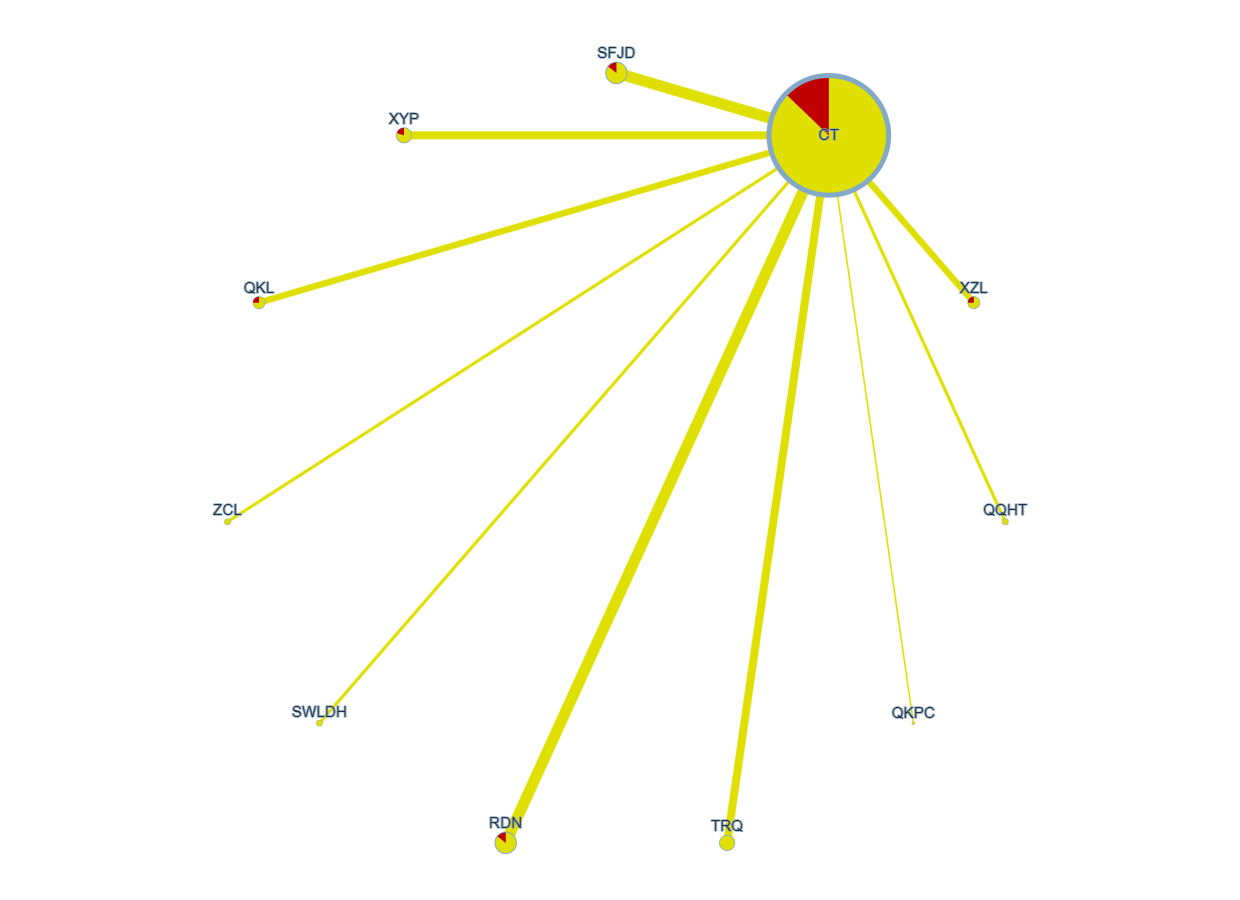


NOTE: **SFJD**: Shufeng Jiedu Capsule; **XZL**: Fresh Bamboo Juice Oral Liquid; **QQHT**: Qingqi Huatan Pill; **QKPC**: Qingke Pingchuan Granule; **TRQ**: Tanreqing Injection; **RDN**: Reduning Injection; **SWLDH**: Shiwei Longdanhua Capsule;

**ZCL**: Zhichuanling Injection; **QKL**: Qingkailing Injection; **XYP**: Xiyanping Injection; **CT**: Conventional biomedicine treatment.

Figure S6.6:Overall risk of bias by treatment comparison in **FEV1**


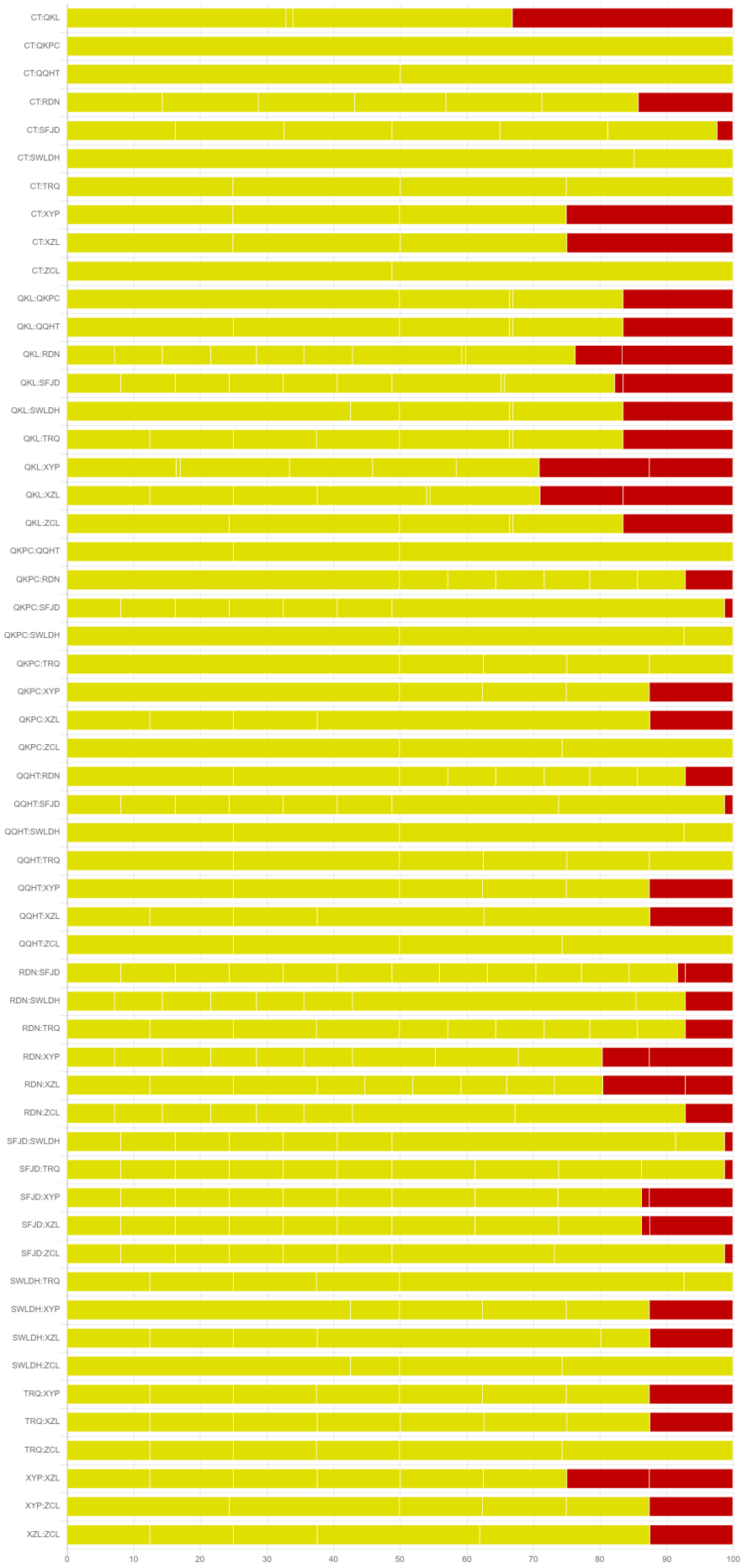


NOTE: **SFJD**: Shufeng Jiedu Capsule; **XZL**: Fresh Bamboo Juice Oral Liquid; **QQHT**: Qingqi Huatan Pill; **QKPC**: Qingke Pingchuan Granule; **TRQ**: Tanreqing Injection; **RDN**: Reduning Injection; **SWLDH**: Shiwei Longdanhua Capsule;

**ZCL**: Zhichuanling Injection; **QKL**: Qingkailing Injection; **XYP**: Xiyanping Injection; **CT**: Conventional biomedicine treatment.

Figure S6.7:Risk of bias contribution by intervention group in **FEV1/FVC**


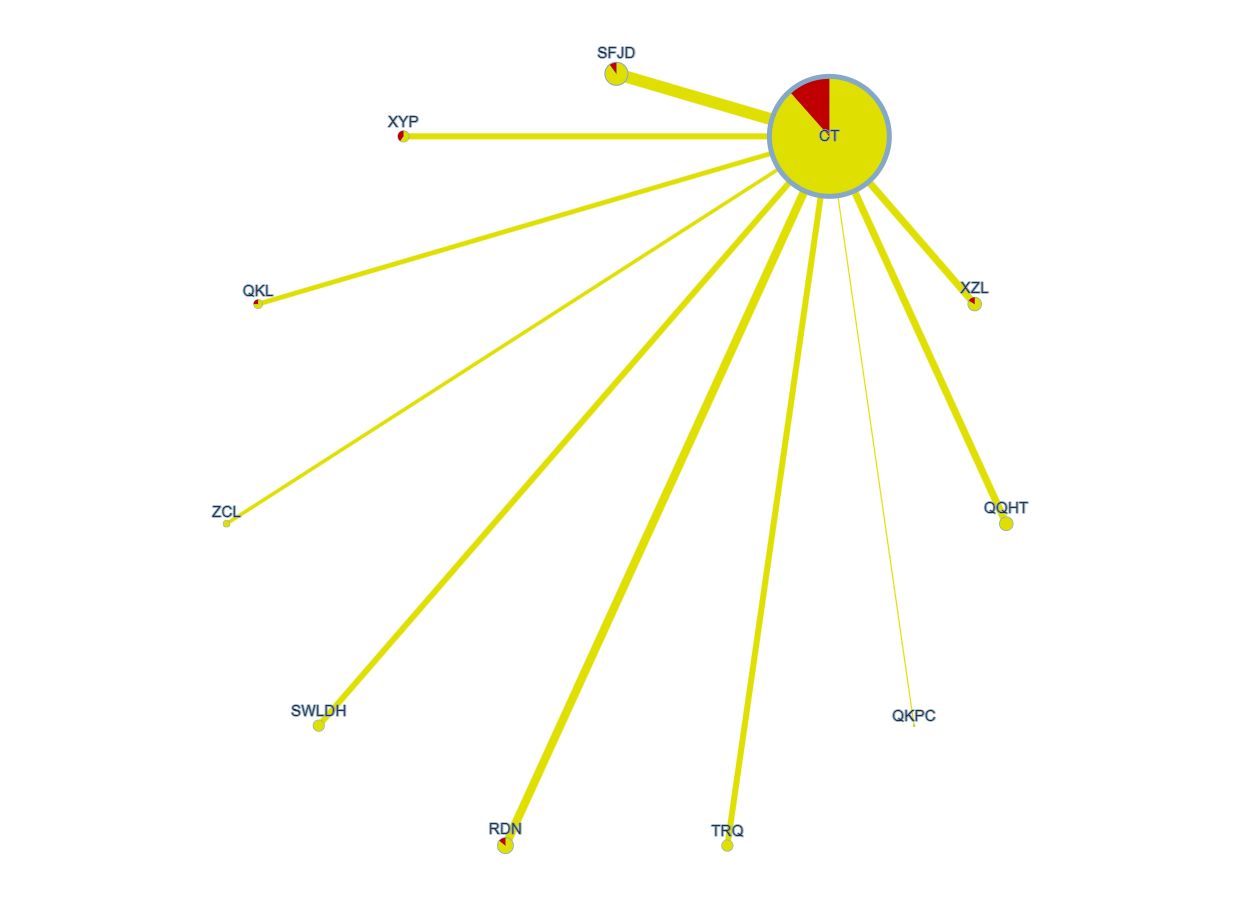


NOTE: **SFJD**: Shufeng Jiedu Capsule; **XZL**: Fresh Bamboo Juice Oral Liquid; **QQHT**: Qingqi Huatan Pill; **QKPC**: Qingke Pingchuan Granule; **TRQ**: Tanreqing Injection; **RDN**: Reduning Injection; **SWLDH**: Shiwei Longdanhua Capsule;

**ZCL**: Zhichuanling Injection; **QKL**: Qingkailing Injection; **XYP**: Xiyanping Injection; **CT**: Conventional biomedicine treatment.

Figure S6.8:Overall risk of bias by treatment comparison in **FEV1/FVC**


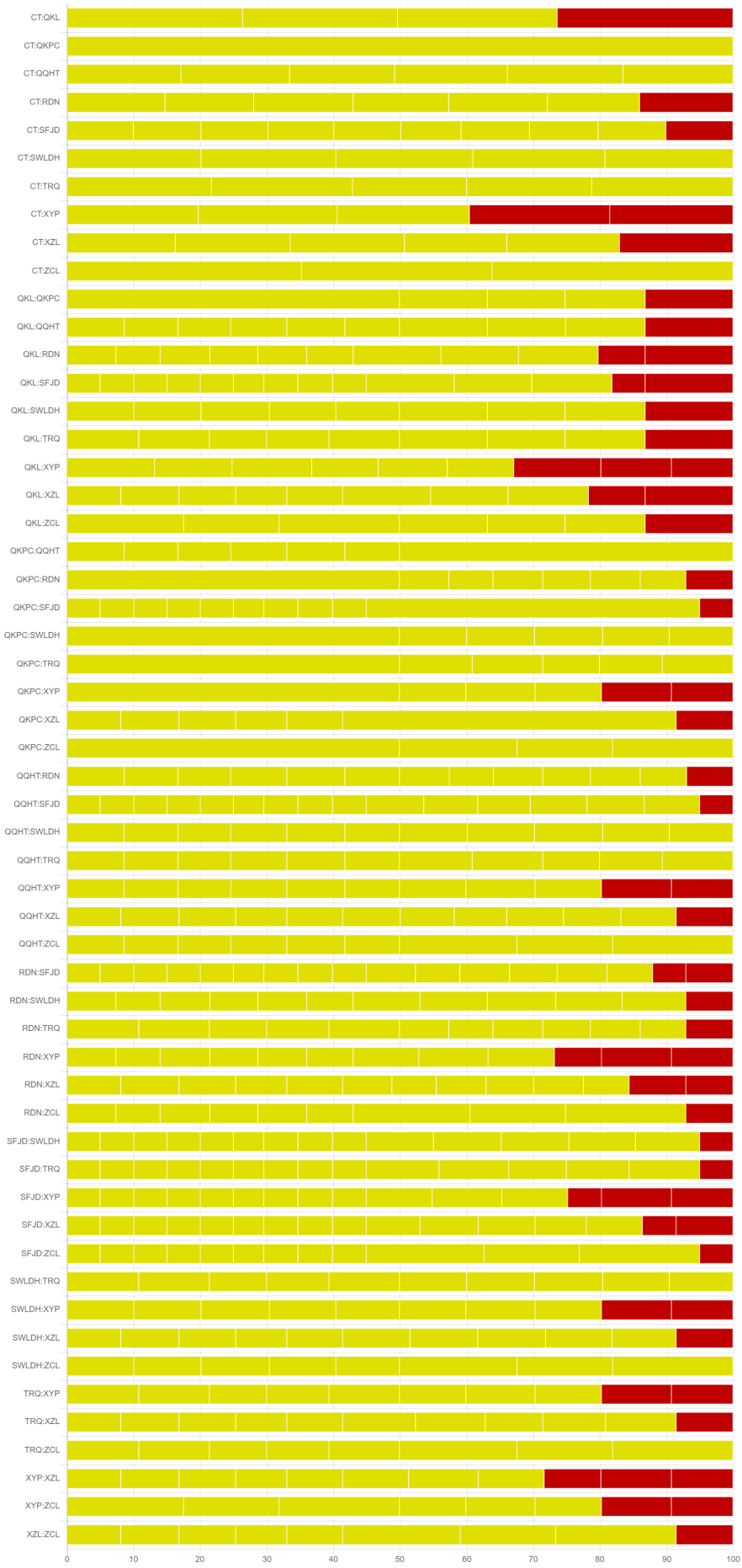


NOTE: **SFJD**: Shufeng Jiedu Capsule; **XZL**: Fresh Bamboo Juice Oral Liquid; **QQHT**: Qingqi Huatan Pill; **QKPC**: Qingke Pingchuan Granule; **TRQ**: Tanreqing Injection; **RDN**: Reduning Injection; **SWLDH**: Shiwei Longdanhua Capsule;

**ZCL**: Zhichuanling Injection; **QKL**: Qingkailing Injection; **XYP**: Xiyanping Injection; **CT**: Conventional biomedicine treatment.

Figure S6.9:Risk of bias contribution by intervention group in **PH**


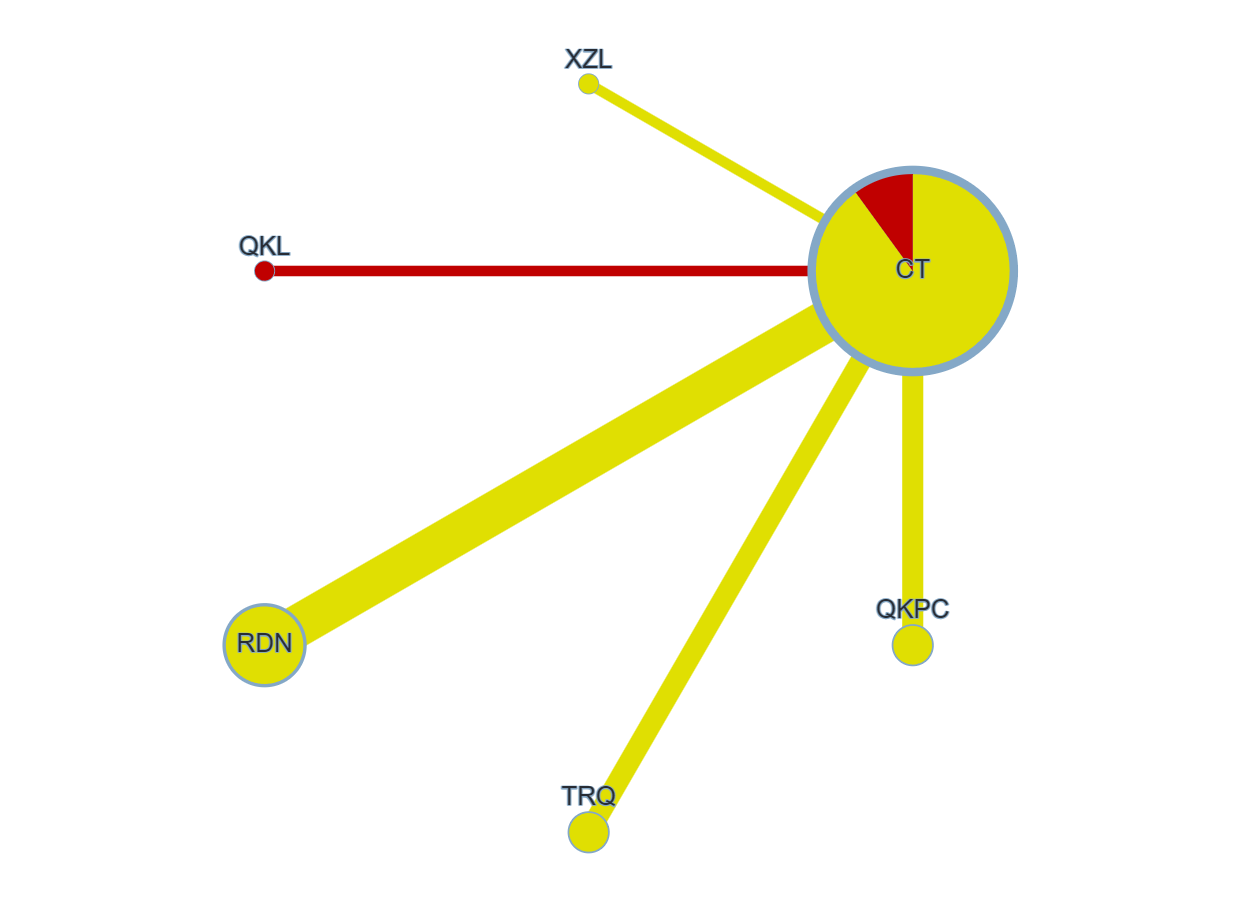


NOTE: **XZL**: Fresh Bamboo Juice Oral Liquid; **QKL**: Qingkailing Injection; **RDN**: Reduning Injection; **TRQ**: Tanreqing Injection; **QKPC**: Qingke Pingchuan Granule; **CT**: Conventional biomedicine treatment.

Figure S6.10:Overall risk of bias by treatment comparison in **PH**


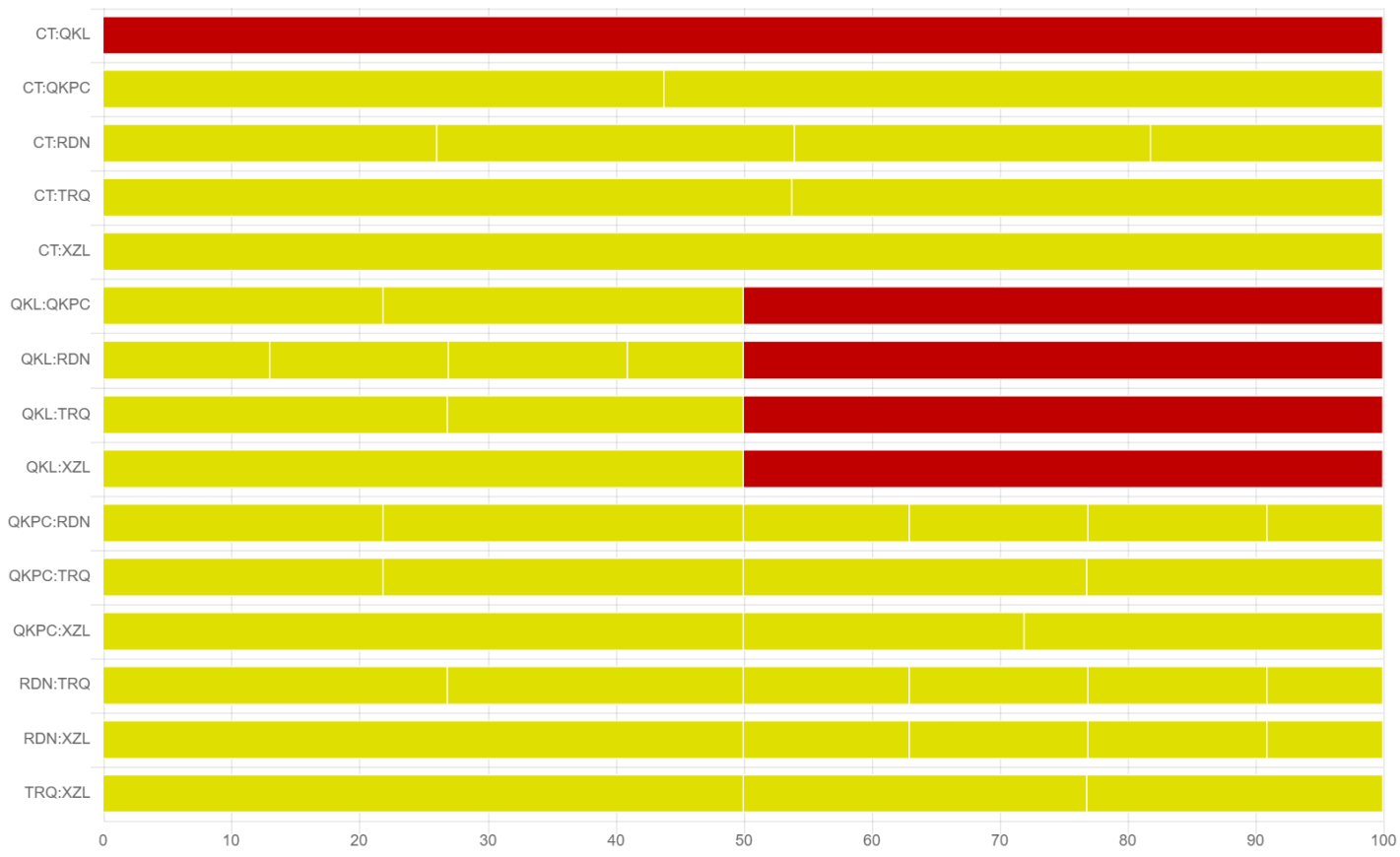


NOTE: **XZL**: Fresh Bamboo Juice Oral Liquid; **QKL**: Qingkailing Injection; **RDN**: Reduning Injection; **TRQ**: Tanreqing Injection; **QKPC**: Qingke Pingchuan Granule; **CT**: Conventional biomedicine treatment.

Figure S6.11:Risk of bias contribution by intervention group in **PaO2**


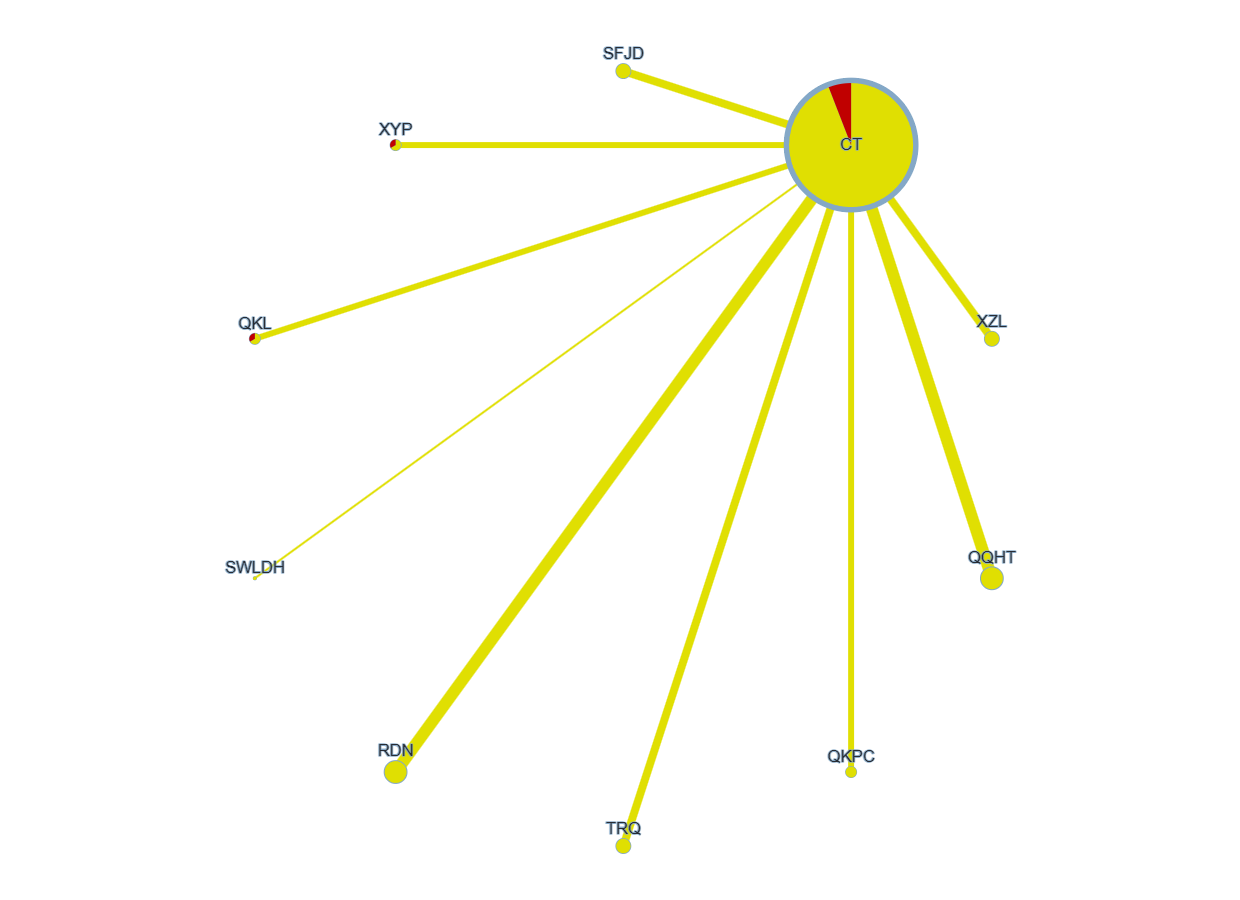


NOTE: **SFJD**: Shufeng Jiedu Capsule; **XZL**: Fresh Bamboo Juice Oral Liquid; **QQHT**: Qingqi Huatan Pill; **QKPC**: Qingke Pingchuan Granule; **TRQ**: Tanreqing Injection; **RDN**: Reduning Injection; **SWLDH**: Shiwei Longdanhua Capsule;

**QKL**: Qingkailing Injection; **XYP**: Xiyanping Injection; **CT**: Conventional biomedicine treatment.

Figure S6.12:Overall risk of bias by treatment comparison in **PaO2**


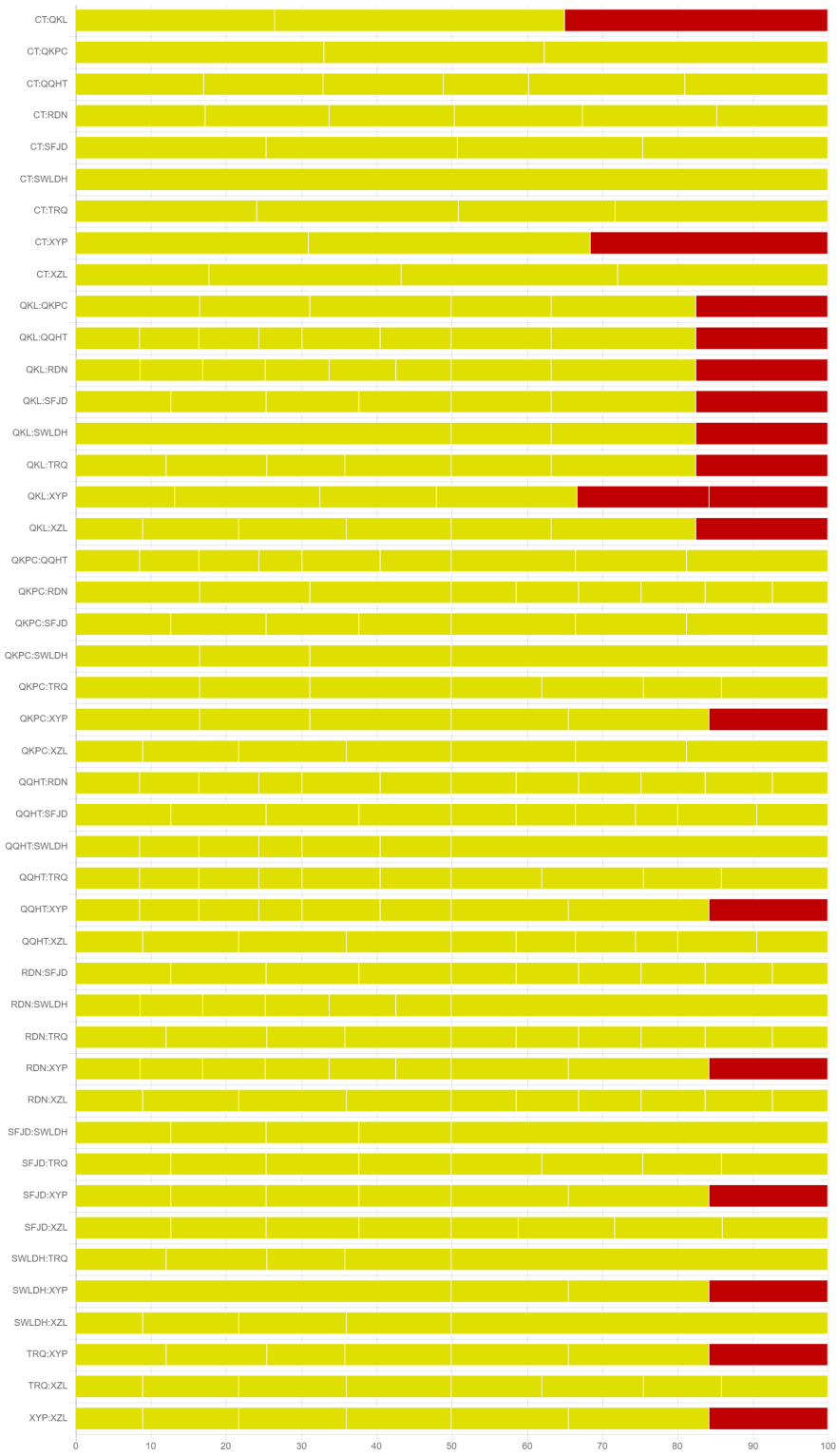


NOTE: **SFJD**: Shufeng Jiedu Capsule; **XZL**: Fresh Bamboo Juice Oral Liquid; **QQHT**: Qingqi Huatan Pill; **QKPC**: Qingke Pingchuan Granule; **TRQ**: Tanreqing Injection; **RDN**: Reduning Injection; **SWLDH**: Shiwei Longdanhua Capsule;

**QKL**: Qingkailing Injection; **XYP**: Xiyanping Injection; **CT**: Conventional biomedicine treatment.

Figure S6.13:Risk of bias contribution by intervention group in **PaCO2**


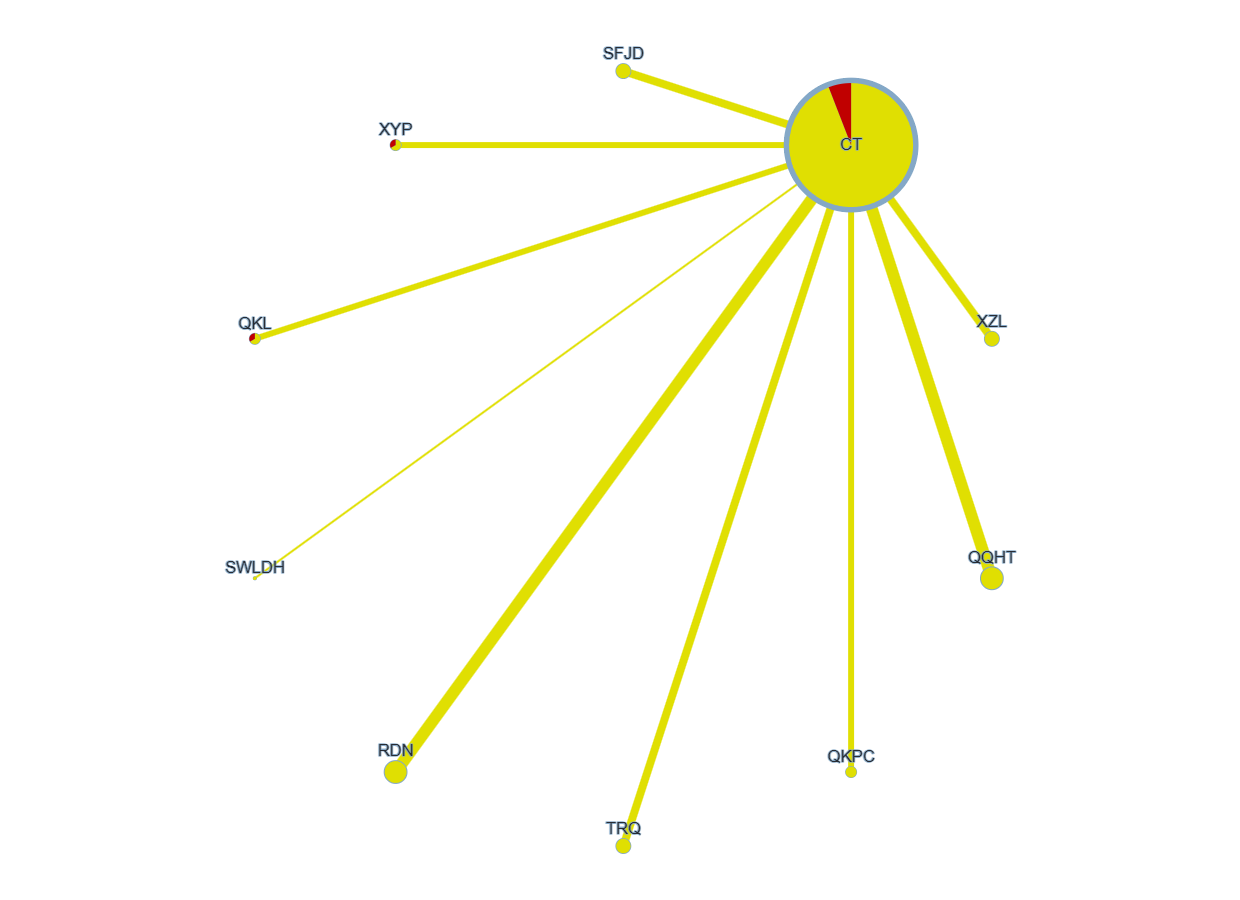


NOTE: **SFJD**: Shufeng Jiedu Capsule; **XZL**: Fresh Bamboo Juice Oral Liquid; **QQHT**: Qingqi Huatan Pill; **QKPC**: Qingke Pingchuan Granule; **TRQ**: Tanreqing Injection; **RDN**: Reduning Injection; **SWLDH**: Shiwei Longdanhua Capsule;

**QKL**: Qingkailing Injection; **XYP**: Xiyanping Injection; **CT**: Conventional biomedicine treatment.

Figure S6.14:Overall risk of bias by treatment comparison in **PaCO2**


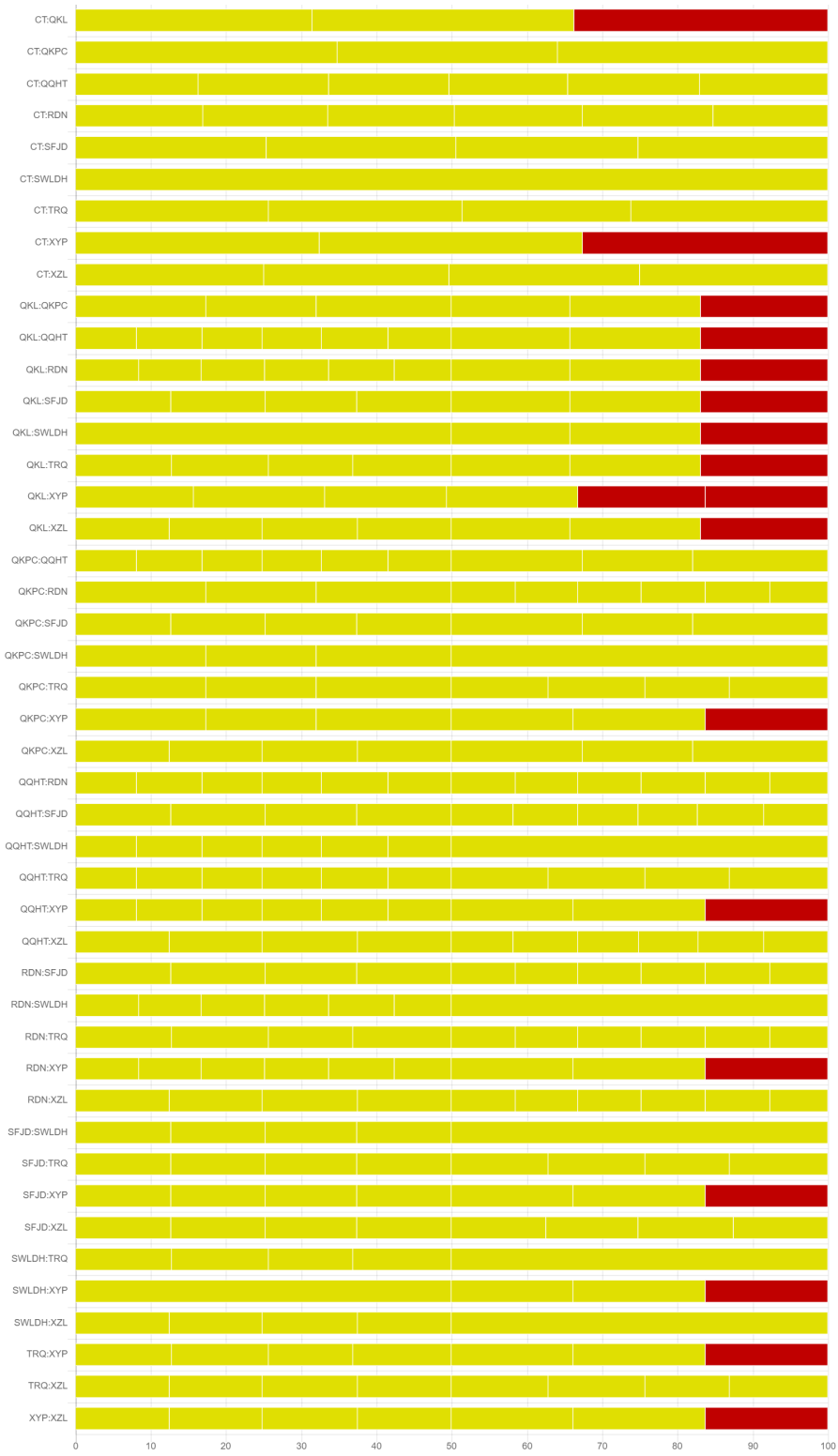


NOTE: **SFJD**: Shufeng Jiedu Capsule; **XZL**: Fresh Bamboo Juice Oral Liquid; **QQHT**: Qingqi Huatan Pill; **QKPC**: Qingke Pingchuan Granule; **TRQ**: Tanreqing Injection; **RDN**: Reduning Injection; **SWLDH**: Shiwei Longdanhua Capsule;

**QKL**: Qingkailing Injection; **XYP**: Xiyanping Injection; **CT**: Conventional biomedicine treatment.

Figure S6.15:Risk of bias contribution by intervention group in **IL-6**


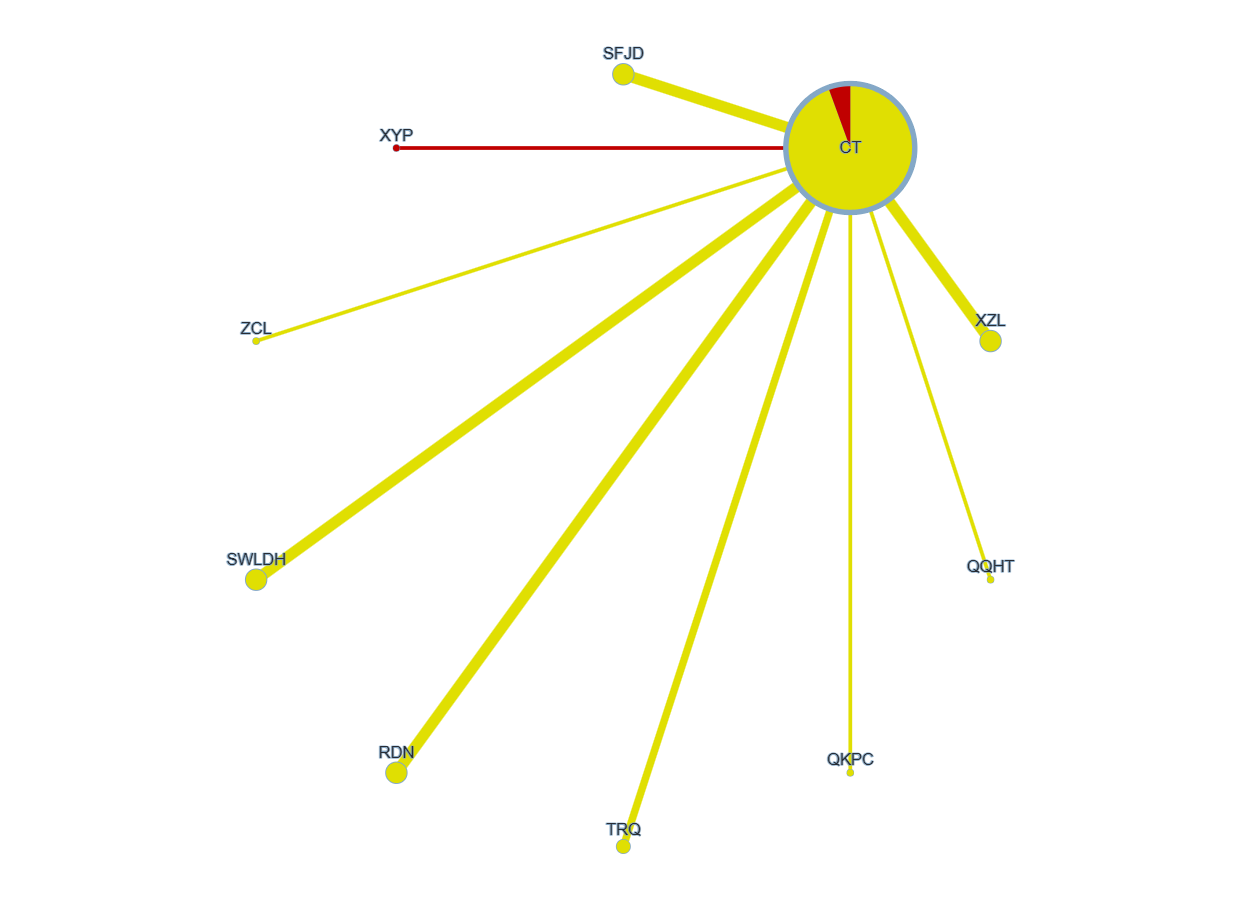


NOTE: **SFJD**: Shufeng Jiedu Capsule; **XZL**: Fresh Bamboo Juice Oral Liquid; **QQHT**: Qingqi Huatan Pill; **QKPC**: Qingke Pingchuan Granule; **TRQ**: Tanreqing Injection; **RDN**: Reduning Injection; **SWLDH**: Shiwei Longdanhua Capsule;

**ZCL**: Zhichuanling Injection; **XYP**: Xiyanping Injection; **CT**: Conventional biomedicine treatment.

Figure S6.16:Overall risk of bias by treatment comparison in **IL-6**

**
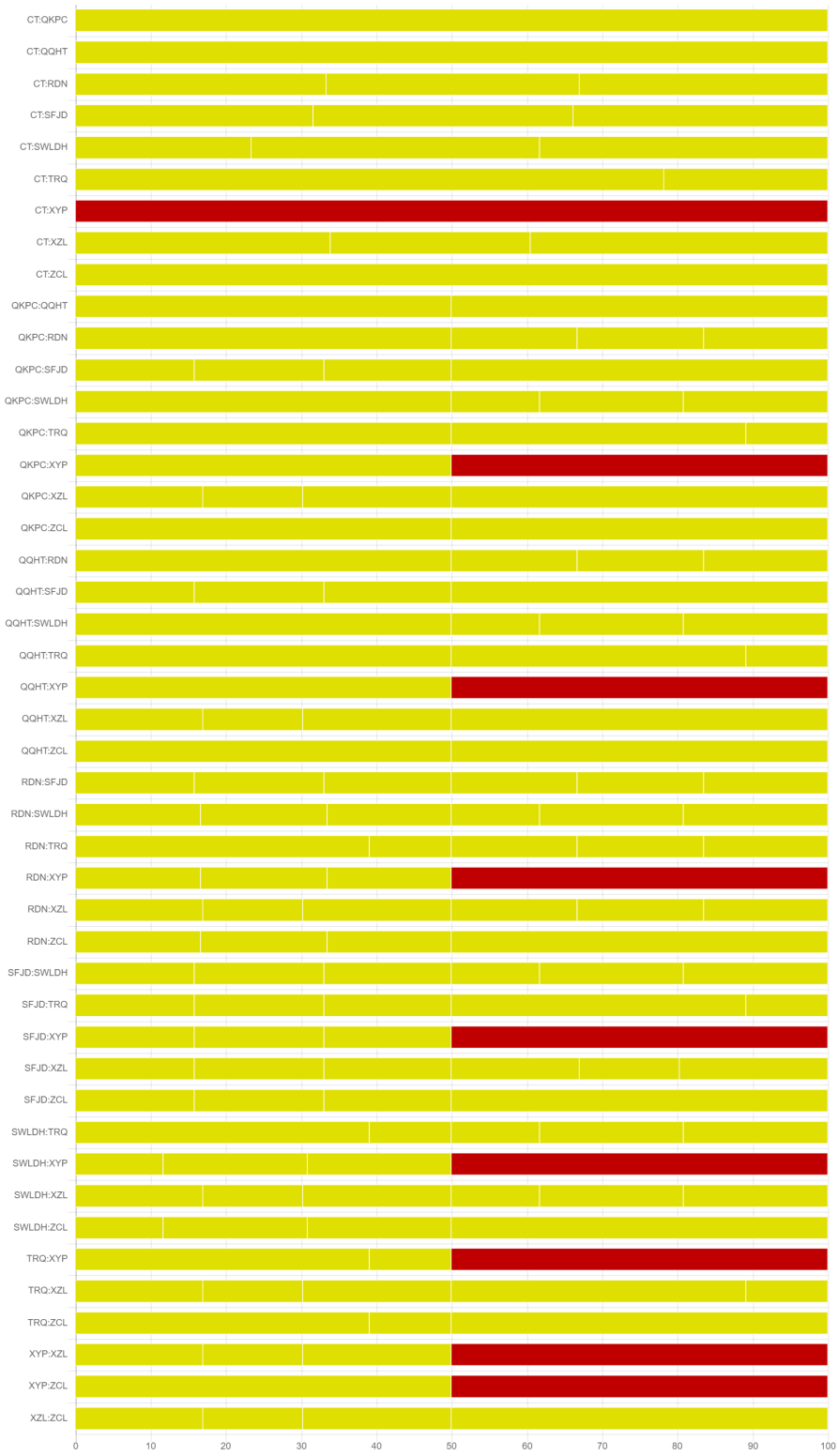
**

NOTE: **SFJD**: Shufeng Jiedu Capsule; **XZL**: Fresh Bamboo Juice Oral Liquid; **QQHT**: Qingqi Huatan Pill; **QKPC**: Qingke Pingchuan Granule; **TRQ**: Tanreqing Injection; **RDN**: Reduning Injection; **SWLDH**: Shiwei Longdanhua Capsule;

**ZCL**: Zhichuanling Injection; **XYP**: Xiyanping Injection; **CT**: Conventional biomedicine treatment.

Figure S6.17:Risk of bias contribution by intervention group in **IL-8**

**
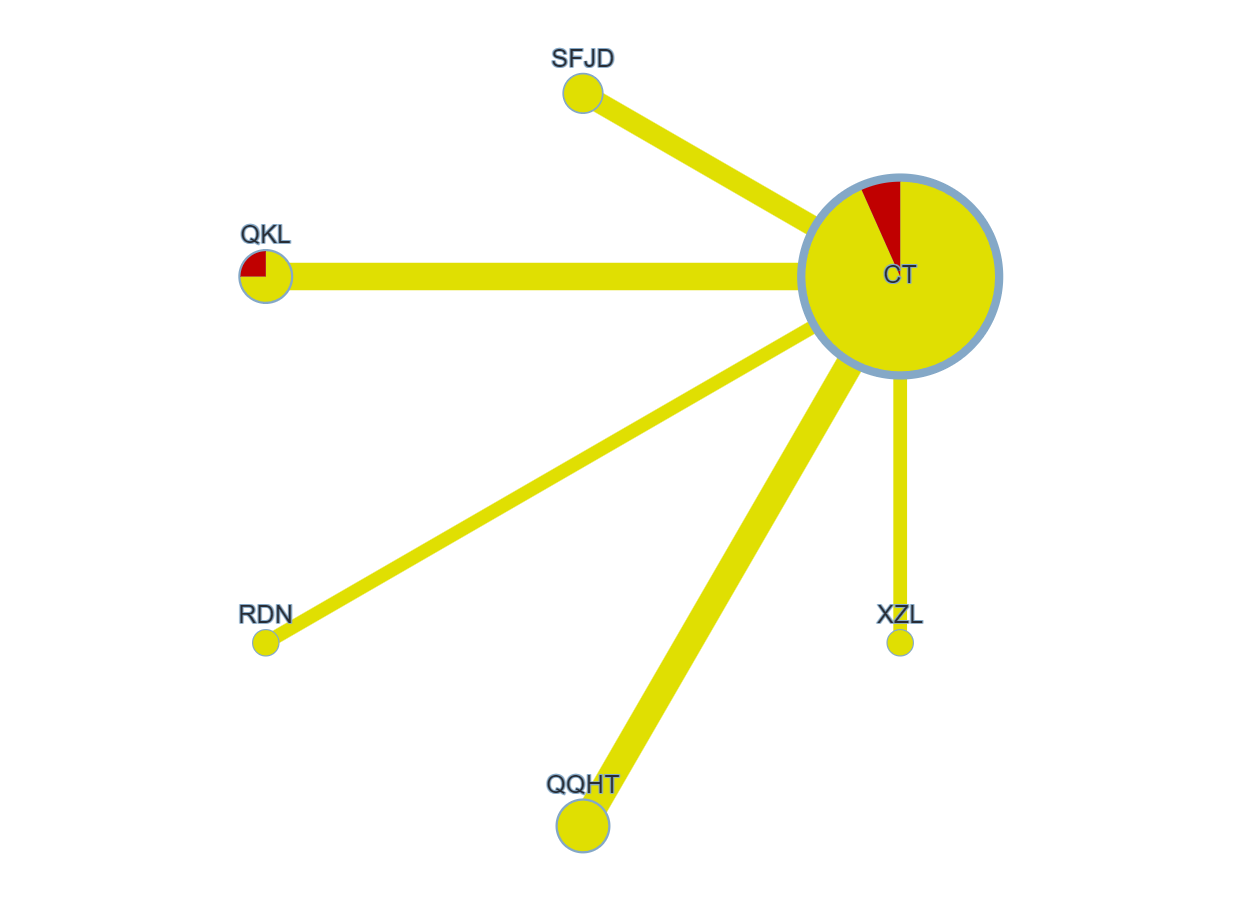
**

NOTE: **SFJD**: Shufeng Jiedu Capsule; **XZL**: Fresh Bamboo Juice Oral Liquid; **QQHT**: Qingqi Huatan Pill; **RDN**: Reduning Injection; **QKL**: Qingkailing Injection; **CT**: Conventional biomedicine treatment.

Figure S6.18:Overall risk of bias by treatment comparison in **IL-8**

**
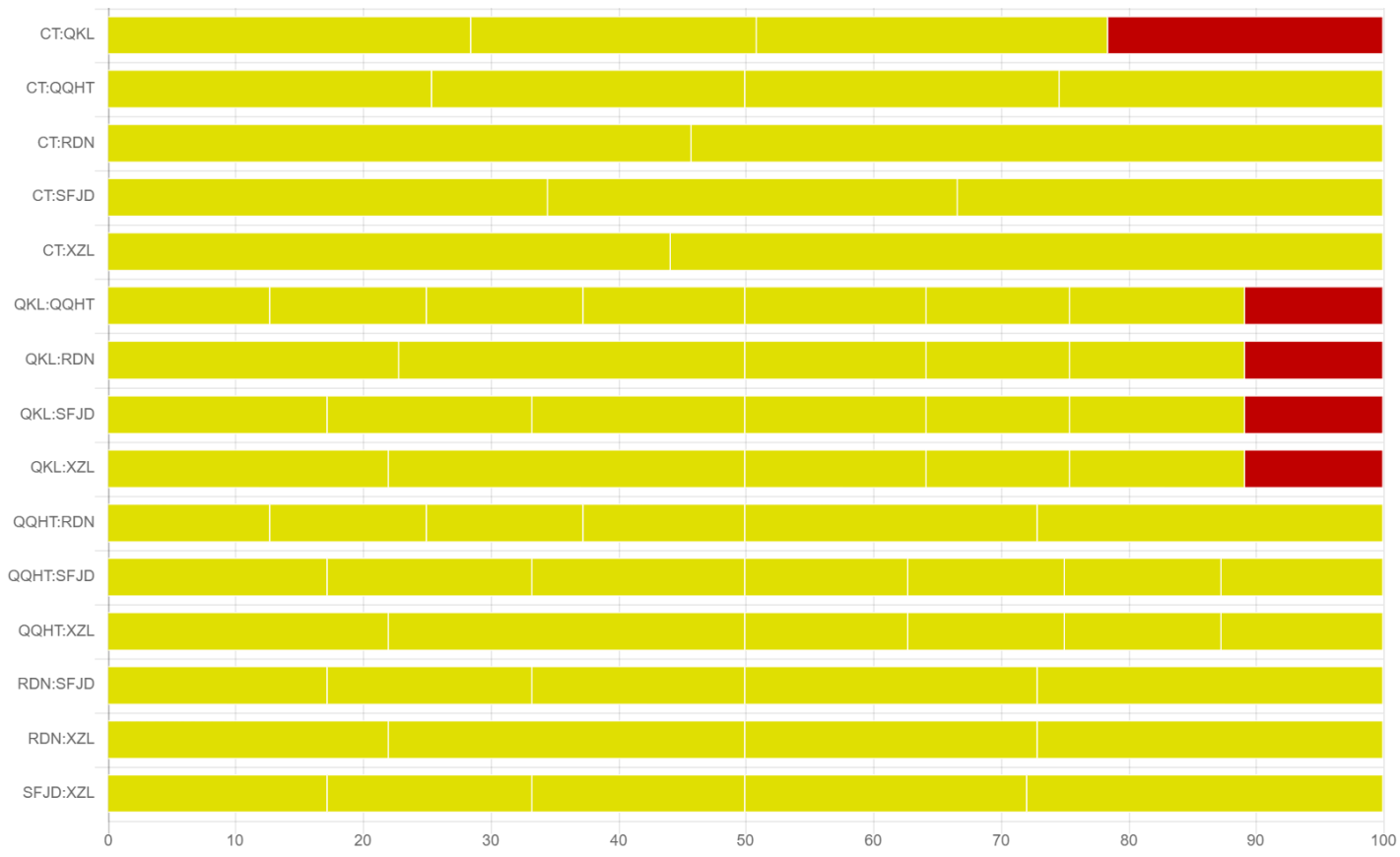
**

NOTE: **SFJD**: Shufeng Jiedu Capsule; **XZL**: Fresh Bamboo Juice Oral Liquid; **QQHT**: Qingqi Huatan Pill; **RDN**: Reduning Injection; **QKL**: Qingkailing Injection; **CT**: Conventional biomedicine treatment.

Figure S6.19:Risk of bias contribution by intervention group in **TNF-α**

**
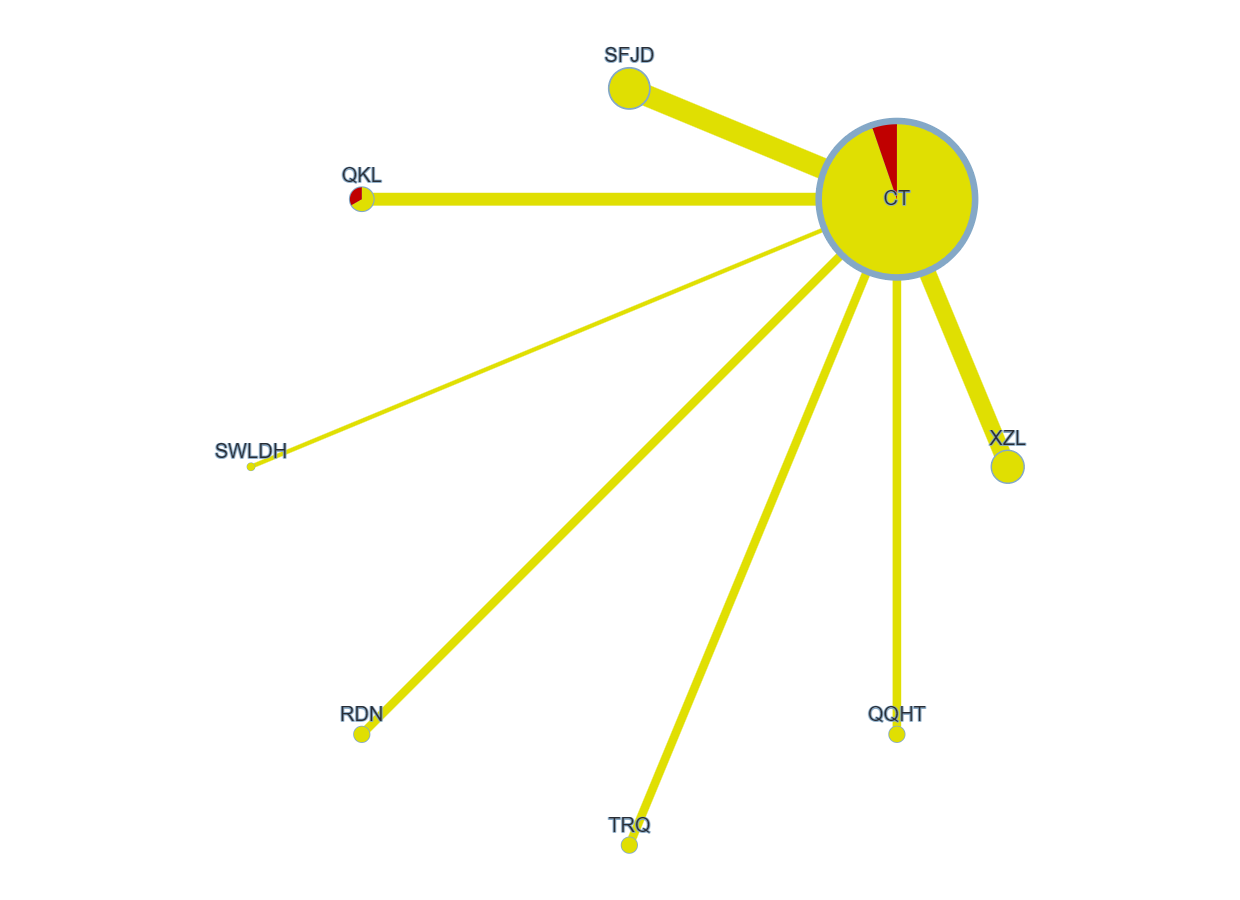
**

NOTE: **SFJD**: Shufeng Jiedu Capsule; **XZL**: Fresh Bamboo Juice Oral Liquid; **QQHT**: Qingqi Huatan Pill; **TRQ**: Tanreqing Injection; **RDN**: Reduning Injection; **SWLDH**: Shiwei Longdanhua Capsule; **QKL**: Qingkailing Injection; **CT**: Conventional biomedicine treatment.

Figure S6.20:Overall risk of bias by treatment comparison in **TNF-α**

**
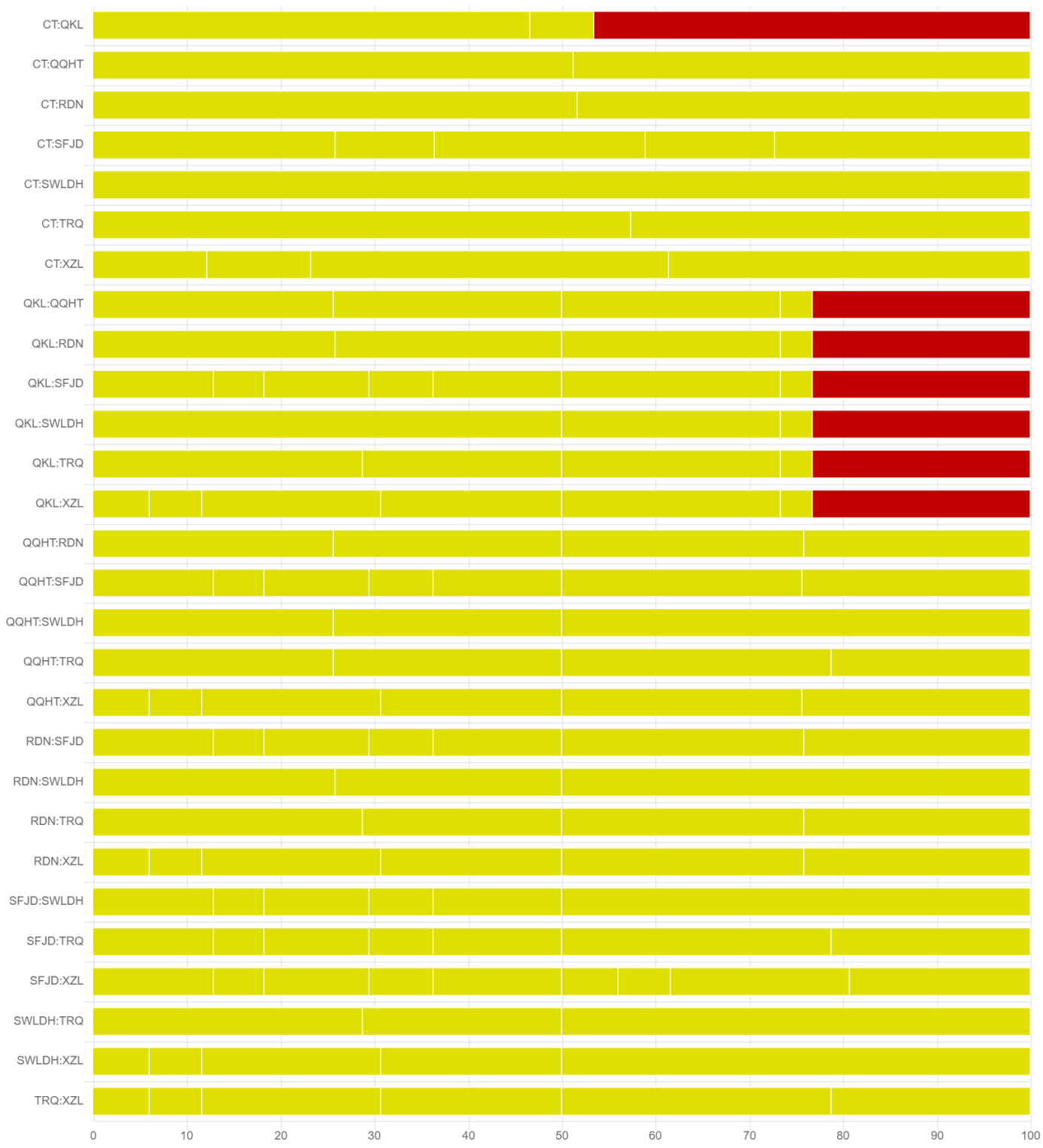
**

NOTE: **SFJD**: Shufeng Jiedu Capsule; **XZL**: Fresh Bamboo Juice Oral Liquid; **QQHT**: Qingqi Huatan Pill; **TRQ**: Tanreqing Injection; **RDN**: Reduning Injection; **SWLDH**: Shiwei Longdanhua Capsule; **QKL**: Qingkailing Injection; **CT**: Conventional biomedicine treatment.

Figure S6.21:Risk of bias contribution by intervention group in **Adverse event**

**
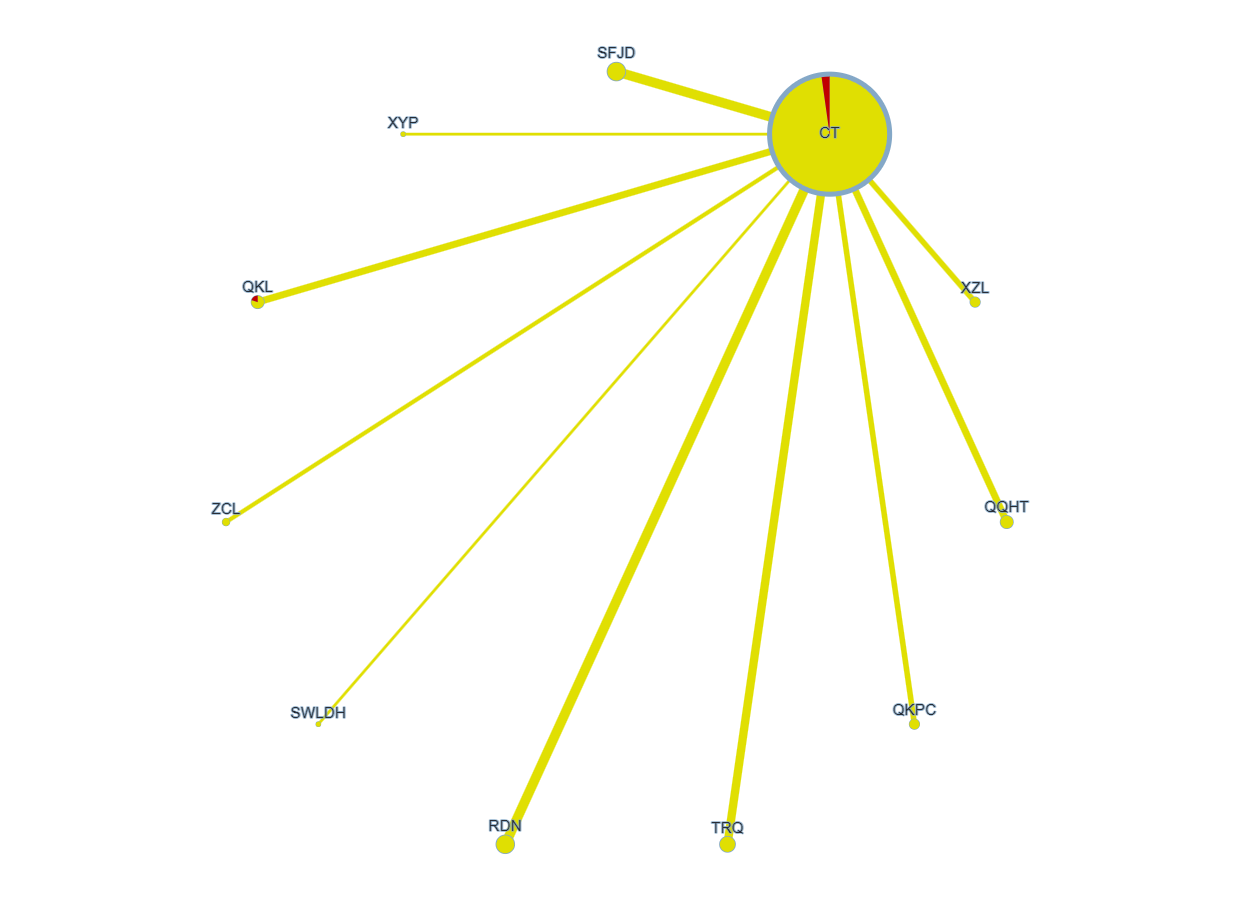
**

NOTE: **SFJD**: Shufeng Jiedu Capsule; **XZL**: Fresh Bamboo Juice Oral Liquid; **QQHT**: Qingqi Huatan Pill; **QKPC**: Qingke Pingchuan Granule; **TRQ**: Tanreqing Injection; **RDN**: Reduning Injection; **SWLDH**: Shiwei Longdanhua Capsule; **ZCL**: Zhichuanling Injection; **QKL**: Qingkailing Injection; **XYP**: Xiyanping Injection; **CT**: Conventional biomedicine treatment.

Figure S6.22:Overall risk of bias by treatment comparison in **Adverse event**

**
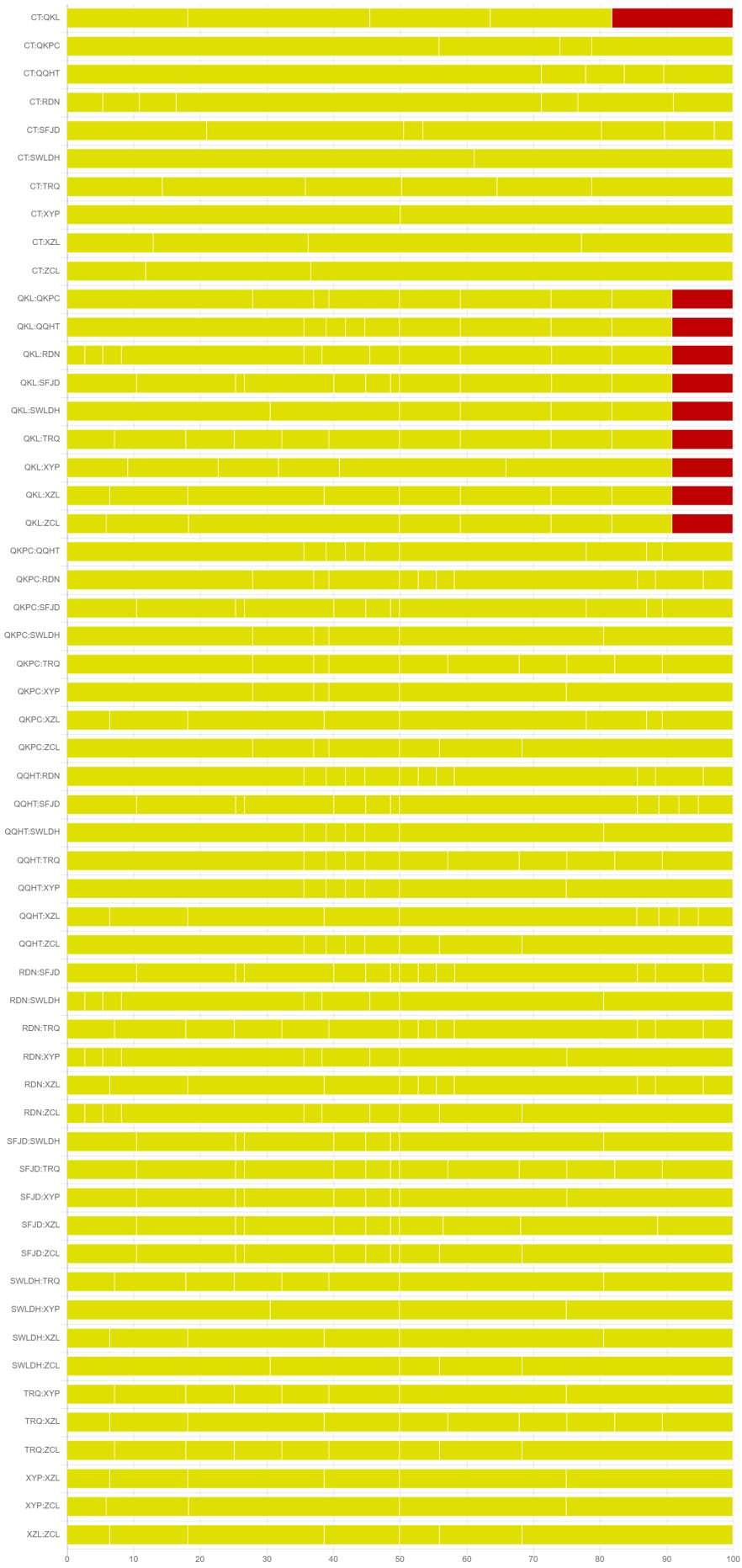
**

NOTE: **SFJD**: Shufeng Jiedu Capsule; **XZL**: Fresh Bamboo Juice Oral Liquid; **QQHT**: Qingqi Huatan Pill; **QKPC**: Qingke Pingchuan Granule; **TRQ**: Tanreqing Injection; **RDN**: Reduning Injection; **SWLDH**: Shiwei Longdanhua Capsule; **ZCL**: Zhichuanling Injection; **QKL**: Qingkailing Injection; **XYP**: Xiyanping Injection; **CT**: Conventional biomedicine treatment.

Table S6.1: Transitivity (Indirectness) Assessment

| **Randomised treatments** | **Age**  **(mean±SD)**  **(Years)** | **Disease duration  (mean±SD)**  **(Years)** |
| --- | --- | --- |
| SFJD+CT | 63.05±6.48 | 8.50±2.98 |
| XZL+CT | 61.70±7.03 | 10.91±4.41 |
| QQHT+CT | 59.68±8.13 | 8.52±3.15 |
| QKPC+CT | 66.44±9.61 | 16.69±5.02 |
| TRQ+CT | 62.96±8.92 | 12.48±4.05 |
| RDN+CT | 63.40±6.53 | 11.62±6.27 |
| SWLDH+CT | 59.23±8.96 | 9.90±5.28 |
| ZCL+CT | 60.76±7.37 | 6.81±3.20 |
| QKL+CT | 67.17±8.31 | 6.68±0.60 |
| XYP+CT | 66.91±8.12 | 8.78±4.01 |

NOTE: **SFJD**: Shufeng Jiedu Capsule; **XZL**: Fresh Bamboo Juice Oral Liquid; **QQHT**: Qingqi Huatan Pill; **QKPC**: Qingke Pingchuan Granule; **TRQ**: Tanreqing Injection; **RDN**: Reduning Injection; **SWLDH**: Shiwei Longdanhua Capsule;

**ZCL**: Zhichuanling Injection; **QKL**: Qingkailing Injection; **XYP**: Xiyanping Injection; **CT**: Conventional biomedicine treatment.

**Imprecision:** We use the CINeMA website to grade the accuracy of each comparison.

**Heterogeneity:** We assessed the degree of worry by comparing clinical reasoning based on 95% confidence intervals (CIs) while applying the same clinical

reasoning framework as for inaccuracy. In particular, we judged the consistency of our findings based on the confidence and prediction intervals associated with clinically important effect sizes. And we used the same thresholds of clinical significance as described above and followed the recommendations automatically provided by CINeMA (https://cinema.ispm.unibe.ch/).

Table S6.2: CINeMA Results of **Total effective rate**

| Comparison | Within-study bias | Reporting bias | Indirectness | Imprecision | Heterogeneity | Incoherence | Confidence rating |
| --- | --- | --- | --- | --- | --- | --- | --- |
| CT:QKL | Some concerns | Low risk | No concerns | No concerns | No concerns | Major concerns | Low |
| CT:QKPC | Some concerns | Low risk | No concerns | No concerns | No concerns | Major concerns | Low |
| CT:QQHT | Some concerns | Low risk | No concerns | No concerns | No concerns | Major concerns | Low |
| CT:RDN | Some concerns | Low risk | No concerns | No concerns | No concerns | Major concerns | Low |
| CT:SFJD | Some concerns | Low risk | No concerns | No concerns | Major concerns | Major concerns | Very low |
| CT:SWLDH | Some concerns | Low risk | No concerns | No concerns | Major concerns | Major concerns | Very low |
| CT:TRQ | Some concerns | Low risk | No concerns | No concerns | No concerns | Major concerns | Low |
| CT:XYP | Some concerns | Low risk | No concerns | No concerns | No concerns | Major concerns | Low |
| CT:XZL | Some concerns | Low risk | No concerns | No concerns | No concerns | Major concerns | Low |
| CT:ZCL | Some concerns | Low risk | No concerns | No concerns | No concerns | Major concerns | Low |
| QKL:QKPC | Some concerns | Low risk | No concerns | Major concerns | No concerns | Major concerns | Very low |
| QKL:QQHT | Some concerns | Low risk | No concerns | Major concerns | No concerns | Major concerns | Very low |
| QKL:RDN | Some concerns | Low risk | No concerns | Major concerns | No concerns | Major concerns | Very low |
| QKL:SFJD | Some concerns | Low risk | No concerns | Major concerns | No concerns | Major concerns | Very low |
| QKL:SWLDH | Some concerns | Low risk | No concerns | Major concerns | No concerns | Major concerns | Very low |
| QKL:TRQ | Some concerns | Low risk | No concerns | Major concerns | No concerns | Major concerns | Very low |
| QKL:XYP | Some concerns | Low risk | No concerns | Major concerns | No concerns | Major concerns | Very low |
| QKL:XZL | Some concerns | Low risk | No concerns | Major concerns | No concerns | Major concerns | Very low |
| QKL:ZCL | Some concerns | Low risk | No concerns | Major concerns | No concerns | Major concerns | Very low |
| QKPC:QQHT | Some concerns | Low risk | No concerns | Major concerns | No concerns | Major concerns | Very low |
| QKPC:RDN | Some concerns | Low risk | No concerns | Major concerns | No concerns | Major concerns | Very low |
| QKPC:SFJD | Some concerns | Low risk | No concerns | Major concerns | No concerns | Major concerns | Very low |
| QKPC:SWLDH | Some concerns | Low risk | No concerns | Major concerns | No concerns | Major concerns | Very low |
| QKPC:TRQ | Some concerns | Low risk | No concerns | Major concerns | No concerns | Major concerns | Very low |
| QKPC:XYP | Some concerns | Low risk | No concerns | Major concerns | No concerns | Major concerns | Very low |
| QKPC:XZL | Some concerns | Low risk | No concerns | Major concerns | No concerns | Major concerns | Very low |
| QKPC:ZCL | Some concerns | Low risk | No concerns | Major concerns | No concerns | Major concerns | Very low |
| QQHT:RDN | Some concerns | Low risk | No concerns | Major concerns | No concerns | Major concerns | Very low |
| QQHT:SFJD | Some concerns | Low risk | No concerns | Major concerns | No concerns | Major concerns | Very low |
| QQHT:SWLDH | Some concerns | Low risk | No concerns | Major concerns | No concerns | Major concerns | Very low |
| QQHT:TRQ | Some concerns | Low risk | No concerns | Major concerns | No concerns | Major concerns | Very low |
| QQHT:XYP | Some concerns | Low risk | No concerns | Major concerns | No concerns | Major concerns | Very low |
| QQHT:XZL | Some concerns | Low risk | No concerns | Major concerns | No concerns | Major concerns | Very low |
| QQHT:ZCL | Some concerns | Low risk | No concerns | Major concerns | No concerns | Major concerns | Very low |
| RDN:SFJD | Some concerns | Low risk | No concerns | No concerns | Major concerns | Major concerns | Very low |
| RDN:SWLDH | Some concerns | Low risk | No concerns | Major concerns | No concerns | Major concerns | Very low |
| RDN:TRQ | Some concerns | Low risk | No concerns | Major concerns | No concerns | Major concerns | Very low |
| RDN:XYP | Some concerns | Low risk | No concerns | Major concerns | No concerns | Major concerns | Very low |
| RDN:XZL | Some concerns | Low risk | No concerns | Major concerns | No concerns | Major concerns | Very low |
| RDN:ZCL | Some concerns | Low risk | No concerns | Major concerns | No concerns | Major concerns | Very low |
| SFJD:SWLDH | Some concerns | Low risk | No concerns | Major concerns | No concerns | Major concerns | Very low |
| SFJD:TRQ | Some concerns | Low risk | No concerns | Major concerns | No concerns | Major concerns | Very low |
| SFJD:XYP | Some concerns | Low risk | No concerns | Major concerns | No concerns | Major concerns | Very low |
| SFJD:XZL | Some concerns | Low risk | No concerns | Major concerns | No concerns | Major concerns | Very low |
| SFJD:ZCL | Some concerns | Low risk | No concerns | Major concerns | No concerns | Major concerns | Very low |
| SWLDH:TRQ | Some concerns | Low risk | No concerns | Major concerns | No concerns | Major concerns | Very low |
| SWLDH:XYP | Some concerns | Low risk | No concerns | Major concerns | No concerns | Major concerns | Very low |
| SWLDH:XZL | Some concerns | Low risk | No concerns | Major concerns | No concerns | Major concerns | Very low |
| SWLDH:ZCL | Some concerns | Low risk | No concerns | Major concerns | No concerns | Major concerns | Very low |
| TRQ:XYP | Some concerns | Low risk | No concerns | Major concerns | No concerns | Major concerns | Very low |
| TRQ:XZL | Some concerns | Low risk | No concerns | Major concerns | No concerns | Major concerns | Very low |
| TRQ:ZCL | Some concerns | Low risk | No concerns | Major concerns | No concerns | Major concerns | Very low |
| XYP:XZL | Some concerns | Low risk | No concerns | Major concerns | No concerns | Major concerns | Very low |
| XYP:ZCL | Some concerns | Low risk | No concerns | Major concerns | No concerns | Major concerns | Very low |

NOTE: **SFJD**: Shufeng Jiedu Capsule; **XZL**: Fresh Bamboo Juice Oral Liquid; **QQHT**: Qingqi Huatan Pill; **QKPC**: Qingke Pingchuan Granule; **TRQ**: Tanreqing Injection; **RDN**: Reduning Injection; **SWLDH**: Shiwei Longdanhua Capsule; **ZCL**: Zhichuanling Injection; **QKL**: Qingkailing Injection; **XYP**: Xiyanping Injection; **CT:** Conventional biomedicine treatment.

Table S6.3: CINeMA Results of **FVC**

| Comparison | Within-study bias | Reporting bias | Indirectness | Imprecision | Heterogeneity | Incoherence | Confidence rating |
| --- | --- | --- | --- | --- | --- | --- | --- |
| CT:QKL | Some concerns | Low risk | No concerns | No concerns | Major concerns | Major concerns | Very low |
| CT:QKPC | Some concerns | Low risk | No concerns | Major concerns | No concerns | Major concerns | Very low |
| CT:QQHT | Some concerns | Low risk | No concerns | No concerns | Major concerns | Major concerns | Very low |
| CT:RDN | Some concerns | Low risk | No concerns | No concerns | Major concerns | Major concerns | Very low |
| CT:SFJD | Some concerns | Low risk | No concerns | No concerns | Major concerns | Major concerns | Very low |
| CT:SFJD+ | Some concerns | Low risk | No concerns | No concerns | Major concerns | Major concerns | Very low |
| CT:SWLDH | Some concerns | Low risk | No concerns | No concerns | No concerns | Major concerns | Low |
| CT:TRQ | Some concerns | Low risk | No concerns | No concerns | Major concerns | Major concerns | Very low |
| CT:XYP | Some concerns | Low risk | No concerns | No concerns | Major concerns | Major concerns | Very low |
| CT:XZL | Some concerns | Low risk | No concerns | Major concerns | No concerns | Major concerns | Very low |
| CT:ZCL | Some concerns | Low risk | No concerns | No concerns | No concerns | Major concerns | Low |
| QKL:QKPC | Some concerns | Low risk | No concerns | Major concerns | No concerns | Major concerns | Very low |
| QKL:QQHT | Some concerns | Low risk | No concerns | No concerns | No concerns | Major concerns | Low |
| QKL:RDN | Some concerns | Low risk | No concerns | Major concerns | No concerns | Major concerns | Very low |
| QKL:SFJD | Some concerns | Low risk | No concerns | Major concerns | No concerns | Major concerns | Very low |
| QKL:SFJD+ | Some concerns | Low risk | No concerns | Major concerns | No concerns | Major concerns | Very low |
| QKL:SWLDH | Some concerns | Low risk | No concerns | Major concerns | No concerns | Major concerns | Very low |
| QKL:TRQ | Some concerns | Low risk | No concerns | Major concerns | No concerns | Major concerns | Very low |
| QKL:XYP | Some concerns | Low risk | No concerns | Major concerns | No concerns | Major concerns | Very low |
| QKL:XZL | Some concerns | Low risk | No concerns | Major concerns | No concerns | Major concerns | Very low |
| QKL:ZCL | Some concerns | Low risk | No concerns | Major concerns | No concerns | Major concerns | Very low |
| QKPC:QQHT | Some concerns | Low risk | No concerns | Major concerns | No concerns | Major concerns | Very low |
| QKPC:RDN | Some concerns | Low risk | No concerns | Major concerns | No concerns | Major concerns | Very low |
| QKPC:SFJD | Some concerns | Low risk | No concerns | Major concerns | No concerns | Major concerns | Very low |
| QKPC:SFJD+ | Some concerns | Low risk | No concerns | Major concerns | No concerns | Major concerns | Very low |
| QKPC:SWLDH | Some concerns | Low risk | No concerns | No concerns | Major concerns | Major concerns | Very low |
| QKPC:TRQ | Some concerns | Low risk | No concerns | Major concerns | No concerns | Major concerns | Very low |
| QKPC:XYP | Some concerns | Low risk | No concerns | Major concerns | No concerns | Major concerns | Very low |
| QKPC:XZL | Some concerns | Low risk | No concerns | Major concerns | No concerns | Major concerns | Very low |
| QKPC:ZCL | Some concerns | Low risk | No concerns | Major concerns | No concerns | Major concerns | Very low |
| QQHT:RDN | Some concerns | Low risk | No concerns | No concerns | No concerns | Major concerns | Low |
| QQHT:SFJD | Some concerns | Low risk | No concerns | No concerns | No concerns | Major concerns | Low |
| QQHT:SFJD+ | Some concerns | Low risk | No concerns | No concerns | No concerns | Major concerns | Low |
| QQHT:SWLDH | Some concerns | Low risk | No concerns | No concerns | No concerns | Major concerns | Low |
| QQHT:TRQ | Some concerns | Low risk | No concerns | No concerns | No concerns | Major concerns | Low |
| QQHT:XYP | Some concerns | Low risk | No concerns | No concerns | No concerns | Major concerns | Low |
| QQHT:XZL | Some concerns | Low risk | No concerns | No concerns | Major concerns | Major concerns | Very low |
| QQHT:ZCL | Some concerns | Low risk | No concerns | No concerns | No concerns | Major concerns | Very low |
| RDN:SFJD | Some concerns | Low risk | No concerns | Major concerns | No concerns | Major concerns | Very low |
| RDN:SFJD+ | Some concerns | Low risk | No concerns | Major concerns | No concerns | Major concerns | Very low |
| RDN:SWLDH | Some concerns | Low risk | No concerns | Major concerns | No concerns | Major concerns | Very low |
| RDN:TRQ | Some concerns | Low risk | No concerns | Major concerns | No concerns | Major concerns | Very low |
| RDN:XYP | Some concerns | Low risk | No concerns | Major concerns | No concerns | Major concerns | Very low |
| RDN:XZL | Some concerns | Low risk | No concerns | Major concerns | No concerns | Major concerns | Very low |
| RDN:ZCL | Some concerns | Low risk | No concerns | Major concerns | No concerns | Major concerns | Very low |
| SFJD:SFJD+ | Some concerns | Low risk | No concerns | Major concerns | No concerns | Major concerns | Very low |
| SFJD:SWLDH | Some concerns | Low risk | No concerns | Major concerns | No concerns | Major concerns | Very low |
| SFJD:TRQ | Some concerns | Low risk | No concerns | Major concerns | No concerns | Major concerns | Very low |
| SFJD:XYP | Some concerns | Low risk | No concerns | Major concerns | No concerns | Major concerns | Very low |
| SFJD:XZL | Some concerns | Low risk | No concerns | Major concerns | No concerns | Major concerns | Very low |
| SFJD:ZCL | Some concerns | Low risk | No concerns | Major concerns | No concerns | Major concerns | Very low |
| SFJD+:SWLDH | Some concerns | Low risk | No concerns | Major concerns | No concerns | Major concerns | Very low |
| SFJD+:TRQ | Some concerns | Low risk | No concerns | Major concerns | No concerns | Major concerns | Very low |
| SFJD+:XYP | Some concerns | Low risk | No concerns | Major concerns | No concerns | Major concerns | Very low |
| SFJD+:XZL | Some concerns | Low risk | No concerns | Major concerns | No concerns | Major concerns | Very low |
| SFJD+:ZCL | Some concerns | Low risk | No concerns | Major concerns | No concerns | Major concerns | Very low |
| SWLDH:TRQ | Some concerns | Low risk | No concerns | Major concerns | No concerns | Major concerns | Very low |
| SWLDH:XYP | Some concerns | Low risk | No concerns | Major concerns | No concerns | Major concerns | Very low |
| SWLDH:XZL | Some concerns | Low risk | No concerns | No concerns | Major concerns | Major concerns | Very low |
| SWLDH:ZCL | Some concerns | Low risk | No concerns | Major concerns | No concerns | Major concerns | Very low |
| TRQ:XYP | Some concerns | Low risk | No concerns | Major concerns | No concerns | Major concerns | Very low |
| TRQ:XZL | Some concerns | Low risk | No concerns | Major concerns | No concerns | Major concerns | Very low |
| TRQ:ZCL | Some concerns | Low risk | No concerns | Major concerns | No concerns | Major concerns | Very low |
| XYP:XZL | Some concerns | Low risk | No concerns | Major concerns | No concerns | Major concerns | Very low |
| XYP:ZCL | Some concerns | Low risk | No concerns | Major concerns | No concerns | Major concerns | Very low |
| XZL:ZCL | Some concerns | Low risk | No concerns | No concerns | Major concerns | Major concerns | Very low |

NOTE: **SFJD**: Shufeng Jiedu Capsule; **XZL**: Fresh Bamboo Juice Oral Liquid; **QQHT**: Qingqi Huatan Pill; **QKPC**: Qingke Pingchuan Granule; **TRQ**: Tanreqing Injection; **RDN**: Reduning Injection; **SWLDH**: Shiwei Longdanhua Capsule; **ZCL**: Zhichuanling Injection; **QKL**: Qingkailing Injection; **XYP**: Xiyanping Injection; **CT:** Conventional biomedicine treatment.

Table S6.4: CINeMA Results of **FEV1**

| Comparison | Within-study bias | Reporting bias | Indirectness | Imprecision | Heterogeneity | Incoherence | Confidence rating |
| --- | --- | --- | --- | --- | --- | --- | --- |
| CT:QKL | Some concerns | Low risk | No concerns | Major concerns | No concerns | Major concerns | Very low |
| CT:QKPC | Some concerns | Low risk | No concerns | Major concerns | No concerns | Major concerns | Very low |
| CT:QQHT | Some concerns | Low risk | No concerns | Major concerns | No concerns | Major concerns | Very low |
| CT:RDN | Some concerns | Low risk | No concerns | Major concerns | No concerns | Major concerns | Very low |
| CT:SFJD | Some concerns | Low risk | No concerns | Major concerns | No concerns | Major concerns | Very low |
| CT:SWLDH | Some concerns | Low risk | No concerns | Major concerns | No concerns | Major concerns | Very low |
| CT:TRQ | Some concerns | Low risk | No concerns | Major concerns | No concerns | Major concerns | Very low |
| CT:XYP | Some concerns | Low risk | No concerns | Major concerns | No concerns | Major concerns | Very low |
| CT:XZL | Some concerns | Low risk | No concerns | Major concerns | No concerns | Major concerns | Very low |
| CT:ZCL | Some concerns | Low risk | No concerns | Major concerns | No concerns | Major concerns | Very low |
| QKL:QKPC | Some concerns | Low risk | No concerns | Major concerns | No concerns | Major concerns | Very low |
| QKL:QQHT | Some concerns | Low risk | No concerns | Major concerns | No concerns | Major concerns | Very low |
| QKL:RDN | Some concerns | Low risk | No concerns | Major concerns | No concerns | Major concerns | Very low |
| QKL:SFJD | Some concerns | Low risk | No concerns | Major concerns | No concerns | Major concerns | Very low |
| QKL:SWLDH | Some concerns | Low risk | No concerns | Major concerns | No concerns | Major concerns | Very low |
| QKL:TRQ | Some concerns | Low risk | No concerns | Major concerns | No concerns | Major concerns | Very low |
| QKL:XYP | Some concerns | Low risk | No concerns | Major concerns | No concerns | Major concerns | Very low |
| QKL:XZL | Some concerns | Low risk | No concerns | Major concerns | No concerns | Major concerns | Very low |
| QKL:ZCL | Some concerns | Low risk | No concerns | Major concerns | No concerns | Major concerns | Very low |
| QKPC:QQHT | Some concerns | Low risk | No concerns | Major concerns | No concerns | Major concerns | Very low |
| QKPC:RDN | Some concerns | Low risk | No concerns | Major concerns | No concerns | Major concerns | Very low |
| QKPC:SFJD | Some concerns | Low risk | No concerns | Major concerns | No concerns | Major concerns | Very low |
| QKPC:SWLDH | Some concerns | Low risk | No concerns | Major concerns | No concerns | Major concerns | Very low |
| QKPC:TRQ | Some concerns | Low risk | No concerns | Major concerns | No concerns | Major concerns | Very low |
| QKPC:XYP | Some concerns | Low risk | No concerns | Major concerns | No concerns | Major concerns | Very low |
| QKPC:XZL | Some concerns | Low risk | No concerns | Major concerns | No concerns | Major concerns | Very low |
| QKPC:ZCL | Some concerns | Low risk | No concerns | Major concerns | No concerns | Major concerns | Very low |
| QQHT:RDN | Some concerns | Low risk | No concerns | Major concerns | No concerns | Major concerns | Very low |
| QQHT:SFJD | Some concerns | Low risk | No concerns | Major concerns | No concerns | Major concerns | Very low |
| QQHT:SWLDH | Some concerns | Low risk | No concerns | Major concerns | No concerns | Major concerns | Very low |
| QQHT:TRQ | Some concerns | Low risk | No concerns | Major concerns | No concerns | Major concerns | Very low |
| QQHT:XYP | Some concerns | Low risk | No concerns | Major concerns | No concerns | Major concerns | Very low |
| QQHT:XZL | Some concerns | Low risk | No concerns | Major concerns | No concerns | Major concerns | Very low |
| QQHT:ZCL | Some concerns | Low risk | No concerns | Major concerns | No concerns | Major concerns | Very low |
| RDN:SFJD | Some concerns | Low risk | No concerns | Major concerns | No concerns | Major concerns | Very low |
| RDN:SWLDH | Some concerns | Low risk | No concerns | Major concerns | No concerns | Major concerns | Very low |
| RDN:TRQ | Some concerns | Low risk | No concerns | Major concerns | No concerns | Major concerns | Very low |
| RDN:XYP | Some concerns | Low risk | No concerns | Major concerns | No concerns | Major concerns | Very low |
| RDN:XZL | Some concerns | Low risk | No concerns | Major concerns | No concerns | Major concerns | Very low |
| RDN:ZCL | Some concerns | Low risk | No concerns | Major concerns | No concerns | Major concerns | Very low |
| SFJD:SWLDH | Some concerns | Low risk | No concerns | Major concerns | No concerns | Major concerns | Very low |
| SFJD:TRQ | Some concerns | Low risk | No concerns | Major concerns | No concerns | Major concerns | Very low |
| SFJD:XYP | Some concerns | Low risk | No concerns | Major concerns | No concerns | Major concerns | Very low |
| SFJD:XZL | Some concerns | Low risk | No concerns | Major concerns | No concerns | Major concerns | Very low |
| SFJD:ZCL | Some concerns | Low risk | No concerns | Major concerns | No concerns | Major concerns | Very low |
| SWLDH:TRQ | Some concerns | Low risk | No concerns | Major concerns | No concerns | Major concerns | Very low |
| SWLDH:XYP | Some concerns | Low risk | No concerns | Major concerns | No concerns | Major concerns | Very low |
| SWLDH:XZL | Some concerns | Low risk | No concerns | Major concerns | No concerns | Major concerns | Very low |
| SWLDH:ZCL | Some concerns | Low risk | No concerns | Major concerns | No concerns | Major concerns | Very low |
| TRQ:XYP | Some concerns | Low risk | No concerns | Major concerns | No concerns | Major concerns | Very low |
| TRQ:XZL | Some concerns | Low risk | No concerns | Major concerns | No concerns | Major concerns | Very low |
| TRQ:ZCL | Some concerns | Low risk | No concerns | Major concerns | No concerns | Major concerns | Very low |
| XYP:XZL | Some concerns | Low risk | No concerns | Major concerns | No concerns | Major concerns | Very low |
| XYP:ZCL | Some concerns | Low risk | No concerns | Major concerns | No concerns | Major concerns | Very low |
| XZL:ZCL | Some concerns | Low risk | No concerns | Major concerns | No concerns | Major concerns | Very low |

NOTE: **SFJD**: Shufeng Jiedu Capsule; **XZL**: Fresh Bamboo Juice Oral Liquid; **QQHT**: Qingqi Huatan Pill; **QKPC**: Qingke Pingchuan Granule; **TRQ**: Tanreqing Injection; **RDN**: Reduning Injection; **SWLDH**: Shiwei Longdanhua Capsule; **ZCL**: Zhichuanling Injection; **QKL**: Qingkailing Injection; **XYP**: Xiyanping Injection; **CT:** Conventional biomedicine treatment.

Table S6.5: CINeMA Results of **FEV1/FVC**

| Comparison | Within-study bias | Reporting bias | Indirectness | Imprecision | Heterogeneity | Incoherence | Confidence rating |
| --- | --- | --- | --- | --- | --- | --- | --- |
| CT:QKL | Some concerns | Low risk | No concerns | No concerns | Major concerns | Major concerns | Very low |
| CT:QKPC | Some concerns | Low risk | No concerns | Major concerns | No concerns | Major concerns | Very low |
| CT:QQHT | Some concerns | Low risk | No concerns | Major concerns | No concerns | Major concerns | Very low |
| CT:RDN | Some concerns | Low risk | No concerns | No concerns | Major concerns | Major concerns | Very low |
| CT:SFJD | Some concerns | Low risk | No concerns | No concerns | Major concerns | Major concerns | Very low |
| CT:SWLDH | Some concerns | Low risk | No concerns | Major concerns | No concerns | Major concerns | Very low |
| CT:TRQ | Some concerns | Low risk | No concerns | Major concerns | No concerns | Major concerns | Very low |
| CT:XYP | Some concerns | Low risk | No concerns | Major concerns | No concerns | Major concerns | Very low |
| CT:XZL | Some concerns | Low risk | No concerns | No concerns | Major concerns | Major concerns | Very low |
| CT:ZCL | Some concerns | Low risk | No concerns | Major concerns | No concerns | Major concerns | Very low |
| QKL:QKPC | Some concerns | Low risk | No concerns | Major concerns | No concerns | Major concerns | Very low |
| QKL:QQHT | Some concerns | Low risk | No concerns | Major concerns | No concerns | Major concerns | Very low |
| QKL:RDN | Some concerns | Low risk | No concerns | Major concerns | No concerns | Major concerns | Very low |
| QKL:SFJD | Some concerns | Low risk | No concerns | Major concerns | No concerns | Major concerns | Very low |
| QKL:SWLDH | Some concerns | Low risk | No concerns | Major concerns | No concerns | Major concerns | Very low |
| QKL:TRQ | Some concerns | Low risk | No concerns | Major concerns | No concerns | Major concerns | Very low |
| QKL:XYP | Some concerns | Low risk | No concerns | Major concerns | No concerns | Major concerns | Very low |
| QKL:XZL | Some concerns | Low risk | No concerns | Major concerns | No concerns | Major concerns | Very low |
| QKL:ZCL | Some concerns | Low risk | No concerns | Major concerns | No concerns | Major concerns | Very low |
| QKPC:QQHT | Some concerns | Low risk | No concerns | Major concerns | No concerns | Major concerns | Very low |
| QKPC:RDN | Some concerns | Low risk | No concerns | Major concerns | No concerns | Major concerns | Very low |
| QKPC:SFJD | Some concerns | Low risk | No concerns | Major concerns | No concerns | Major concerns | Very low |
| QKPC:SWLDH | Some concerns | Low risk | No concerns | Major concerns | No concerns | Major concerns | Very low |
| QKPC:TRQ | Some concerns | Low risk | No concerns | Major concerns | No concerns | Major concerns | Very low |
| QKPC:XYP | Some concerns | Low risk | No concerns | Major concerns | No concerns | Major concerns | Very low |
| QKPC:XZL | Some concerns | Low risk | No concerns | Major concerns | No concerns | Major concerns | Very low |
| QKPC:ZCL | Some concerns | Low risk | No concerns | Major concerns | No concerns | Major concerns | Very low |
| QQHT:RDN | Some concerns | Low risk | No concerns | Major concerns | No concerns | Major concerns | Very low |
| QQHT:SFJD | Some concerns | Low risk | No concerns | Major concerns | No concerns | Major concerns | Very low |
| QQHT:SWLDH | Some concerns | Low risk | No concerns | Major concerns | No concerns | Major concerns | Very low |
| QQHT:TRQ | Some concerns | Low risk | No concerns | Major concerns | No concerns | Major concerns | Very low |
| QQHT:XYP | Some concerns | Low risk | No concerns | Major concerns | No concerns | Major concerns | Very low |
| QQHT:XZL | Some concerns | Low risk | No concerns | Major concerns | No concerns | Major concerns | Very low |
| QQHT:ZCL | Some concerns | Low risk | No concerns | Major concerns | No concerns | Major concerns | Very low |
| RDN:SFJD | Some concerns | Low risk | No concerns | Major concerns | No concerns | Major concerns | Very low |
| RDN:SWLDH | Some concerns | Low risk | No concerns | Major concerns | No concerns | Major concerns | Very low |
| RDN:TRQ | Some concerns | Low risk | No concerns | Major concerns | No concerns | Major concerns | Very low |
| RDN:XYP | Some concerns | Low risk | No concerns | Major concerns | No concerns | Major concerns | Very low |
| RDN:XZL | Some concerns | Low risk | No concerns | Major concerns | No concerns | Major concerns | Very low |
| RDN:ZCL | Some concerns | Low risk | No concerns | Major concerns | No concerns | Major concerns | Very low |
| SFJD:SWLDH | Some concerns | Low risk | No concerns | Major concerns | No concerns | Major concerns | Very low |
| SFJD:TRQ | Some concerns | Low risk | No concerns | Major concerns | No concerns | Major concerns | Very low |
| SFJD:XYP | Some concerns | Low risk | No concerns | Major concerns | No concerns | Major concerns | Very low |
| SFJD:XZL | Some concerns | Low risk | No concerns | Major concerns | No concerns | Major concerns | Very low |
| SFJD:ZCL | Some concerns | Low risk | No concerns | Major concerns | No concerns | Major concerns | Very low |
| SWLDH:TRQ | Some concerns | Low risk | No concerns | Major concerns | No concerns | Major concerns | Very low |
| SWLDH:XYP | Some concerns | Low risk | No concerns | Major concerns | No concerns | Major concerns | Very low |
| SWLDH:XZL | Some concerns | Low risk | No concerns | Major concerns | No concerns | Major concerns | Very low |
| SWLDH:ZCL | Some concerns | Low risk | No concerns | Major concerns | No concerns | Major concerns | Very low |
| TRQ:XYP | Some concerns | Low risk | No concerns | Major concerns | No concerns | Major concerns | Very low |
| TRQ:XZL | Some concerns | Low risk | No concerns | Major concerns | No concerns | Major concerns | Very low |
| TRQ:ZCL | Some concerns | Low risk | No concerns | Major concerns | No concerns | Major concerns | Very low |
| XYP:XZL | Some concerns | Low risk | No concerns | Major concerns | No concerns | Major concerns | Very low |
| XYP:ZCL | Some concerns | Low risk | No concerns | Major concerns | No concerns | Major concerns | Very low |
| XZL:ZCL | Some concerns | Low risk | No concerns | Major concerns | No concerns | Major concerns | Very low |

NOTE: **SFJD**: Shufeng Jiedu Capsule; **XZL**: Fresh Bamboo Juice Oral Liquid; **QQHT**: Qingqi Huatan Pill; **QKPC**: Qingke Pingchuan Granule; **TRQ**: Tanreqing Injection; **RDN**: Reduning Injection; **SWLDH**: Shiwei Longdanhua Capsule; **ZCL**: Zhichuanling Injection; **QKL**: Qingkailing Injection; **XYP**: Xiyanping Injection; **CT:** Conventional biomedicine treatment.

Table S6.6: CINeMA Results of **PH**

| Comparison | Within-study bias | Reporting bias | Indirectness | Imprecision | Heterogeneity | Incoherence | Confidence rating |
| --- | --- | --- | --- | --- | --- | --- | --- |
| CT:QKL | Major concerns | Low risk | No concerns | No concerns | Major concerns | Major concerns | Very low |
| CT:QKPC | Some concerns | Low risk | No concerns | Major concerns | No concerns | Major concerns | Very low |
| CT:RDN | Some concerns | Low risk | No concerns | No concerns | Major concerns | Major concerns | Very low |
| CT:TRQ | Some concerns | Low risk | No concerns | Major concerns | No concerns | Major concerns | Very low |
| CT:XZL | Some concerns | Low risk | No concerns | Major concerns | No concerns | Major concerns | Very low |
| QKL:QKPC | Some concerns | Low risk | No concerns | Major concerns | No concerns | Major concerns | Very low |
| QKL:RDN | Some concerns | Low risk | No concerns | Major concerns | No concerns | Major concerns | Very low |
| QKL:TRQ | Some concerns | Low risk | No concerns | Major concerns | No concerns | Major concerns | Very low |
| QKL:XZL | Some concerns | Low risk | No concerns | Major concerns | No concerns | Major concerns | Very low |
| QKPC:RDN | Some concerns | Low risk | No concerns | Major concerns | No concerns | Major concerns | Very low |
| QKPC:TRQ | Some concerns | Low risk | No concerns | Major concerns | No concerns | Major concerns | Very low |
| QKPC:XZL | Some concerns | Low risk | No concerns | Major concerns | No concerns | Major concerns | Very low |
| RDN:TRQ | Some concerns | Low risk | No concerns | Major concerns | No concerns | Major concerns | Very low |
| RDN:XZL | Some concerns | Low risk | No concerns | Major concerns | No concerns | Major concerns | Very low |
| TRQ:XZL | Some concerns | Low risk | No concerns | Major concerns | No concerns | Major concerns | Very low |

NOTE: **QKL**: Qingkailing Injection; **QKPC**: Qingke Pingchuan Granule; **RDN**: Reduning Injection; **TRQ**: Tanreqing Injection; **XZL**: Fresh Bamboo Juice Oral Liquid; **CT:** Conventional biomedicine treatment.

Table S6.7: CINeMA Results of **PaO2**

| Comparison | Within-study bias | Reporting bias | Indirectness | Imprecision | Heterogeneity | Incoherence | Confidence rating |
| --- | --- | --- | --- | --- | --- | --- | --- |
| CT:QKL | Some concerns | Low risk | No concerns | No concerns | Major concerns | Major concerns | Very low |
| CT:QKPC | Some concerns | Low risk | No concerns | No concerns | Major concerns | Major concerns | Very low |
| CT:QQHT | Some concerns | Low risk | No concerns | No concerns | Major concerns | Major concerns | Very low |
| CT:RDN | Some concerns | Low risk | No concerns | No concerns | No concerns | Major concerns | Low |
| CT:SFJD | Some concerns | Low risk | No concerns | No concerns | Major concerns | Major concerns | Very low |
| CT:SWLDH | Some concerns | Low risk | No concerns | Major concerns | No concerns | Major concerns | Very low |
| CT:TRQ | Some concerns | Low risk | No concerns | No concerns | Major concerns | Major concerns | Very low |
| CT:XYP | Some concerns | Low risk | No concerns | Major concerns | No concerns | Major concerns | Very low |
| CT:XZL | Some concerns | Low risk | No concerns | No concerns | No concerns | Major concerns | Low |
| QKL:QKPC | Some concerns | Low risk | No concerns | Major concerns | No concerns | Major concerns | Very low |
| QKL:QQHT | Some concerns | Low risk | No concerns | Major concerns | No concerns | Major concerns | Very low |
| QKL:RDN | Some concerns | Low risk | No concerns | Major concerns | No concerns | Major concerns | Very low |
| QKL:SFJD | Some concerns | Low risk | No concerns | Major concerns | No concerns | Major concerns | Very low |
| QKL:SWLDH | Some concerns | Low risk | No concerns | Major concerns | No concerns | Major concerns | Very low |
| QKL:TRQ | Some concerns | Low risk | No concerns | Major concerns | No concerns | Major concerns | Very low |
| QKL:XYP | Some concerns | Low risk | No concerns | Major concerns | No concerns | Major concerns | Very low |
| QKL:XZL | Some concerns | Low risk | No concerns | No concerns | Major concerns | Major concerns | Very low |
| QKPC:QQHT | Some concerns | Low risk | No concerns | Major concerns | No concerns | Major concerns | Very low |
| QKPC:RDN | Some concerns | Low risk | No concerns | Major concerns | No concerns | Major concerns | Very low |
| QKPC:SFJD | Some concerns | Low risk | No concerns | Major concerns | No concerns | Major concerns | Very low |
| QKPC:SWLDH | Some concerns | Low risk | No concerns | Major concerns | No concerns | Major concerns | Very low |
| QKPC:TRQ | Some concerns | Low risk | No concerns | Major concerns | No concerns | Major concerns | Very low |
| QKPC:XYP | Some concerns | Low risk | No concerns | Major concerns | No concerns | Major concerns | Very low |
| QKPC:XZL | Some concerns | Low risk | No concerns | No concerns | Major concerns | Major concerns | Very low |
| QQHT:RDN | Some concerns | Low risk | No concerns | Major concerns | No concerns | Major concerns | Very low |
| QQHT:SFJD | Some concerns | Low risk | No concerns | Major concerns | No concerns | Major concerns | Very low |
| QQHT:SWLDH | Some concerns | Low risk | No concerns | Major concerns | No concerns | Major concerns | Very low |
| QQHT:TRQ | Some concerns | Low risk | No concerns | Major concerns | No concerns | Major concerns | Very low |
| QQHT:XYP | Some concerns | Low risk | No concerns | Major concerns | No concerns | Major concerns | Very low |
| QQHT:XZL | Some concerns | Low risk | No concerns | No concerns | Major concerns | Major concerns | Very low |
| RDN:SFJD | Some concerns | Low risk | No concerns | Major concerns | No concerns | Major concerns | Very low |
| RDN:SWLDH | Some concerns | Low risk | No concerns | Major concerns | No concerns | Major concerns | Very low |
| RDN:TRQ | Some concerns | Low risk | No concerns | Major concerns | No concerns | Major concerns | Very low |
| RDN:XYP | Some concerns | Low risk | No concerns | Major concerns | No concerns | Major concerns | Very low |
| RDN:XZL | Some concerns | Low risk | No concerns | No concerns | Major concerns | Major concerns | Very low |
| SFJD:SWLDH | Some concerns | Low risk | No concerns | Major concerns | No concerns | Major concerns | Very low |
| SFJD:TRQ | Some concerns | Low risk | No concerns | Major concerns | No concerns | Major concerns | Very low |
| SFJD:XYP | Some concerns | Low risk | No concerns | Major concerns | No concerns | Major concerns | Very low |
| SFJD:XZL | Some concerns | Low risk | No concerns | No concerns | No concerns | Major concerns | Low |
| SWLDH:TRQ | Some concerns | Low risk | No concerns | Major concerns | No concerns | Major concerns | Very low |
| SWLDH:XYP | Some concerns | Low risk | No concerns | Major concerns | No concerns | Major concerns | Very low |
| SWLDH:XZL | Some concerns | Low risk | No concerns | No concerns | Major concerns | Major concerns | Very low |
| TRQ:XYP | Some concerns | Low risk | No concerns | Major concerns | No concerns | Major concerns | Very low |
| TRQ:XZL | Some concerns | Low risk | No concerns | No concerns | No concerns | Major concerns | Low |
| XYP:XZL | Some concerns | Low risk | No concerns | No concerns | Major concerns | Major concerns | Very low |

NOTE: **SFJD**: Shufeng Jiedu Capsule; **XZL**: Fresh Bamboo Juice Oral Liquid; **QQHT**: Qingqi Huatan Pill; **QKPC**: Qingke Pingchuan Granule; **TRQ**: Tanreqing Injection; **RDN**: Reduning Injection; **SWLDH**: Shiwei Longdanhua Capsule; **QKL**: Qingkailing Injection; **XYP**: Xiyanping Injection; **CT:** Conventional biomedicine treatment.

Table S6.8: CINeMA Results of **PaCO2**

| Comparison | Within-study bias | Reporting bias | Indirectness | Imprecision | Heterogeneity | Incoherence | Confidence rating |
| --- | --- | --- | --- | --- | --- | --- | --- |
| CT:QKL | Some concerns | Low risk | No concerns | No concerns | Major concerns | Major concerns | Very low |
| CT:QKPC | Some concerns | Low risk | No concerns | Major concerns | No concerns | Major concerns | Very low |
| CT:QQHT | Some concerns | Low risk | No concerns | Major concerns | No concerns | Major concerns | Very low |
| CT:RDN | Some concerns | Low risk | No concerns | No concerns | Major concerns | Major concerns | Very low |
| CT:SFJD | Some concerns | Low risk | No concerns | Major concerns | No concerns | Major concerns | Very low |
| CT:SWLDH | Some concerns | Low risk | No concerns | Major concerns | No concerns | Major concerns | Very low |
| CT:TRQ | Some concerns | Low risk | No concerns | No concerns | Major concerns | Major concerns | Very low |
| CT:XYP | Some concerns | Low risk | No concerns | Major concerns | No concerns | Major concerns | Very low |
| CT:XZL | Some concerns | Low risk | No concerns | No concerns | Major concerns | Major concerns | Very low |
| QKL:QKPC | Some concerns | Low risk | No concerns | Major concerns | No concerns | Major concerns | Very low |
| QKL:QQHT | Some concerns | Low risk | No concerns | Major concerns | No concerns | Major concerns | Very low |
| QKL:RDN | Some concerns | Low risk | No concerns | Major concerns | No concerns | Major concerns | Very low |
| QKL:SFJD | Some concerns | Low risk | No concerns | Major concerns | No concerns | Major concerns | Very low |
| QKL:SWLDH | Some concerns | Low risk | No concerns | Major concerns | No concerns | Major concerns | Very low |
| QKL:TRQ | Some concerns | Low risk | No concerns | Major concerns | No concerns | Major concerns | Very low |
| QKL:XYP | Some concerns | Low risk | No concerns | Major concerns | No concerns | Major concerns | Very low |
| QKL:XZL | Some concerns | Low risk | No concerns | Major concerns | No concerns | Major concerns | Very low |
| QKPC:QQHT | Some concerns | Low risk | No concerns | Major concerns | No concerns | Major concerns | Very low |
| QKPC:RDN | Some concerns | Low risk | No concerns | Major concerns | No concerns | Major concerns | Very low |
| QKPC:SFJD | Some concerns | Low risk | No concerns | Major concerns | No concerns | Major concerns | Very low |
| QKPC:SWLDH | Some concerns | Low risk | No concerns | Major concerns | No concerns | Major concerns | Very low |
| QKPC:TRQ | Some concerns | Low risk | No concerns | Major concerns | No concerns | Major concerns | Very low |
| QKPC:XYP | Some concerns | Low risk | No concerns | Major concerns | No concerns | Major concerns | Very low |
| QKPC:XZL | Some concerns | Low risk | No concerns | Major concerns | No concerns | Major concerns | Very low |
| QQHT:RDN | Some concerns | Low risk | No concerns | Major concerns | No concerns | Major concerns | Very low |
| QQHT:SFJD | Some concerns | Low risk | No concerns | Major concerns | No concerns | Major concerns | Very low |
| QQHT:SWLDH | Some concerns | Low risk | No concerns | Major concerns | No concerns | Major concerns | Very low |
| QQHT:TRQ | Some concerns | Low risk | No concerns | Major concerns | No concerns | Major concerns | Very low |
| QQHT:XYP | Some concerns | Low risk | No concerns | Major concerns | No concerns | Major concerns | Very low |
| QQHT:XZL | Some concerns | Low risk | No concerns | Major concerns | No concerns | Major concerns | Very low |
| RDN:SFJD | Some concerns | Low risk | No concerns | Major concerns | No concerns | Major concerns | Very low |
| RDN:SWLDH | Some concerns | Low risk | No concerns | Major concerns | No concerns | Major concerns | Very low |
| RDN:TRQ | Some concerns | Low risk | No concerns | Major concerns | No concerns | Major concerns | Very low |
| RDN:XYP | Some concerns | Low risk | No concerns | Major concerns | No concerns | Major concerns | Very low |
| RDN:XZL | Some concerns | Low risk | No concerns | Major concerns | No concerns | Major concerns | Very low |
| SFJD:SWLDH | Some concerns | Low risk | No concerns | Major concerns | No concerns | Major concerns | Very low |
| SFJD:TRQ | Some concerns | Low risk | No concerns | Major concerns | No concerns | Major concerns | Very low |
| SFJD:XYP | Some concerns | Low risk | No concerns | Major concerns | No concerns | Major concerns | Very low |
| SFJD:XZL | Some concerns | Low risk | No concerns | Major concerns | No concerns | Major concerns | Very low |
| SWLDH:TRQ | Some concerns | Low risk | No concerns | Major concerns | No concerns | Major concerns | Very low |
| SWLDH:XYP | Some concerns | Low risk | No concerns | Major concerns | No concerns | Major concerns | Very low |
| SWLDH:XZL | Some concerns | Low risk | No concerns | Major concerns | No concerns | Major concerns | Very low |
| TRQ:XYP | Some concerns | Low risk | No concerns | Major concerns | No concerns | Major concerns | Very low |
| TRQ:XZL | Some concerns | Low risk | No concerns | Major concerns | No concerns | Major concerns | Very low |
| XYP:XZL | Some concerns | Low risk | No concerns | Major concerns | No concerns | Major concerns | Very low |

NOTE: **SFJD**: Shufeng Jiedu Capsule; **XZL**: Fresh Bamboo Juice Oral Liquid; **QQHT**: Qingqi Huatan Pill; **QKPC**: Qingke Pingchuan Granule; **TRQ**: Tanreqing Injection; **RDN**: Reduning Injection; **SWLDH**: Shiwei Longdanhua Capsule; **QKL**: Qingkailing Injection; **XYP**: Xiyanping Injection; **CT:** Conventional biomedicine treatment.

Table S6.9: CINeMA Results of **IL-6**

| Comparison | Within-study bias | Reporting bias | Indirectness | Imprecision | Heterogeneity | Incoherence | Confidence rating |
| --- | --- | --- | --- | --- | --- | --- | --- |
| CT:QKPC | Some concerns | Low risk | No concerns | Major concerns | No concerns | Major concerns | Very low |
| CT:QQHT | Some concerns | Low risk | No concerns | Major concerns | No concerns | Major concerns | Very low |
| CT:RDN | Some concerns | Low risk | No concerns | Major concerns | No concerns | Major concerns | Very low |
| CT:SFJD | Some concerns | Low risk | No concerns | Major concerns | No concerns | Major concerns | Very low |
| CT:SWLDH | Some concerns | Low risk | No concerns | No concerns | No concerns | Major concerns | Low |
| CT:TRQ | Some concerns | Low risk | No concerns | Major concerns | No concerns | Major concerns | Very low |
| CT:XYP | Major concerns | Low risk | No concerns | Major concerns | No concerns | Major concerns | Very low |
| CT:XZL | Some concerns | Low risk | No concerns | No concerns | No concerns | Major concerns | Low |
| CT:ZCL | Some concerns | Low risk | No concerns | Major concerns | No concerns | Major concerns | Very low |
| QKPC:QQHT | Some concerns | Low risk | No concerns | Major concerns | No concerns | Major concerns | Very low |
| QKPC:RDN | Some concerns | Low risk | No concerns | Major concerns | No concerns | Major concerns | Very low |
| QKPC:SFJD | Some concerns | Low risk | No concerns | Major concerns | No concerns | Major concerns | Very low |
| QKPC:SWLDH | Some concerns | Low risk | No concerns | No concerns | Major concerns | Major concerns | Very low |
| QKPC:TRQ | Some concerns | Low risk | No concerns | Major concerns | No concerns | Major concerns | Very low |
| QKPC:XYP | Some concerns | Low risk | No concerns | Major concerns | No concerns | Major concerns | Very low |
| QKPC:XZL | Some concerns | Low risk | No concerns | No concerns | Major concerns | Major concerns | Very low |
| QKPC:ZCL | Some concerns | Low risk | No concerns | Major concerns | No concerns | Major concerns | Very low |
| QQHT:RDN | Some concerns | Low risk | No concerns | Major concerns | No concerns | Major concerns | Very low |
| QQHT:SFJD | Some concerns | Low risk | No concerns | Major concerns | No concerns | Major concerns | Very low |
| QQHT:SWLDH | Some concerns | Low risk | No concerns | No concerns | Major concerns | Major concerns | Very low |
| QQHT:TRQ | Some concerns | Low risk | No concerns | Major concerns | No concerns | Major concerns | Very low |
| QQHT:XYP | Some concerns | Low risk | No concerns | Major concerns | No concerns | Major concerns | Very low |
| QQHT:XZL | Some concerns | Low risk | No concerns | No concerns | No concerns | Major concerns | Low |
| QQHT:ZCL | Some concerns | Low risk | No concerns | Major concerns | No concerns | Major concerns | Very low |
| RDN:SFJD | Some concerns | Low risk | No concerns | Major concerns | No concerns | Major concerns | Very low |
| RDN:SWLDH | Some concerns | Low risk | No concerns | No concerns | Major concerns | Major concerns | Very low |
| RDN:TRQ | Some concerns | Low risk | No concerns | Major concerns | No concerns | Major concerns | Very low |
| RDN:XYP | Some concerns | Low risk | No concerns | Major concerns | No concerns | Major concerns | Very low |
| RDN:XZL | Some concerns | Low risk | No concerns | No concerns | No concerns | Major concerns | Low |
| RDN:ZCL | Some concerns | Low risk | No concerns | Major concerns | No concerns | Major concerns | Very low |
| SFJD:SWLDH | Some concerns | Low risk | No concerns | No concerns | Major concerns | Major concerns | Very low |
| SFJD:TRQ | Some concerns | Low risk | No concerns | Major concerns | No concerns | Major concerns | Very low |
| SFJD:XYP | Some concerns | Low risk | No concerns | Major concerns | No concerns | Major concerns | Very low |
| SFJD:XZL | Some concerns | Low risk | No concerns | No concerns | Major concerns | Major concerns | Very low |
| SFJD:ZCL | Some concerns | Low risk | No concerns | Major concerns | No concerns | Major concerns | Very low |
| SWLDH:TRQ | Some concerns | Low risk | No concerns | Major concerns | No concerns | Major concerns | Very low |
| SWLDH:XYP | Some concerns | Low risk | No concerns | No concerns | Major concerns | Major concerns | Very low |
| SWLDH:XZL | Some concerns | Low risk | No concerns | Major concerns | No concerns | Major concerns | Very low |
| SWLDH:ZCL | Some concerns | Low risk | No concerns | No concerns | Major concerns | Major concerns | Very low |
| TRQ:XYP | Some concerns | Low risk | No concerns | Major concerns | No concerns | Major concerns | Very low |
| TRQ:XZL | Some concerns | Low risk | No concerns | No concerns | Major concerns | Major concerns | Very low |
| TRQ:ZCL | Some concerns | Low risk | No concerns | Major concerns | No concerns | Major concerns | Very low |
| XYP:XZL | Some concerns | Low risk | No concerns | No concerns | Major concerns | Major concerns | Very low |
| XYP:ZCL | Some concerns | Low risk | No concerns | Major concerns | No concerns | Major concerns | Very low |
| XZL:ZCL | Some concerns | Low risk | No concerns | No concerns | No concerns | Major concerns | Low |

NOTE: **SFJD**: Shufeng Jiedu Capsule; **XZL**: Fresh Bamboo Juice Oral Liquid; **QQHT**: Qingqi Huatan Pill; **QKPC**: Qingke Pingchuan Granule; **TRQ**: Tanreqing Injection; **RDN**: Reduning Injection; **SWLDH**: Shiwei Longdanhua Capsule; **ZCL**: Zhichuanling Injection; **XYP**: Xiyanping Injection; **CT:** Conventional biomedicine treatment.

Table S6.10: CINeMA Results of **IL-8**

| Comparison | Within-study bias | Reporting bias | Indirectness | Imprecision | Heterogeneity | Incoherence | Confidence rating |
| --- | --- | --- | --- | --- | --- | --- | --- |
| CT:QKL | Some concerns | Low risk | No concerns | No concerns | Major concerns | Major concerns | Very low |
| CT:QQHT | Some concerns | Low risk | No concerns | Major concerns | No concerns | Major concerns | Very low |
| CT:RDN | Some concerns | Low risk | No concerns | No concerns | Major concerns | Major concerns | Very low |
| CT:SFJD | Some concerns | Low risk | No concerns | No concerns | Major concerns | Major concerns | Very low |
| CT:XZL | Some concerns | Low risk | No concerns | Major concerns | No concerns | Major concerns | Very low |
| QKL:QQHT | Some concerns | Low risk | No concerns | Major concerns | No concerns | Major concerns | Very low |
| QKL:RDN | Some concerns | Low risk | No concerns | Major concerns | No concerns | Major concerns | Very low |
| QKL:SFJD | Some concerns | Low risk | No concerns | Major concerns | No concerns | Major concerns | Very low |
| QKL:XZL | Some concerns | Low risk | No concerns | Major concerns | No concerns | Major concerns | Very low |
| QQHT:RDN | Some concerns | Low risk | No concerns | Major concerns | No concerns | Major concerns | Very low |
| QQHT:SFJD | Some concerns | Low risk | No concerns | Major concerns | No concerns | Major concerns | Very low |
| QQHT:XZL | Some concerns | Low risk | No concerns | Major concerns | No concerns | Major concerns | Very low |
| RDN:SFJD | Some concerns | Low risk | No concerns | Major concerns | No concerns | Major concerns | Very low |
| RDN:XZL | Some concerns | Low risk | No concerns | Major concerns | No concerns | Major concerns | Very low |
| SFJD:XZL | Some concerns | Low risk | No concerns | Major concerns | No concerns | Major concerns | Very low |

NOTE: **QKL**: Qingkailing Injection; **QQHT**: Qingqi Huatan Pill; **RDN**: Reduning Injection; **SFJD**: Shufeng Jiedu Capsule; **XZL**: Fresh Bamboo Juice Oral Liquid; **CT:** Conventional biomedicine treatment.

Table S6.11: CINeMA Results of **TNF-α**

| Comparison | Within-study bias | Reporting bias | Indirectness | Imprecision | Heterogeneity | Incoherence | Confidence rating |
| --- | --- | --- | --- | --- | --- | --- | --- |
| CT:QKL | Some concerns | Low risk | No concerns | Major concerns | No concerns | Major concerns | Very low |
| CT:QQHT | Some concerns | Low risk | No concerns | No concerns | No concerns | Major concerns | Low |
| CT:RDN | Some concerns | Low risk | No concerns | Major concerns | No concerns | Major concerns | Very low |
| CT:SFJD | Some concerns | Low risk | No concerns | No concerns | No concerns | Major concerns | Low |
| CT:SWLDH | Some concerns | Low risk | No concerns | No concerns | No concerns | Major concerns | Low |
| CT:TRQ | Some concerns | Low risk | No concerns | No concerns | No concerns | Major concerns | Low |
| CT:XZL | Some concerns | Low risk | No concerns | No concerns | No concerns | Major concerns | Low |
| QKL:QQHT | Some concerns | Low risk | No concerns | No concerns | No concerns | Major concerns | Low |
| QKL:RDN | Some concerns | Low risk | No concerns | Major concerns | No concerns | Major concerns | Very low |
| QKL:SFJD | Some concerns | Low risk | No concerns | No concerns | No concerns | Major concerns | Low |
| QKL:SWLDH | Some concerns | Low risk | No concerns | No concerns | No concerns | Major concerns | Low |
| QKL:TRQ | Some concerns | Low risk | No concerns | No concerns | No concerns | Major concerns | Low |
| QKL:XZL | Some concerns | Low risk | No concerns | No concerns | Major concerns | Major concerns | Very low |
| QQHT:RDN | Some concerns | Low risk | No concerns | No concerns | No concerns | Major concerns | Low |
| QQHT:SFJD | Some concerns | Low risk | No concerns | Major concerns | No concerns | Major concerns | Very low |
| QQHT:SWLDH | Some concerns | Low risk | No concerns | Major concerns | No concerns | Major concerns | Very low |
| QQHT:TRQ | Some concerns | Low risk | No concerns | Major concerns | No concerns | Major concerns | Very low |
| QQHT:XZL | Some concerns | Low risk | No concerns | No concerns | Major concerns | Major concerns | Very low |
| RDN:SFJD | Some concerns | Low risk | No concerns | No concerns | No concerns | Major concerns | Low |
| RDN:SWLDH | Some concerns | Low risk | No concerns | No concerns | No concerns | Major concerns | Low |
| RDN:TRQ | Some concerns | Low risk | No concerns | No concerns | No concerns | Major concerns | Low |
| RDN:XZL | Some concerns | Low risk | No concerns | No concerns | Major concerns | Major concerns | Very low |
| SFJD:SWLDH | Some concerns | Low risk | No concerns | Major concerns | No concerns | Major concerns | Very low |
| SFJD:TRQ | Some concerns | Low risk | No concerns | Major concerns | No concerns | Major concerns | Very low |
| SFJD:XZL | Some concerns | Low risk | No concerns | No concerns | Major concerns | Major concerns | Very low |
| SWLDH:TRQ | Some concerns | Low risk | No concerns | Major concerns | No concerns | Major concerns | Very low |
| SWLDH:XZL | Some concerns | Low risk | No concerns | Major concerns | No concerns | Major concerns | Very low |
| TRQ:XZL | Some concerns | Low risk | No concerns | No concerns | Major concerns | Major concerns | Very low |

NOTE: **SFJD**: Shufeng Jiedu Capsule; **XZL**: Fresh Bamboo Juice Oral Liquid; **QQHT**: Qingqi Huatan Pill; **TRQ**: Tanreqing Injection; **RDN**: Reduning Injection; **SWLDH**: Shiwei Longdanhua Capsule; **QKL**: Qingkailing Injection; **CT:** Conventional biomedicine treatment.

Table S6.12: CINeMA Results of **Adverse event**

| Comparison | Within-study bias | Reporting bias | Indirectness | Imprecision | Heterogeneity | Incoherence | Confidence rating |
| --- | --- | --- | --- | --- | --- | --- | --- |
| CT:QKL | Some concerns | Low risk | No concerns | Major concerns | No concerns | Major concerns | Very low |
| CT:QKPC | Some concerns | Low risk | No concerns | Major concerns | No concerns | Major concerns | Very low |
| CT:QQHT | Some concerns | Low risk | No concerns | Major concerns | No concerns | Major concerns | Very low |
| CT:RDN | Some concerns | Low risk | No concerns | Major concerns | No concerns | Major concerns | Very low |
| CT:SFJD | Some concerns | Low risk | No concerns | Major concerns | No concerns | Major concerns | Very low |
| CT:SWLDH | Some concerns | Low risk | No concerns | Major concerns | No concerns | Major concerns | Very low |
| CT:TRQ | Some concerns | Low risk | No concerns | Major concerns | No concerns | Major concerns | Very low |
| CT:XYP | Some concerns | Low risk | No concerns | Major concerns | No concerns | Major concerns | Very low |
| CT:XZL | Some concerns | Low risk | No concerns | Major concerns | No concerns | Major concerns | Very low |
| CT:ZCL | Some concerns | Low risk | No concerns | Major concerns | No concerns | Major concerns | Very low |
| QKL:QKPC | Some concerns | Low risk | No concerns | Major concerns | No concerns | Major concerns | Very low |
| QKL:QQHT | Some concerns | Low risk | No concerns | Major concerns | No concerns | Major concerns | Very low |
| QKL:RDN | Some concerns | Low risk | No concerns | Major concerns | No concerns | Major concerns | Very low |
| QKL:SFJD | Some concerns | Low risk | No concerns | Major concerns | No concerns | Major concerns | Very low |
| QKL:SWLDH | Some concerns | Low risk | No concerns | Major concerns | No concerns | Major concerns | Very low |
| QKL:TRQ | Some concerns | Low risk | No concerns | Major concerns | No concerns | Major concerns | Very low |
| QKL:XYP | Some concerns | Low risk | No concerns | Major concerns | No concerns | Major concerns | Very low |
| QKL:XZL | Some concerns | Low risk | No concerns | Major concerns | No concerns | Major concerns | Very low |
| QKL:ZCL | Some concerns | Low risk | No concerns | Major concerns | No concerns | Major concerns | Very low |
| QKPC:QQHT | Some concerns | Low risk | No concerns | Major concerns | No concerns | Major concerns | Very low |
| QKPC:RDN | Some concerns | Low risk | No concerns | Major concerns | No concerns | Major concerns | Very low |
| QKPC:SFJD | Some concerns | Low risk | No concerns | Major concerns | No concerns | Major concerns | Very low |
| QKPC:SWLDH | Some concerns | Low risk | No concerns | Major concerns | No concerns | Major concerns | Very low |
| QKPC:TRQ | Some concerns | Low risk | No concerns | Major concerns | No concerns | Major concerns | Very low |
| QKPC:XYP | Some concerns | Low risk | No concerns | Major concerns | No concerns | Major concerns | Very low |
| QKPC:XZL | Some concerns | Low risk | No concerns | Major concerns | No concerns | Major concerns | Very low |
| QKPC:ZCL | Some concerns | Low risk | No concerns | Major concerns | No concerns | Major concerns | Very low |
| QQHT:RDN | Some concerns | Low risk | No concerns | Major concerns | No concerns | Major concerns | Very low |
| QQHT:SFJD | Some concerns | Low risk | No concerns | Major concerns | No concerns | Major concerns | Very low |
| QQHT:SWLDH | Some concerns | Low risk | No concerns | Major concerns | No concerns | Major concerns | Very low |
| QQHT:TRQ | Some concerns | Low risk | No concerns | Major concerns | No concerns | Major concerns | Very low |
| QQHT:XYP | Some concerns | Low risk | No concerns | Major concerns | No concerns | Major concerns | Very low |
| QQHT:XZL | Some concerns | Low risk | No concerns | Major concerns | No concerns | Major concerns | Very low |
| QQHT:ZCL | Some concerns | Low risk | No concerns | Major concerns | No concerns | Major concerns | Very low |
| RDN:SFJD | Some concerns | Low risk | No concerns | Major concerns | No concerns | Major concerns | Very low |
| RDN:SWLDH | Some concerns | Low risk | No concerns | Major concerns | No concerns | Major concerns | Very low |
| RDN:TRQ | Some concerns | Low risk | No concerns | Major concerns | No concerns | Major concerns | Very low |
| RDN:XYP | Some concerns | Low risk | No concerns | Major concerns | No concerns | Major concerns | Very low |
| RDN:XZL | Some concerns | Low risk | No concerns | Major concerns | No concerns | Major concerns | Very low |
| RDN:ZCL | Some concerns | Low risk | No concerns | Major concerns | No concerns | Major concerns | Very low |
| SFJD:SWLDH | Some concerns | Low risk | No concerns | Major concerns | No concerns | Major concerns | Very low |
| SFJD:TRQ | Some concerns | Low risk | No concerns | Major concerns | No concerns | Major concerns | Very low |
| SFJD:XYP | Some concerns | Low risk | No concerns | Major concerns | No concerns | Major concerns | Very low |
| SFJD:XZL | Some concerns | Low risk | No concerns | Major concerns | No concerns | Major concerns | Very low |
| SFJD:ZCL | Some concerns | Low risk | No concerns | Major concerns | No concerns | Major concerns | Very low |
| SWLDH:TRQ | Some concerns | Low risk | No concerns | Major concerns | No concerns | Major concerns | Very low |
| SWLDH:XYP | Some concerns | Low risk | No concerns | Major concerns | No concerns | Major concerns | Very low |
| SWLDH:XZL | Some concerns | Low risk | No concerns | Major concerns | No concerns | Major concerns | Very low |
| SWLDH:ZCL | Some concerns | Low risk | No concerns | Major concerns | No concerns | Major concerns | Very low |
| TRQ:XYP | Some concerns | Low risk | No concerns | Major concerns | No concerns | Major concerns | Very low |
| TRQ:XZL | Some concerns | Low risk | No concerns | Major concerns | No concerns | Major concerns | Very low |
| TRQ:ZCL | Some concerns | Low risk | No concerns | Major concerns | No concerns | Major concerns | Very low |
| XYP:XZL | Some concerns | Low risk | No concerns | Major concerns | No concerns | Major concerns | Very low |
| XYP:ZCL | Some concerns | Low risk | No concerns | Major concerns | No concerns | Major concerns | Very low |
| XZL:ZCL | Some concerns | Low risk | No concerns | Major concerns | No concerns | Major concerns | Very low |

NOTE: **SFJD**: Shufeng Jiedu Capsule; **XZL**: Fresh Bamboo Juice Oral Liquid; **QQHT**: Qingqi Huatan Pill; **QKPC**: Qingke Pingchuan Granule; **TRQ**: Tanreqing Injection; **RDN**: Reduning Injection; **SWLDH**: Shiwei Longdanhua Capsule; **ZCL**: Zhichuanling Injection; **QKL**: Qingkailing Injection; **XYP**: Xiyanping Injection; **CT:** Conventional biomedicine treatment.

**Appendix 7：Funnel plot of each outcome**

The figures show the assessment of small study effect bias in studies on the effects of various Chinese patent medicines on various measures of AECOPD. The funnel plots pertain to all trials comparing at least one Chinese patent medicine versus conventional biomedicine treatment.

Figure S7.1: Funnel plot of **total effective rate**


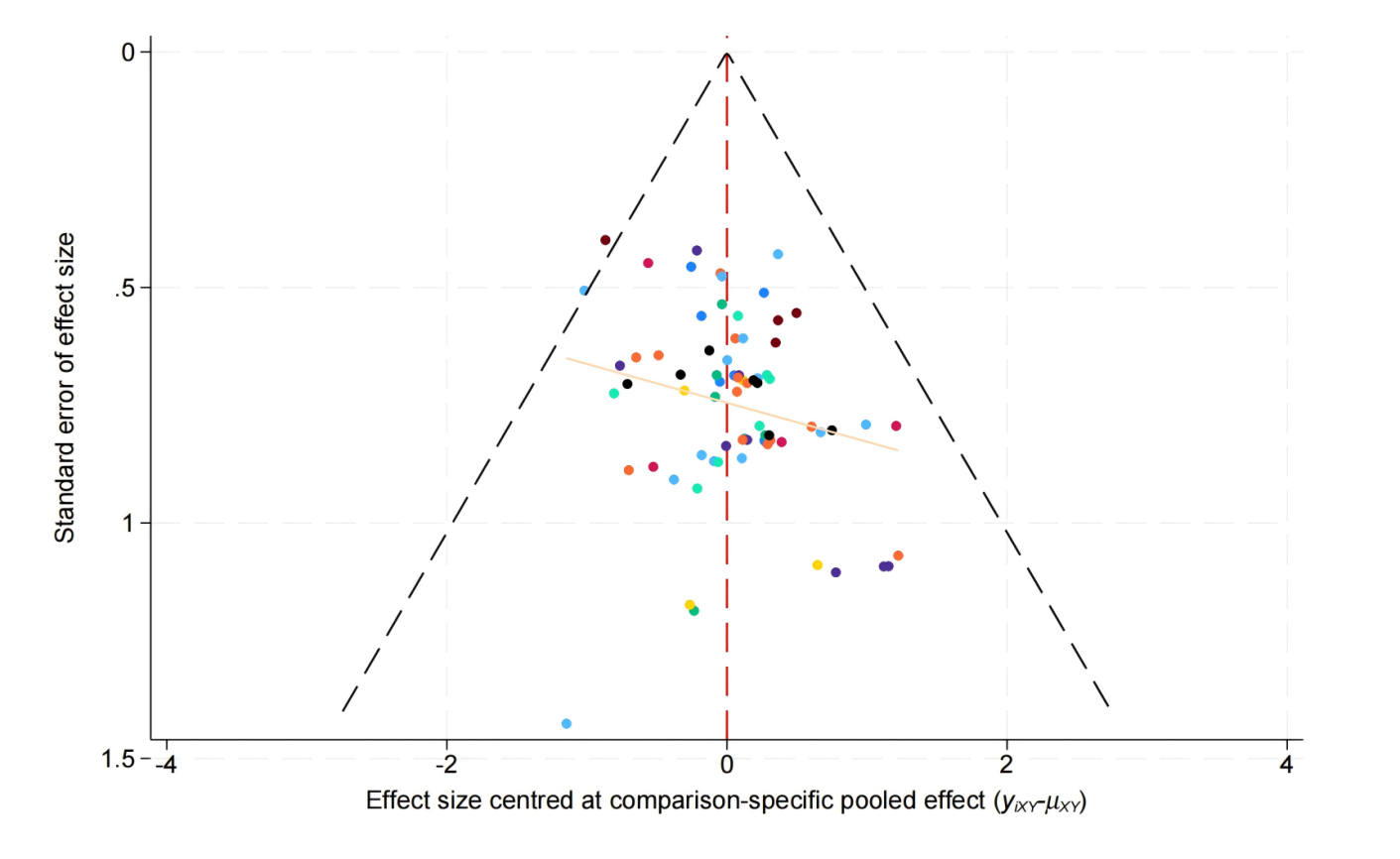


Figure S7.2: Funnel plot of **FVC**


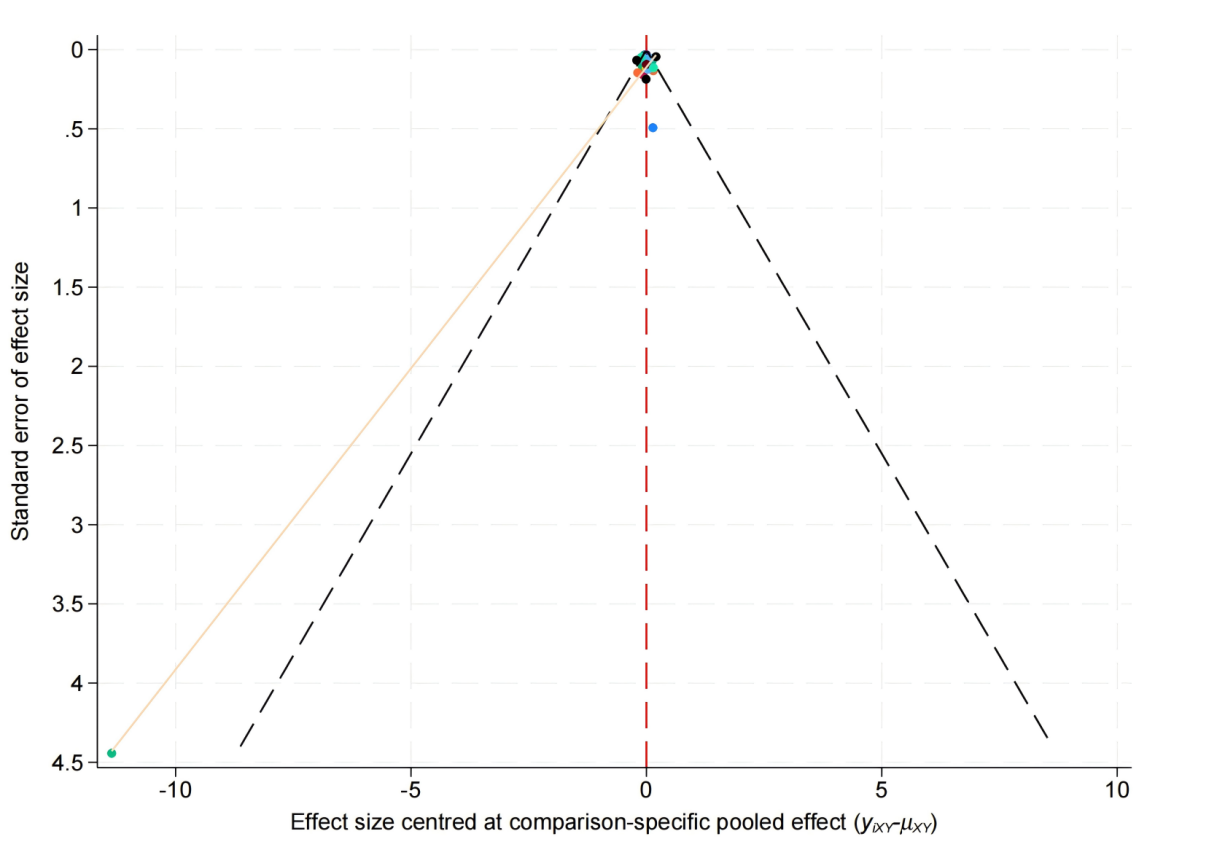


Figure S7.3: Funnel plot of **FEV1**


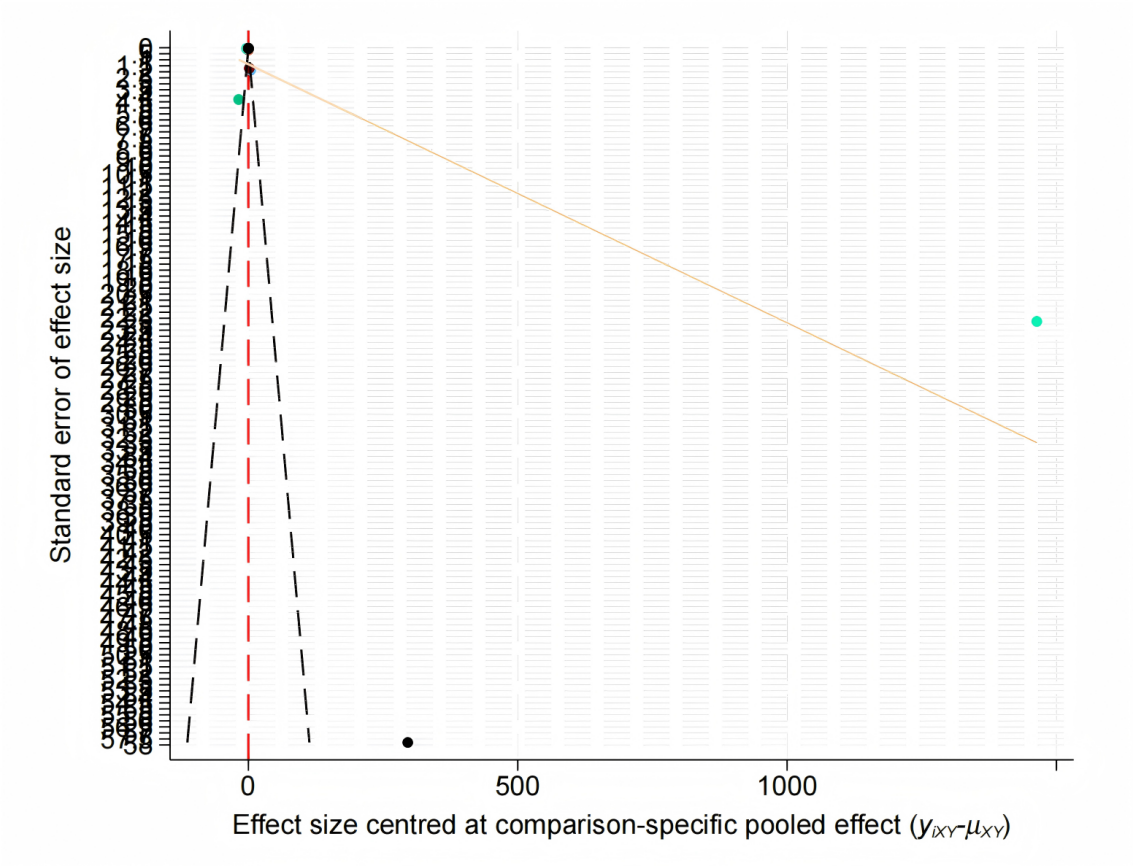


Figure S7.4: Funnel plot of **FEV1/FVC**

**
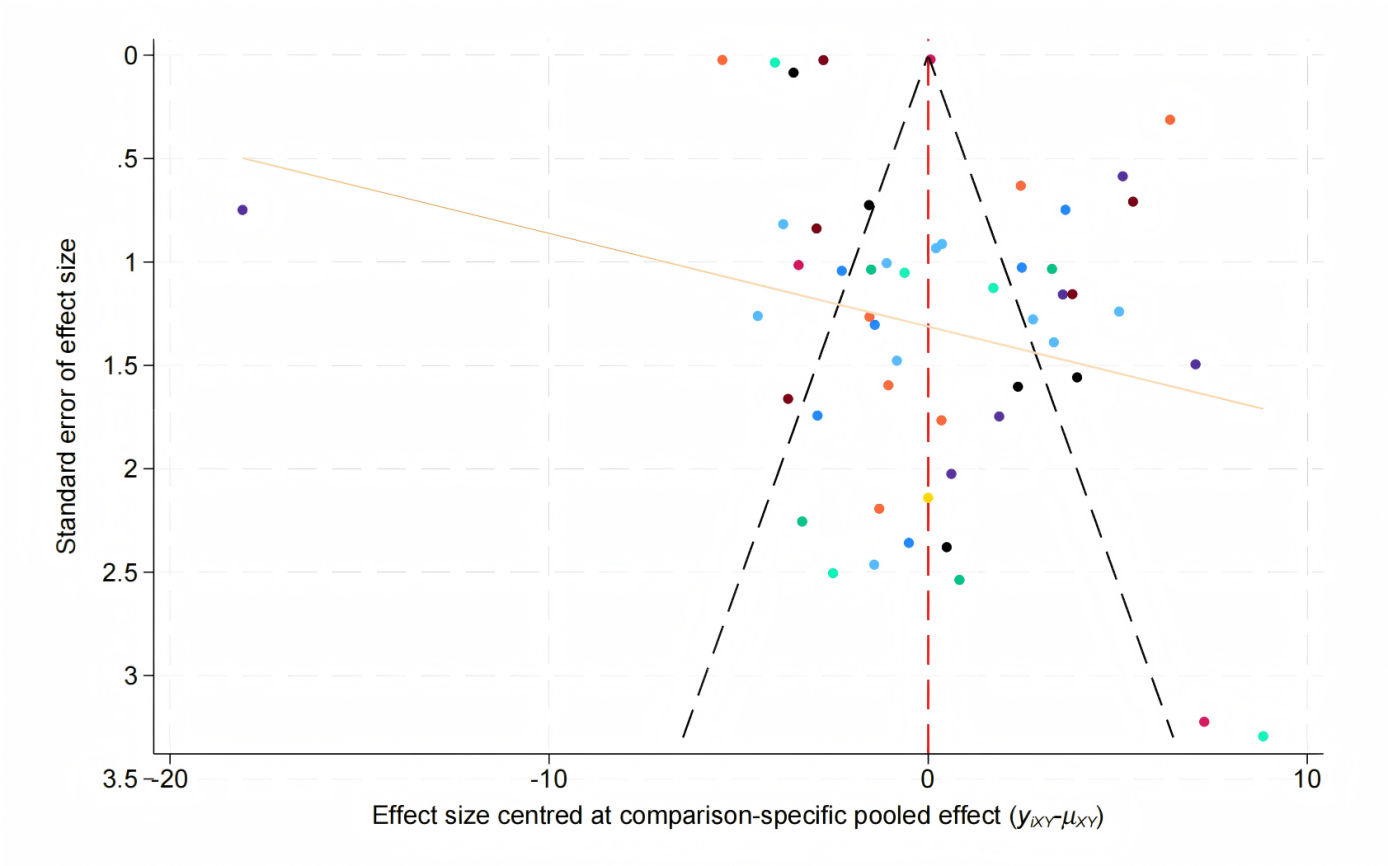
**

Figure S7.5: Funnel plot of **PH**

**
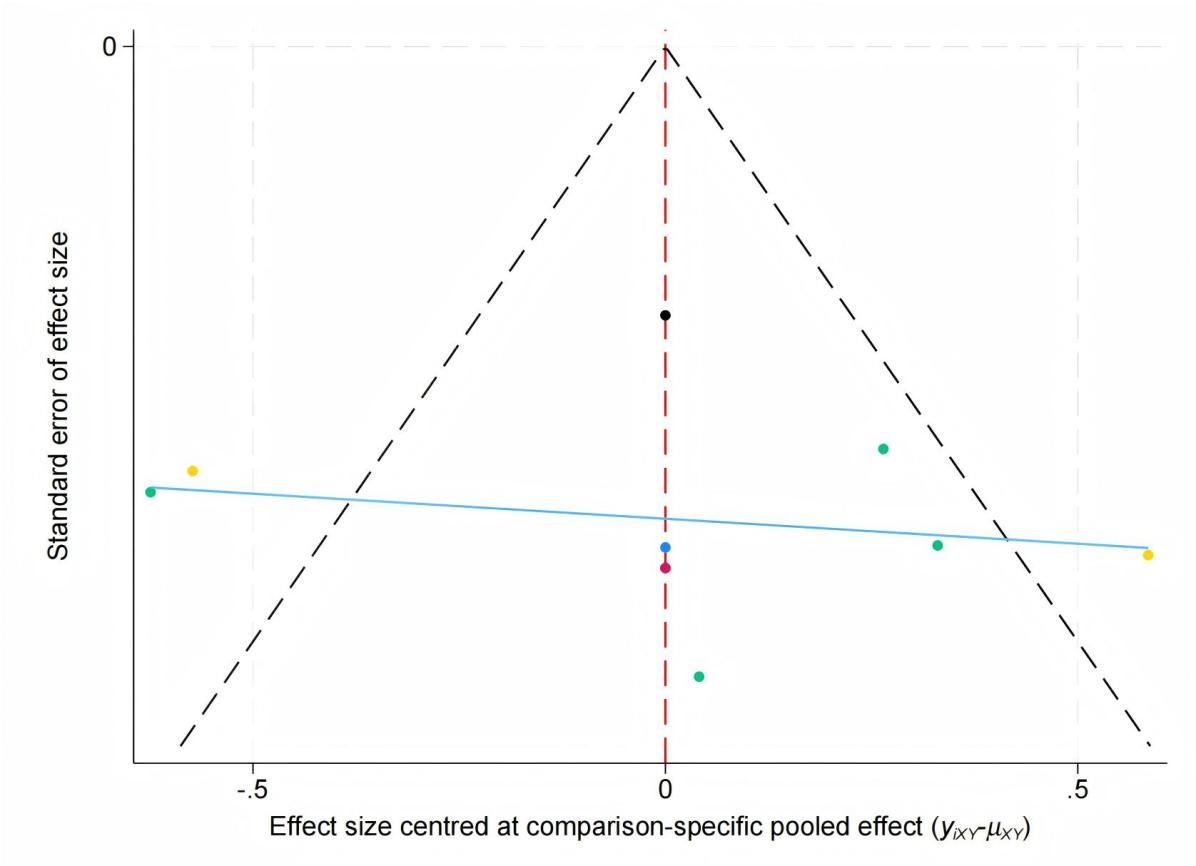
**

Figure S7.6: Funnel plot of **PaO2**

**
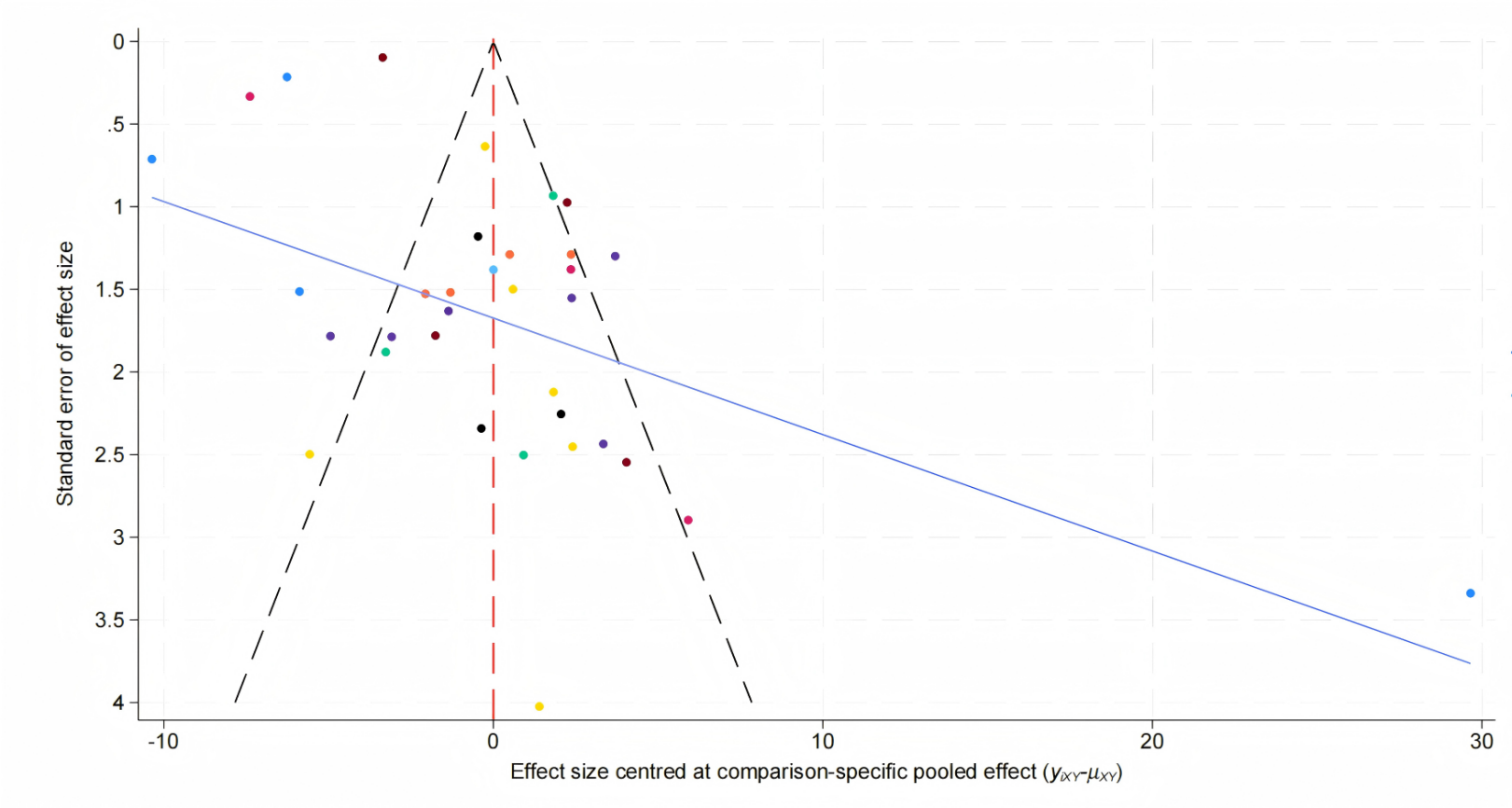
**

Figure S7.7: Funnel plot of **PaCO2**

**
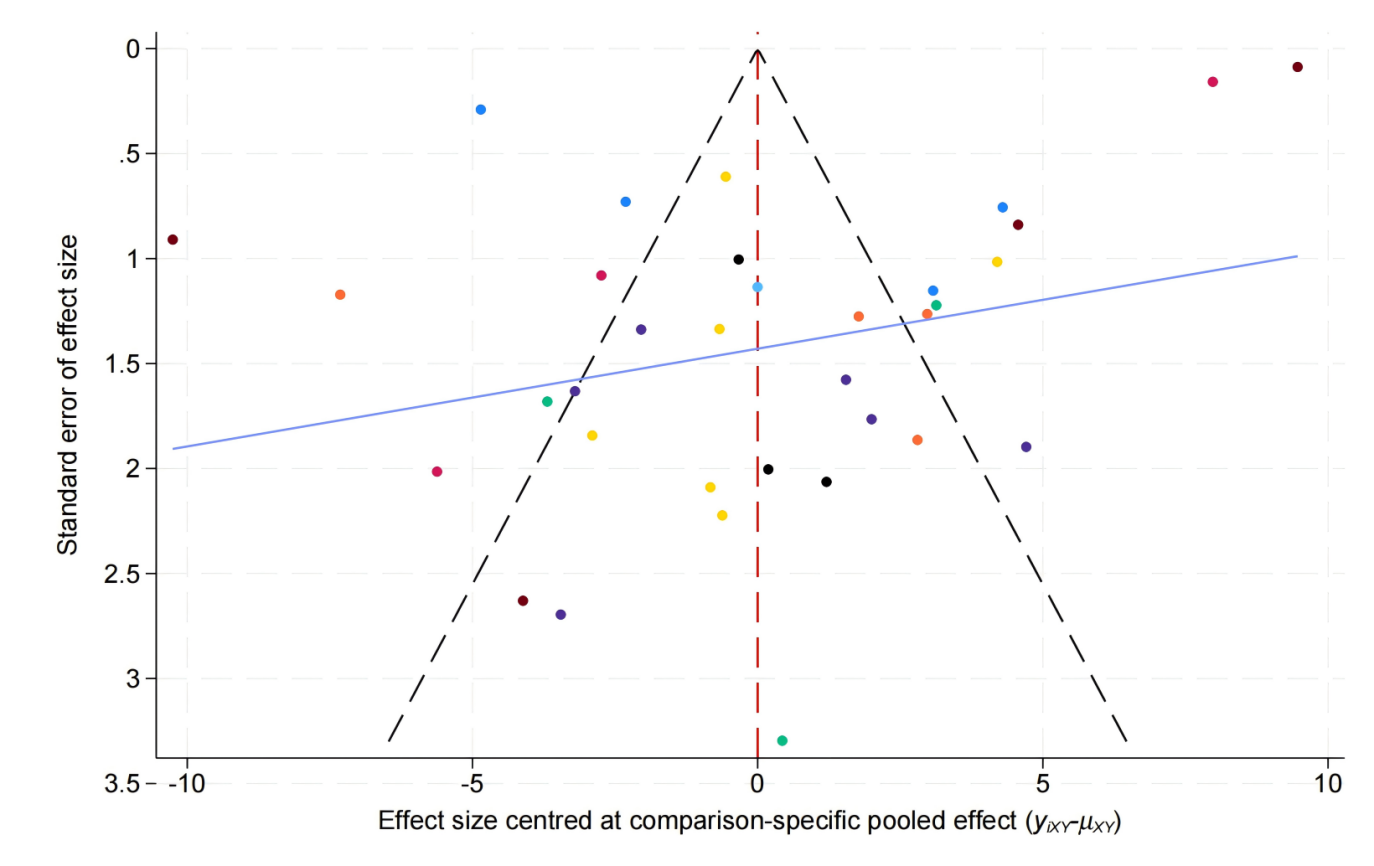
**

Figure S7.8: Funnel plot of **IL-6**


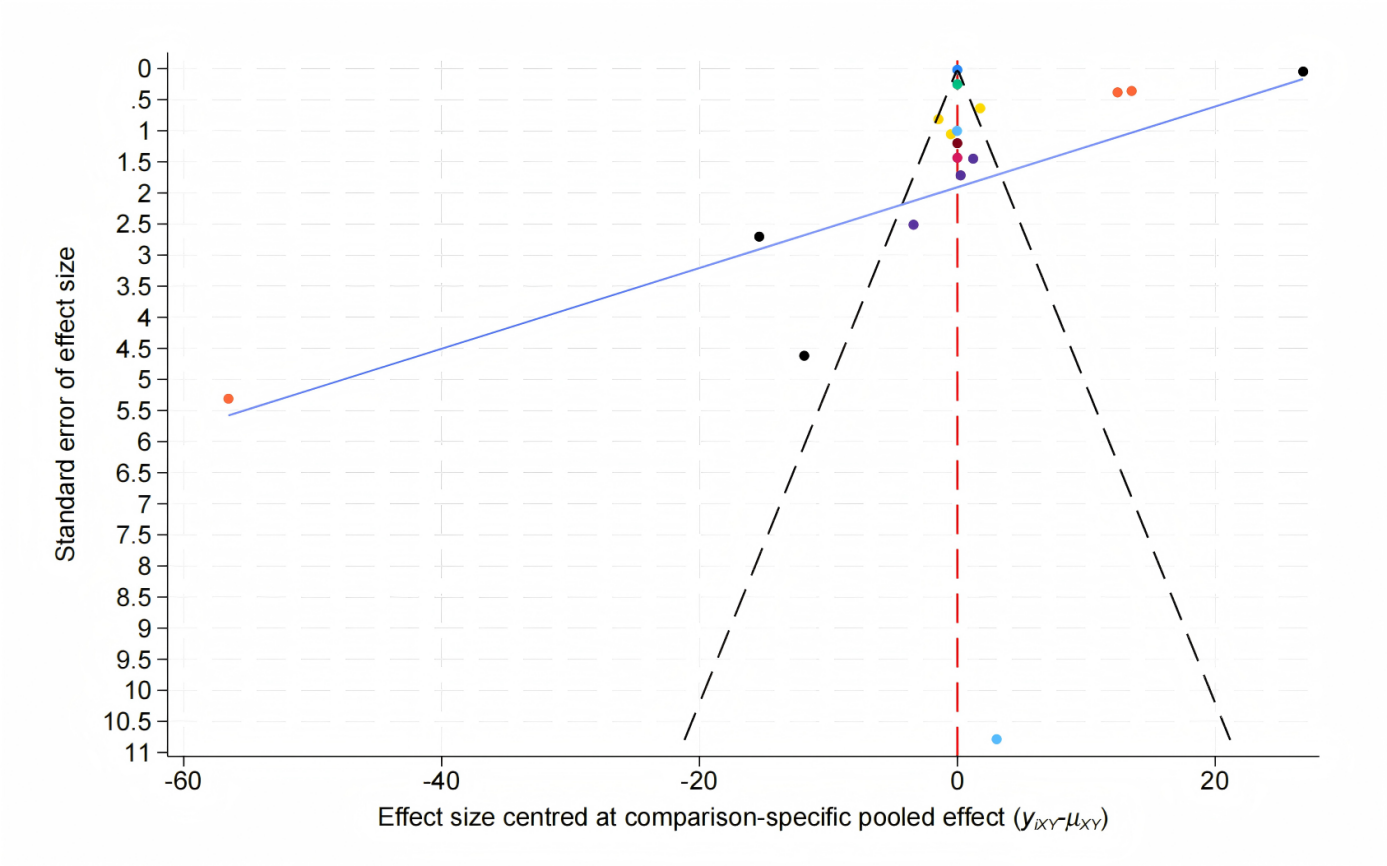


Figure S7.9: Funnel plot of **IL-8**

**
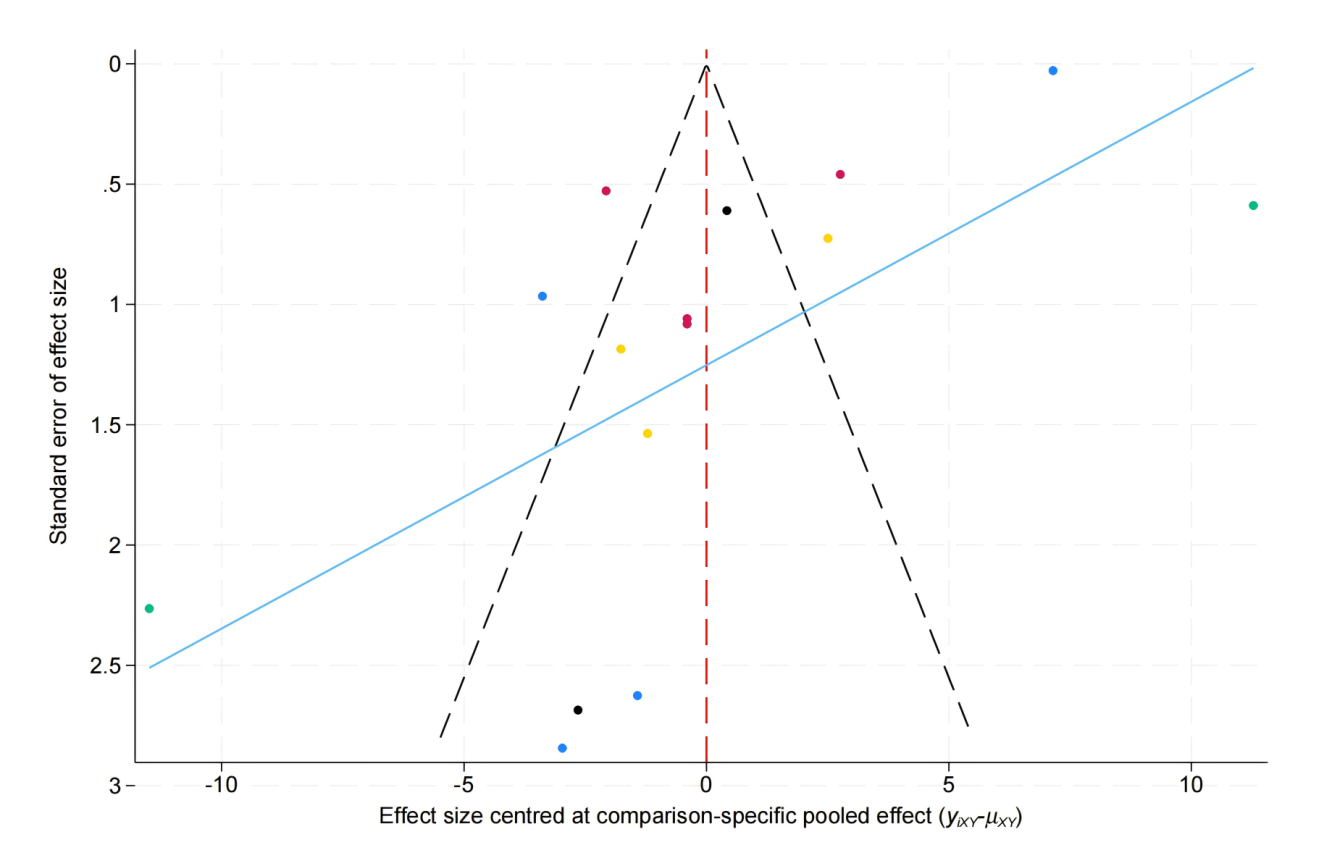
**

Figure S7.10: Funnel plot of **TNF-α**

**
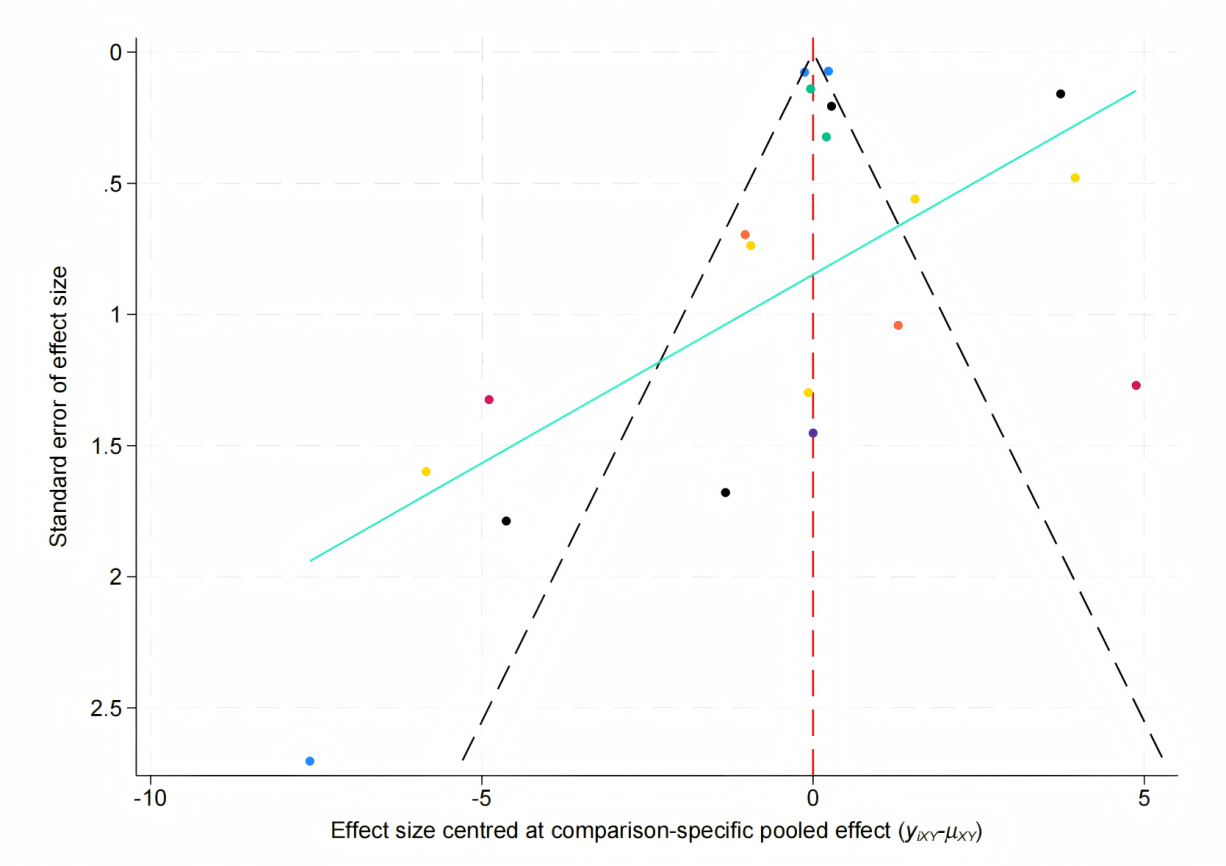
**

Figure S7.11: Funnel plot of **Adverse event**

**
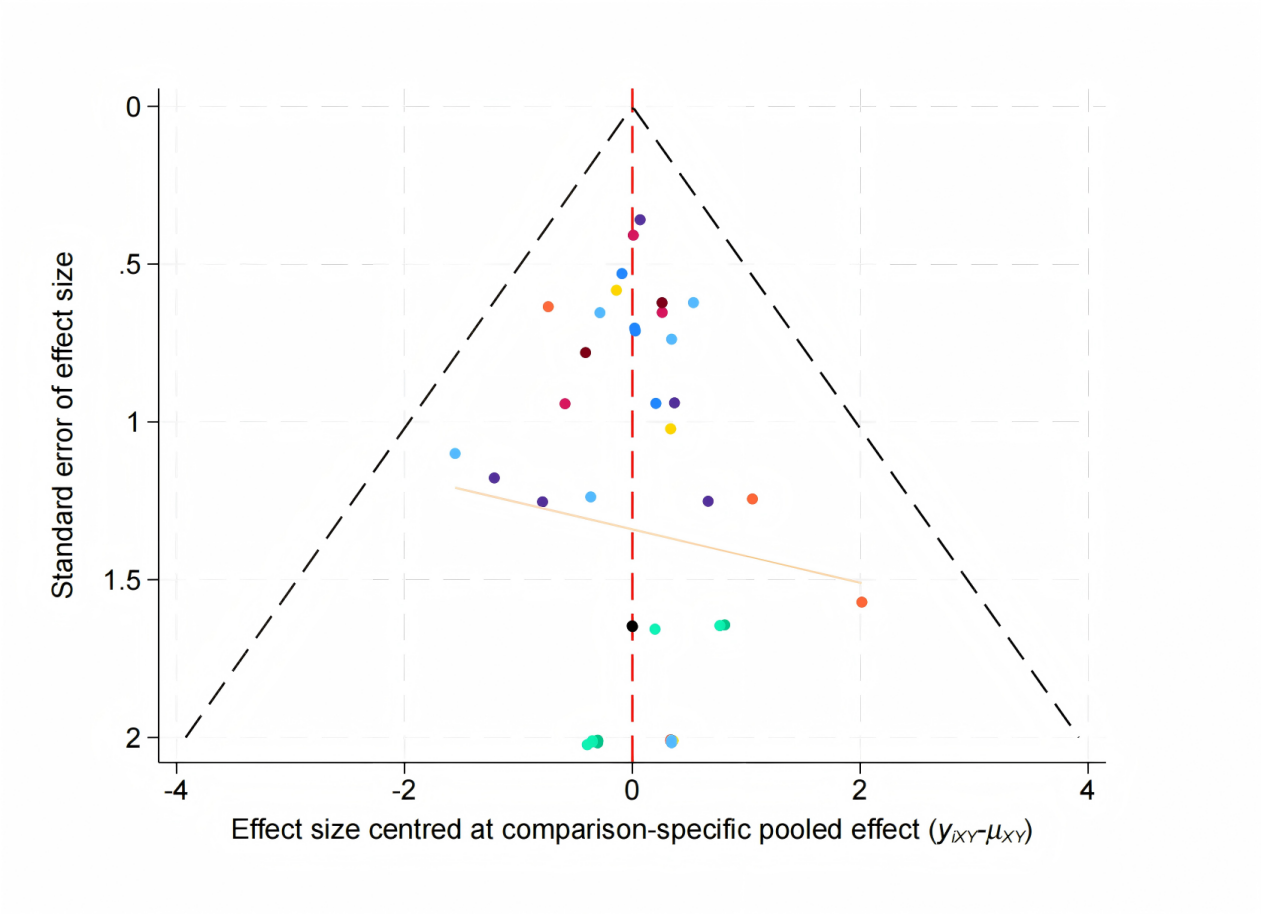
**

**Appendix 8: SUCRA and cumulative probability plots**

Figure S8.1: Cumulative ranking curve plots of CPMs for **Total effective rate** in range network.

Higher surface under the curve reflects higher probability of association with Total effective rate.


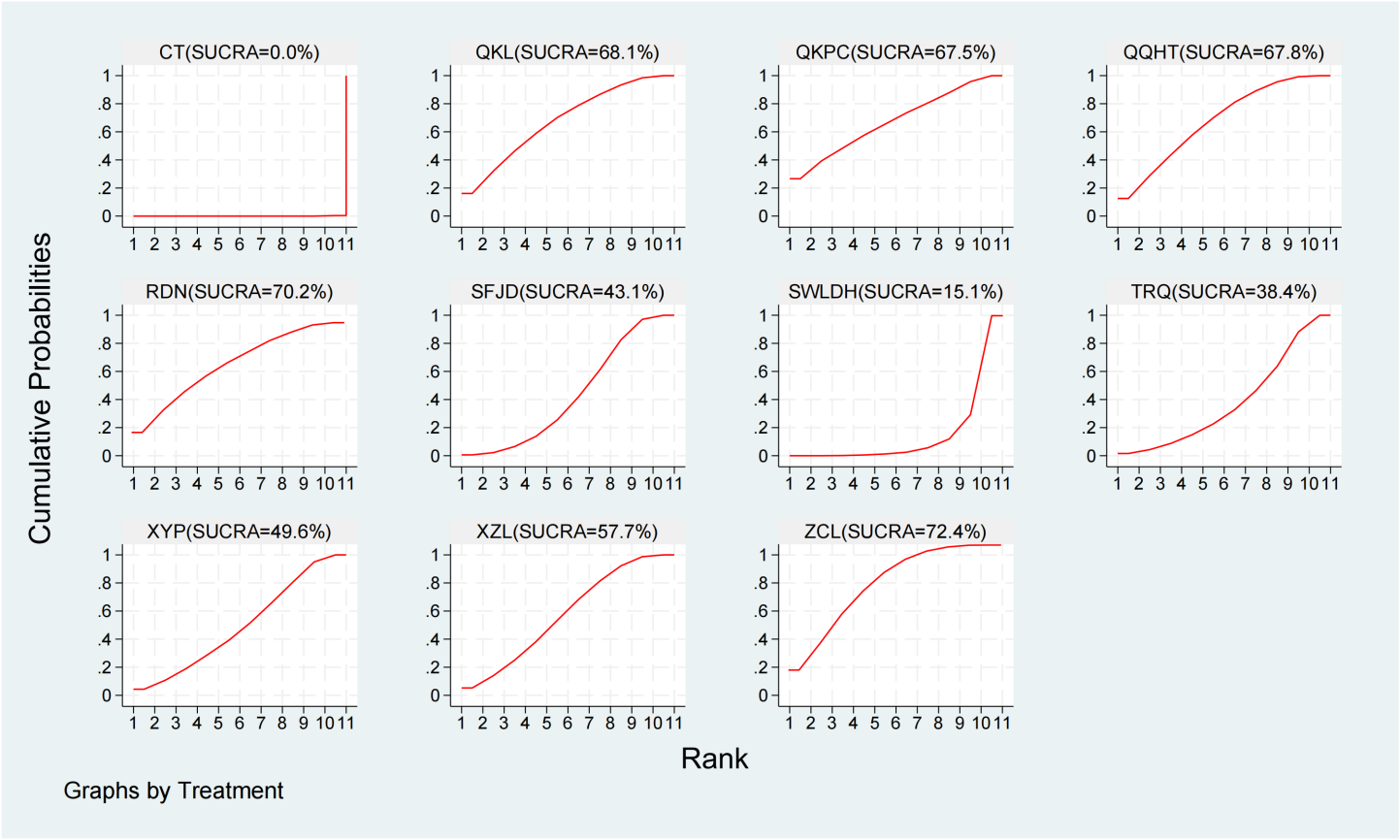


NOTE: **SFJD**: Shufeng Jiedu Capsule; **XZL**: Fresh Bamboo Juice Oral Liquid; **QQHT**: Qingqi Huatan Pill; **QKPC**: Qingke Pingchuan Granule; **TRQ**: Tanreqing Injection; **RDN**: Reduning Injection; **SWLDH**: Shiwei Longdanhua Capsule;

**ZCL**: Zhichuanling Injection; **QKL**: Qingkailing Injection; **XYP**: Xiyanping Injection; **CT**: Conventional biomedicine treatment.

Figure S8.2: Cumulative ranking curve plots of CPMs for **FVC** in range network. Higher surface under the curve reflects higher probability of association with FVC.


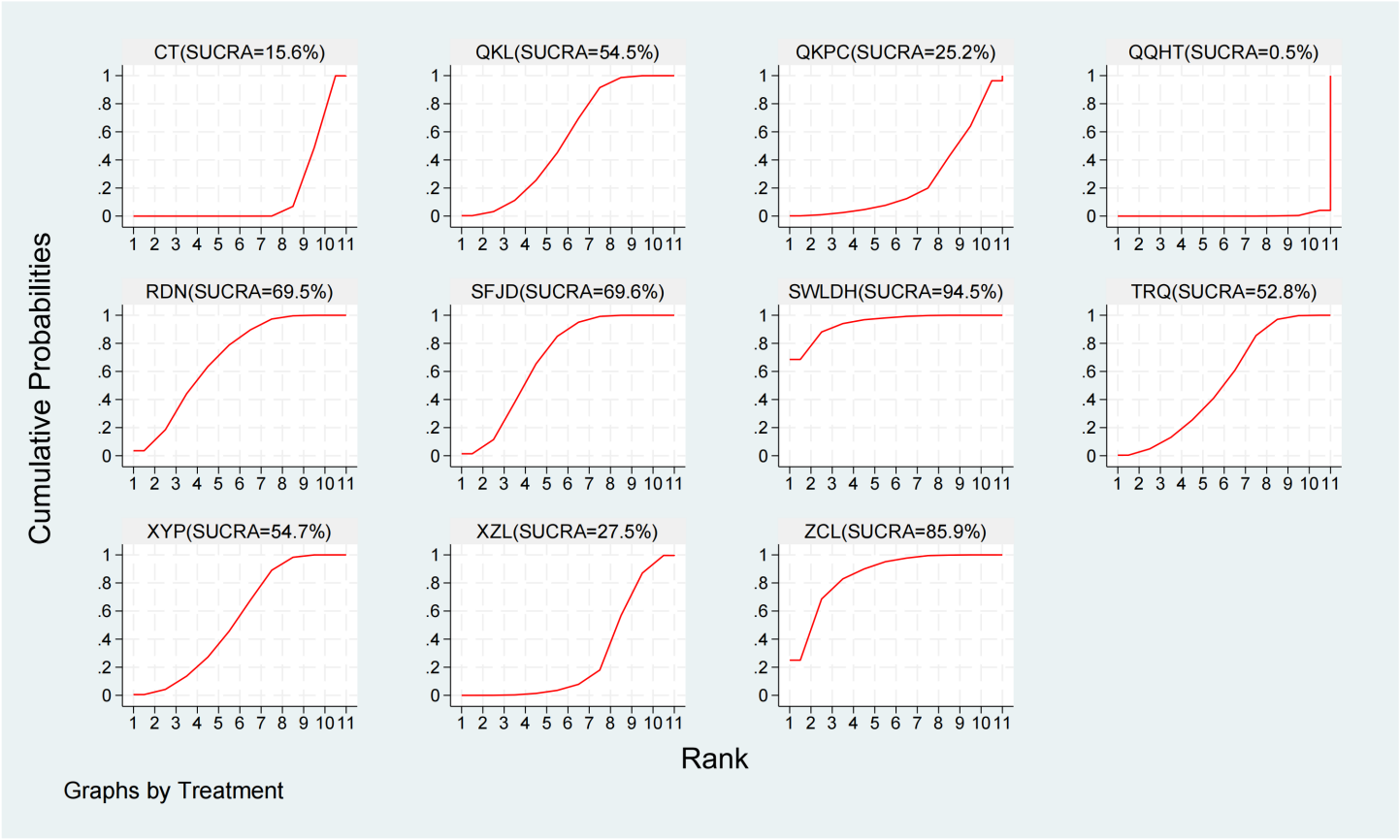


NOTE: **SFJD**: Shufeng Jiedu Capsule; **XZL**: Fresh Bamboo Juice Oral Liquid; **QQHT**: Qingqi Huatan Pill; **QKPC**: Qingke Pingchuan Granule; **TRQ**: Tanreqing Injection; **RDN**: Reduning Injection; **SWLDH**: Shiwei Longdanhua Capsule;

**ZCL**: Zhichuanling Injection; **QKL**: Qingkailing Injection; **XYP**: Xiyanping Injection; **CT**: Conventional biomedicine treatment.

Figure S8.3: Cumulative ranking curve plots of CPMs for **FEV1** in range network. Higher surface under the curve reflects higher probability of association with FEV1.

**
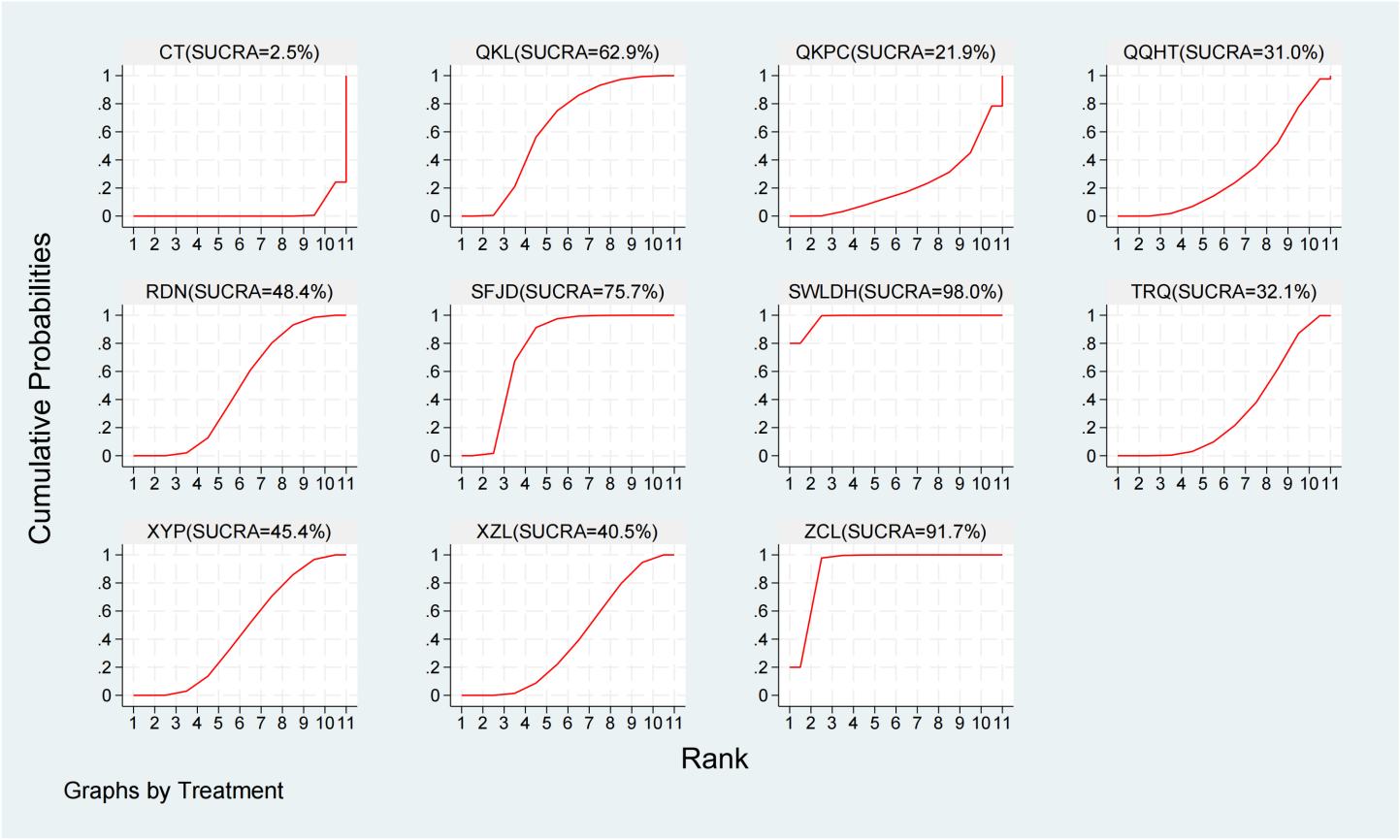
**

NOTE: **SFJD**: Shufeng Jiedu Capsule; **XZL**: Fresh Bamboo Juice Oral Liquid; **QQHT**: Qingqi Huatan Pill; **QKPC**: Qingke Pingchuan Granule; **TRQ**: Tanreqing Injection; **RDN**: Reduning Injection; **SWLDH**: Shiwei Longdanhua Capsule;

**ZCL**: Zhichuanling Injection; **QKL**: Qingkailing Injection; **XYP**: Xiyanping Injection; **CT**: Conventional biomedicine treatment.

Figure S8.4: Cumulative ranking curve plots of CPMs for **FEV1/FVC** in range network. Higher surface under the curve reflects higher probability of association with FEV1/FVC.


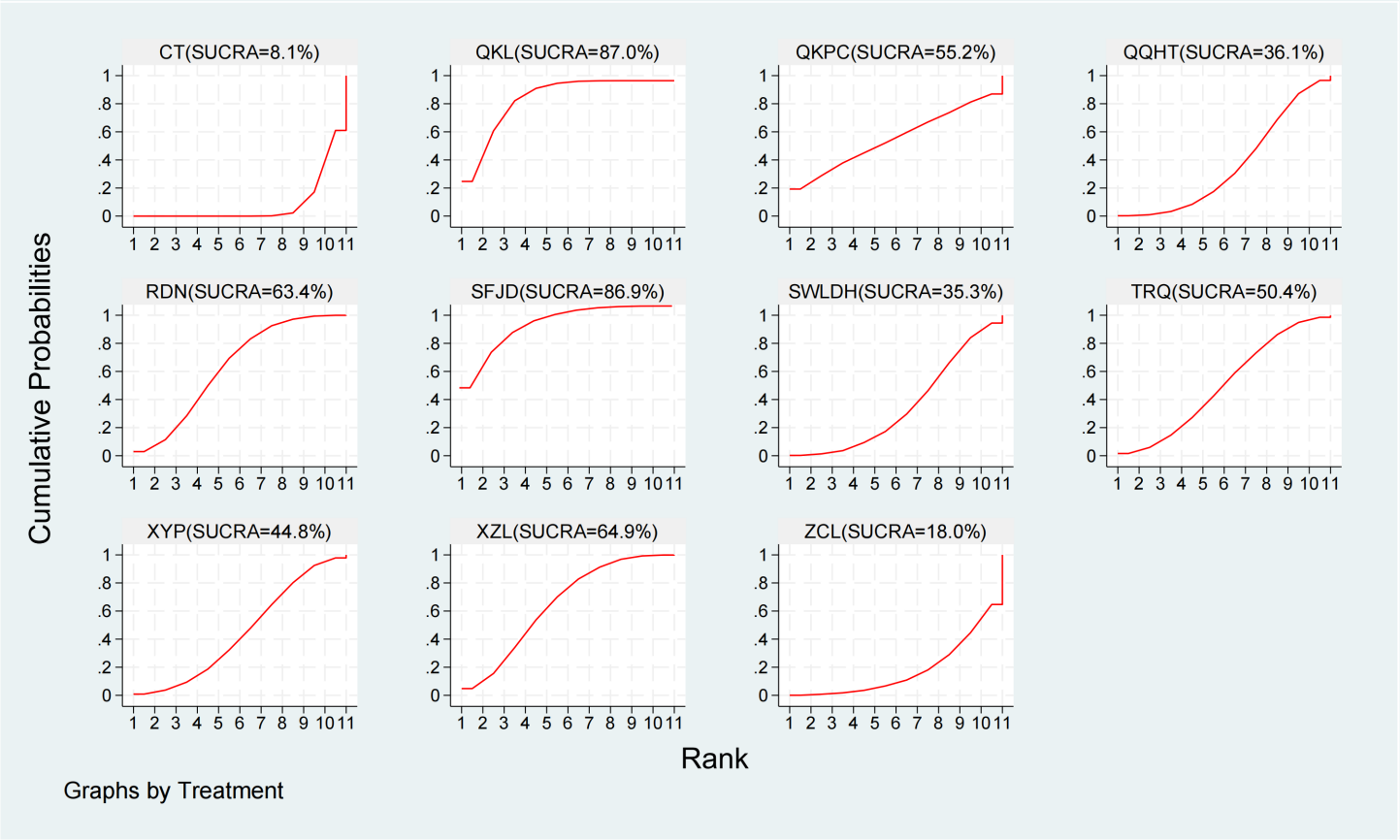


NOTE: **SFJD**: Shufeng Jiedu Capsule; **XZL**: Fresh Bamboo Juice Oral Liquid; **QQHT**: Qingqi Huatan Pill; **QKPC**: Qingke Pingchuan Granule; **TRQ**: Tanreqing Injection; **RDN**: Reduning Injection; **SWLDH**: Shiwei Longdanhua Capsule;

**ZCL**: Zhichuanling Injection; **QKL**: Qingkailing Injection; **XYP**: Xiyanping Injection; **CT**: Conventional biomedicine treatment.

Figure S8.5: Cumulative ranking curve plots of CPMs for **PH** in range network. Higher surface under the curve reflects higher probability of association with PH.

**
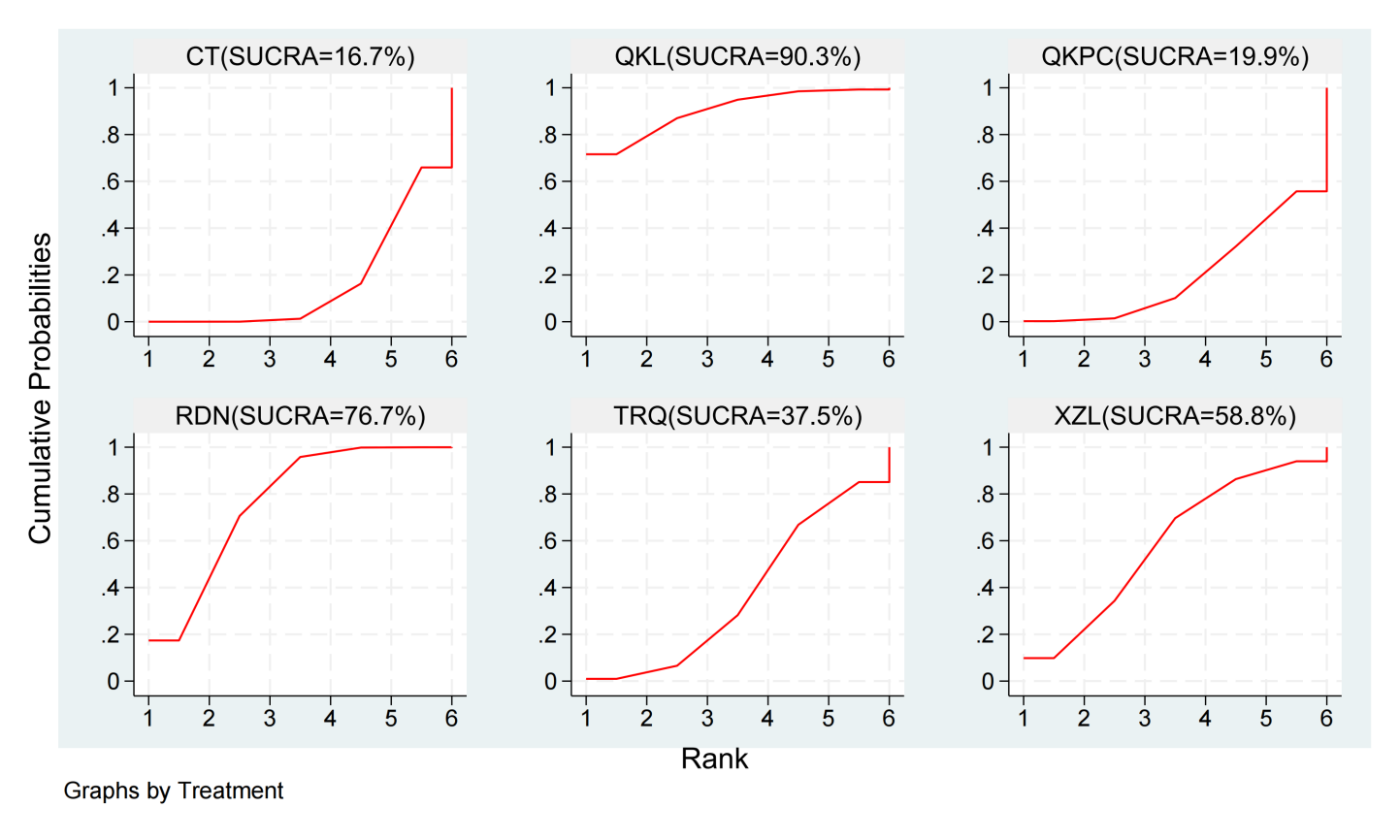
**

NOTE: **QKL**: Qingkailing Injection; **QKPC**: Qingke Pingchuan Granule; **RDN**: Reduning Injection; **TRQ**: Tanreqing Injection; **XZL**: Fresh Bamboo Juice Oral Liquid; **CT**: Conventional biomedicine treatment.

Figure S8.6: Cumulative ranking curve plots of CPMs for **PaO2** in range network. Higher surface under the curve reflects higher probability of association with PaO2.

**
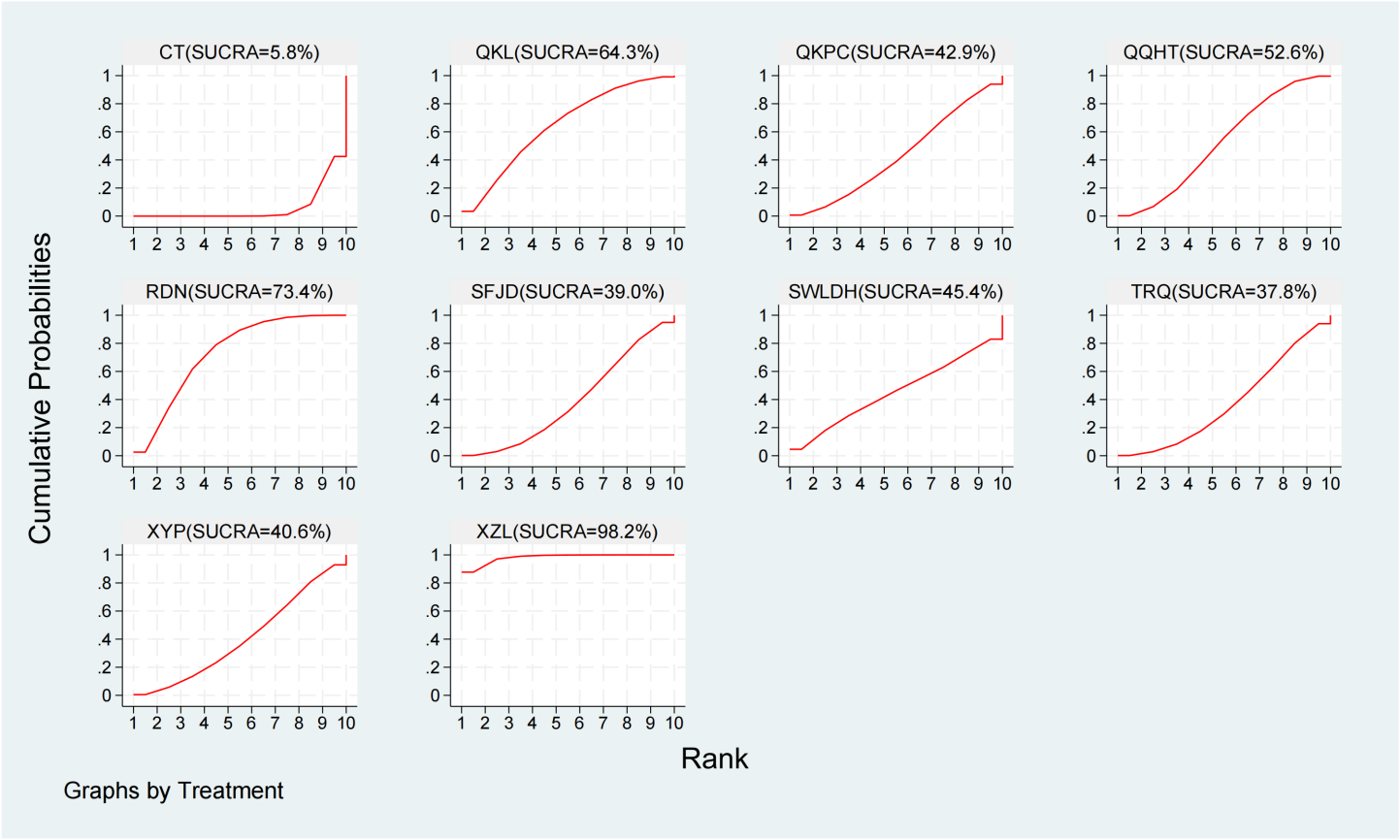
**

NOTE: **SFJD**: Shufeng Jiedu Capsule; **XZL**: Fresh Bamboo Juice Oral Liquid; **QQHT**: Qingqi Huatan Pill; **QKPC**: Qingke Pingchuan Granule; **TRQ**: Tanreqing Injection; **RDN**: Reduning Injection; **SWLDH**: Shiwei Longdanhua Capsule;

**QKL**: Qingkailing Injection; **XYP**: Xiyanping Injection; **CT**: Conventional biomedicine treatment.

Figure S8.7: Cumulative ranking curve plots of CPMs for **PaCO2** in range network. Higher surface under the curve reflects higher probability of association with PaCO2.

**
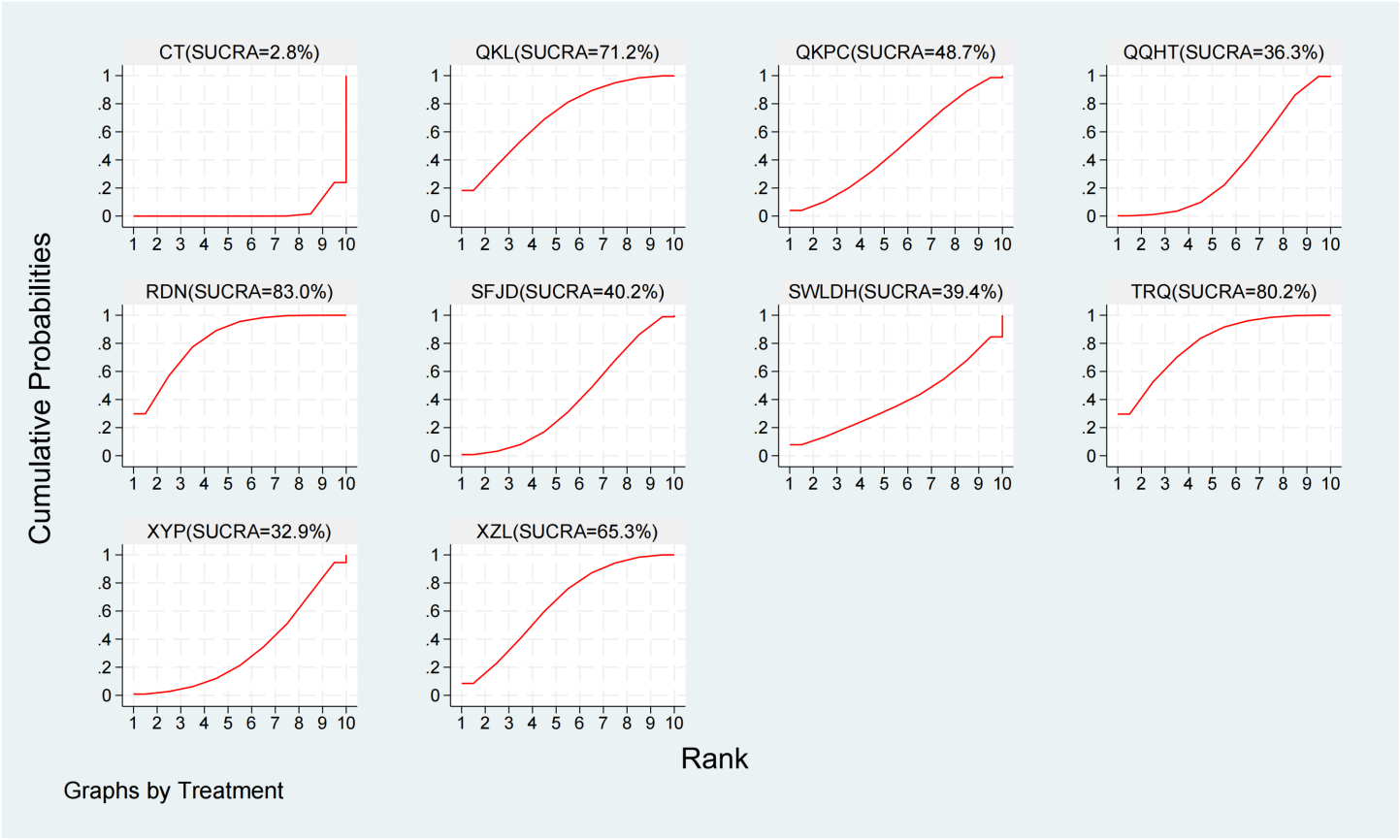
**

NOTE: **SFJD**: Shufeng Jiedu Capsule; **XZL**: Fresh Bamboo Juice Oral Liquid; **QQHT**: Qingqi Huatan Pill; **QKPC**: Qingke Pingchuan Granule; **TRQ**: Tanreqing Injection; **RDN**: Reduning Injection; **SWLDH**: Shiwei Longdanhua Capsule;

**QKL**: Qingkailing Injection; **XYP**: Xiyanping Injection; **CT**: Conventional biomedicine treatment.

Figure S8.8: Cumulative ranking curve plots of CPMs for **IL-6** in range network. Higher surface under the curve reflects higher probability of association with IL-6.

**
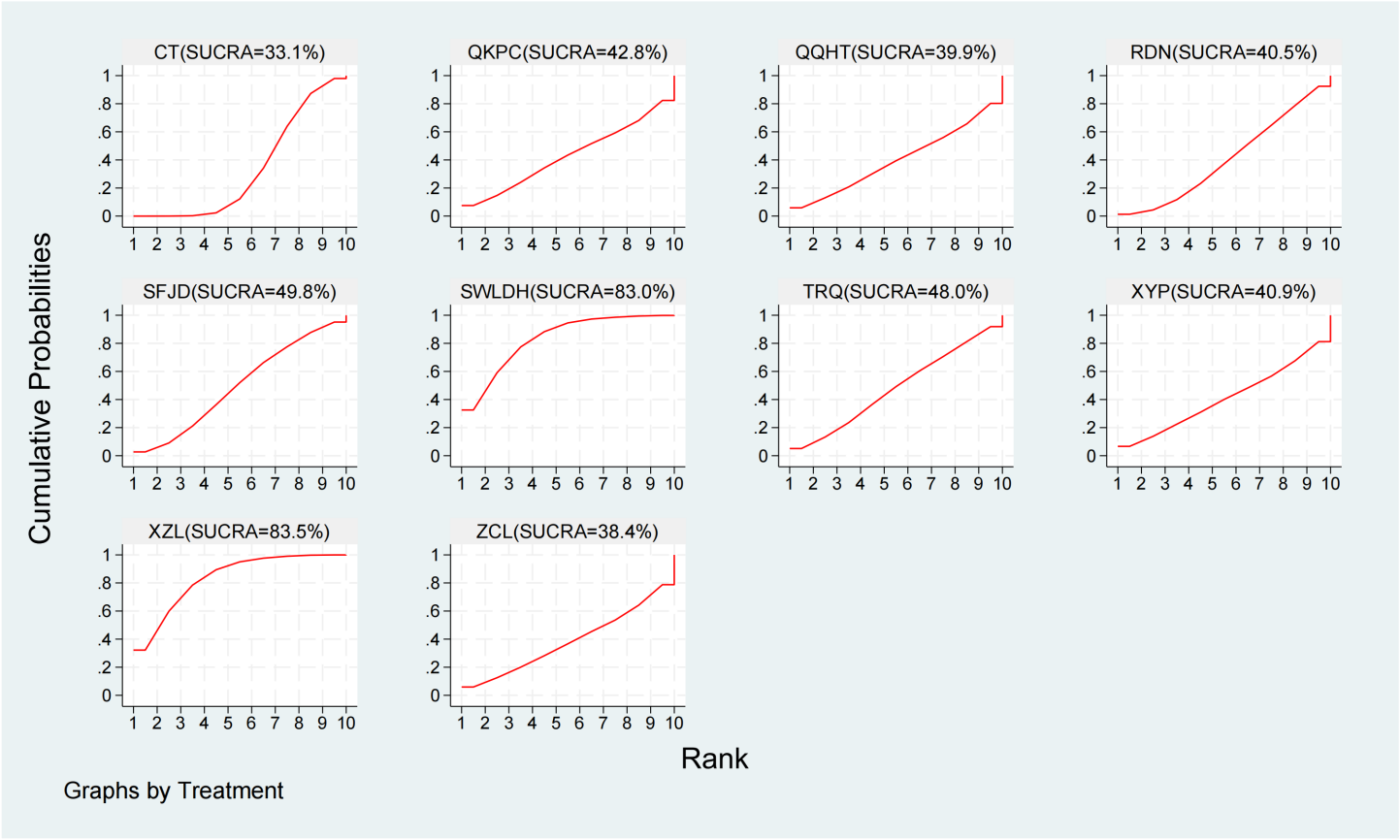
**

NOTE: **SFJD**: Shufeng Jiedu Capsule; **XZL**: Fresh Bamboo Juice Oral Liquid; **QQHT**: Qingqi Huatan Pill; **QKPC**: Qingke Pingchuan Granule; **TRQ**: Tanreqing Injection; **RDN**: Reduning Injection; **SWLDH**: Shiwei Longdanhua Capsule;

**ZCL**: Zhichuanling Injection; **XYP**: Xiyanping Injection; **CT**: Conventional biomedicine treatment.

Figure S8.9: Cumulative ranking curve plots of CPMs for **IL-8** in range network. Higher surface under the curve reflects higher probability of association with IL-8.

**
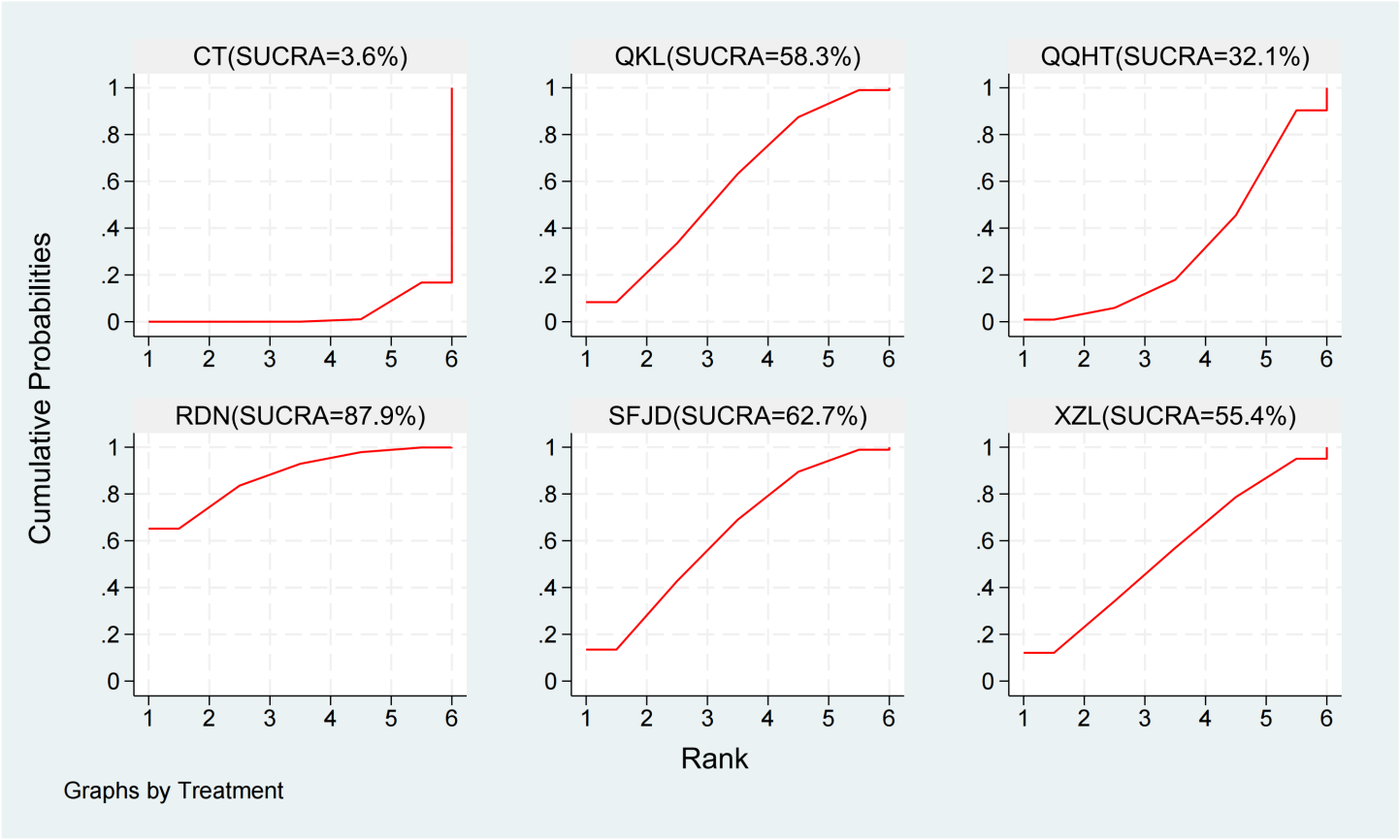
**

NOTE: **SFJD**: Shufeng Jiedu Capsule; **XZL**: Fresh Bamboo Juice Oral Liquid; **QQHT**: Qingqi Huatan Pill; **RDN**: Reduning Injection; **QKL**: Qingkailing Injection; **CT**: Conventional biomedicine treatment.

Figure S8.10: Cumulative ranking curve plots of CPMs for **TNF-α** in range network. Higher surface under the curve reflects higher probability of association with TNF-α.

**
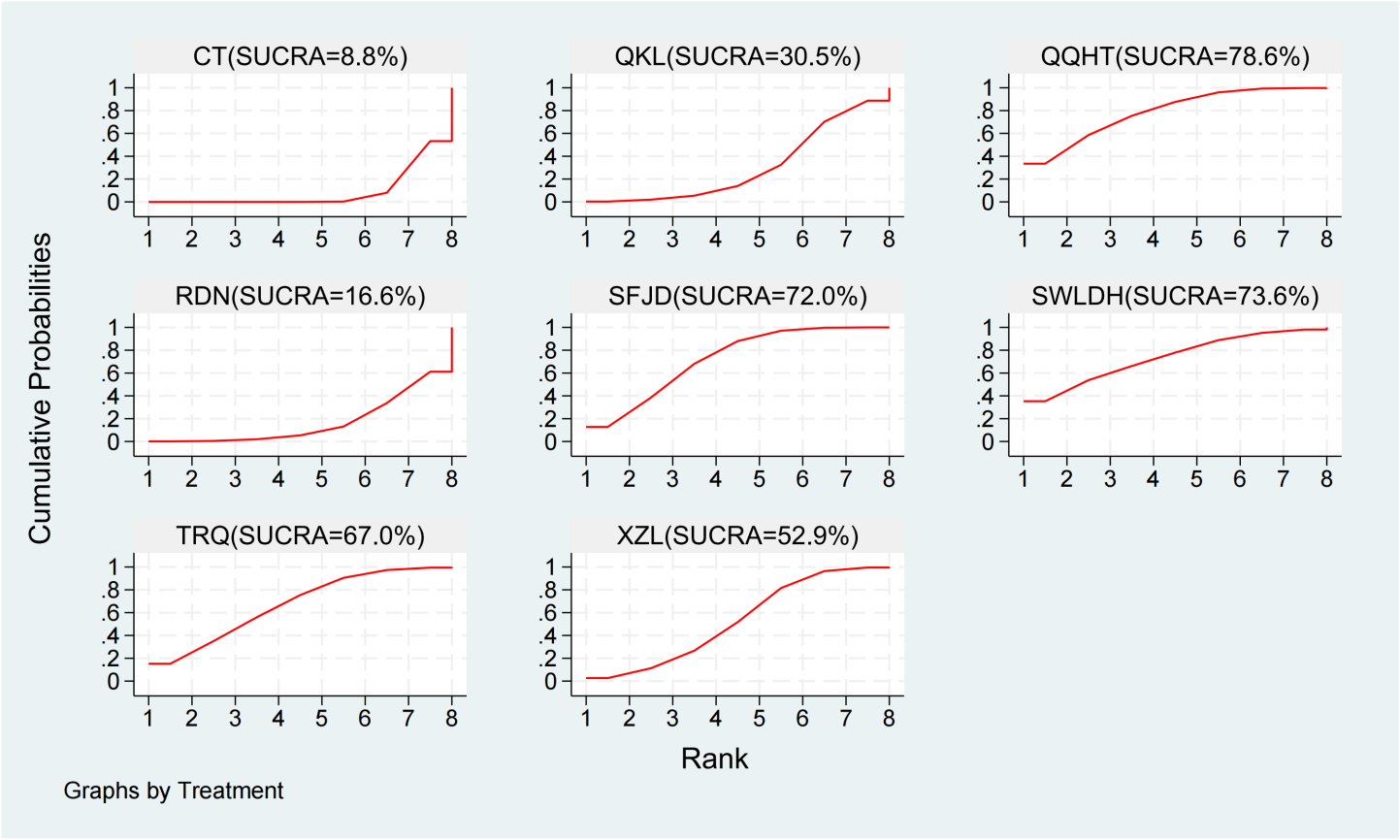
**

NOTE: **SFJD**: Shufeng Jiedu Capsule; **XZL**: Fresh Bamboo Juice Oral Liquid; **QQHT**: Qingqi Huatan Pill; **TRQ**: Tanreqing Injection; **RDN**: Reduning Injection; **SWLDH**: Shiwei Longdanhua Capsule; **QKL**: Qingkailing Injection; **CT**: Conventional biomedicine treatment.

Figure S8.11: Cumulative ranking curve plots of CPMs for **Adverse event** in range network. Higher surface under the curve reflects higher probability of association with Adverse event.

**
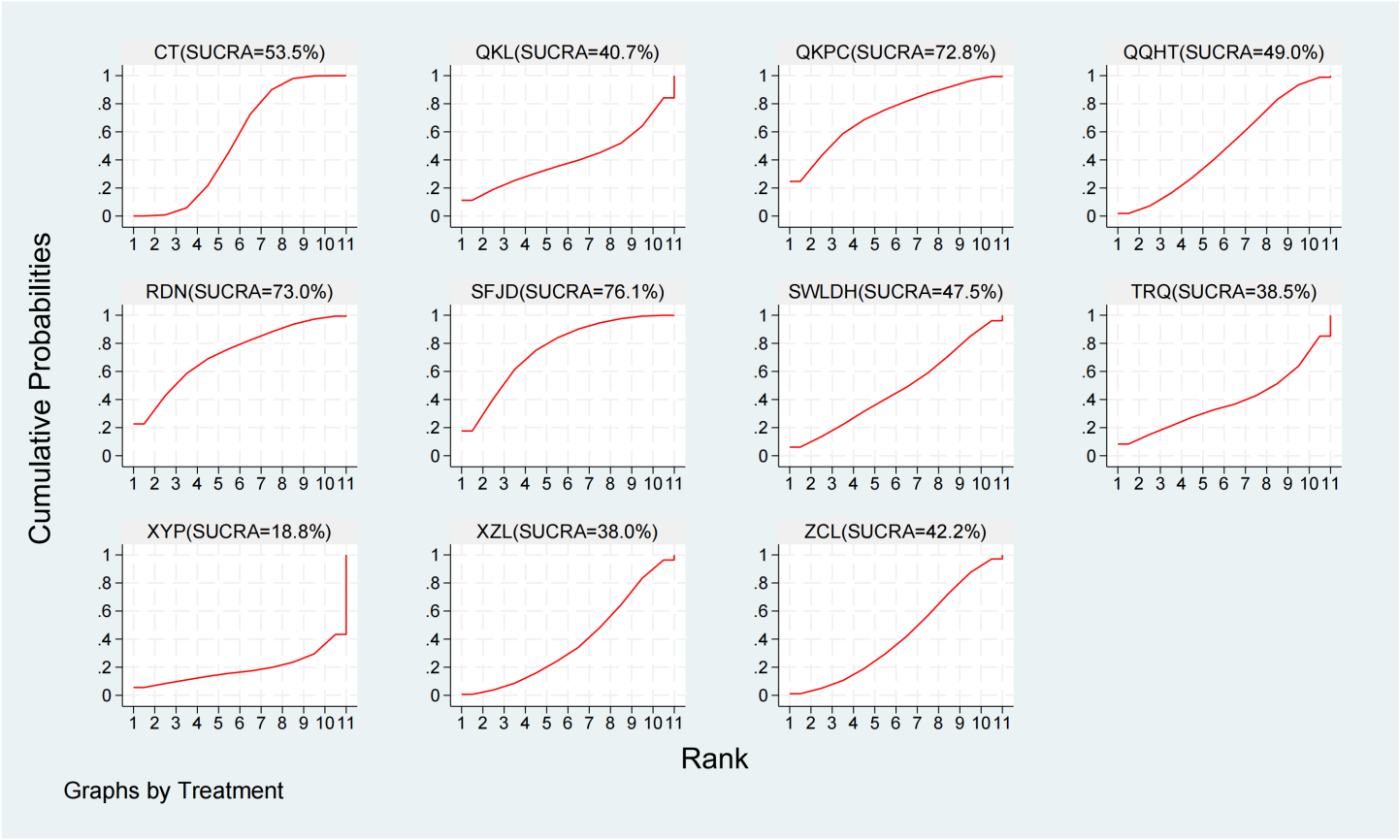
**

NOTE: **SFJD**: Shufeng Jiedu Capsule; **XZL**: Fresh Bamboo Juice Oral Liquid; **QQHT**: Qingqi Huatan Pill; **QKPC**: Qingke Pingchuan Granule; **TRQ**: Tanreqing Injection; **RDN**: Reduning Injection; **SWLDH**: Shiwei Longdanhua Capsule;

**ZCL**: Zhichuanling Injection; **QKL**: Qingkailing Injection; **XYP**: Xiyanping Injection; **CT**: Conventional biomedicine treatment.

**Appendix 9:Network meta-analysis of various CPMs on secondary endpoints of AECOPD.**

Figure S9.1 PH

A1 Network map of PH. Comparative network of CPMs and CT in AECOPD, where node size indicates the number of participants per group, and edge thickness represents the number of trials per comparison.

A2 Forest plot of PH.


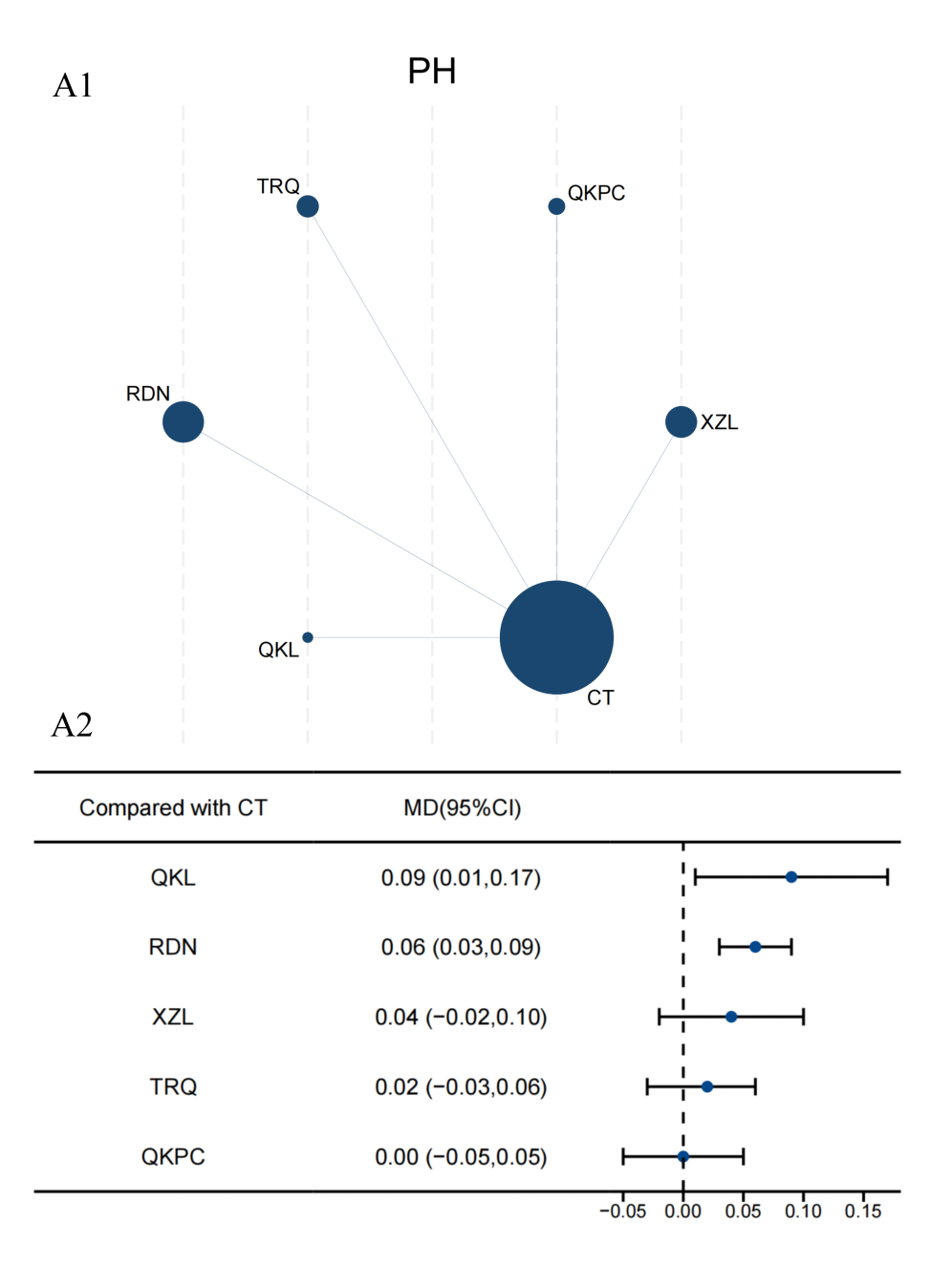


NOTE: **QKL**: Qingkailing Injection; **QKPC**: Qingke Pingchuan Granule; **RDN**: Reduning Injection; **TRQ**: Tanreqing Injection; **XZL**: Fresh Bamboo Juice Oral Liquid; **CT**: Conventional biomedicine treatment.

Figure S9.2 PaO2

B1 Network map of PaO2. Comparative network of CPMs and CT in AECOPD, where node size indicates the number of participants per group, and edge thickness represents the number of trials per comparison.

B2 Forest plot of PaO2.


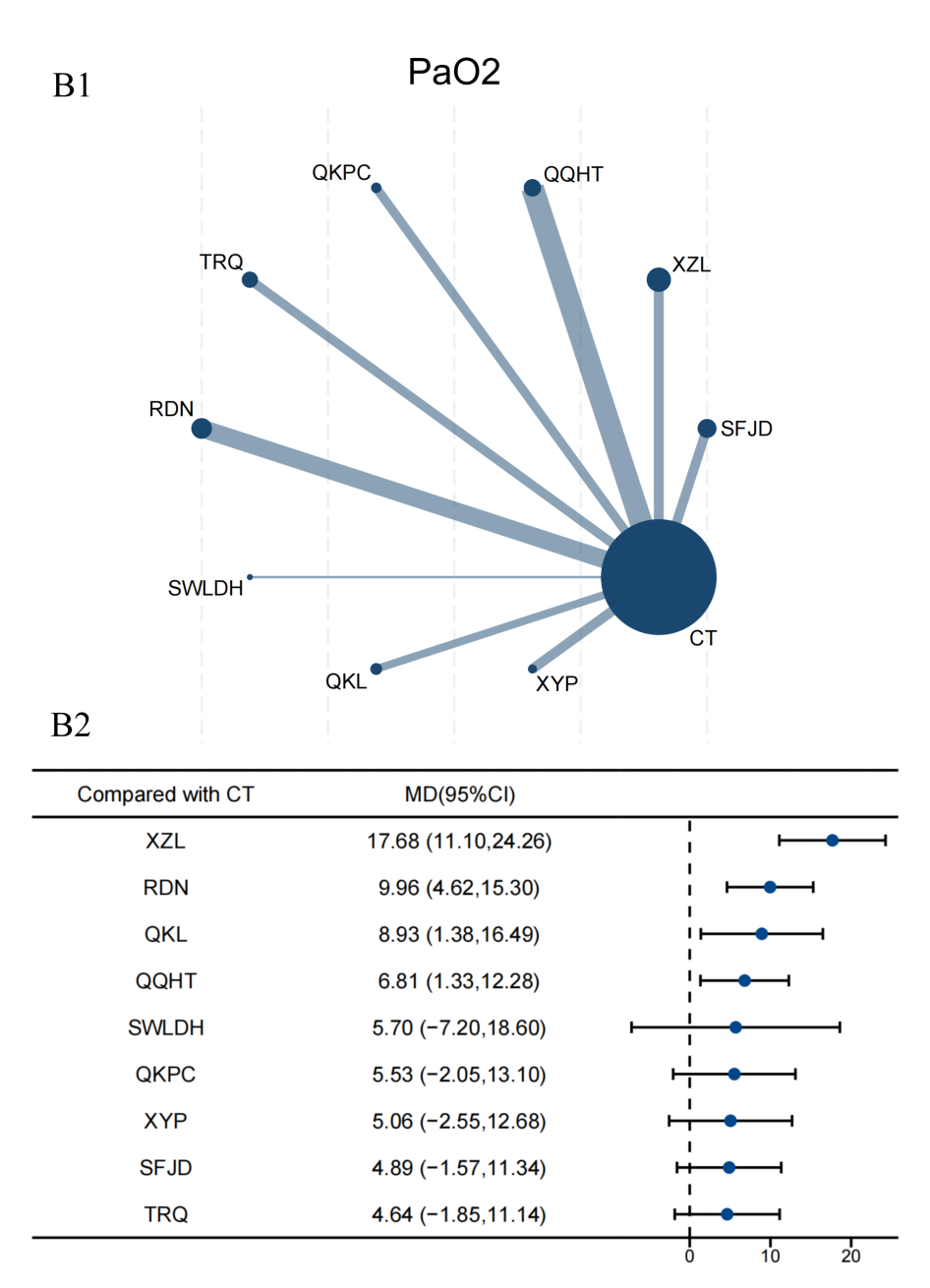


NOTE: **SFJD**: Shufeng Jiedu Capsule; **XZL**: Fresh Bamboo Juice Oral Liquid; **QQHT**: Qingqi Huatan Pill; **QKPC**: Qingke Pingchuan Granule; **TRQ**: Tanreqing Injection; **RDN**: Reduning Injection; **SWLDH**: Shiwei Longdanhua Capsule;

**QKL**: Qingkailing Injection; **XYP**: Xiyanping Injection; **CT**: Conventional biomedicine treatment.

Figure S9.3 PaCO2

C1 Network map of PaCO2. Comparative network of CPMs and CT in AECOPD, where node size indicates the number of participants per group, and edge thickness represents the number of trials per comparison.

C2 Forest plot of PaCO2.


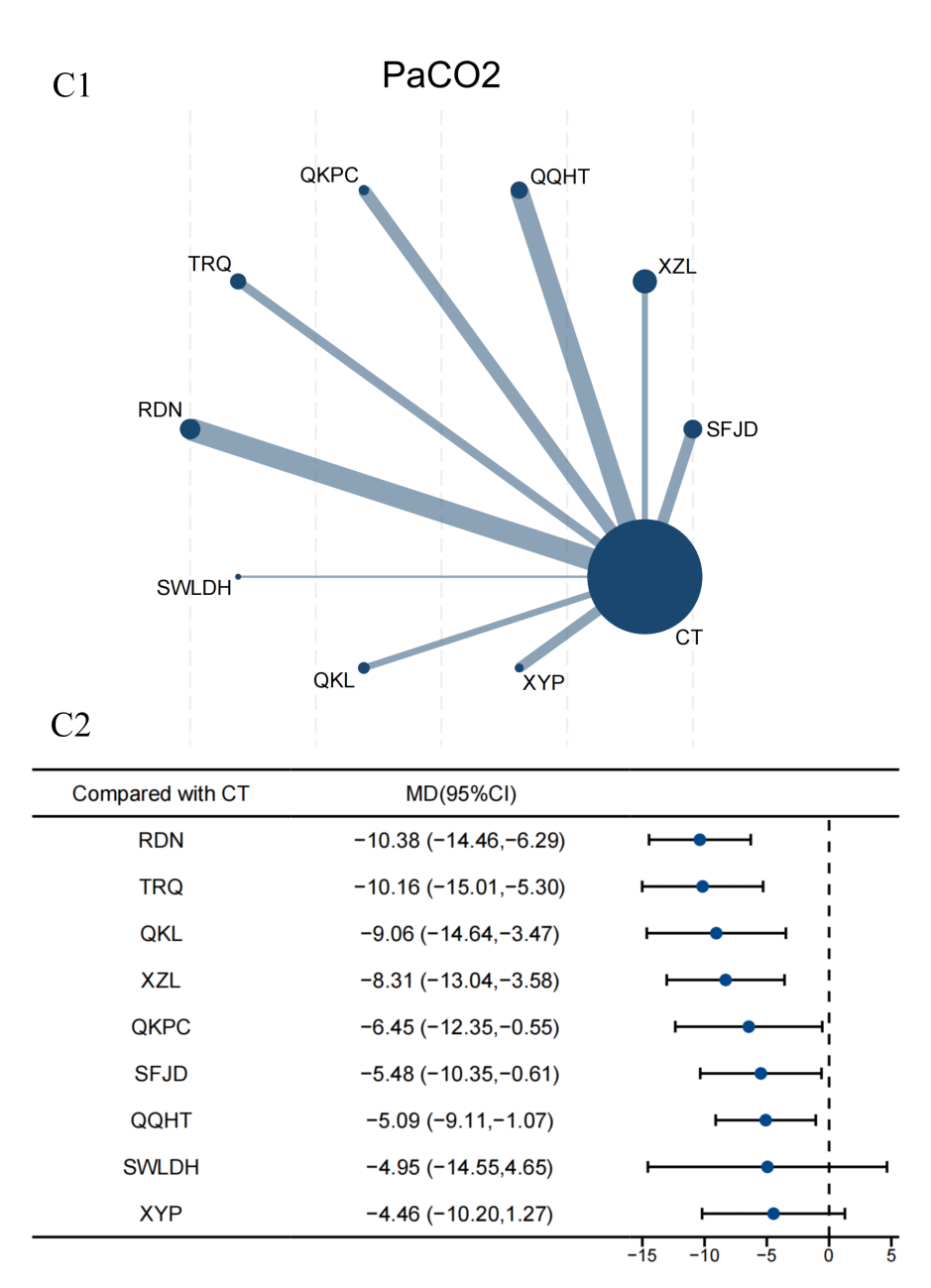


NOTE: **SFJD**: Shufeng Jiedu Capsule; **XZL**: Fresh Bamboo Juice Oral Liquid; **QQHT**: Qingqi Huatan Pill; **QKPC**: Qingke Pingchuan Granule; **TRQ**: Tanreqing Injection; **RDN**: Reduning Injection; **SWLDH**: Shiwei Longdanhua Capsule;

**QKL**: Qingkailing Injection; **XYP**: Xiyanping Injection; **CT**: Conventional biomedicine treatment.

Figure S9.4 IL-6

D1 Network map of IL-6. Comparative network of CPMs and CT in AECOPD, where node size indicates the number of participants per group, and edge thickness represents the number of trials per comparison.

D2 Forest plot of IL-6.


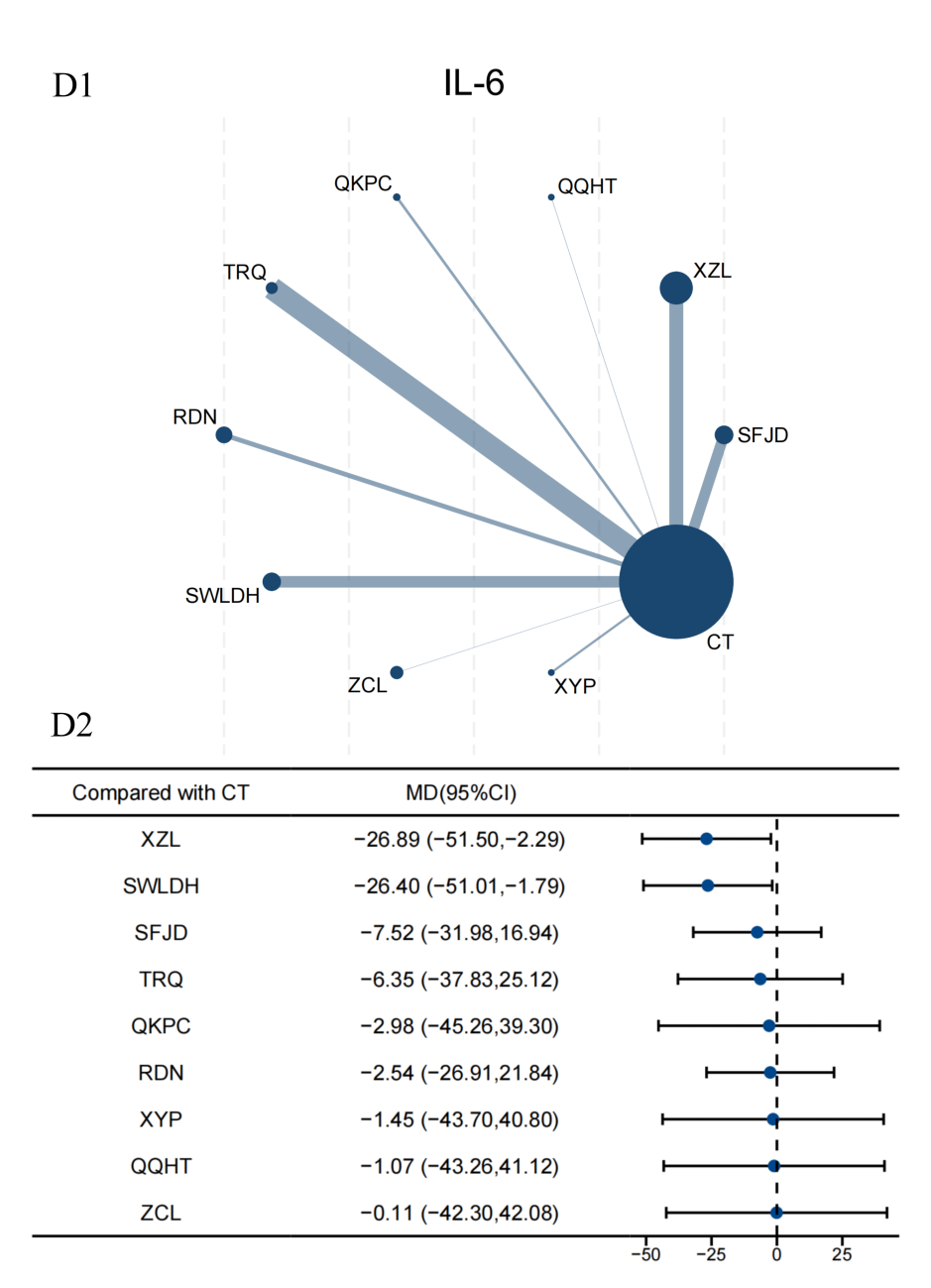


NOTE: **SFJD**: Shufeng Jiedu Capsule; **XZL**: Fresh Bamboo Juice Oral Liquid; **QQHT**: Qingqi Huatan Pill; **QKPC**: Qingke Pingchuan Granule; **TRQ**: Tanreqing Injection; **RDN**: Reduning Injection; **SWLDH**: Shiwei Longdanhua Capsule;

**ZCL**: Zhichuanling Injection; **XYP**: Xiyanping Injection; **CT**: Conventional biomedicine treatment.

Figure S9.5 IL-8

E1 Network map of IL-8. Comparative network of CPMs and CT in AECOPD, where node size indicates the number of participants per group, and edge thickness represents the number of trials per comparison.

E2 Forest plot of IL-8.


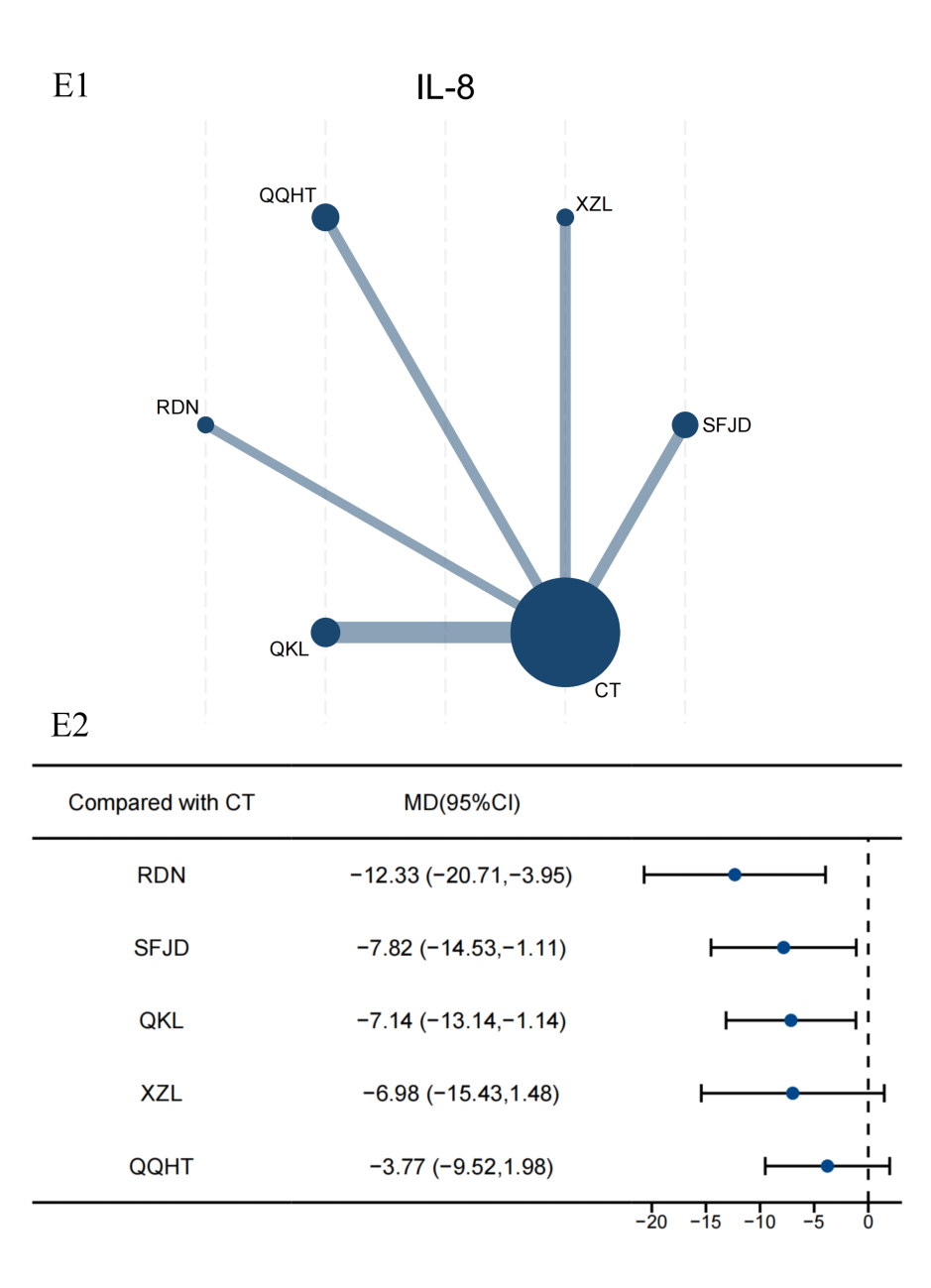


NOTE: **SFJD**: Shufeng Jiedu Capsule; **XZL**: Fresh Bamboo Juice Oral Liquid; **QQHT**: Qingqi Huatan Pill; **RDN**: Reduning Injection; **QKL**: Qingkailing Injection; **CT**: Conventional biomedicine treatment.

Figure S9.6 TNF-α

F1 Network map of TNF-α. Comparative network of CPMs and CT in AECOPD, where node size indicates the number of participants per group, and edge thickness represents the number of trials per comparison.

F2 Forest plot of TNF-α.


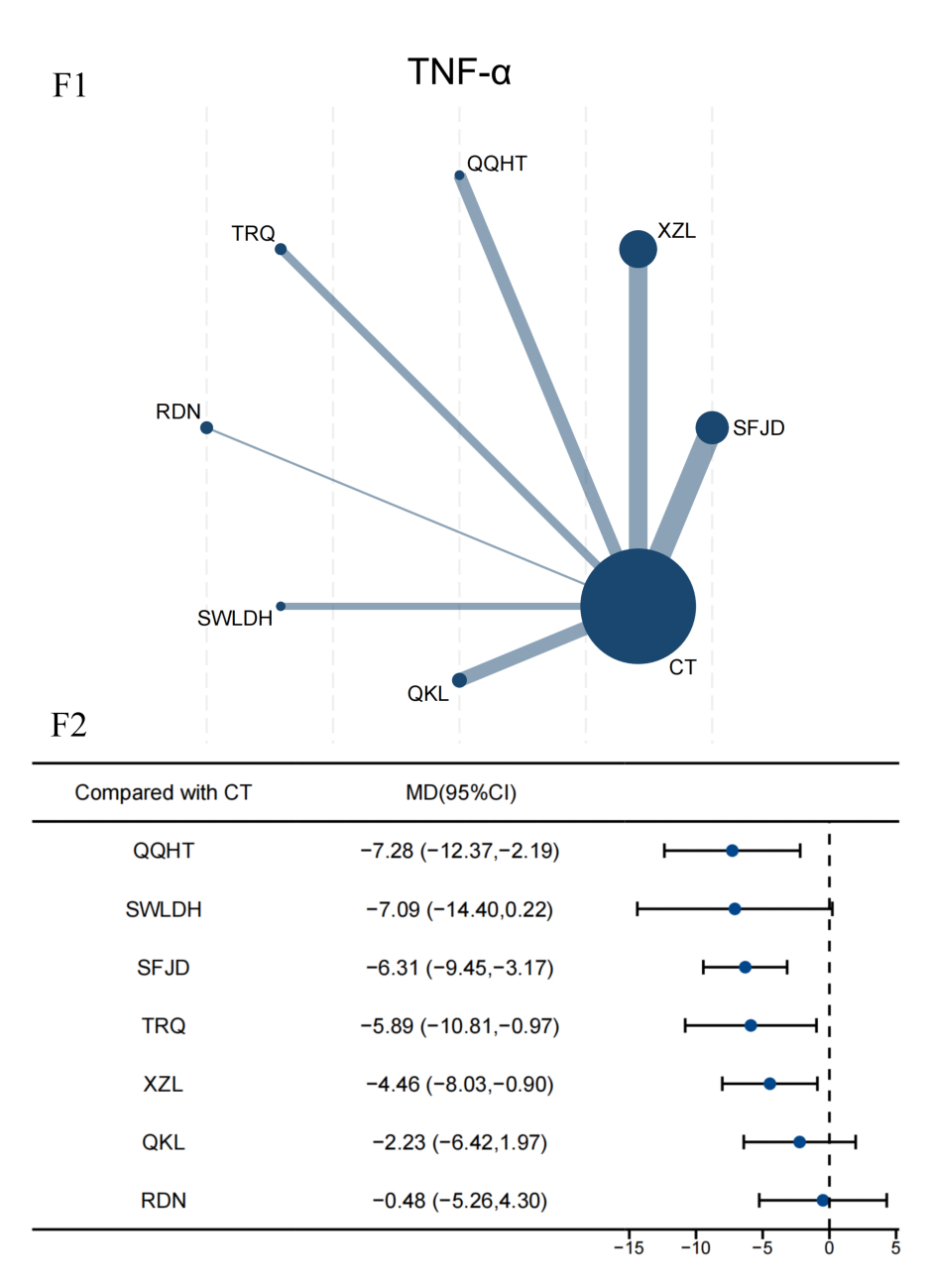


NOTE: **SFJD**: Shufeng Jiedu Capsule; **XZL**: Fresh Bamboo Juice Oral Liquid; **QQHT**: Qingqi Huatan Pill; **TRQ**: Tanreqing Injection; **RDN**: Reduning Injection; **SWLDH**: Shiwei Longdanhua Capsule; **QKL**: Qingkailing Injection; **CT**: Conventional biomedicine treatment.

Figure S9.7 Adverse event

G1 Network map of adverse event. Comparative network of CPMs and CT in AECOPD, where node size indicates the number of participants per group, and edge thickness represents the number of trials per comparison.

G2 Forest plot of adverse event.


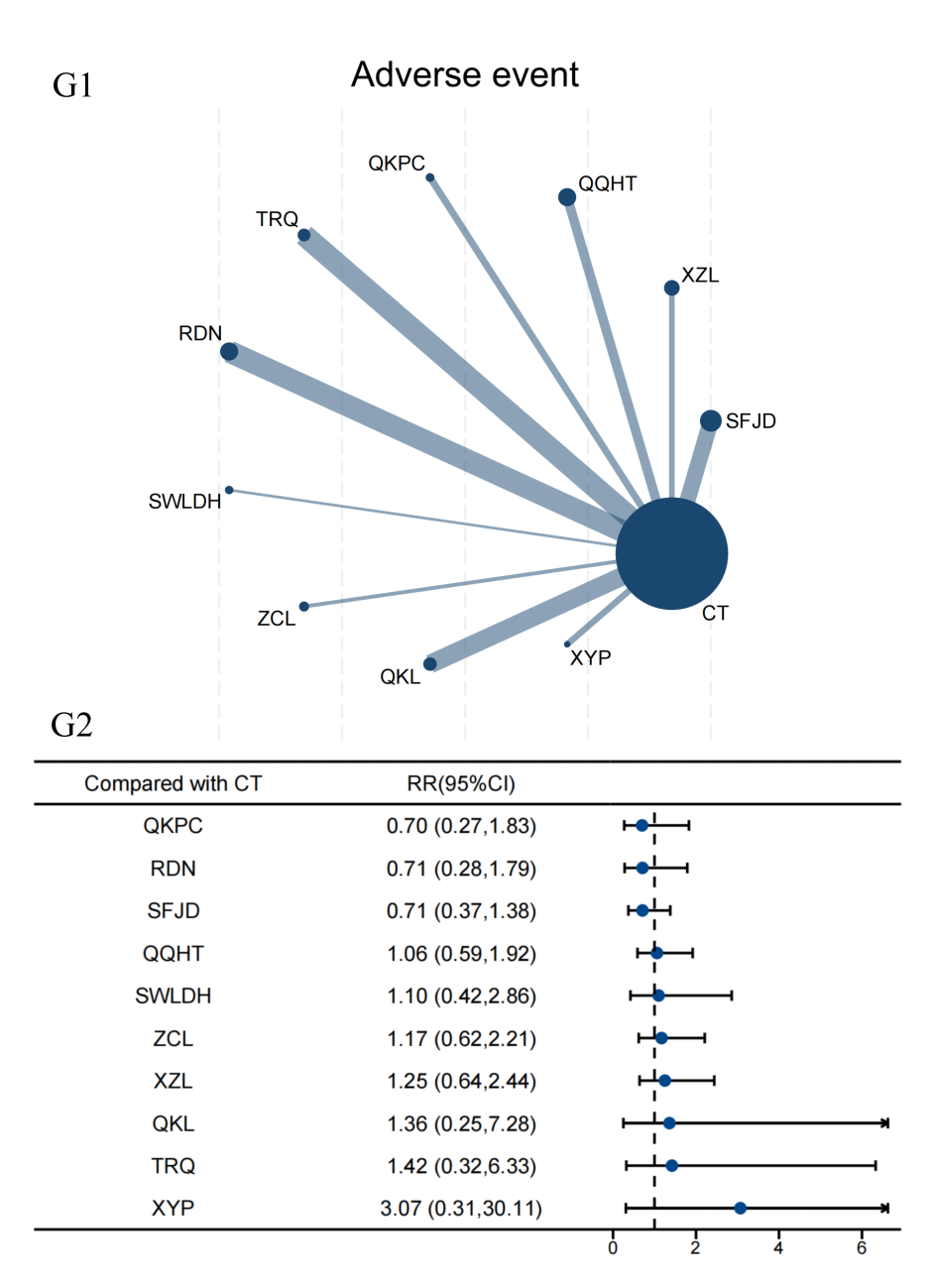


NOTE: **SFJD**: Shufeng Jiedu Capsule; **XZL**: Fresh Bamboo Juice Oral Liquid; **QQHT**: Qingqi Huatan Pill; **QKPC**: Qingke Pingchuan Granule; **TRQ**: Tanreqing Injection; **RDN**: Reduning Injection; **SWLDH**: Shiwei Longdanhua Capsule; **ZCL**: Zhichuanling Injection; **QKL**: Qingkailing Injection; **XYP**: Xiyanping Injection; **CT**: Conventional biomedicine treatment.

**Appendix 10: League table of Summary Estimates for CPMs on AECOPD derived from Network Meta-analysis of of 84 Trials**

Table S10.1: Total effective rate

The columns represent the comparison of the row drug class to the column drug class. The rows represent the comparison of the row drug class to the column drug class. The effect estimates are expressed as RR and 95% CI. For example, the RR in **Total effective rate** for SFJD compared to XZL is 0.87 (0.49, 1.55). **RR＞1 favors the drug in the column (yellow), and RR＜1 favors the drug in the row (pink).**

| QKL |  |  |  |  |  |  |  |  |  |
| --- | --- | --- | --- | --- | --- | --- | --- | --- | --- |
| 0.98 (0.35,2.71) | QKPC |  |  |  |  |  |  |  |  |
| 1.01 (0.47,2.18) | 1.03 (0.39,2.71) | QQHT |  |  |  |  |  |  |  |
| 0.98 (0.48,1.96) | 1.00 (0.40,2.49) | 0.97 (0.52,1.82) | RDN |  |  |  |  |  |  |
| 1.30 (0.65,2.58) | 1.33 (0.54,3.28) | 1.29 (0.70,2.39) | 1.33 (0.79,2.25) | SFJD |  |  |  |  |  |
| 2.06 (0.95,4.45) | 2.10 (0.80,5.55) | 2.04 (1.01,4.15) | 2.11 (1.12,3.97) | 1.58 (0.85,2.94) | SWLDH |  |  |  |  |
| 1.41 (0.64,3.11) | 1.44 (0.53,3.86) | 1.40 (0.67,2.90) | 1.44 (0.75,2.79) | 1.08 (0.57,2.06) | 0.68 (0.33,1.42) | TRQ |  |  |  |
| 1.24 (0.56,2.72) | 1.26 (0.47,3.38) | 1.23 (0.59,2.54) | 1.27 (0.66,2.43) | 0.95 (0.50,1.80) | 0.60 (0.29,1.24) | 0.88 (0.41,1.86) | XYP |  |  |
| 1.13 (0.54,2.37) | 1.15 (0.45,2.97) | 1.12 (0.57,2.20) | 1.16 (0.64,2.09) | 0.87 (0.49,1.55) | 0.55 (0.28,1.08) | 0.80 (0.40,1.62) | 0.91 (0.46,1.83) | XZL |  |
| 0.96 (0.40,2.29) | 0.98 (0.34,2.80) | 0.95 (0.42,2.15) | 0.98 (0.47,2.08) | 0.74 (0.35,1.54) | 0.47 (0.21,1.05) | 0.68 (0.30,1.58) | 0.78 (0.34,1.78) | 0.85 (0.39,1.86) | ZCL |

NOTE: **SFJD**: Shufeng Jiedu Capsule; **XZL**: Fresh Bamboo Juice Oral Liquid; **QQHT**: Qingqi Huatan Pill; **QKPC**: Qingke Pingchuan Granule; **TRQ**: Tanreqing Injection; **RDN**: Reduning Injection; **SWLDH**: Shiwei Longdanhua Capsule; **ZCL**: Zhichuanling Injection; **QKL**: Qingkailing Injection; **XYP**: Xiyanping Injection.

Table S10.2: FVC

The columns represent the comparison of the row drug class to the column drug class. The rows represent the comparison of the row drug class to the column drug class. The effect estimates are expressed as MD and 95% CI. For example, the MD in **FVC** for SFJD compared to XZL is 0.25 (0.02, 0.49). MD＞0 favors the drug in the column(yellow), and MD＜0 favors the drug in the row(pink).

| QKL |  |  |  |  |  |  |  |  |  |
| --- | --- | --- | --- | --- | --- | --- | --- | --- | --- |
| 0.22 (-0.14,0.58) | QKPC |  |  |  |  |  |  |  |  |
| 0.59 (0.31,0.87) | 0.37 (-0.03,0.77) | QQHT |  |  |  |  |  |  |  |
| -0.07 (-0.31,0.16) | -0.29 (-0.66,0.08) | -0.66 (-0.96,-0.36) | RDN |  |  |  |  |  |  |
| -0.07 (-0.27,0.12) | -0.29 (-0.64,0.06) | -0.66 (-0.92,-0.40) | 0.00 (-0.22,0.22) | SFJD |  |  |  |  |  |
| -0.30 (-0.63,0.02) | -0.52 (-0.95,-0.09) | -0.89 (-1.26,-0.52) | -0.23 (-0.57,0.11) | -0.23 (-0.54,0.08) | SWLDH |  |  |  |  |
| 0.01 (-0.24,0.26) | -0.21 (-0.59,0.17) | -0.58 (-0.88,-0.27) | 0.08 (-0.18,0.35) | 0.08 (-0.15,0.31) | 0.31 (-0.04,0.66) | TRQ |  |  |  |
| -0.00 (-0.22,0.22) | -0.22 (-0.58,0.15) | -0.59 (-0.87,-0.30) | 0.07 (-0.17,0.32) | 0.07 (-0.13,0.28) | 0.30 (-0.03,0.64) | -0.01 (-0.27,0.25) | XYP |  |  |
| 0.18 (-0.07,0.44) | -0.04 (-0.42,0.35) | -0.41 (-0.72,-0.09) | 0.26 (-0.02,0.53) | 0.25 (0.02,0.49) | 0.48 (0.13,0.84) | 0.17 (-0.11,0.45) | 0.18 (-0.08,0.45) | XZL |  |
| -0.19 (-0.46,0.08) | -0.41 (-0.80,-0.01) | -0.78 (-1.10,-0.45) | -0.12 (-0.41,0.17) | -0.12 (-0.38,0.14) | 0.11 (-0.26,0.48) | -0.20 (-0.50,0.10) | -0.19 (-0.47,0.09) | -0.37 (-0.68,-0.07) | ZCL |

NOTE: **SFJD**: Shufeng Jiedu Capsule; **XZL**: Fresh Bamboo Juice Oral Liquid; **QQHT**: Qingqi Huatan Pill; **QKPC**: Qingke Pingchuan Granule; **TRQ**: Tanreqing Injection; **RDN**: Reduning Injection; **SWLDH**: Shiwei Longdanhua Capsule; **ZCL**: Zhichuanling Injection; **QKL**: Qingkailing Injection; **XYP**: Xiyanping Injection.

Table S10.3: FEV1

The columns represent the comparison of the row drug class to the column drug class. The rows represent the comparison of the row drug class to the column drug class. The effect estimates are expressed as MD and 95% CI. For example, the MD in **FEV1** for SFJD compared to XZL is 0.17 (-0.01, 0.34). MD＞0 favors the drug in the column(yellow), and MD＜0 favors the drug in the row(pink).

| QKL |  |  |  |  |  |  |  |  |  |
| --- | --- | --- | --- | --- | --- | --- | --- | --- | --- |
| 0.22 (-0.12,0.57) | QKPC |  |  |  |  |  |  |  |  |
| 0.15 (-0.10,0.40) | -0.08 (-0.44,0.29) | QQHT |  |  |  |  |  |  |  |
| 0.07 (-0.12,0.26) | -0.16 (-0.49,0.17) | -0.08 (-0.31,0.14) | RDN |  |  |  |  |  |  |
| -0.07 (-0.26,0.12) | -0.29 (-0.62,0.03) | -0.22 (-0.44,0.01) | -0.14 (-0.29,0.02) | SFJD |  |  |  |  |  |
| -0.51 (-0.84,-0.18) | -0.73 (-1.16,-0.31) | -0.66 (-1.01,-0.31) | -0.58 (-0.89,-0.26) | -0.44 (-0.75,-0.13) | SWLDH |  |  |  |  |
| 0.14 (-0.07,0.34) | -0.09 (-0.43,0.25) | -0.01 (-0.25,0.23) | 0.07 (-0.11,0.24) | 0.21 (0.03,0.38) | 0.65 (0.32,0.97) | TRQ |  |  |  |
| 0.08 (-0.12,0.28) | -0.15 (-0.48,0.19) | -0.07 (-0.31,0.17) | 0.01 (-0.16,0.18) | 0.15 (-0.02,0.32) | 0.59 (0.27,0.91) | -0.06 (-0.25,0.13) | XYP |  |  |
| 0.10 (-0.11,0.30) | -0.13 (-0.46,0.21) | -0.05 (-0.29,0.19) | 0.03 (-0.14,0.21) | 0.17 (-0.01,0.34) | 0.61 (0.29,0.93) | -0.04 (-0.23,0.16) | 0.02 (-0.17,0.21) | XZL |  |
| -0.35 (-0.63,-0.08) | -0.58 (-0.96,-0.19) | -0.50 (-0.80,-0.20) | -0.42 (-0.67,-0.17) | -0.28 (-0.54,-0.03) | 0.16 (-0.21,0.53) | -0.49 (-0.75,-0.22) | -0.43 (-0.69,-0.17) | -0.45 (-0.72,-0.19) | ZCL |

NOTE: **SFJD**: Shufeng Jiedu Capsule; **XZL**: Fresh Bamboo Juice Oral Liquid; **QQHT**: Qingqi Huatan Pill; **QKPC**: Qingke Pingchuan Granule; **TRQ**: Tanreqing Injection; **RDN**: Reduning Injection; **SWLDH**: Shiwei Longdanhua Capsule; **ZCL**: Zhichuanling Injection; **QKL**: Qingkailing Injection; **XYP**: Xiyanping Injection.

Table S10.4: FEV1/FVC

The columns represent the comparison of the row drug class to the column drug class. The rows represent the comparison of the row drug class to the column drug class. The effect estimates are expressed as MD and 95% CI. For example, the MD in **FEV1/FVC** for QKL compared to QKPC is 3.60 (-7.31, 14.51). MD＞0 favors the drug in the column(yellow), and MD＜0 favors the drug in the row(pink).

| QKL |  |  |  |  |  |  |  |  |  |
| --- | --- | --- | --- | --- | --- | --- | --- | --- | --- |
| 3.60 (-7.31,14.51) | QKPC |  |  |  |  |  |  |  |  |
| 5.61 (-0.47,11.70) | 2.01 (-8.51,12.53) | QQHT |  |  |  |  |  |  |  |
| 2.99 (-2.92,8.90) | -0.61 (-11.03,9.81) | -2.63 (-7.77,2.52) | RDN |  |  |  |  |  |  |
| 0.40 (-5.19,5.99) | -3.20 (-13.44,7.04) | -5.22 (-10.00,-0.43) | -2.59 (-7.14,1.96) | SFJD |  |  |  |  |  |
| 5.71 (-0.56,11.98) | 2.11 (-8.52,12.73) | 0.10 (-5.46,5.65) | 2.72 (-2.64,8.08) | 5.31 (0.30,10.32) | SWLDH |  |  |  |  |
| 4.17 (-2.23,10.58) | 0.57 (-10.13,11.28) | -1.44 (-7.15,4.27) | 1.18 (-4.34,6.70) | 3.77 (-1.41,8.95) | -1.54 (-7.44,4.37) | TRQ |  |  |  |
| 4.81 (-1.52,11.15) | 1.21 (-9.45,11.88) | -0.80 (-6.43,4.83) | 1.83 (-3.61,7.26) | 4.42 (-0.67,9.50) | -0.89 (-6.72,4.93) | 0.64 (-5.33,6.61) | XYP |  |  |
| 2.79 (-3.31,8.89) | -0.81 (-11.34,9.71) | -2.83 (-8.19,2.54) | -0.20 (-5.36,4.96) | 2.39 (-2.40,7.18) | -2.92 (-8.49,2.64) | -1.38 (-7.11,4.34) | -2.03 (-7.66,3.61) | XZL |  |
| 7.91 (0.63,15.18) | 4.31 (-6.94,15.55) | 2.29 (-4.38,8.96) | 4.92 (-1.59,11.42) | 7.51 (1.29,13.73) | 2.20 (-4.64,9.03) | 3.73 (-3.22,10.69) | 3.09 (-3.80,9.98) | 5.12 (-1.56,11.80) | ZCL |

NOTE: **SFJD**: Shufeng Jiedu Capsule; **XZL**: Fresh Bamboo Juice Oral Liquid; **QQHT**: Qingqi Huatan Pill; **QKPC**: Qingke Pingchuan Granule; **TRQ**: Tanreqing Injection; **RDN**: Reduning Injection; **SWLDH**: Shiwei Longdanhua Capsule; **ZCL**: Zhichuanling Injection; **QKL**: Qingkailing Injection; **XYP**: Xiyanping Injection.

Table S10.5: PH

The columns represent the comparison of the row drug class to the column drug class. The rows represent the comparison of the row drug class to the column drug class. The effect estimates are expressed as MD and 95% CI. For example, the MD in **PH** for XZL compared to QKPC is 0.04 (-0.04, 0.12). MD＞0 favors the drug in the column(yellow), and MD＜0 favors the drug in the row(pink).

| QKL |  |  |  |  |
| --- | --- | --- | --- | --- |
| 0.09 (-0.00,0.18) | QKPC |  |  |  |
| 0.03 (-0.05,0.12) | -0.06 (-0.12,-0.00) | RDN |  |  |
| 0.07 (-0.02,0.16) | -0.02 (-0.08,0.05) | 0.04 (-0.01,0.10) | TRQ |  |
| 0.05 (-0.05,0.15) | -0.04 (-0.12,0.04) | 0.02 (-0.05,0.09) | -0.02 (-0.10,0.05) | XZL |

NOTE: **QKL**: Qingkailing Injection; **QKPC**: Qingke Pingchuan Granule; **RDN**: Reduning Injection; **TRQ**: Tanreqing Injection; **XZL**: Fresh Bamboo Juice Oral Liquid.

Table S10.6: PaO2

The columns represent the comparison of the row drug class to the column drug class. The rows represent the comparison of the row drug class to the column drug class. The effect estimates are expressed as MD and 95% CI. For example, the MD in **PaO2** for SFJD compared to XZL is -12.84 (-21.92, -3.76). MD＞0 favors the drug in the column(yellow), and MD＜0 favors the drug in the row(pink).

| QKL |  |  |  |  |  |  |  |  |
| --- | --- | --- | --- | --- | --- | --- | --- | --- |
| 3.41 (-7.29,14.11) | QKPC |  |  |  |  |  |  |  |
| 2.13 (-7.20,11.46) | -1.28 (-10.63,8.07) | QQHT |  |  |  |  |  |  |
| -1.03 (-10.28,8.23) | -4.43 (-13.70,4.84) | -3.15 (-10.80,4.49) | RDN |  |  |  |  |  |
| 4.05 (-5.89,13.99) | 0.64 (-9.31,10.59) | 1.92 (-6.54,10.38) | 5.07 (-3.30,13.45) | SFJD |  |  |  |  |
| 3.23 (-11.71,18.18) | -0.17 (-15.13,14.78) | 1.11 (-12.90,15.12) | 4.26 (-9.70,18.22) | -0.81 (-15.23,13.61) | SWLDH |  |  |  |
| 4.29 (-5.67,14.26) | 0.89 (-9.09,10.86) | 2.16 (-6.33,10.66) | 5.32 (-3.09,13.72) | 0.24 (-8.91,9.40) | 1.06 (-13.38,15.50) | TRQ |  |  |
| 3.87 (-6.86,14.60) | 0.47 (-10.28,11.21) | 1.74 (-7.63,11.12) | 4.90 (-4.40,14.20) | -0.17 (-10.16,9.81) | 0.64 (-14.34,15.61) | -0.42 (-10.43,9.59) | XYP |  |
| -8.75 (-18.75,1.26) | -12.15 (-22.19,-2.12) | -10.88 (-19.44,-2.32) | -7.72 (-16.20,0.75) | -12.79 (-22.01,-3.58) | -11.98 (-26.46,2.50) | -13.04 (-22.28,-3.80) | -12.62 (-22.68,-2.56) | XZL |

NOTE: **SFJD**: Shufeng Jiedu Capsule; **XZL**: Fresh Bamboo Juice Oral Liquid; **QQHT**: Qingqi Huatan Pill; **QKPC**: Qingke Pingchuan Granule; **TRQ**: Tanreqing Injection; **RDN**: Reduning Injection; **SWLDH**: Shiwei Longdanhua Capsule; **QKL**: Qingkailing Injection; **XYP**: Xiyanping Injection.

Table S10.7: PaCO2

The columns represent the comparison of the row drug class to the column drug class. The rows represent the comparison of the row drug class to the column drug class. The effect estimates are expressed as MD and 95% CI. For example, the MD in **PaCO2** for SFJD compared to XZL is 2.85 (-3.85, 9.56). MD <0 favors the drug in the column(yellow), and MD >0 favors the drug in the row(pink).

| QKL |  |  |  |  |  |  |  |  |
| --- | --- | --- | --- | --- | --- | --- | --- | --- |
| -2.60 (-10.73,5.52) | QKPC |  |  |  |  |  |  |  |
| -3.96 (-10.84,2.92) | -1.36 (-8.50,5.78) | QQHT |  |  |  |  |  |  |
| 1.32 (-5.60,8.24) | 3.93 (-3.25,11.10) | 5.28 (-0.45,11.01) | RDN |  |  |  |  |  |
| -3.57 (-10.98,3.84) | -0.97 (-8.62,6.68) | 0.39 (-5.92,6.70) | -4.90 (-11.25,1.46) | SFJD |  |  |  |  |
| -4.11 (-15.21,7.00) | -1.50 (-12.77,9.76) | -0.14 (-10.55,10.26) | -5.43 (-15.86,5.00) | -0.53 (-11.29,10.23) | SWLDH |  |  |  |
| 1.10 (-6.29,8.50) | 3.71 (-3.93,11.35) | 5.06 (-1.24,11.37) | -0.22 (-6.57,6.12) | 4.67 (-2.20,11.55) | 5.21 (-5.55,15.96) | TRQ |  |  |
| -4.59 (-12.60,3.41) | -1.99 (-10.22,6.24) | -0.63 (-7.63,6.37) | -5.91 (-12.96,1.13) | -1.02 (-8.54,6.50) | -0.49 (-11.67,10.69) | -5.69 (-13.21,1.82) | XYP |  |
| -0.75 (-8.07,6.57) | 1.85 (-5.71,9.42) | 3.21 (-2.99,9.42) | -2.07 (-8.32,4.18) | 2.82 (-3.97,9.61) | 3.36 (-7.34,14.06) | -1.85 (-8.63,4.93) | 3.84 (-3.59,11.28) | XZL |

NOTE: **SFJD**: Shufeng Jiedu Capsule; **XZL**: Fresh Bamboo Juice Oral Liquid; **QQHT**: Qingqi Huatan Pill; **QKPC**: Qingke Pingchuan Granule; **TRQ**: Tanreqing Injection; **RDN**: Reduning Injection; **SWLDH**: Shiwei Longdanhua Capsule; **QKL**: Qingkailing Injection; **XYP**: Xiyanping Injection.

Table S10.8: IL-6

The columns represent the comparison of the row drug class to the column drug class. The rows represent the comparison of the row drug class to the column drug class. The effect estimates are expressed as MD and 95% CI. For example, the MD in **IL-6** for SFJD compared to XZL is 18.54 (-6.11, 43.19). MD＜0 favors the drug in the column(yellow), and MD＞0 favors the drug in the row(pink).

| QKPC |  |  |  |  |  |  |  |  |
| --- | --- | --- | --- | --- | --- | --- | --- | --- |
| -1.91 (-61.64,57.82) | QQHT |  |  |  |  |  |  |  |
| -0.44 (-49.25,48.36) | 1.47 (-47.26,50.19) | RDN |  |  |  |  |  |  |
| 4.54 (-44.31,53.38) | 6.45 (-42.32,55.22) | 4.98 (-29.55,39.51) | SFJD |  |  |  |  |  |
| 23.42 (-25.50,72.35) | 25.33 (-23.51,74.18) | 23.87 (-10.77,58.51) | 18.89 (-15.81,53.58) | SWLDH |  |  |  |  |
| 3.37 (-49.33,56.08) | 5.28 (-47.35,57.92) | 3.82 (-35.99,43.63) | -1.16 (-41.02,38.69) | -20.05 (-60.00,19.90) | TRQ |  |  |  |
| -1.53 (-61.31,58.25) | 0.38 (-59.33,60.09) | -1.09 (-49.87,47.69) | -6.07 (-54.89,42.75) | -24.95 (-73.85,23.94) | -4.90 (-57.59,47.78) | XYP |  |  |
| 23.91 (-25.01,72.83) | 25.82 (-23.02,74.67) | 24.36 (-10.28,58.99) | 19.38 (-15.32,54.07) | 0.49 (-34.30,35.28) | 20.54 (-19.41,60.49) | 25.44 (-23.45,74.34) | XZL |  |
| -2.87 (-62.60,56.86) | -0.96 (-60.63,58.71) | -2.43 (-51.15,46.30) | -7.41 (-56.17,41.36) | -26.29 (-75.14,22.55) | -6.24 (-58.88,46.39) | -1.34 (-61.05,58.37) | -26.78 (-75.62,22.05) | ZCL |

NOTE: **SFJD**: Shufeng Jiedu Capsule; **XZL**: Fresh Bamboo Juice Oral Liquid; **QQHT**: Qingqi Huatan Pill; **QKPC**: Qingke Pingchuan Granule; **TRQ**: Tanreqing Injection; **RDN**: Reduning Injection; **SWLDH**: Shiwei Longdanhua Capsule; **ZCL**: Zhichuanling Injection; **XYP**: Xiyanping Injection.

Table S10.9: IL-8

The columns represent the comparison of the row drug class to the column drug class. The rows represent the comparison of the row drug class to the column drug class. The effect estimates are expressed as MD and 95% CI. For example, the MD in **IL-8** for SFJD compared to XZL is 0.20 (-9.81, 10.21). MD <0 favors the drug in the column(yellow), and MD >0 favors the drug in the row(pink).

| QKL |  |  |  |  |
| --- | --- | --- | --- | --- |
| -3.37 (-11.68,4.94) | QQHT |  |  |  |
| 5.19 (-5.10,15.48) | 8.56 (-1.61,18.72) | RDN |  |  |
| 0.68 (-8.32,9.68) | 4.05 (-4.79,12.89) | -4.51 (-15.24,6.23) | SFJD |  |
| -0.17 (-10.53,10.20) | 3.20 (-7.03,13.43) | -5.35 (-17.26,6.55) | -0.85 (-11.64,9.95) | XZL |

NOTE: **SFJD**: Shufeng Jiedu Capsule; **XZL**: Fresh Bamboo Juice Oral Liquid; **QQHT**: Qingqi Huatan Pill; **RDN**: Reduning Injection; **QKL**: Qingkailing Injection.

Table S10.10: TNF-α

The columns represent the comparison of the row drug class to the column drug class. The rows represent the comparison of the row drug class to the column drug class. The effect estimates are expressed as MD and 95% CI. For example, the MD in **TNF-α** for SFJD compared to XZL is -1.52 (-6.15, 3.12). MD <0 favors the drug in the column(yellow), and MD >0 favors the drug in the row(pink).

| QKL |  |  |  |  |  |  |
| --- | --- | --- | --- | --- | --- | --- |
| 5.05 (-1.54,11.65) | QQHT |  |  |  |  |  |
| -1.75 (-8.10,4.61) | -6.80 (-13.78,0.18) | RDN |  |  |  |  |
| 4.09 (-1.14,9.31) | -0.97 (-6.95,5.01) | 5.83 (0.11,11.55) | SFJD |  |  |  |
| 4.86 (-3.57,13.29) | -0.19 (-9.10,8.72) | 6.61 (-2.12,15.34) | 0.78 (-7.18,8.74) | SWLDH |  |  |
| 3.67 (-2.80,10.13) | -1.39 (-8.47,5.69) | 5.41 (-1.44,12.27) | -0.42 (-6.26,5.42) | -1.20 (-10.01,7.62) | TRQ |  |
| 2.24 (-3.24,7.72) | -2.81 (-9.03,3.40) | 3.98 (-1.97,9.94) | -1.85 (-6.59,2.90) | -2.63 (-10.76,5.51) | -1.43 (-7.50,4.65) | XZL |

NOTE: **SFJD**: Shufeng Jiedu Capsule; **XZL**: Fresh Bamboo Juice Oral Liquid; **QQHT**: Qingqi Huatan Pill; **TRQ**: Tanreqing Injection; **RDN**: Reduning Injection; **SWLDH**: Shiwei Longdanhua Capsule; **QKL**: Qingkailing Injection.

Table S10.11:Adverse event

The columns represent the comparison of the row drug class to the column drug class. The rows represent the comparison of the row drug class to the column drug class. The effect estimates are expressed as RR and 95% CI. For example, the RR in **Adverse event** for SFJD compared to XZL is 0.57 (0.22, 1.45). RR＜1 favors the drug in the column(yellow), and RR＞1 favors the drug in the row(pink).

| QKL |  |  |  |  |  |  |  |  |  |
| --- | --- | --- | --- | --- | --- | --- | --- | --- | --- |
| 1.94 (0.28,13.46) | QKPC |  |  |  |  |  |  |  |  |
| 1.28 (0.21,7.59) | 0.66 (0.21,2.04) | QQHT |  |  |  |  |  |  |  |
| 1.90 (0.28,12.91) | 0.98 (0.26,3.71) | 1.49 (0.50,4.45) | RDN |  |  |  |  |  |  |
| 1.91 (0.31,11.65) | 0.99 (0.31,3.17) | 1.50 (0.62,3.65) | 1.01 (0.32,3.13) | SFJD |  |  |  |  |  |
| 1.23 (0.18,8.49) | 0.63 (0.16,2.46) | 0.96 (0.31,2.96) | 0.65 (0.17,2.44) | 0.64 (0.20,2.05) | SWLDH |  |  |  |  |
| 0.95 (0.10,9.06) | 0.49 (0.08,2.91) | 0.75 (0.15,3.74) | 0.50 (0.09,2.91) | 0.50 (0.10,2.56) | 0.78 (0.13,4.57) | TRQ |  |  |  |
| 0.44 (0.03,7.52) | 0.23 (0.02,2.71) | 0.35 (0.03,3.66) | 0.23 (0.02,2.73) | 0.23 (0.02,2.49) | 0.36 (0.03,4.26) | 0.46 (0.03,7.08) | XYP |  |  |
| 1.08 (0.18,6.60) | 0.56 (0.17,1.80) | 0.85 (0.35,2.07) | 0.57 (0.18,1.78) | 0.57 (0.22,1.45) | 0.88 (0.28,2.81) | 1.13 (0.22,5.82) | 2.45 (0.23,26.44) | XZL |  |
| 1.16 (0.19,6.99) | 0.60 (0.19,1.89) | 0.91 (0.38,2.17) | 0.61 (0.20,1.87) | 0.61 (0.24,1.52) | 0.94 (0.30,2.96) | 1.21 (0.24,6.16) | 2.62 (0.25,28.06) | 1.07 (0.43,2.69) | ZCL |

NOTE: **SFJD**: Shufeng Jiedu Capsule; **XZL**: Fresh Bamboo Juice Oral Liquid; **QQHT**: Qingqi Huatan Pill; **QKPC**: Qingke Pingchuan Granule; **TRQ**: Tanreqing Injection; **RDN**: Reduning Injection; **SWLDH**: Shiwei Longdanhua Capsule; **ZCL**: Zhichuanling Injection; **QKL**: Qingkailing Injection; **XYP**: Xiyanping Injection.

**Appendix 11: Specific examples of Adverse events**

| Study | Intervention | Tremor  T/C | Headache  T/C | Tachycardia  T/C | Nausea and Vomiting  T/C | Anorexia  T/C | Diarrhea T/C | Rash  T/C | Blurred vision  T/C | Abnormal liver function  T/C | Xerostomia and Hoarseness  T/C | Insomnia  T/C |
| --- | --- | --- | --- | --- | --- | --- | --- | --- | --- | --- | --- | --- |
| Yang2021 | SFJD vs CT | 1/1 | 2/2 | 0/1 | 2/0 |  |  |  |  |  |  |  |
| He2020 | SFJD vs CT | 2/1 | 2/2 | 2/1 | 1/0 |  | 1/0 | 1/0 |  |  |  |  |
| Chen2021 | SFJD vs CT |  |  |  |  |  |  |  |  |  |  |  |
| Jiang2022 | SFJD vs CT |  | 2/3 | 1/1 |  |  |  | 1/3 |  |  |  |  |
| Tian2021 | SFJD vs CT |  |  |  | 1/4 |  | 0/1 | 0/1 |  |  |  |  |
| Yin2022 | SFJD vs CT |  |  |  |  |  |  |  |  |  |  |  |
| Tang2022 | SFJD vs CT |  |  |  |  |  |  |  |  |  |  |  |
| Wang2016 | SFJD vs CT |  |  |  |  |  |  |  |  |  |  |  |
| Zhang2015 | SFJD vs CT |  |  |  | 1/1 |  | 0/1 |  |  |  |  |  |
| Li2025 | SFJD vs CT |  |  |  |  |  |  |  |  |  |  |  |
| Yu2024 | SFJD vs CT |  |  |  |  |  |  |  |  |  |  |  |
| Chen2020 | SFJD vs CT |  |  |  |  |  |  |  |  |  |  |  |
| Zhang2019 | SFJD vs CT |  |  |  |  |  |  |  |  |  |  |  |
| Wang2015 | SFJD vs CT |  |  |  |  |  |  |  |  |  |  |  |
| Wang2020 | XZLvs CT |  |  |  | 2/1 | 0/1 | 1/0 |  |  |  |  |  |
| He2021 | XZLvs CT |  | 2/1 |  |  | 1/2 |  | 1/0 | 1/1 |  |  |  |
| Luo2020 | XZLvs CT |  |  |  |  |  |  |  |  |  |  |  |
| Wang2017 | XZLvs CT |  | 1/1 |  | 2/2 |  | 2/2 | 3/3 |  | 2/1 |  |  |
| Pei2019 | XZLvs CT |  |  |  |  |  |  |  |  |  |  |  |
| Zheng2016 | XZLvs CT |  |  |  |  |  |  |  |  |  |  |  |
| Li2024 | XZLvs CT |  |  | 0/1 | 2/2 |  |  |  |  |  | 3/1 |  |
| Wang2021 | QQHT vs CT |  | 6/4 | 5/4 | 7/8 |  |  |  |  |  |  |  |
| Hou2019 | QQHT vs CT |  | 0/2 |  |  |  | 1/1 |  |  |  |  |  |
| Jiang2019 | QQHT vs CT |  |  |  |  |  |  |  |  |  |  |  |
| Jiang2021 | QQHT vs CT |  | 0/1 |  |  |  | 2/1 |  |  |  |  |  |
| Liu2019 | QQHT vs CT |  | 0/1 |  |  |  | 1/1 |  |  |  |  |  |
| Wei2020 | QQHT vs CT |  |  |  |  |  |  |  |  |  |  |  |
| Ji2017 | QQHT vs CT |  |  |  |  |  |  |  |  |  |  |  |
| Qu2023 | QQHT vs CT |  | 1/1 |  | 1/0 |  |  |  |  |  | 1/1 |  |
| Wu2014 | QKPC vs CT |  |  |  |  |  |  |  |  |  |  |  |
| Cai2023 | QKPC vs CT |  |  | 2/5 | 2/2 |  |  | 2/2 |  |  |  |  |
| Yu2024 | QKPC vs CT |  |  |  |  | 2/2 |  |  |  |  |  |  |
| Liu2024 | QKPC vs CT |  |  |  |  |  |  |  |  |  |  |  |
| Li2009 | TRQ vs CT |  |  |  |  |  |  |  |  |  |  |  |
| Liu2012 | TRQ vs CT |  |  |  |  |  |  |  |  |  |  |  |
| Yu2019 | TRQ vs CT |  |  |  |  |  |  |  |  |  |  |  |
| Xiang2022 | TRQ vs CT |  |  |  |  |  |  | 1/0 |  |  |  |  |
| Zhang2024 | TRQ vs CT |  |  |  |  |  |  |  |  |  |  |  |
| Zhang2006 | TRQ vs CT |  |  |  |  |  |  |  |  |  |  |  |
| Hong2008 | TRQ vs CT |  |  |  |  |  |  |  |  |  |  |  |
| Zhang2010 | TRQ vs CT |  | 1/0 |  |  |  |  |  |  |  |  |  |
| Yao2020 | TRQ vs CT |  |  |  |  |  |  |  |  |  |  |  |
| Rao2012 | RDN vs CT |  |  |  |  |  |  |  |  |  |  |  |
| Sun2012 | RDN vs CT |  |  |  |  |  |  |  |  |  |  |  |
| Ma2020 | RDN vs CT |  |  |  |  |  |  |  |  |  |  |  |
| Yu2022 | RDN vs CT |  |  |  |  |  |  |  |  |  |  |  |
| Shao2023 | RDN vs CT |  | 1/2 |  | 2/5 |  |  | 1/3 |  |  |  |  |
| Tang2021 | RDN vs CT |  |  |  |  |  |  |  |  |  |  |  |
| Pang2015 | RDN vs CT |  |  |  |  |  |  |  |  |  |  |  |
| Zeng2014 | RDN vs CT |  |  |  |  |  |  |  |  |  |  |  |
| Chen2014 | RDN vs CT |  |  |  |  |  |  |  |  |  |  |  |
| Lu2018 | RDN vs CT |  | 2/1 |  |  |  |  |  |  |  |  |  |
| Wang2013 | RDN vs CT |  |  |  |  |  |  |  |  |  |  |  |
| Wei2014 | RDN vs CT |  |  |  |  |  |  |  |  |  |  |  |
| Zhou2014 | RDN vs CT |  | 0/2 |  |  |  |  |  |  |  |  |  |
| Peng2021 | RDN vs CT |  |  |  |  |  |  |  |  |  |  |  |
| Sun2022 | SWLDH vs CT |  |  |  |  |  |  |  |  |  |  |  |
| Fu2021 | SWLDH vs CT |  |  |  |  |  |  |  |  |  |  |  |
| Liu2020 | SWLDH vs CT |  | 0/1 |  | 5/2 |  |  |  |  |  |  | 2/2 |
| Peng2019 | SWLDH vs CT |  |  |  |  |  |  |  |  |  |  |  |
| Zhang2015 | SWLDH vs CT |  |  | 1/2 | 0/1 |  | 3/0 |  |  |  |  |  |
| Lei2019 | SWLDH vs CT |  |  |  |  |  |  |  |  |  |  |  |
| Re2022 | ZCL vs CT |  | 1/1 |  | 2/1 |  |  |  |  |  |  |  |
| Zhang2017 | ZCL vs CT |  |  |  |  |  |  |  |  |  |  |  |
| Zhang2025 | ZCL vs CT |  | 2/1 |  |  | 2/2 | 2/1 |  |  |  | 1/1 |  |
| Zhao2024 | ZCL vs CT |  |  |  | 8/6 |  | 5/4 | 3/3 |  |  |  |  |
| Zeng2009 | QKL vs CT |  |  |  |  |  |  |  |  |  |  |  |
| Sun2001 | QKL vs CT |  |  |  |  |  |  |  |  |  |  |  |
| Ding2013 | QKL vs CT |  |  |  |  |  |  |  |  |  |  |  |
| Li2016 | QKL vs CT |  |  |  |  |  |  |  |  |  |  |  |
| Cao2012 | QKL vs CT |  |  |  |  |  |  | 1/0 |  |  |  |  |
| Chang2006 | QKL vs CT |  |  |  |  |  |  |  |  |  |  |  |
| Yang2009 | QKL vs CT |  |  |  |  |  |  |  |  |  |  |  |
| He2008 | QKL vs CT |  |  |  |  |  |  |  |  |  |  |  |
| Xie2022 | XYP vs CT |  |  |  |  |  |  |  |  |  |  |  |
| Zeng2018 | XYP vs CT |  |  |  |  |  |  |  |  |  |  |  |
| Liang2012 | XYP vs CT |  |  |  |  |  |  |  |  |  |  |  |
| Chang2015 | XYP vs CT |  |  |  |  |  |  |  |  |  |  |  |
| Zhang2014 | XYP vs CT |  |  |  |  |  | 1/0 |  |  |  |  |  |
| Han2012 | XYP vs CT |  |  |  |  |  |  |  |  |  |  |  |
| Zhang2011 | XYP vs CT |  |  |  |  |  |  |  |  |  |  |  |
| Liu2016 | XYP vs CT |  |  |  |  |  |  |  |  |  |  |  |
| Zhang2017 | XYP vs CT |  |  |  |  |  |  |  |  |  |  |  |
| Han2015 | XYP vs CT |  |  |  |  |  |  |  |  |  |  |  |

NOTE: **SFJD**: Shufeng Jiedu Capsule; **XZL**: Fresh Bamboo Juice Oral Liquid; **QQHT**: Qingqi Huatan Pill; **QKPC**: Qingke Pingchuan Granule; **TRQ**: Tanreqing Injection; **RDN**: Reduning Injection; **SWLDH**: Shiwei Longdanhua Capsule;**ZCL**: Zhichuanling Injection; **QKL**: Qingkailing Injection; **XYP**: Xiyanping Injection; **CT**: Conventional biomedicine treatment.

**Appendix 12: Sensitivity analyses**

The sensitivity analysis was conducted by excluding all high-risk-of-bias trials from the 84 clinical studies.

Table S12.1: Sensitivity analyses of primary outcome

| CPMs | Total effective rate | | FVC | | FEV1 | | FEV1/FVC | |
| --- | --- | --- | --- | --- | --- | --- | --- | --- |
|  | Main estimate | Sensitivity analyses | Main estimate | Sensitivity analyses | Main estimate | Sensitivity analyses | Main estimate | Sensitivity analyses |
| SFJD | 3.14 (2.19,4.50) | 3.13 (2.17,4.52) | 0.35 (0.23,0.47) | 0.35 (0.26,0.44) | 0.41 (0.30,0.52) | 0.41 (0.31,0.51) | 8.17 (5.25,11.09) | 8.68 (5.45,11.90) |
| XZL | 3.62 (2.31,5.68) | 3.75 (2.29,6.15) | 0.10 (-0.11,0.30) | 0.01 (-0.20,0.22) | 0.25 (0.11,0.38) | 0.22 (0.08,0.36) | 5.78 (1.98,9.58) | 6.23 (1.86,10.60) |
| QQHT | 4.06 (2.46,6.68) | 4.06 (2.46,6.68) | -0.31(-0.54,-0.08) | -0.31 (-0.46,-0.16) | 0.20 (-0.00,0.39) | 0.20 (0.01,0.38) | 2.96 (-0.83,6.74) | 2.97 (-0.99,6.93) |
| QKPC | 4.18 (1.82,9.59) | 4.18 (1.82,9.59) | 0.06 (-0.26,0.38) | 0.06 (-0.21,0.33) | 0.12 (-0.19,0.43) | 0.12 (-0.18,0.42) | 4.97 (-4.85,14.79) | 4.97 (-5.25,15.19) |
| TRQ | 2.91 (1.70,4.97) | 2.91 (1.70,4.97) | 0.27 (0.07,0.46) | 0.24 (0.10,0.39) | 0.21 (0.07,0.35) | 0.21 (0.08,0.34) | 4.40 (0.12,8.68) | 4.43 (-0.04,8.90) |
| RND | 4.19 (2.86,6.15) | 3.95 (2.63,5.93) | 0.35 (0.17,0.53) | 0.35 (0.21,0.50) | 0.28 (0.17,0.39) | 0.26 (0.15,0.37) | 5.58 (2.09,9.07) | 5.75 (1.82,9.68) |
| SWLDH | 1.99 (1.20,3.28) | 1.99 (1.20,3.28) | 0.58 (0.29,0.87) | 0.58 (0.35,0.81) | 0.85 (0.56,1.15) | 0.85 (0.58,1.13) | 2.86 (-1.21,6.93) | 2.86 (-1.40,7.12) |
| ZCL | 4.26 (2.24,8.09) | 4.26 (2.24,8.09) | 0.47 (0.24,0.70) | 0.47 (0.29,0.66) | 0.70 (0.47,0.92) | 0.70 (0.49,0.91) | 0.66 (-4.83,6.16) | 0.71 (-5.03,6.45) |
| QKL | 4.09 (2.27,7.34) | 4.01 (2.14,7.52) | 0.28 (0.13,0.43) | 0.32 (0.16,0.47) | 0.34 (0.19,0.50) | 0.32 (0.14,0.51) | 8.57 (3.80,13.34) | 9.09 (3.25,14.92) |
| XYP | 3.31 (1.95,5.61) | 3.73 (1.87,7.43) | 0.28 (0.11,0.44) | 0.44 (0.29,0.59) | 0.27 (0.13,0.40) | 0.36 (0.21,0.50) | 3.76 (-0.41,7.92) | 5.04 (-0.55,10.63) |

NOTE: **SFJD**: Shufeng Jiedu Capsule; **XZL**: Fresh Bamboo Juice Oral Liquid; **QQHT**: Qingqi Huatan Pill; **QKPC**: Qingke Pingchuan Granule; **TRQ**: Tanreqing Injection; **RDN**: Reduning Injection; **SWLDH**: Shiwei Longdanhua Capsule; **ZCL**: Zhichuanling Injection; **QKL**: Qingkailing Injection; **XYP**: Xiyanping Injection.
